# The Neural Mechanism of Motion Sickness Alleviation by Anticipatory Sound

**DOI:** 10.1038/s41467-026-77950-x

**Published:** 2026-09-24

**Authors:** Panpan Wan, Jie Cao, Lifang Huo, Xingxing Lai, Zhimin Ye, Yanyan Zhang, Congping Shang

**Affiliations:** 1 Guangzhou Medical University, Guangzhou, China; 2 Guangzhou National Laboratory, Guangzhou, China

**Keywords:** Neural circuits, Sensory processing

## Abstract

Motion sickness (MS) is a common response to conflicting motion signals and can cause nausea, dizziness, malaise, and reduced activity. Sound can lessen MS, but how auditory cues influence the brain circuits that generate these symptoms is unclear. Here we show, using a repeated rotation paradigm in male mice, that a brief anticipatory tone reduces MS-like responses. We identify Cerebellin-2-expressing (Cbln2^+^) glutamatergic neurons in the medial vestibular nuclei (MVN), a brainstem center for balance signals, as key drivers of these responses. These neurons act through two separable outputs: a projection to the ventrolateral periaqueductal gray that promotes MS-like behavior, and a projection to the nucleus of the solitary tract that contributes to nausea-related aversion, likely through the parabrachial nucleus. Anticipatory tone cues recruit an inhibitory pathway from the central amygdala to suppress Cbln2^+^ MVN neurons. These findings reveal how auditory prediction can dampen vestibular circuits that drive MS.

## Abstract (web summary)

The neural basis of motion sickness is not fully understood. Here, the authors show that anticipatory sound reduces motion sickness-like responses in mice by activating an inhibitory brain pathway that dampens balance-processing neurons and separates movement symptoms from nausea-related aversion.

## Introduction

Motion sickness (MS) is a complex physiological syndrome triggered by provocative motion conditions such as altered gravitoinertial forces, off-vertical axis rotation, centripetal acceleration, and spaceflight. Depending on the exposure context, it is commonly referred to as seasickness, carsickness, airsickness, space MS, or simulator sickness^1–3^. In humans, MS typically manifests as nausea, vomiting, pallor, cold sweating, dizziness, malaise, and drowsiness^4–6^, whereas in rodents it is more often assessed using MS-like readouts such as hypolocomotion, hypothermia, hypophagia, and other nausea-related behaviors^7–9^. The dominant explanatory framework is the sensory conflict theory, which proposes that MS arises when the brain receives mismatched motion and spatial-orientation signals from the vestibular, visual, and somatosensory/proprioceptive systems^1,7,10,11^.

Understanding the neural basis of MS is important for developing more effective preventive strategies. Current countermeasures include pharmacological treatment^12,13^, non-pharmacological (e.g., adaptive^14,15^) interventions, and other complementary therapies (e.g., acupressure^16^ and breath control^17^), but their efficacy is often incomplete and side effects can limit routine use. In this context, anticipatory sensory cueing^18,19^ has emerged as a potential strategy, because increasing motion predictability can reduce MS in some settings^20–22^. In particular, naïve anticipatory auditory cues have been shown to mitigate MS in controlled human paradigms^23–25^. A recent study in mice and humans also suggests that brief daily exposure to a low-frequency pure tone may alleviate motion-induced imbalance and autonomic dysregulation^26,27^. These findings raise the possibility of a peripheral auditory-vestibular interaction. To our knowledge, however, the central neural mechanisms by which auditory cues influence MS-related circuits remain largely unknown.

A large body of work has implicated the vestibular system in the generation of MS^7,28–30^. Vestibular dysfunction, postural imbalance, and vestibulo-autonomic dysregulation are all closely linked to MS susceptibility and symptom expression^28,31,32^. Within this framework, the vestibular nuclei, including the medial vestibular nucleus (MVN), are well positioned to transform head-motion signals into diverse physiological and behavioral outputs. They send extensive projections to brainstem and forebrain regions involved in autonomic, affective, and behavioral regulation, including the solitary tract (NTS), parabrachial nucleus (PBN), central amygdala (CeA), periaqueductal gray (PAG), and locus coeruleus (LC)^33–36^. Consistent with this role, recent studies have shown that activation of vestibular glutamatergic neurons can reproduce key MS-like responses in rodents, and that distinct vestibular subpopulations contribute to specific autonomic components of MS-like behavior^35,37,38^. For example, glutamatergic neurons in the medial vestibular nuclei parvocellular part (MVePC) drive motion-induced hypothermia^39^. In particular, cholecystokinin (Cck)-expressing vestibular neurons mediate multiple autonomic and nausea-related responses, in part through projections to the PBN^9^. However, the cell types and circuit pathways that govern other prominent motion-induced phenotypes, especially imbalance- and vertigo-like motor responses, remain poorly understood. In addition, whether and how anticipatory auditory cues modulate these vestibular circuits has not been investigated.

Here, we established a mouse model of MS and investigated the effects of anticipatory tone cues on repeated rotation-induced MS-like behavior. Guided by the single-cell expression data from the MVN, we then performed a candidate-based functional screen to identify specific neuronal subtypes and circuits that drive MS-like behavior. Finally, we sought to investigate how anticipatory auditory cues modulate these vestibular pathways. Our findings identify a specific MVN neuronal subpopulation that contributes to MS-like behavior in mice and provide a circuit-level framework for understanding how anticipatory auditory cues modulate MS-related responses.

## Results

### Paradigm for studying MS-like behavior

**Fig. 1:**
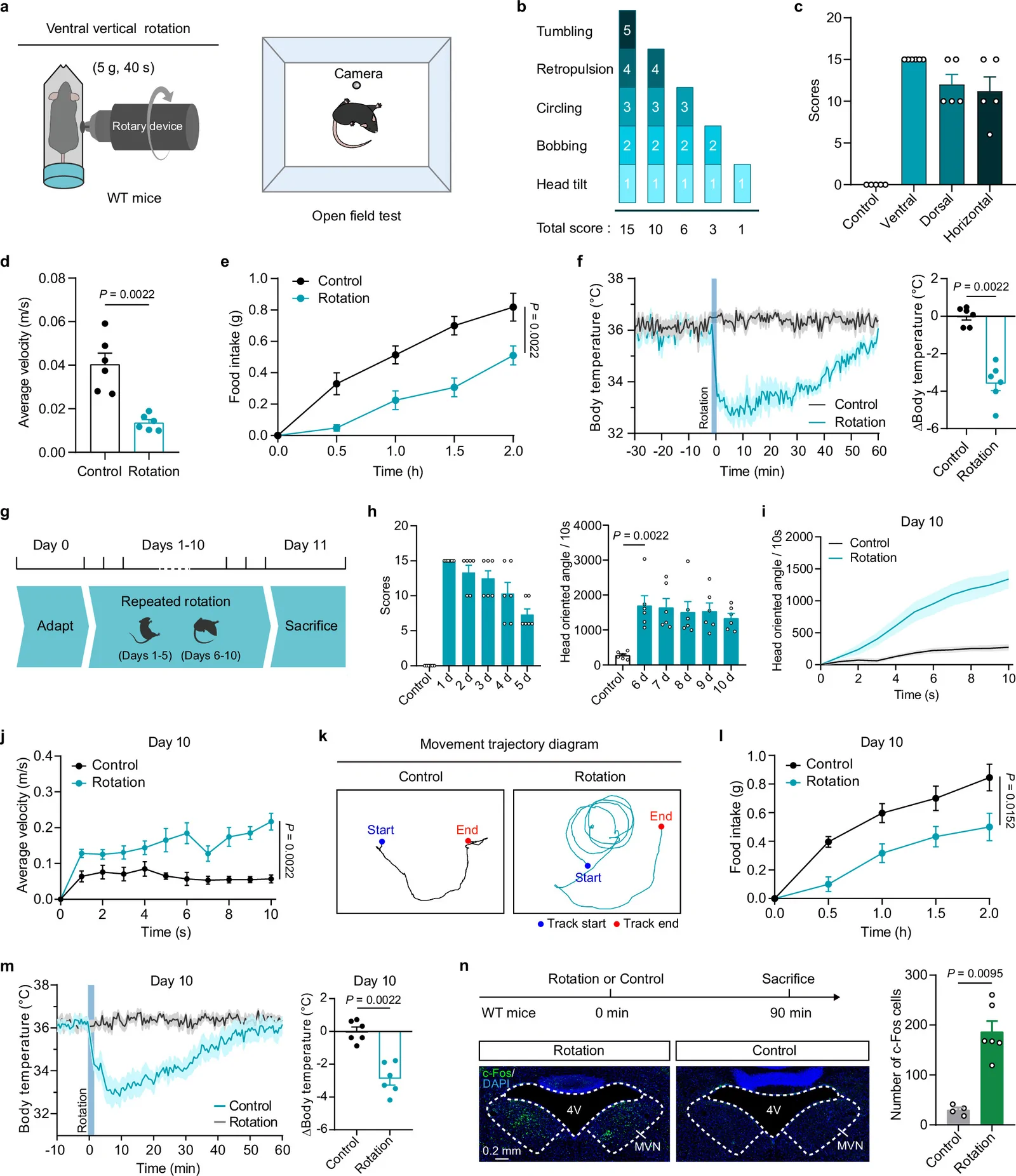
Experimental paradigm for measuring MS-like behavior in mice. **a** Schematic of the paradigm using 5 g ventral vertical rotation for 40 s in mice. **b** Scoring grid for evaluating MS-like symptoms. A score of 15 denotes all listed symptoms.**c** Scores during the first 10 s after ventral vertical (*n* = 6), dorsal vertical (*n* = 5), or horizontal rotation (*n* = 5); controls were not rotated (*n* = 5). Average velocity during a 10-min open-field test (**d**), cumulative food intake over 2 hours (**e**), and body temperature changes over 1 h and changes from baseline at 10 min (ΔT) (**f**) after rotation or control stimulation (*n* = 6 per group, *U* = 0, *P* = 0.0022).**g** Repeated rotation paradigm: 5 g ventral vertical rotation for 40 s daily for 10 days. **h** Scores and cumulative head-orientation angles during the first 10 s post-rotation across 10 days. (*n* = 6 per group, *U* = 0, *P* = 0.0022). Cumulative head-orientation angles (**i**), average velocity (**j**), and representative movement trajectories (**k**) during the first 10 s post-rotation on day 10 (*n* = 6 per group, *U* = 0, *P* = 0.0022). **l** Cumulative food intake on day 10 after repeated rotation or control stimulation (*n* = 6 per group, *U* = 3, *P* = 0.0125). **m** Body temperature changes over 1 hour and ΔT at 10 min on day 10 (*n* = 6 per group, *U* = 0, *P* = 0.0022).**n** Strategy for identifying MS-associated brain regions in WT mice. Representative MVN images show c-Fos immunostaining after rotation or control stimulation. c-Fos^+^ cells are quantified on the right (Rotation: *n* = 6, Control: *n* = 4; *U* = 0, *P* = 0.0095). Data are mean ± s.e.m.; error bars and shaded areas indicate s.e.m. Data in (**d**–**f**, **h**, **j** and **l**–**n** were analyzed using two-sided Mann-Whitney *U* tests. Scale bars are indicated in the images. Source data are provided as a Source Data file.

Previous studies have shown that rotational or gravitational stimulation can evoke behavioral and autonomic MS-like responses in rodents^35–37,40^. Motivated by recent evidence that vestibular Cck^+^ neurons contribute to motion-induced malaise^9^, we established a custom-built rotational paradigm to assess rotation-induced MS-like behavior in mice and compared different rotational configurations separately (Fig. 1a). Specifically, mice were subjected to ventral vertical, dorsal vertical, or horizontal rotation at 5 g for 40 s, whereas control mice underwent the same handling procedure without rotation. MS-like symptoms were then assessed using a behavioral evaluation score^38^ during the subsequent open-field test (Fig. 1b). Mice displayed transient MS-like symptoms to different extents immediately after rotation, with ventral vertical rotation producing the most overt tumbling phenotype (Fig. 1c). We next compared different intensities of ventral vertical rotation and found that 5 g stimulation elicited the most pronounced behavioral responses (Supplementary Fig. 1a), indicating that 5 g ventral vertical rotation produced the strongest MS-like behavioral responses under our experimental conditions. In addition to these acute behavioral abnormalities, mice subjected to ventral vertical rotation also exhibited significant reductions in locomotor activity, food intake, and body temperature compared with controls (rotation versus control, −3.55 ± 0.42 °C versus 0 ± 0.20 °C; Fig. 1d-f), consistent with previously described MS-like responses in rodents^5,36,39,41^. Subsequent calcium imaging analysis further confirmed our choice of ventral vertical rotation as the most appropriate paradigm.

We next establish a repeated rotation-induced MS-like model by exposing mice to daily rotational stimulation for 10 consecutive days (Fig. 1g). Repeatedly rotated mice exhibited a progressive change in behavioral phenotype, evolving from tumbling and retropulsive symptoms during days 1–5 to transient directed circling behavior during days 6–10 (Fig. 1h and Supplementary Movie 1), which was then followed by prolonged hypoactivity. This progressive behavioral adaptation is consistent with the well-recognized habituation of MS following repeated motion exposure^1,42–44^. To quantify the prominent transient circling phenotype observed from days 6–10, we measured the cumulative head-orientation angle during the first 10 s post-rotation. Relative to controls, rotated mice displayed a significant increase in cumulative head-orientation angle (Fig. 1h, i). In addition, average velocity during the first 10 s post-rotation was increased on day 10, and representative movement traces showed more pronounced circling behavior in the rotation group (Fig. 1j, k). Repeatedly rotated mice also exhibited sustained reductions in food intake and body temperature compared with controls (Fig. 1l, m). These results demonstrate that repeated rotation induced both classical MS-like physiological changes and a reproducible circling phenotype in mice.

Here, to identify candidate brain regions activated by rotational stimulation, we next performed *c-Fos* immunostaining. Compared with control stimulation, rotational stimulation increased *c-Fos* expression most prominently in the MVN (Fig. 1n). Increased *c-Fos* labeling was also observed in the NTS, CeA, PBN, and ventrolateral periaqueductal gray (vlPAG), albeit to a lesser extent (Supplementary Fig. 1b, c). Overall, we established a repeated rotation-induced mouse model of MS-like behavior and identified the MVN as a prominently activated candidate node associated with the MS-like behavior.

### Anticipatory tone attenuates repeated rotation-induced MS-like behavior

**Fig. 2:**
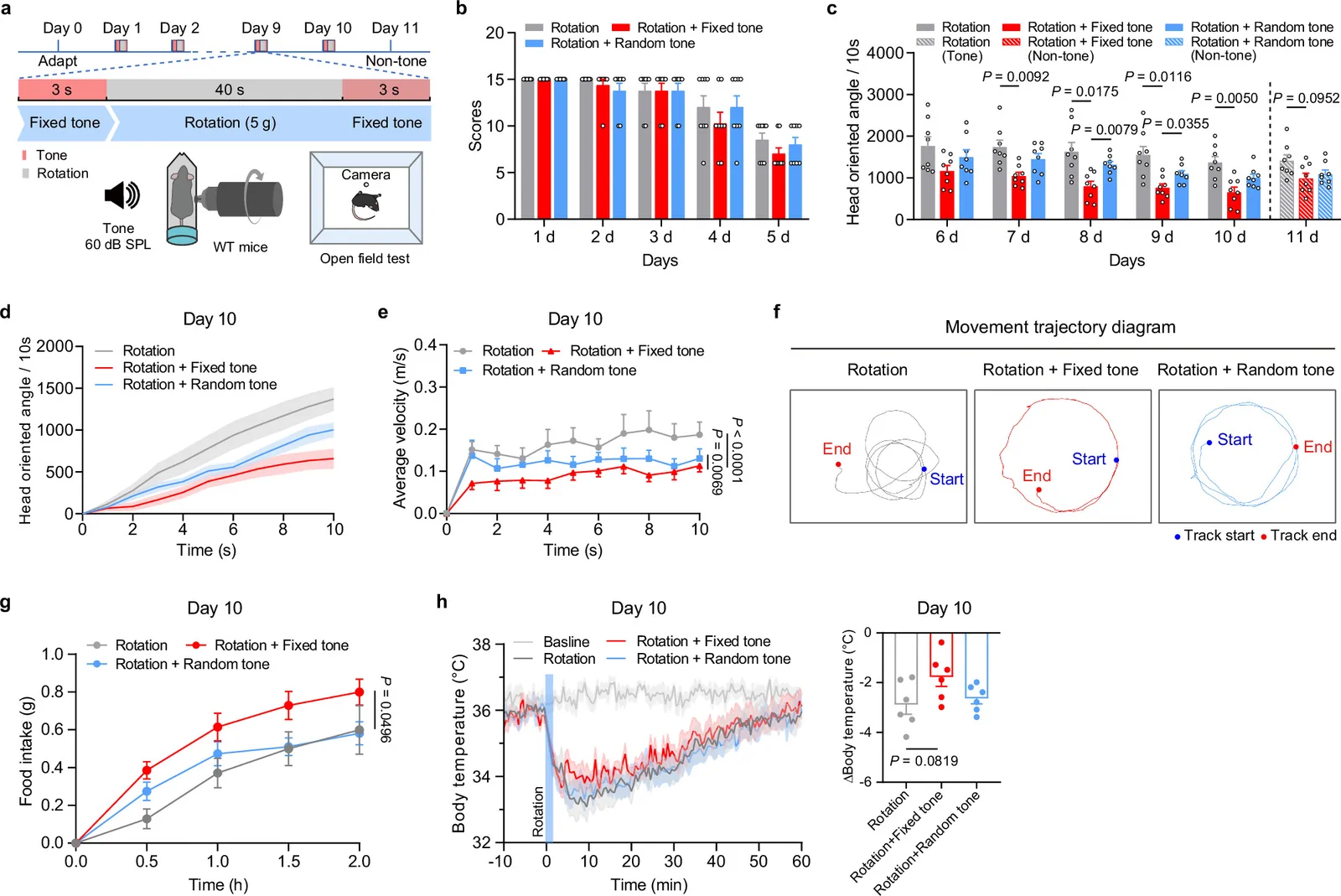
Anticipatory tone attenuates repeated rotation-induced MS-like behavior. **a** Experimental timeline and tone intervention. Mice underwent 5 g ventral vertical rotation for 40 s daily for 10 consecutive days, with or without tone pairing. In the Rotation + Fixed tone group, a 3 s white noise cue at 60 dB sound pressure level (SPL) was presented immediately before rotation onset and offset. In the Rotation + Random tone group, which served as an auditory-exposure control for the Fixed tone group, two 3 s white noise cues were delivered at random, unpredictable times during the 40 s rotation period. Behavioral evaluation scores (**b**) and cumulative head orientation angles (**c**) during the first 10 s of the post-rotation open-field test across 10 days (*n* = 8 per group). Cumulative head-orientation angles (**d**) and average velocity (**e**) during the first 10 s of the post-rotation open-field test on day 10 (*n* = 8 per group). **f** Representative movement trajectories from one mouse per group during the same 10 s period on day 10. **g** Cumulative food intake in hungry mice on day 10 after rotation with or without tone pairing (*n* = 7 per group). **h** Body temperature changes over 1 h and ΔT at 10 min on day 10 following rotation with or without tone pairing (*n* = 6 per group). Data are presented as mean values ± s.e.m.; error bars and shaded areas indicate s.e.m. Data in (**c**) and (**e**) were analyzed using two-way repeated-measures ANOVA with the Geisser-Greenhouse correction, followed by Tukey’s multiple-comparisons test. Data in (**g**) and (**h**) were analyzed using one-way ANOVA followed by Tukey’s multiple-comparisons test. All post hoc comparisons were two-sided. ANOVA statistics are provided in Supplementary Table 4, and adjusted *P* values are shown in the figure. Source data are provided as a Source Data file.

Anticipatory auditory cues, including music and noise, have been shown to affect MS by regulating vestibular function^23,26,45^. Inspired by these findings, we investigated whether anticipatory tone cues modulate repeated rotation-induced MS-like behavior. Since intense noise (above ~75 dB sound pressure levels, SPL) reliably elicits escape and anxiety behavior in mice^46,47^, we used a mild tone (~60 dB SPL) delivered through a speaker placed close to the animals, with ambient noise maintained at ~40 dB. Mice were subjected to daily rotational stimulation for 10 consecutive days paired with tone cues (Fig. 2a). Rotated mice that received fixed-tone training (anticipatory tone), but not random-tone training (non-anticipatory tone), exhibited significantly reduced cumulative head-orientation angles and average velocity compared to rotated mice exposed only to ambient noise on days 6-10 (Fig. 2c–e and Supplementary Fig. 2a). By contrast, no overt differences in behavioral evaluation scores were observed among the three groups during days 1-5 (Fig. 2b). To further test whether this effect depended on anticipatory cueing, on day 11, we removed the tone from the Rotation+Fixed tone and Rotation+Random tone groups, while presenting the anticipatory tone to the Rotation alone group. Under these conditions, the reduction in cumulative head-orientation angle was no longer observed relative to the Rotation group (Fig. 2c and Supplementary Fig. 2a), indicating that anticipatory tone cues actively modulated MS-like behavior. Representative movement trajectories of the three groups following rotation on day 10 are shown (Fig. 2f). We next examined whether anticipatory tone cues also influenced autonomic MS-like responses, such as appetite and body temperature. Mice that received fixed-tone training exhibited improved food intake compared with the other two groups, whereas body temperature was not significantly altered (Fig. 2g, h; and Supplementary Fig. 2b, c). Together, these results demonstrate that anticipatory tone cues attenuate repeated rotation-induced MS-like behavior in mice.

### Cbln2^+^ MVN neurons are a key neuronal subtype for rotation-induced MS-like behavior

**Fig. 3:**
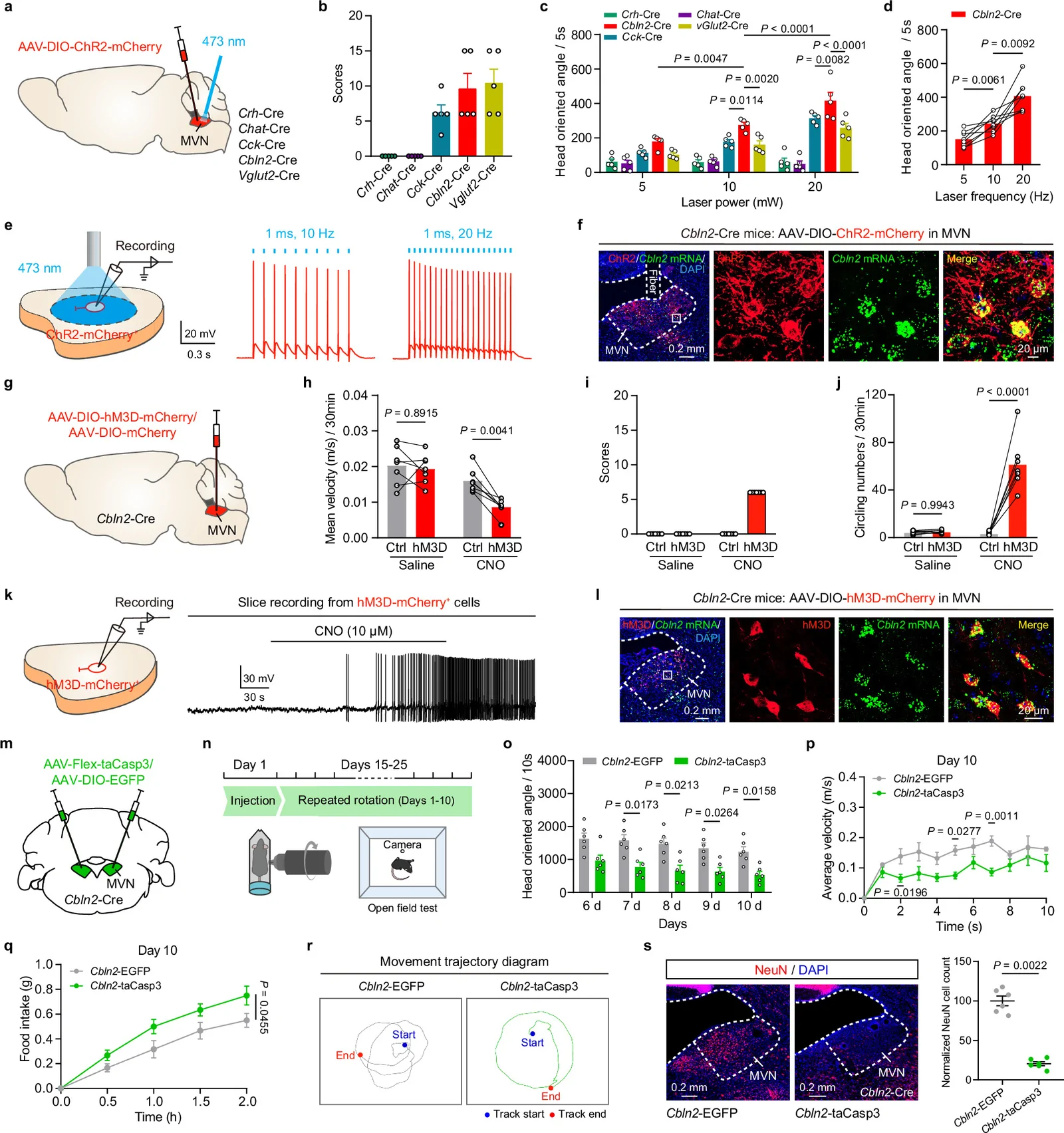
Cbln2^+^ MVN neurons contribute to rotation-induced MS-like behavior. **a** Viral strategy for Cre-dependent ChR2-mCherry expression and fiber implantation in the MVN across Cre lines. **b** Behavioral scores during 5 s photostimulation in indicated Cre lines (*n* = 5 per line). **c** Cumulative head-orientation angles across Cre lines; power responses in *Cbln2*-IRES-Cre mice were assessed at 5-20 mW (20 Hz; *n* = 5 per group). **d** Cumulative head-orientation angles at 5-20 Hz in *Cbln2*-IRES-Cre mice (20 mW; *n* = 8). Representative light-evoked phase-locked spiking activity in a ChR2-expressing Cbln2^+^ MVN neuron (**e**) and ChR2-mCherry expression, fiber placement, and colocalization with *Cbln2* mRNA (**f**). **g** Viral strategy for hM3Dq-mCherry or Control mCherry expression in Cbln2^+^ MVN neurons. CNO activation of Cbln2^+^ neurons reduced average velocity (**h**), increased scores (**i**), and induced circling (**j**) within 30 min (1 mg/kg; *n* = 7 per group). Representative CNO-evoked firing in an hM3Dq-expressing neuron (**k**) and hM3Dq-mCherry expression with *Cbln2* mRNA colocalization (**l**). Viral strategy for Cbln2^+^ MVN neuron ablation (**m**) and repeated-rotation testing after ablation (**n**). Cumulative head-orientation angles on days 6–10 (**o**), average velocity (**p**), and food intake (**q**) on day 10 after ablation (*n* = 6 per group). **r** Representative movement trajectories during the first 10 s post-rotation on day 10. **s** Representative sections and summary of the number of MVN neurons (*n* = 6 per group). Data are mean ± s.e.m.; error bars indicate s.e.m. Data in (**c**) and (**d**) were analyzed using two-way and one-way ANOVA, respectively, followed by two-sided Tukey’s multiple-comparisons test. Data in (**h**), (**j**), (**o**), and (**p**) were analyzed using two-way repeated-measures ANOVA followed by two-sided Sidak’s multiple-comparisons test, and data in (**q**) and (**s**) were analyzed using two-sided Mann-Whitney *U* tests. ANOVA statistics are provided in Supplementary Table 4, and adjusted and exact *P* values are shown in the figure. Scale bars are indicated in the images. Source data are provided as a Source Data file.

Having established an effective rotation paradigm, we next sought to identify the MVN neuronal subpopulations that contribute to rotation-induced MS-like behavior. Previous studies have shown that glutamatergic, but not GABAergic, vestibular neurons are critically involved in vestibular responses to rotation, hypergravity, and postural imbalance^9,37,38^. Guided by previous single-cell expression profiling studies of the MVN^48^ and mRNA in situ hybridization data from the Allen Brain Atlas, we identified several marker-defined neuronal populations in the MVN, including Crh⁺, Chat⁺, Cck⁺, and Cbln2⁺ neurons. These populations showed detectable or enriched expression in the MVN and were selected as candidate populations potentially relevant to autonomic responses^49,50^. Cck⁺ neurons were also included because recent work provides evidence that CCK-expressing vestibular neurons are involved in motion-induced malaise^9^. To identify MVN key excitatory neuronal subtypes that mediate rotation-induced MS-like behavior, we optogenetically activated these marker-defined populations in the corresponding Cre mouse lines, using *Vglut2*-IRES-Cre mice as a positive control for excitatory vestibular neurons. Specifically, we injected AAV-DIO-ChR2-mCherry into the MVN of these five Cre mouse lines and implanted optical fibers above the MVN (Fig. 3a). Allen Brain Atlas in situ hybridization data supported the expression of these marker genes in the MVN (Supplementary Fig. 3a), and immunostaining confirmed accurate viral targeting within the MVN (Supplementary Fig. 3b). Among the tested populations, optogenetic activation of Cbln2^+^ neurons induced most pronounced MS-like symptoms, except for Vglut2^+^ neurons, reaching a behavioral score of 9 and a cumulative head-orientation angle of 416 ± 31 during the 5 s stimulation period (Fig. 3b, c; and Supplementary Movie 2). Laser-induced cumulative head-orientation angles of MS-like symptoms were correlated with laser power and frequency (Fig. 3c, d). Furthermore, the average velocity during the 5 s optogenetic activation of Cbln2^+^ neurons was also significantly greater than that of other neuronal subtypes (Supplementary Fig. 3c, d). In acute MVN slices, a light pulse train (473 nm, 1 ms, 20 mW, 10 Hz or 20 Hz) reliably evoked phase-locked spiking activity in Cbln2^+^ neurons expressing ChR2-mCherry (Fig. 3e). In situ hybridization result showed that ChR2-mCherry expression was primarily distributed within the MVN, and that the majority of mCherry^+^ cells (84% ± 4.8%, *n* = 4 mice) co-expressed *Cbln2* mRNA (Fig. 3f and Supplementary Fig. 3e). These results indicate that optogenetic activation of Cbln2^+^ MVN neurons recapitulates major features the rotation-induced MS-like behavioral phenotype.

Consistently, we employed chemogenetics to achieve sustained activation of Cbln2^+^ MVN neurons (Fig. 3g). Intraperitoneal injection of CNO (1 mg/kg) in mice expressing hM3Dq-mCherry in Cbln2^+^ MVN neurons triggered obvious hypolocomotion and hypothermia compared to controls (Fig. 3h and Supplementary Fig. 3f, g), resembling the MS-like responses observed following a single rotation. Notably, chemogenetic activation of Cbln2^+^ neurons also elicited MS-like circling symptoms, with circling up to 62 ± 6 times/30 min (Fig. 3i, j; and Supplementary Movie 3). Given that optogenetic activation of Cck^+^ neurons also evoked MS-like symptoms, we compared the effects of chemogenetic activation of Cbln2^+^ and Cck^+^ neurons. Activation of Cck^+^ neurons expressing hM3Dq-mCherry produced similarly pronounced hypothermia and hypolocomotion (Supplementary Fig. 3f–h). However, Cck^+^ neuron activation elicited significantly fewer circling episodes than Cbln2^+^ neuron activation, with mice instead displaying predominantly body tottering (Supplementary Fig. 3i and Supplementary Movie 4). RNAscope analysis showed minimal colocalization between *Cbln2* mRNA and H2B-EGFP^+^ cells in *Cck*-IRES-Cre mice (3.8% ± 3.0%, *n* = 3 mice), implying that *Cck* and *Cbln2* define different excitatory neuronal subpopulations within the MVN (Supplementary Fig. 3j, k). Additionally, in acute slice physiology, CNO (10 *μ*M) increased action potential firing in Cbln2^+^ MVN neurons expressing hM3Dq-mCherry, confirming effective chemogenetic activation (Fig. 3k). In situ hybridization successfully validated the cell-type specificity of hM3Dq-mCherry expression in Cbln2^+^ MVN neurons (Fig. 3l and Supplementary Fig. 3l). These results indicate that Cbln2^+^ neurons make a strong contribution to rotation-induced MS-like symptoms.

To examine whether Cbln2^+^ MVN neurons are required for rotation-induced MS-like behavior, we bilaterally injecting AAV-Flex-taCasp3 or AAV-DIO-EGFP into the MVN of *Cbln2*-IRES-Cre mice to ablate this population (Fig. 3m). Two weeks after viral expression, the mice underwent repeated rotational stimulation for 10 consecutive days (Fig. 3n). Of note, ablation of Cbln2^+^ MVN neurons exhibited significant suppression of MS-like behavior, including decreased behavioral evaluation scores, cumulative head-orientation angles, and average velocity, relative to the EGFP (non-apoptosis) group (Fig. 3o, p; and Supplementary Fig. 4a–c). Importantly, ablation of Cbln2^+^ MVN neurons also improved appetite on day 10 (Fig. 3q and Supplementary Fig. 4d). Representative movement trajectories on day 10 are shown (Fig. 3r). We further quantified the extent of ablation by counting the number of surviving neurons (Fig. 3s), and found that the number of surviving neurons was significantly correlated with cumulative head-orientation angles on day 10 (Supplementary Fig. 4e). These findings underscore that Cbln2^+^ MVN neurons as a major neuronal population required for rotation-induced MS-like behavior.

### Physiological properties of Cbln2^+^ MVN neurons

**Fig. 4:**
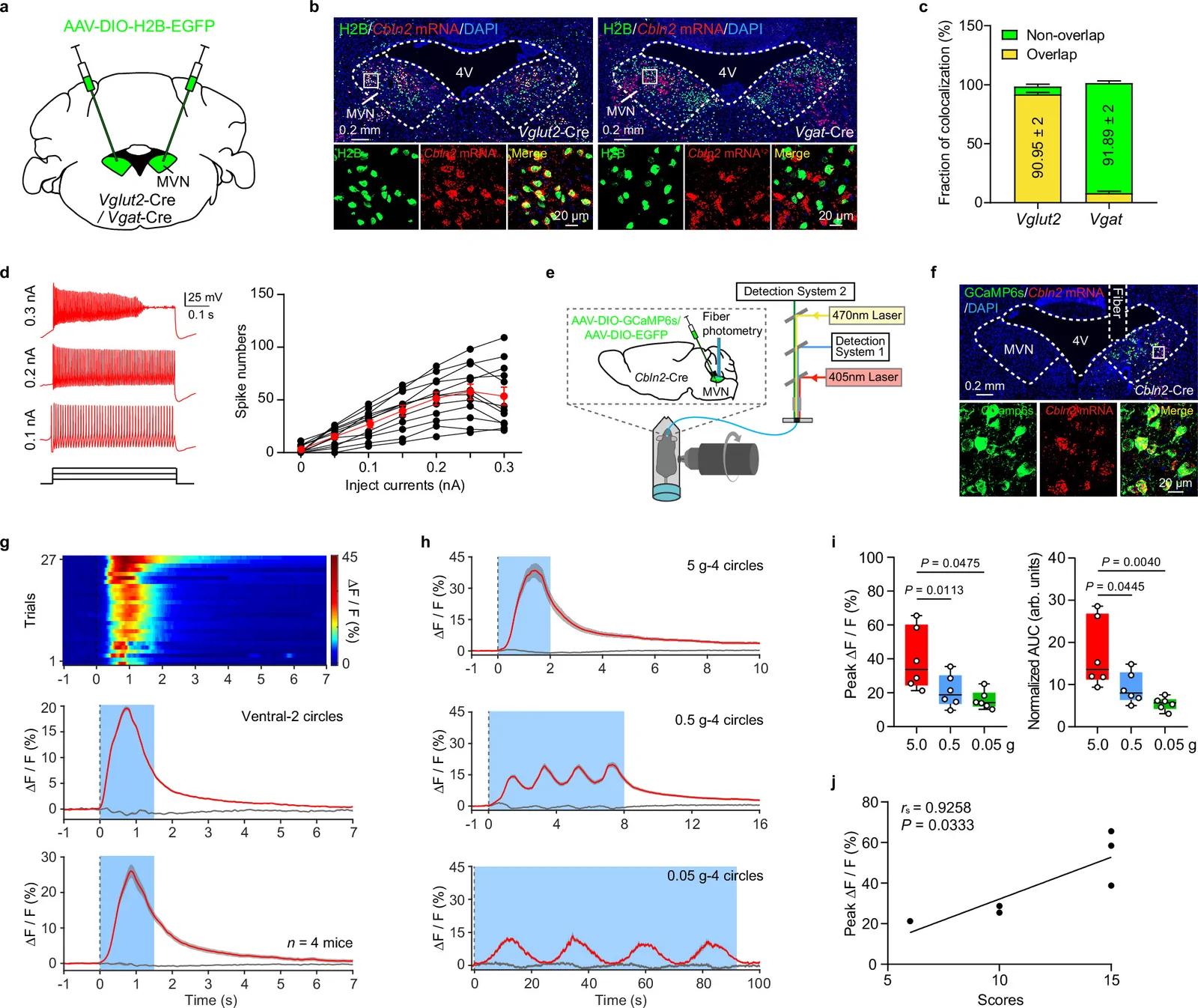
Physiological properties and rotation-evoked activity of Cbln2^+^ MVN neurons. **a** Cre-dependent H2B-EGFP expression in the MVN of *Vglut2*-IRES-Cre and *Vgat*-IRES-Cre mice. Representative MVN sections showing *Cbln2* mRNA expression in H2B-EGFP^+^ neurons from *Vglut2*-IRES-Cre and *Vgat*-IRES-Cre mice (**b**), and quantification of the percentages of H2B-EGFP^+^ neurons expressing *Cbln2* mRNA in each genotype (**c**; *n* = 3 mice per genotype). **d** Representative current-evoked firing and spike numbers across current steps in Cbln2^+^ MVN neurons. Black lines represent individual cells; the red line and error bars indicate mean ± s.e.m. **e** GCaMP6s or EGFP expression and fiber implantation in the MVN of *Cbln2*-IRES-Cre mice.**f** Representative optical fiber placement and GCaMP6s^+^ neurons colocalized with *Cbln2* mRNA. **g** Trial-by-trial GCaMP6s activity in Cbln2^+^ MVN neurons aligned to rotation onset (5 g, 2 cycles), shown as a heatmap of 27 trials from 4 mice (top), mean responses for individual mice (middle), and the population average (bottom; *n* = 4 mice). **h** GCaMP6s responses to 5, 0.5, and 0.05 g rotations (4 circles, *n* = 6 mice). Red and gray traces indicate GCaMP6s and EGFP signals, respectively. Thick traces and shaded regions denote mean ± s.e.m. Dashed lines mark rotation onset, and blue shading indicates the rotation period. **i** Peak amplitude and time-normalized AUC of GCaMP responses at different intensities (*n* = 6 mice). Box plots show the median, 25th-75th percentiles, minimum-to-maximum whiskers, and individual mice. **j** Association between GCaMP6s responses to rotation (5.0 g, 4 circles) and MS-like symptom severity (*n* = 6 mice; two-sided Spearman rank correlation).Data are mean ± s.e.m.; error bars and shaded areas indicate s.e.m. Data in (**i**) were analyzed using one-way ANOVA followed by two-sided Tukey’s multiple-comparisons test. ANOVA statistics are provided in Supplementary Table 4, and adjusted *P* values are shown in the figure. Scale bars are indicated in the images. Source data are provided as a Source Data file.

To determine the molecular characteristics of Cbln2^+^ MVN neurons, we first injected AAV-DIO-H2B-EGFP into the MVN of *Vglut2*-IRES-Cre and *Vgat*-IRES-Cre mice and then performed in situ hybridization for *Cbln2* mRNA (Fig. 4a). Colocalization analysis revealed that the majority of H2B-EGFP^+^ MVN neurons in *Vglut2*-IRES-Cre mice were positive for *Cbln2* mRNA (91% ± 2%, *n* = 3 mice), whereas in *Vgat*-IRES-Cre mice, only a small fraction of H2B-EGFP^+^ neurons expressed *Cbln2* mRNA (8% ± 2%, *n* = 3 mice) (Fig. 4b, c). These results indicate that Cbln2^+^ MVN neurons are predominantly glutamatergic rather than GABAergic. In acute MVN slices, EGFP-labeled Cbln2^+^ neurons exhibited spiking activity with a slow adaptive pattern to depolarizing current injections after labeling Cbln2^+^ neurons with AAV-DIO-EGFP in *Cbln2*-IRES-Cre mice (Fig. 4d).

To monitor the activity of Cbln2^+^ neurons during rotation-induced MS-like behavior, we injected AAV-DIO-GCaMP6s or AAV-DIO-EGFP into the MVN and implanted optical fibers above the MVN of *Cbln2*-IRES-Cre mice, resulting in the specific expression of GCaMP6s in Cbln2^+^ MVN neurons (Fig. 4e, f and Supplementary Fig. 5a). Fiber photometry recordings were then record population calcium dynamics from Cbln2^+^ MVN neurons in mice during the rotation paradigm. Clear transient increases in GCaMP fluorescence were observed in Cbln2^+^ MVN neurons expressing GCaMP6s at the onset of 5 g ventral vertical rotation over two circles (Fig. 4g and Supplementary Movie 5). In contrast, no stimulus-locked fluorescence changes were detected in the EGFP-expressing control mice under the same conditions (Fig. 4g). Moreover, quantitative analysis revealed that ventral vertical rotation tended to evoke higher GCaMP signals than dorsal vertical rotation, as reflected by increased peak amplitude and unit area under the curve (AUC), although these differences were not statistically significant (Supplementary Fig. 5b, c). We next asked whether Cbln2^+^ MVN neuron activity scales with ventral vertical rotation intensity. During ventral vertical rotations over four circles, calcium responses increased progressively across 0.05 g, 0.5 g, and 5 g stimulation conditions (Fig. 4h, i). Furthermore, Cbln2^+^ neuron activity during the 5 g condition was correlated with the severity of MS-like behavioral symptoms (Fig. 4j).

Given that Cck^+^ neurons have previously been implicated in MS-like behavior induced by rotation combining horizontal and vertical forces, we further compared calcium responses in Cck^+^ and Cbln2^+^ MVN neurons during both ventral vertical and horizontal (a previously reported^9^) rotation. Quantitative analysis revealed that both the peak amplitude and unit AUC of GCaMP signals in Cck^+^ neurons were substantially lower than those in Cbln2^+^ neurons under either stimulus condition (Supplementary Fig. 6a–c). Notably, in Cbln2^+^ neurons, ventral vertical rotation evoked a significantly larger GCaMP peak amplitude than horizontal rotation, providing further evidence for the validity of our ventral vertical rotation paradigm in inducing MS-like behavior in mice (Supplementary Fig. 6a–c). These findings suggest that Cbln2^+^ MVN neurons encode rotation-induced MS-like responses in an intensity-dependent manner and are preferentially engaged during ventral vertical rotation.

### Cbln2^+^ MVN-vlPAG pathway is required for rotation-induced MS-like behavior

**Fig. 5:**
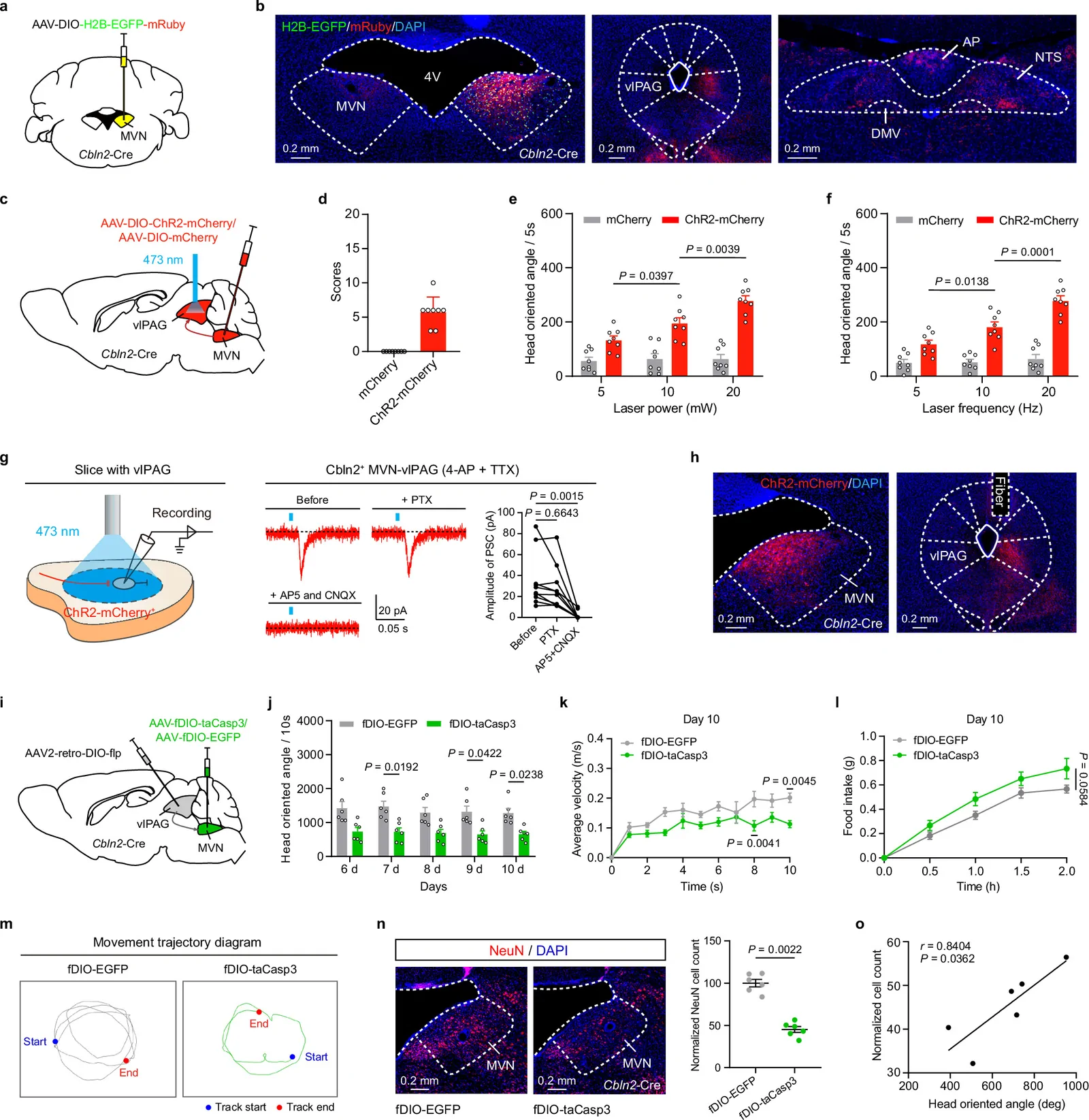
The Cbln2^+^ MVN-vlPAG pathway contributes to rotation-induced MS-like behavior. Cre-dependent H2B-EGFP-synaptophysin-mRuby expression in the MVN of *Cbln2*-IRES-Cre mice (**a**) and representative mRuby^+^ projections in the vlPAG and NTS (**b**). **c** Schematic of optogenetic activation of Cbln2^+^ MVN axon terminals in the vlPAG. **d** Behavioral scores during 5 s optogenetic activation of the MVN-vlPAG pathway (*n* = 8 per group). Cumulative head orientation angles during 5 s optogenetic activation of the MVN-vlPAG pathway at laser power (**e**; 5–20 mW, 20 Hz) and laser frequency (**f**; 5–20 Hz, 20 mW) (*n* = 8 per group). **g** Recordings from vlPAG neurons during photostimulation of ChR2-expressing Cbln2^+^ MVN terminals. Light-evoked PSCs were blocked by PTX and D-AP5/CNQX (*n* = 10 cells from 4 mice). **h** Representative ChR2-mCherry expression in the MVN and fiber placement above terminals in the vlPAG. **i** Viral injection strategy for taCasp3-mediated ablation of vlPAG-projecting Cbln2^+^ MVN neurons. Cumulative head-orientation angles on days 6–10 (**j**), average velocity (**k**) and food intake (**l**) on day 10 after repeated rotation in mice with or without pathway ablation (*n* = 6 per group). **m** Representative movement trajectories during the first 10 s post-rotation on day 10.**n** Representative sections and summary of the number of MVN neurons (*n* = 6 per group).**o** Association between the number of surviving MVN neurons and cumulative head-orientation angles on day 10 (*n* = 6 mice; two-sided Pearson correlation, *r* = 0.8404, 95% CI 0.0907−0.9821, *P* = 0.0362). Data are mean ± s.e.m.; error bars indicate s.e.m. Data in (**e**), (**f**), (**j**), and (**k**) were analyzed using two-way repeated-measures ANOVA followed by two-sided Sidak’s multiple-comparisons test, and data in (**g**) using one-way ANOVA followed by two-sided Dunnett’s multiple-comparisons test. Data in (**l**) and (**n**) were analyzed using two-sided Mann-Whitney U tests. ANOVA statistics are provided in Supplementary Table 4, and adjusted and exact *P* values are shown in the figure. Scale bars are indicated in the images. Source data are provided as a Source Data file.

Next, we hypothesized that specific projections of Cbln2^+^ MVN neurons likely mediate rotation-induced MS-like responses. To investigate the anterograde targets of these neurons, we mapped the projections by injecting AAV-DIO-H2B-EGFP-synaptophysin-mRuby into the MVN of *Cbln2*-IRES-Cre mice (Fig. 5a). As expected, abundant EGFP^+^ neuron cell bodies and mRuby^+^ axonal terminals were observed within the MVN (Fig. 5b). Dense mRuby^+^ axon terminals were detected in several downstream regions, including the vlPAG, NTS, interpeduncular nucleus (IPN) and caudate putamen (CPu) (Fig. 5b and Supplementary Fig. 7a). We further compared the downstream projections of Cck^+^ and Cbln2^+^ MVN neurons and found that the projection patterns of Cbln2^+^ neurons were distinct from those of Cck^+^ neurons (Supplementary Fig. 7b), suggesting that these two MVN subpopulations may influence different aspects of MS-like behavior through their specialized output pathways. Previous studies have reported that vlPAG is implicated in motor control and sensory modulation^51–54^, we hypothesized that the Cbln2^+^ MVN neurons may relay rotation-induced MS information to the vlPAG. To test this, we selectively optogenetically activated the Cbln2^+^ MVN-vlPAG pathway by injecting AAV-DIO-ChR2-mCherry or AAV-DIO-mCherry into the MVN and implanting optical fibers above the axon terminals in the vlPAG of *Cbln2*-IRES-Cre mice (Fig. 5c). Optogenetic activation of this pathway reliably evoked MS-like symptoms, producing a behavioral score of 6 and cumulative head-orientation angle of 279 ± 24 during 5 s of stimulation (Fig. 5d, e; and Supplementary Movie 6), similar to the phenotype elicited by activation of Cbln2^+^ MVN cell bodies. Laser-induced cumulative head-orientation angles of MS-like symptoms were correlated with laser power and frequency (Fig. 5e, f). Moreover, the average velocity during the 5 s stimulation period of this pathway in the ChR2-mCherry group was also significantly higher than the mCherry control group (Supplementary Fig. 8a). By contrast, selective activation of other downstream pathways, including Cbln2^+^ MVN projections to the NTS, IPN, or CPu, did not elicit comparably overt behavioral phenotypes (Supplementary Fig. 8b–d), highlighting a preferential role for the MVN-vlPAG circuit in mediating MS-like behavioral response. To examine synaptic connectivity, we recorded from vlPAG neurons in acute slices while photostimulating ChR2-mCherry^+^ axon terminals arising from Cbln2^+^ MVN neurons. Light pulses (473 nm, 2 ms, 20 mW) reliably evoked postsynaptic currents (PSCs) in vlPAG neurons (Fig. 5g), and these responses were abolished by CNQX and AP5, indicating glutamatergic transmission. Immunostaining revealed ChR2-mCherry expression in MVN cell bodies and dense axonal projections in the vlPAG (Fig. 5h).

We next investigated whether neurons constituting this pathway are required for rotation-induced MS-like behavior. To do so, we bilaterally injected AAV2-retro-DIO-flp into the vlPAG and AAV-fDIO-taCasp3 or AAV-fDIO-EGFP into the MVN of *Cbln2*-IRES-Cre mice (Fig. 5i). Three weeks after viral expression, the mice underwent repeated rotation stimulation for 10 consecutive days. Notably, ablation of the Cbln2^+^ MVN-vlPAG circuit largely reduced behavioral evaluation scores, cumulative head-orientation angles, and average velocity relative to non-ablated controls (Fig. 5j, k; and Supplementary Fig. 8e–g). However, pathway ablation did not significantly alter on appetite on day 10 compared to the EGFP control group (Fig. 5l and Supplementary Fig. 8h). Representative movement trajectories from both groups following rotation on day 10 are shown (Fig. 5m). We further quantified the extent of ablation by summarizing the number of surviving neurons (Fig. 5n), and found that the number of surviving neurons was significantly correlated with cumulative head-orientation angles on day 10 (Fig. 5o). Together, these results imply that the Cbln2^+^ MVN-vlPAG circuit is sufficient and required for the rotation-induced MS-like behavioral response.

### Cbln2^+^ MVN-NTS pathway mediates MS-related nausea

**Fig. 6:**
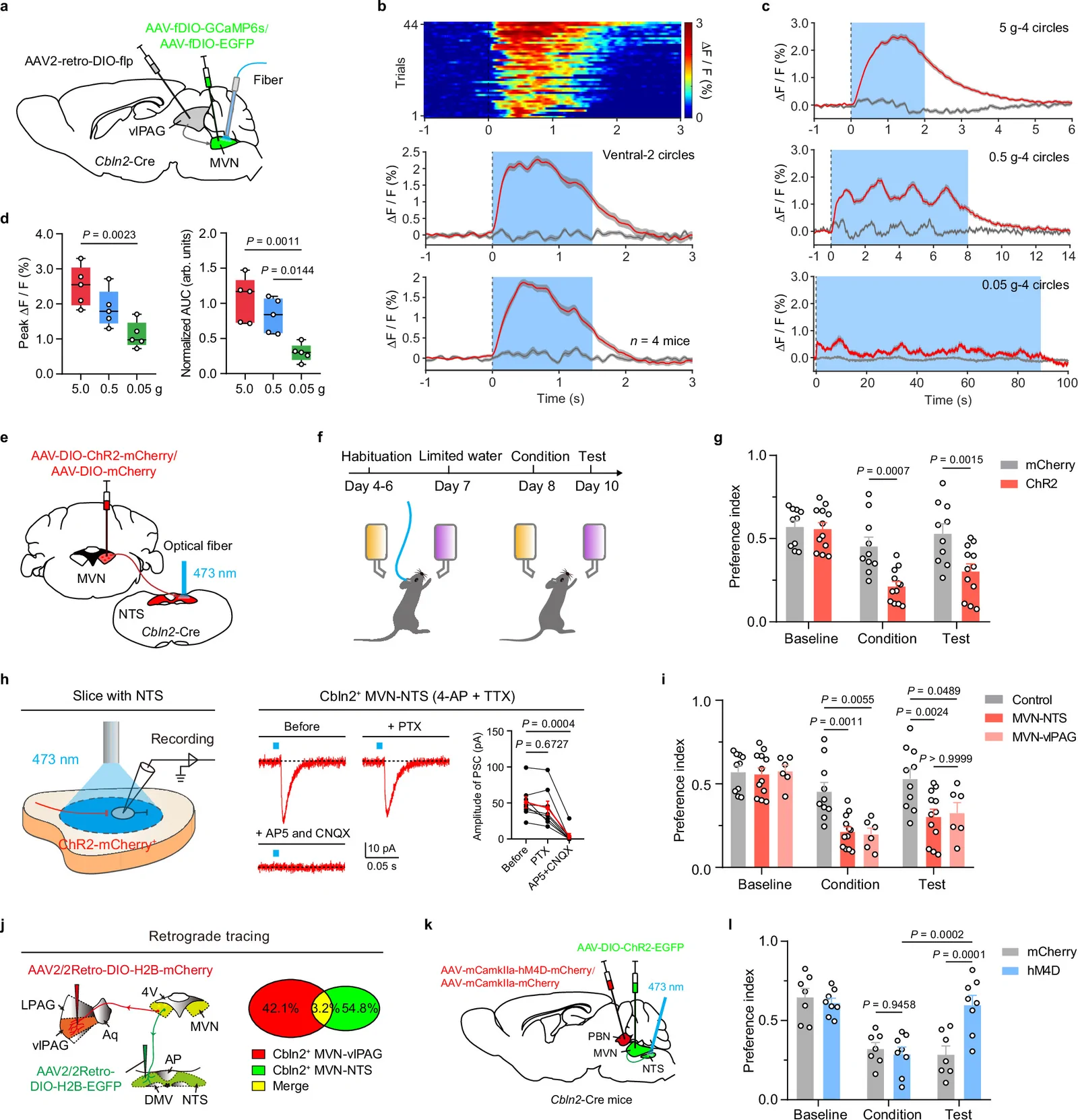
Functional dissection of Cbln2^+^ MVN projections to vlPAG and NTS in MS-like behavior and nausea. **a** GCaMP6s expression in vlPAG-projecting Cbln2^+^ MVN neurons and fiber implantation above the MVN. **b** Rotation-aligned GCaMP6s activity (5 g, 2 cycles), shown as a heatmap of 44 trials from 4 mice (top), individual-mouse traces (middle), and the population average (bottom; *n* = 4).**c** GCaMP6s responses to 5, 0.5, and 0.05 g rotations (4 circles, *n* = 5). Red and gray traces indicate GCaMP6s and EGFP signals. Thick traces and shading denote mean ± s.e.m. Dashed lines mark rotation onset. Blue shading indicates the rotation period. **d** Peak amplitude and time-normalized AUC at different intensities (*n* = 5). Box plots show the median, 25th-75th percentiles, minimum-to-maximum whiskers and individual mice. Schematic of optogenetic stimulation of MVN terminals in the NTS (**e**), conditioned flavor avoidance (CFA) protocol (**f**), and flavor preference following activation of the MVN-NTS pathway (**g**; mCherry: *n* = 10, ChR2: *n* = 12). **h** NTS-neuron recordings during stimulation of ChR2-expressing Cbln2^+^ MVN terminals. Light-evoked PSCs were blocked by PTX and D-AP5/CNQX (*n* = 10 cells from 3 mice). **i** CFA preference after MVN-NTS or MVN-vlPAG pathways optogenetic activation (Control: *n* = 10, MVN-NTS: *n* = 12, MVN-vlPAG: *n* = 6). **j** Dual retrograde tracing and fractions of NTS-projecting, vlPAG-projecting, and dual-projecting Cbln2^+^ MVN neurons (*n* = 3). **k** ChR2-EGFP expression in NTS-projecting Cbln2^+^ MVN neurons and hM4D-mCherry or mCherry expression in the PBN. **l** CFA preference after MVN-NTS pathway activation with or without PBN inhibition (mCherry: *n* = 7, hM4D: *n* = 8). Data are mean ± s.e.m.; error bars and shaded areas indicate s.e.m. Data in (**d**) and (**h**) were analyzed using one-way ANOVA followed by two-sided Tukey’s and Dunnett’s multiple-comparisons tests, respectively. Data in (**g**), (**i**), and (**l**) were analyzed using two-way ANOVA, followed by two-sided Sidak’s multiple-comparisons test. ANOVA statistics are provided in Supplementary Table 4, and adjusted *P* values are shown in the figure. Source data are provided as a Source Data file.

Since Cbln2^+^ MVN neurons showed pronounced responses during rotation-induced MS-like behavior, we hypothesized that vlPAG serves as a key downstream region that may process rotation-induced MS information. To test this hypothesis, we monitored the activity of the Cbln2^+^ MVN-vlPAG pathway during rotation-evoked MS-like behavior. We first injected AAV2-retro-DIO-flp into the vlPAG, AAV-fDIO-GCaMP6s or AAV-fDIO-EGFP into the MVN, and implanted optical fibers above the MVN of *Cbln2*-IRES-Cre mice (Fig. 6a), resulting in the specific expression of GCaMP6s in Cbln2^+^ MVN neurons that project to vlPAG (Supplementary Fig. 9a). At the onset of 5 g ventral vertical rotation over two circles, clear transient increases in GCaMP fluorescence were observed from mice expressing GCaMP6s in the Cbln2^+^ MVN-vlPAG pathway, whereas no stimulus-locked fluorescence changes were detected in the expressing EGFP controls (Fig. 6b). Quantitative analysis showed a trend toward greater GCaMP responses to ventral vertical rotation than to dorsal vertical rotation in both peak amplitude and unit AUC, although these differences did not reach statistical significance (Supplementary Fig. 9b, c). Moreover, during ventral vertical rotation over four circles, pathway activity increased progressively across 0.05 g, 0.5 g, and 5 g conditions (Fig. 6c, d). These findings reveal that the Cbln2^+^ MVN-vlPAG pathway is recruited by vertical rotation and encodes the intensity of ventral vertical rotation-induced MS-like responses.

Previous studies have confirmed that MS is linked to autonomic dysfunction, such as nausea and vomiting^5,31^. In previous results, we found abundant mRuby^+^ axon terminals of Cbln2^+^ MVN neurons in the NTS, a region in the brainstem that processes visceral sensory input and triggers nausea and vomiting reflexes^55,56^. Therefore, we hypothesized that the Cbln2^+^ MVN-NTS pathway may signal malaise and nausea triggered by MS-like behavior and establish conditioned flavor aversion (CFA), a typical paradigm used to assess nausea-related aversion^57,58^. To test this hypothesis, we performed optogenetic activation of the Cbln2^+^ MVN-NTS pathway in combination with CFA to determine whether this pathway promotes CFA (Fig. 6e, f). In the memory test phase, mice expressing ChR2-mCherry in this pathway exhibited significant conditioned aversion compared to mice expressing mCherry (Fig. 6g). In acute NTS slices, light pulses (473 nm, 2 ms, 20 mW) illuminating ChR2-mCherry^+^ axon terminals from the Cbln2^+^ MVN neurons evoked obvious PSCs in NTS neurons (Fig. 6h), and these responses were abolished by CNQX and AP5, confirming glutamatergic transmission. Immunostaining showed ChR2-mCherry expression in MVN cell bodies and axonal projections in the NTS (Supplementary Fig. 9d).

Given that optogenetic activation of axon terminals can in principle co-activate collaterals, and the vlPAG pathway also responded during MS-like behavior, we next examined whether selective optogenetic activation of the Cbln2^+^ MVN projections to the vlPAG promotes CFA. Interestingly, in the memory test phase, mice expressing ChR2-mCherry in the Cbln2^+^ MVN-vlPAG pathway also exhibited conditioned aversion compared to mice expressing mCherry (Fig. 6i). One possible explanation is that activation of this pathway-induced rotational movement may disturb drinking behavior, potentially confounding the assessment of conditioned aversion. To further assess pathway specificity, we applied retrograde tracing by injecting AAV2/2Retro-DIO-H2B-mCherry into the vlPAG and AAV2/2Retro-DIO-H2B-EGFP into the NTS of *Cbln2*-IRES-Cre mice, respectively. The results showed that retro-labeled Cbln2^+^ neurons were primarily distributed in the MVN, and only 3.2% of these neurons were double-labeled (Fig. 6j and Supplementary Fig. 9e), indicating that the two pathways arise largely from distinct Cbln2^+^ neuronal populations and are unlikely to be co-activated.

As the PBN is a classic brain region mediating malaise and CTA^58,59^, we hypothesized that activation of the Cbln2^+^ MVN-NTS pathway promotes CFA through its indirect relay to the PBN. To verify this, we optogenetically activated the Cbln2^+^ MVN-NTS pathway while chemogenetically inhibiting PBN excitatory neurons, and then performed CFA (Fig. 6k and Supplementary Fig. 9f). In the memory test stage, mice expressing hM4D-mCherry in the PBN exhibited significantly reduced CFA compared to mice expressing mCherry controls (Fig. 6l). In summary, these results suggest that the Cbln2^+^ MVN-NTS pathway contributes to nausea-/aversion-related component of MS-like behavior and likely does so through a downstream PBN-dependent circuit mechanism.

### Anticipatory tone alleviates rotation-evoked MS-like behavior by attenuating Cbln2^+^ MVN neuron activity

**Fig. 7:**
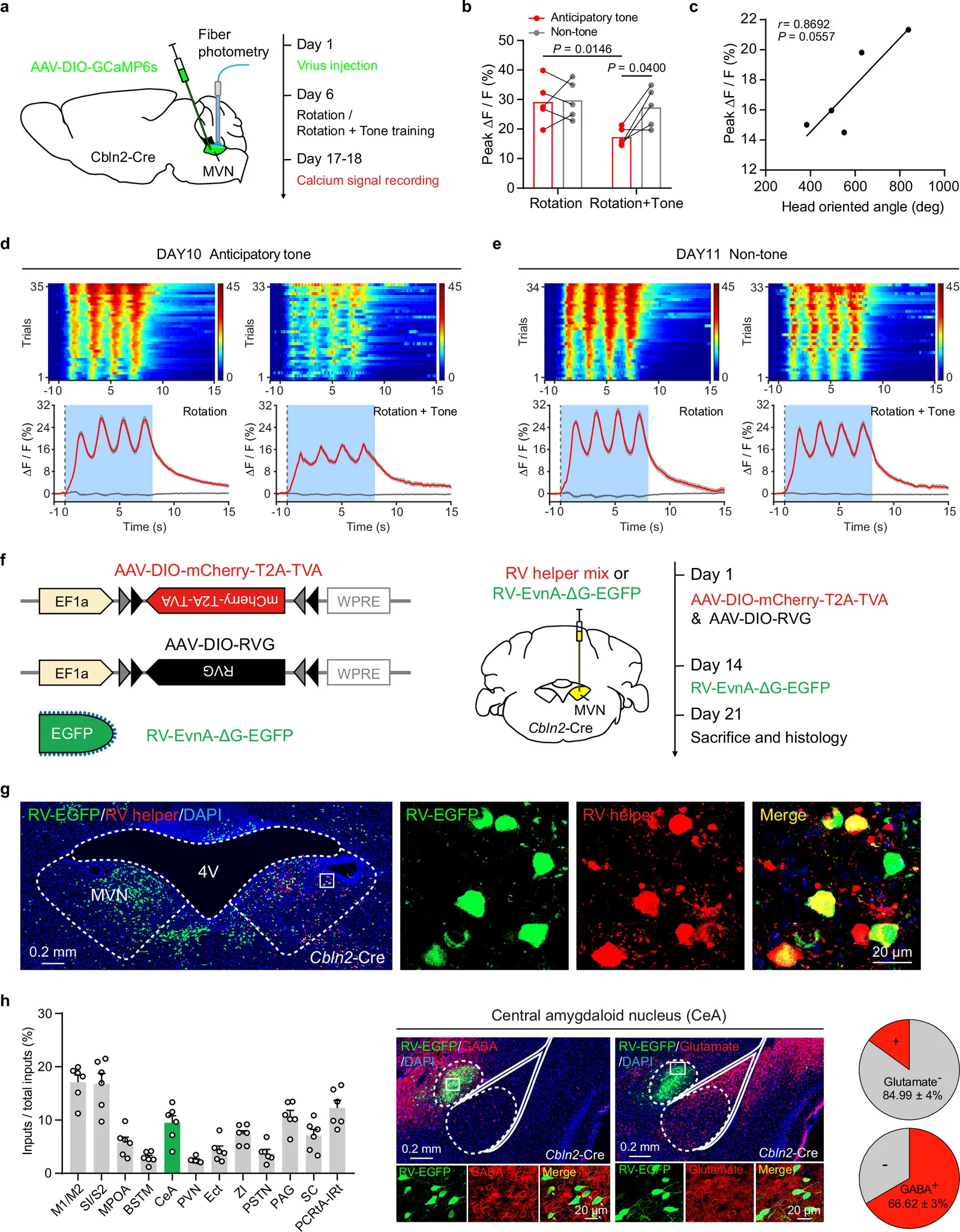
Anticipatory tone modulates rotation-induced MS-like behavior through MVN Cbln2^+^ neurons. **a** Schematic diagram showing GCaMP6s expression and optical fiber implantation in the MVN of *Cbln2*-IRES-Cre mice. **b** Quantitation of peak GCaMP responses in Cbln2^+^ MVN neurons during the second rotation cycle with or without anticipatory tone in Rotation+Tone and Rotation mice (*n* = 5 per group). **c** Association between peak GCaMP6s responses and cumulative head-orientation angles in Rotation+Tone group on day 10. (*n* = 5 mice; two-sided Pearson correlation, *r* = 0.8692, 95% CI −0.0561−0.9913, *P* = 0.0557). Rotation-aligned GCaMP6s activity in Cbln2^+^ MVN neurons on day 10 with tone presentation (**d**) and day 11 without tone presentation (**e**), shown as trial heatmaps of normalized fluorescence changes (ΔF/F) and group-mean traces (*n* = 5 per group). Red and gray traces indicate GCaMP6s and 405-nm control signals, respectively; thick traces and shaded regions denote mean ± s.e.m. Dashed lines mark rotation onset, and blue shading indicates the rotation period.**f** Strategy for monosynaptic retrograde tracing of Cbln2^+^ MVN neurons using AAV and rabies virus (RV) in the *Cbln2*-IRES-Cre mice. **g** Representative MVN sections showing dual-labeled starter cells in the *Cbln2*-IRES-Cre mice. **h** Regional distribution of monosynaptic RV-labeled inputs to Cbln2^+^ MVN neurons, expressed as the percentage of total EGFP^+^ input cells (*n* = 6 mice; left). Representative CeA images show EGFP^+^ neurons colocalized with glutamate or GABA (middle). Most retrogradely labeled EGFP^+^ CeA neurons were GABA^+^ and glutamate^-^ (*n* = 6 mice; right). Data are mean ± s.e.m.; error bars and shaded areas indicate s.e.m. Data in (**b**) were analyzed using two-way ANOVA, followed by two-sided Sidak’s multiple-comparisons test. ANOVA statistics are provided in Supplementary Table 4, and adjusted *P* values are shown in the figure. Scale bars are labeled in images. Source data are provided as a Source Data file.

The preceding results demonstrated that anticipatory tone training improves repeated rotation-induced MS-like behavior in mice. Our next question was how the anticipatory tone modulates this behavioral effect. We investigated whether Cbln2^+^ MVN neurons respond to such tone cues and contribute to this behavioral improvement. To address this, we utilized fiber photometry to record Ca2^+^ dynamics in Cbln2^+^ MVN neurons in mice that had undergone 10 days of repeated rotation training either with or without tone pairing (Fig. 7a). On day 10, both the Rotation alone and Rotation+Tone groups received tone presentation before rotation onset. Interestingly, the Rotation+Tone group exhibited significantly reduced GCaMP fluorescence in Cbln2^+^ MVN neurons relative to the Rotation group (Fig. 7b, d). In addition, the activity of Cbln2^+^ neurons was correlated with the severity of MS-like behavior in the Rotation+Tone group (Fig. 7c). On day 11, when both groups underwent rotation without tone presentation, there was no significant difference in GCaMP fluorescence between the two groups (Fig. 7b, e). However, within the Rotation+Tone group, GCaMP signals were significantly higher on day 11 (Non-tone) than on day 10 (tone present) (Fig. 7b). By contrast, tone exposure does not modulate Cbln2^+^ neurons' activity in the absence of rotation (Supplementary Fig. 10a). These results demonstrate that anticipatory tone training attenuates the rotation-induced activation of Cbln2⁺ MVN neurons, thereby alleviating MS-like behaviors.

To identify upstream circuits that might convey this anticipatory cue effect, we next mapped the afferent inputs to Cbln2^+^ MVN neurons using retrograde trans-synaptic tracing with recombinant rabies virus (RV). Cre-dependent helper AAVs were injected into the MVN of *Cbln2*-IRES-Cre mice, followed by RV-EGFP injection two weeks later (Fig. 7f). Retrogradely labeled EGFP^+^ neurons were observed in the MVN (Fig. 7g and Supplementary Fig. 10b), and further analysis revealed that Cbln2^+^ MVN neurons receive inputs from several brain areas, including preoptic area (POA), paraventricular thalamic nucleus (PVN), and central amygdaloid nucleus (CeA) (Fig. 7h and Supplementary Fig. 10c). The CeA is a well-established region implicated in attention, motivation, and associative processing^60–62^, and showed substantial connectivity with the Cbln2^+^ MVN neurons, we considered it a plausible candidate region for mediating tone-related modulation of MS-like behavior. To further characterize this input, we applied co-immunostaining for glutamate and GABA in retro-labeled EGFP^+^ neurons within the CeA, respectively. Quantitative analysis revealed that more than half of these neurons were GABAergic (67% ± 3%, *n* = 3 mice), whereas only a small fraction was glutamatergic (15% ± 4%, *n* = 3 mice) (Fig. 7h). These tracing data therefore identify a predominantly inhibitory CeA-MVN input as a candidate circuit that may contribute to the tone-induced attenuation of repeated rotation-induced MS-like behavior.

### CeA-MVN^Cbln2^ inhibitory pathway mediates the alleviation of rotation-induced MS-like behavior by anticipatory tone

**Fig. 8:**
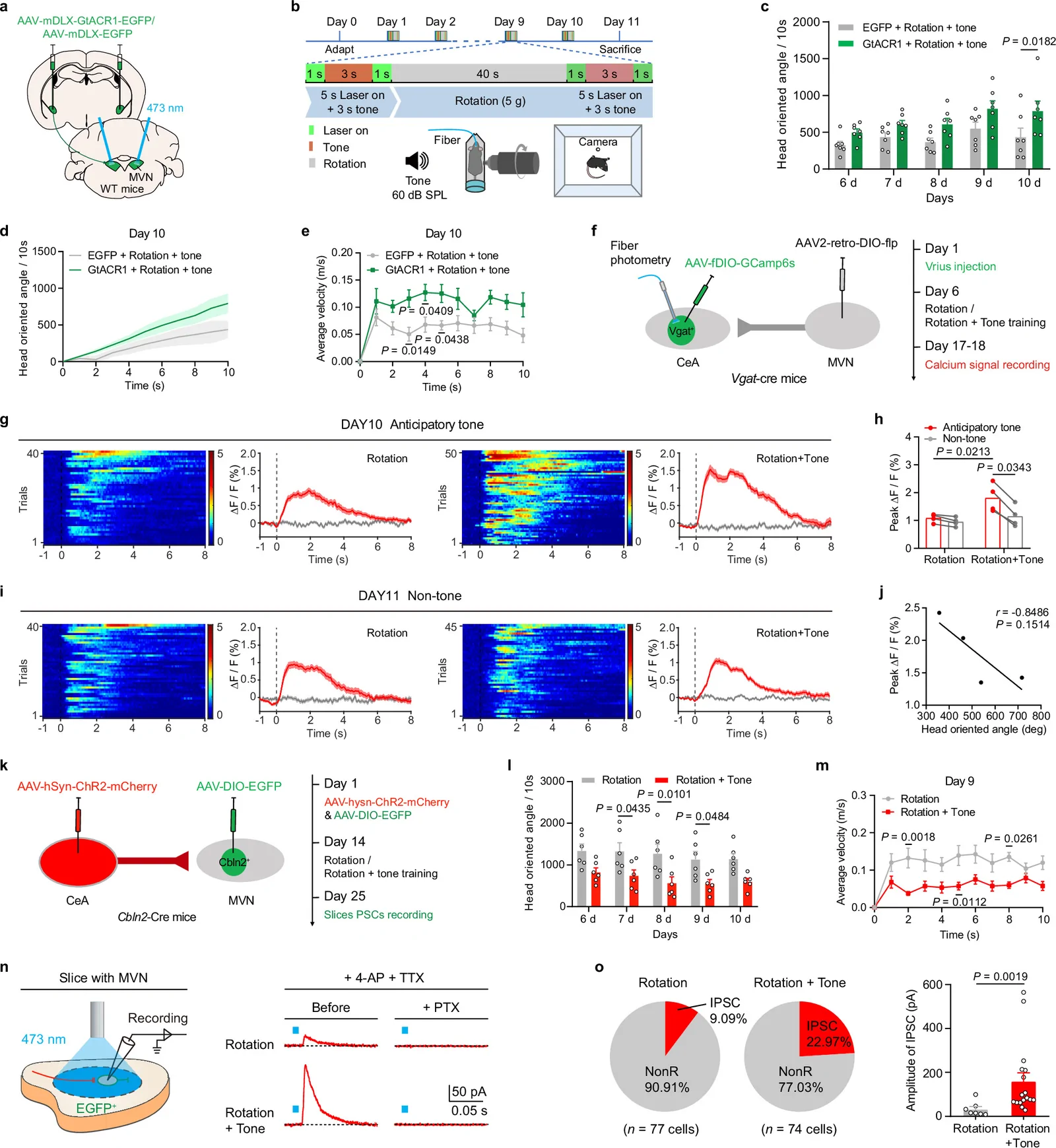
The CeA-MVN^Cbln2^ inhibitory pathway mediates tone-induced alleviation of MS-like behavior. Schematic of viral injection and optical fiber implantation for inhibiting CeA-MVN pathway (**a**). Tone-paired rotation with CeA-MVN pathway inhibition for 10 days (1,000 ms, 10 mW) (**b**). **c** Head orientation angles during the first 10 s post-rotation on days 6–10 (*n* = 7 per group). Head orientation angles (**d**) and average velocity (**e**) during the first 10 s post-rotation on day 10 (*n* = 7 per group). **f** Schematic diagram of projection-specific GCaMP6s recording from Vgat^+^ CeA-MVN neurons. Rotation-aligned GCaMP6s activity on day 10 with tone (**g**), peak responses with or without tone (**h**), and day 11 without tone (**i**) (*n* = 4 per group). Trial heatmaps and group means are shown. Red and gray traces indicate GCaMP6s and 405-nm signals, respectively; thick traces and shaded regions denote mean ± s.e.m. Dashed lines mark onset. Blue shading indicates rotation. **j** Association between peak GCaMP6s responses and head-orientation angles in Rotation+Tone group on day 10. (*n* = 4 mice; two-sided Pearson correlation, 95% CI −0.9968–0.6099) **k** Labeling strategy for Cbln2^+^ MVN neurons and MVN-projecting CeA neurons. Head orientation angles on days 6–10 (**l**) and average velocity on day 9 (**m**) with or without tone training (*n* = 6 per group). **n** Light-evoked IPSCs in EGFP^+^ Cbln2^+^ MVN neurons during stimulation of ChR2-expressing CeA terminals; responses were abolished by PTX. **o** Proportions and amplitudes of light-evoked IPSCs in Rotation (77 cells from 5 mice) and Rotation + Tone mice (74 cells from 5 mice).Data are mean ± s.e.m.; error bars and shading indicate s.e.m. Data in (**c**), (**e**), (**h**), (**l**), and (**m**) were analyzed using two-way ANOVA followed by two-sided Sidak’s multiple-comparisons test. Data in (**o**) were analyzed using a two-sided Mann-Whitney *U* test. ANOVA statistics are provided in Supplementary Table 4, and adjusted and exact *P* values are shown in the figure. Source data are provided as a Source Data file.

To test whether the inhibitory CeA-MVN pathway is necessary and sufficient for tone-induced improvement of MS-like behavior, we performed two complementary experiments. First, we optogenetically inhibited this pathway by injecting AAV-mDLX-GtACR1-EGFP or AAV-mDLX-EGFP into the CeA and implanting optical fibers above the axon terminals in the MVN of WT mice (Fig. 8a). Three weeks after viral expression, mice underwent 10 consecutive days of repeated rotation paired with anticipatory tone, with pathway inhibition delivered during tone presentation each day (Fig. 8b; and Supplementary Fig. 11a, b). Surprisingly, optogenetic inhibition of this pathway during tone presentation (1000 ms, 10 mW) reversed the ameliorative effect of anticipatory tone, leading to increased cumulative head-orientation angles and average velocity in the GtACR1 group compared to the EGFP control group on day 10 (Fig. 8c–e). We next asked whether activation of the same pathway was sufficient to mimic the behavioral benefit of tone training. To do so, we optogenetically activated this pathway during repeated rotation sessions for 10 consecutive days (Supplementary Fig. 11c, d). Notably, optogenetic activation of this pathway (10 ms, 10 mW) mitigated repeated rotation-induced MS-like behavior, as evidenced by reduced cumulative head-orientation angles and average velocity in the ChR2 group compared to the mCherry control group on days 8 and 10 (Supplementary Fig. 11e–g). These findings support a necessary and sufficient role for a predominantly inhibitory CeA-MVN pathway in anticipatory tone-induced alleviation of repeated rotation-induced MS-like behavior.

We next performed calcium imaging to monitor the activity of the inhibitory CeA-MVN pathway in response to anticipatory tones. AAV2-retro-DIO-flp was injected into the MVN, and AAV-fDIO-GCaMP6s was injected into the CeA of *Vgat*-IRES-Cre mice, followed by implantation of an optical fiber above the CeA (Fig. 8f). Three weeks after viral expression, mice underwent 10 consecutive days of repeated rotation, either with or without anticipatory tone pairing. On day 10, both the Rotation alone and Rotation+Tone groups received tone presentation before rotation onset. The Rotation+Tone group exhibited higher GCaMP fluorescence in Vgat^+^ CeA-MVN projection neurons compared to the Rotation group (Fig. 8g, h). On day 11, when both groups underwent rotation without tone presentation, there was no significant difference in GCaMP signals between the two groups (Fig. 8h, i). However, within the Rotation+Tone group, GCaMP fluorescence was lower on day 11 (Non-tone) than on day 10 (tone present) (Fig. 8h). The activity of these neurons was negatively correlated with the severity of MS-like behavior in the Rotation+Tone group on day10 (Fig. 8j). In addition, tone exposure alone (in the absence of rotation) also increased the activity of Vgat^+^ CeA-MVN projection neurons compared to baseline (Supplementary Fig. 11h), confirming that auditory cues can directly engage this pathway and establishing a functional link between auditory input and MVN modulation via the CeA. These results indicate that anticipatory tone reliably enhances the activation of the Vgat^+^ CeA-MVN pathway during rotation, thereby contributing to the alleviation of MS-like behavior.

We then asked whether Cbln2^+^ MVN neurons are a downstream target through which this tone-responsive CeA input exerts its effect. To explore this potential mechanism, we bilaterally injected AAV-hSyn-ChR2-mCherry into the CeA and AAV-EF1a-DIO-EGFP into the MVN of *Cbln2*-IRES-Cre mice (Fig. 8k). First, mice underwent 10 days of repeated rotation with or without tone pairing, starting three weeks after viral expression. Consistent with our earlier results, the Rotation+Tone group exhibited reduced cumulative head-orientation angles and average velocity compared with the Rotation group on days 7-9 (Fig. 8l, m and Supplementary Fig. 12a, b). Second, to investigate the synaptic mechanisms underlying the tone-induced behavioral improvement, we performed postsynaptic current (PSC) recordings in acute brain slices from both groups. Light pulses (473 nm, 2 ms, 20 mW) delivered to ChR2-mCherry^+^ axon terminals from CeA neurons evoked obvious PSCs in Cbln2^+^ MVN neurons (Fig. 8n and Supplementary Fig. 12c). We then compared the proportion and amplitude of PSCs between the two groups. Notably, the Rotation+tone group exhibited a higher proportion and greater amplitude of inhibitory PSCs (IPSCs) compared to the Rotation group (Fig. 8o), whereas the amplitude of excitatory PSCs (EPSCs) did not differ (Supplementary Fig. 12d). The results indicate that tone training selectively enhances inhibitory synaptic transmission in the CeA-MVN^Cbln2^ pathway, without affecting excitatory inputs. Taken together, these findings highlight that anticipatory tone training mitigates repeated rotation-induced MS-like behavior by potentiating inhibitory signaling within the CeA-MVN^Cbln2^ pathway.

## Discussion

**Fig. 9:**
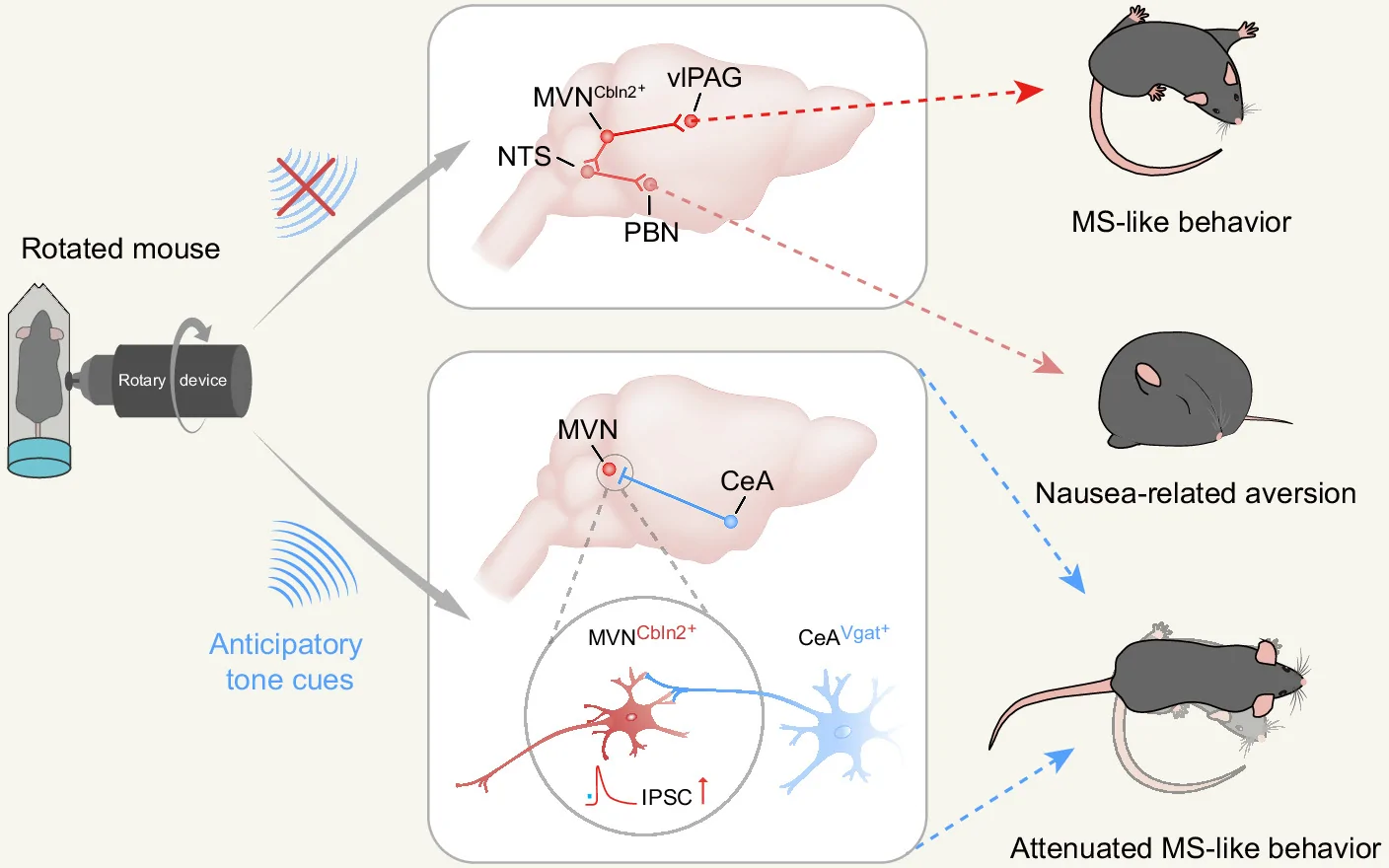
Proposed circuit mechanism by which anticipatory tone cues attenuate rotation-induced MS-like behavior. Rotation activates Cbln2^+^ MVN neurons, which engage the MVN-vlPAG and MVN-NTS-PBN pathways to promote MS-like motor responses and nausea-related aversion, respectively. Anticipatory tone cues recruit Vgat^+^ CeA neurons projecting to the MVN, enhancing inhibitory input onto Cbln2^+^ MVN neurons and thereby suppressing their activity and attenuating rotation-induced MS-like behavior. Created with BioRender.com.

The neural circuitry underlying MS remains inadequately understood, and a clearer mechanistic understanding will be important for the development of more effective prophylactic strategies. Existing countermeasures for MS are limited by undesirable side effects, inconsistent outcomes, and cumbersome implementation. In contrast, tone cues may represent an alternative non-pharmacological approach for modulating MS-related responses^26,27^. In this study, we established a mouse paradigm for rotation-induced MS-like behaviors and identified Cbln2^+^ neurons in the MVN as a major neuronal population contributing to this response. We further found that distinct downstream projections of these neurons mediate the rotation-related motor and nausea-/aversion-related components of MS-like behavior, respectively. In addition, we identified a CeA-MVN^Cbln2^ inhibitory circuit that is recruited by anticipatory tone cues and contributes to the attenuation of repeated rotation-induced MS-like behavior. Together, these findings provide a circuit-level framework for understanding how vestibular and auditory cue-related signals interact to modulate MS-like behavior. We summarized the main findings and showed the neural mechanism of this study with a cartoon (Fig. 9).

### Experimental paradigm for assessing MS-like behavior

One of the most classic behavioral paradigms for eliciting MS (including the visually induced MS and space MS) utilizes gravitational or centripetal acceleration^40,63,64^. In the present study, we used a custom-built vertical rotation paradigm in which mice were physically restrained and visually shielded, thereby minimizing visual input and better controlling proprioceptive confounds. Although rodents lack a vomiting reflex and cannot acquire nausea-related indicators^65–67^, they can actually exhibit certain behaviors and autonomic responses associated with MS through motion stimulation^35,37,40^. High-g rotational paradigms have been widely used in rodent MS research to reliably evoke MS-like responses^9,68,69^. Under our conditions, 5 g ventral vertical rotation generates a robust sensory conflict by maximally engaging the gravitoinertial and otolith-related signals during rotation, thereby eliciting pronounced MS-like responses. We therefore believe that our paradigm is more appropriately described as a model of strong vestibular stimulation-induced MS-like responses, rather than a complete recapitulation of the milder sensory-mismatch conditions that underlie everyday MS. Consistent with this interpretation, a single 40 s session of 5 g ventral vertical rotation was sufficient to induce pronounced tumbling, hypolocomotion, hypothermia, and anorexia, indicating that this paradigm reliably evokes acute MS-like behavioral and autonomic abnormalities.

To figure out the neural mechanism underlying cue-dependent adaptation and mitigation of MS-like behavior, we further developed a repeated rotation paradigm consisting of 10 consecutive days of stimulation. In contrast to the single rotation paradigm, which assesses prolonged post-stimulation effects, the repeated rotation paradigm was designed to reveal a transient but reproducible circling phenotype that emerges immediately after rotation and lasts for approximately 10–20 s. To sensitively detect and compare group differences in this brief but behaviorally relevant window, we specifically analyzed the first 10 s post-rotation. During this period, the increase in average velocity reflected the active motor expression of circling, rather than the reduced spontaneous locomotion observed after acute rotation. Thus, the opposite changes in velocity observed in the single- and repeated-rotation paradigms may reflect different behavioral states measured over different time windows. Moreover, repeated rotation produced sustained reductions in appetite and body temperature, indicating that this paradigm retained classical autonomic MS-like features while showing a distinct motor phenotype.

### Anticipatory tone affects repeated rotation-induced MS-like behavior

Previous studies suggest that anticipatory audio cues^23–25^ and exposure to a pure tone daily at tolerable SPL can mitigate MS^26,27^, possibly through peripheral auditory modulation of vestibular function. Our study extends these insights by focusing on the central neural mechanisms—specifically, how auditory cues influence MS by modulating vestibular processing. We propose that anticipatory tone may exert stronger effects at this level. Thus, the effect of anticipatory tone on repeated rotation-induced MS-like behavior was investigated in this study. Our findings show that anticipatory tone mitigates MS-like behaviors, as reflected by decreased head-orientation angles and average velocity, and also improved appetite. We presented the 3-s tone both before the onset and before the termination of rotation because both phases involve abrupt changes in motion dynamics. By presenting the auditory cue shortly before each change in motion state, the mice could potentially use the tone as a predictive signal for salient vestibular events during the rotation cycle. This design helps the mice actively adjust their behavioral responses to motion onset and offset and supports the formation of stable cue–motion associations. At the same time, the use of a fixed 3 s lead time represents a limitation of the current study. Future work will be needed to determine whether shorter or longer cue durations produce different degrees of behavioral attenuation and whether an optimal anticipatory interval exists. Critically, while prior work highlights that naïve sound can reduce MS, likely mediated via the peripheral auditory system affecting vestibular signaling^26,27^, our results point to anticipatory tone cues alleviating MS through a distinct central mechanism involving the MVN. Both approaches may provide complementary insights into the non-pharmacological modulation of MS-related responses. Further research is required to identify the specific targets and mechanisms of anticipatory tone cues’ action for broader preventive applications.

### Cbln2^+^ MVN neurons are required for rotation-induced MS-like behavior

Elucidating the neural circuit that initiates MS-like behavior is essential for understanding the underlying mechanism of MS and for explaining how anticipatory tone cues alleviate these responses. Several lines of evidence have confirmed the functional roles of vestibular neurons in MS induced by various paradigms^9,35,70^, but the exact cellular components and their specific functional contributions have remained incompletely defined. To address this, we identified Cbln2^+^ neurons as a major neuronal population contributing to rotation-induced MS-like behavior, guided by MVN single-cell expression profiling^9,48^ and a candidate-based functional screen. Our findings indicate that activation of Cbln2^+^ MVN neurons recapitulates major features of the rotation-induced phenotype, whereas ablation of this population substantially suppressed these responses. Chemogenetic activation of Cbln2^+^ MVN neurons produced intermittent, stereotyped circling episodes during prolonged activation, while also causing an overall reduction in locomotor activity and body temperature. We interpret this pattern as reflecting the coexistence of a transient rotation-related motor output and a more sustained MS-like depressive state, resembling the hypolocomotion and hypothermia observed after acute rotation. In this context, the transient increase in movement associated with circling is outweighed by the broader suppression of spontaneous locomotion when average velocity is measured across the longer recording window. Our loss-of-function data further support an important role for Cbln2^+^ MVN neurons in the expression of rotation-induced MS-like behavior. At the same time, one limitation of the current study is that ablation efficiency was estimated using NeuN immunostaining^71–74^ to quantify overall neuronal loss within the MVN, rather than by directly counting the surviving Cbln2^+^ neurons themselves. Direct cell-type-specific quantification would provide stronger evidence in future studies. In addition, fiber photometry showed that Cbln2^+^ MVN neurons responded to rotational stimulation in an intensity-dependent manner, with neuronal activity scaling with stimulus strength. These findings support the view that Cbln2^+^ MVN neurons are a major vestibular cell population engaged by strong rotational stimulation and required for a substantial component of the resulting MS-like behavioral response.

### Role of Cbln2- and Cck-expressing neurons in the MVN on MS-like behavior

Since Machuca-Márquez and colleagues previously demonstrated that Cck^+^ MVN neurons drive rotation-induced autonomic responses^9^, we compared the effects of Cbln2^+^ and Cck^+^ MVN neurons on rotation-induced MS-like behavior using chemogenetic activation together with fiber photometry. Activation of either population triggered MS-like autonomic responses, including hypolocomotion and hypothermia. However, the motor phenotypes differed. Activation of Cbln2^+^ neurons induced pronounced circling behavior (Supplementary Movie 3), consistent with a previous report showing that activation of Vglut2^+^ MVN neurons leads to counterclockwise rotation along the edge of the cage and spinning behavior^37^. In contrast, activation of Cck^+^ neurons predominantly induced body tottering (Supplementary Movie 4), resembling the phenotype observed following activation of Vgat^+^ MVN neurons, which caused freezing behavior and tottering with the body tilted to the right side^37^. Our anatomical data further support the idea that *Cbln2* and *Cck* define largely separate excitatory neuronal subpopulations within the MVN (Supplementary Fig. 4e). Besides, fiber photometry showed that Cbln2^+^ neurons exhibited larger calcium responses than Cck^+^ neurons during rotation-induced MS-like behavior, suggesting that Cbln2^+^ neurons may be more strongly engaged in processing this behavior. Also noteworthy is that Cbln2^+^ and Cck^+^ neurons showed partly distinct downstream projection patterns (Supplementary Fig. 7b), suggesting that they may mediate different aspects of MS-like behavior through their specialized output pathways. At present, however, these differences should be interpreted cautiously. Further work will be needed to define the precise functional relationship between these two vestibular cell populations and to determine whether they operate independently or in parallel during motion-induced responses.

### Brain circuits modulating rotation-induced MS-like behavior

Our data indicate that Cbln2^+^ MVN neurons project to multiple downstream targets, including the vlPAG, NTS, IPN, and CPu. Among these projections, the Cbln2^+^ MVN-vlPAG pathway appears to be most closely associated with the rotation-induced MS-like behavioral response, whereas activation of the MVN-NTS, -IPN, or -CPu pathways did not produce comparably overt behavioral effects in our assays. This raises the important question of whether the observed circling phenotype reflects a direct motor command or, instead, a motor manifestation of vestibular imbalance associated with MS-like states. Prior studies have shown that unilateral activation of vestibular glutamatergic neurons is sufficient to induce immediate postural imbalance, directional turning/spinning, and impaired motor coordination^37,38^. In parallel, activation of vestibular CCK^+^ neurons can induce several MS-like responses^9^, indicating that vestibular neuronal activation can simultaneously engage malaise-related and motor/postural outputs. We therefore favor a cautious interpretation. Unilateral activation of Cbln2^+^ MVN neurons or the MVN-vlPAG pathway likely produces a vestibular-imbalance-associated MS-related behavior. The resulting circling may reflect a reflexive or compensatory response to dizziness, imbalance, or altered body-orientation estimates^75^, rather than control of an isolated motor action^76,77^. At the same time, a more direct contribution to motor output remains possible, and our current data do not distinguish between these possibilities. Moreover, selective ablation of the Cbln2^+^ MVN-vlPAG circuit largely alleviated rotation-related behavioral phenotype, supporting an important role for this circuit in the motor expression of rotation-induced MS-like behavior. By contrast, activation of the Cbln2^+^ MVN-NTS circuit, but not the MVN-vlPAG circuit, promoted an MS-related nausea/aversion response via CFA analysis. These findings suggest that the Cbln2^+^ MVN-NTS pathway represents an important nausea-related branch of the vestibular circuit, but not necessarily the sole pathway mediating MS-like nausea-related responses. Our results further suggest that this pathway may engage a downstream PBN-dependent mechanism to support the aversion-related component of the response.

### CeA-MVN^Cbln2^ inhibitory circuit in anticipatory tone-induced mitigation of MS-like behavior

We previously found that an anticipatory tone mitigates repeated rotation-induced MS-like behavior (Fig. 2). Building on this observation, we further showed that anticipatory tone cues suppress the activity of Cbln2^+^ MVN neurons, and that this suppression correlates with MS-like behavioral improvement. To identify a potential source of this modulation, we used rabies-mediated retrograde tracing and found that Cbln2^+^ MVN neurons receive inputs from several upstream regions, including the CeA (Fig. 7h). The amygdala is particularly sensitive to sound with valence or learned significance and plays a central role in auditory fear conditioning and emotional behavioral responses^78–82^. The CeA serves as a key output station that integrates diverse sensory inputs and regulates automatic or autonomic functions^83,84^. Auditory information can reach the amygdala through both thalamic and cortical pathways^85^, making the CeA a plausible node through which anticipatory auditory cues could influence vestibular processing. Consistent with this idea, we identified a predominantly inhibitory CeA-MVN pathway that contributes to anticipatory tone-induced attenuation of repeated rotation-evoked MS-like behavior, as supported by optogenetic activation and inhibition, together with fiber photometry analyses. At the synaptic level, tone training enhanced inhibitory input from CeA onto Cbln2^+^ MVN neurons, as reflected by the increased proportion and amplitude of light-evoked IPSCs. These findings support the interpretation that anticipatory tone engages a central inhibitory circuit that dampens the activation of Cbln2^+^ MVN neurons during rotation. At present, however, the upstream route by which auditory cues recruit this circuit remains unresolved. Although it is plausible that auditory signals reach the amygdala through known thalamic and cortical pathways, our data do not directly establish the precise sequence of nodes linking tone perception to CeA-MVN inhibition. We therefore interpret the CeA-MVN^Cbln2^ pathway as a candidate circuit mechanism through which anticipatory tone may reduce MS-like behavior, rather than as the only pathway involved. Overall, our findings support the view that learned anticipatory auditory cues mitigate repeated rotation-induced MS-like behavior, at least in part, by strengthening inhibitory CeA input onto Cbln2^+^ MVN neurons. These findings provide a circuit-level basis for understanding how anticipatory cues modulate MS-like behavior.

## Methods

### Animals

Male C57BL/6J and Cre-driver mice (*Mus musculus*) were used in this study. C57BL/6 J mice were obtained from Guangdong GemPharmatech Co., Ltd (Foshan, China). *Cbln2*-IRES-Cre, *Chat*-IRES-Cre, *Crh*-IRES-Cre, *Cck*-IRES-Cre, *Vglut2*-IRES-Cre, and *Vgat*-IRES-Cre mouse lines were obtained from the Jackson Laboratory (JAX Mice and Services). Mice were housed at 23 ± 1 °C and 50 ± 5% humidity on a 12-h light/12-h dark cycle. Standard chow and water were provided *ad libitum* unless otherwise indicated; where specified, mice were fasted overnight for 16–18 hours before testing. Mice used in individual experiments were 8–12 weeks old and were at least 8 weeks old at the time of viral injection. After virus injection, mice were individually housed for 2-3 weeks before subsequent experiments. Only male mice were used to reduce sex-related variability in the present study; therefore, sex was not evaluated as a biological variable. All experimental procedures were approved by the Administrative Panel on Laboratory Animal Care (GZLABAUCP202404A10) in the Guangzhou laboratory (Guangzhou, China).

### Rotation-induced MS-like behavior paradigm

To induce motion sickness (MS)-like behavior, mice were subjected to rotational stimulation using a custom-built device consisting of a speed-adjustable rotator and a modified restraint system made from a well-ventilated, visually shielded 50-mL conical tube. Mice were habituated to the restraint tube for 3 consecutive days (3 min per day) before testing to minimize stress-related confounds. On the test day, each mouse was placed into the tube, which was mounted horizontally onto the rotator arm, such that the estimated position of the head was approximately 5.0 ± 0.1 cm from the center of rotation. Mice remained in the stationary tube for 3 min before stimulation. Rotation was delivered at 300 ± 5 rpm for 40 s, generating a centripetal acceleration of 5.0 ± 0.2 g according to the formula *a* = (2π*n*/60) ² × *r* (where *n* is the rotational speed in rpm and *r* is the radius in meters). Depending on the experiment, mice underwent ventral vertical, dorsal vertical, or horizontal rotation. Ventral vertical rotation at 1.25, 2.5, and 5 g was used for behavioral comparisons, whereas 0.05, 0.5, and 5 g were used for fiber-photometry experiments. Control mice underwent the same handling and restraint procedures without rotation.

### Measuring MS-like behavior

Mice were individually habituated to a white acrylic circular open-field arena (28 cm in diameter) for 15 min before rotational or control treatment. Immediately after stimulation, mice were returned to the arena, and their behavior was recorded for 30 min using an overhead camera (30 frames/s; Point Grey Research, Canada). MS-like behavior during the first 10 s after rotation was assessed using a scoring system, which included (i) tumbling: whole-body rolling around the longitudinal body axis (score 5); (ii) Retropulsion: backward locomotion (score 4); (iii) Circling: circular locomotion in the horizontal plane (score 3); (iv) Bobbing: rapid head tilts to the rear and is assimilated with cephalic nystagmus (score 2); and (v) Head tilt (score 1). A score of 0 was assigned when none of these behaviors were observed. When multiple behaviors occurred within the scoring period, the highest score was assigned. Behavioral scoring was performed by an investigator blinded to the experimental groups. Locomotor activity was quantified using animal behavior analysis software (VisuTrack XR-VT, Xinruan Information Technology, China).

### Food intake and temperature measurement

For the food intake test, mice were individually habituated to the testing cage for 3 consecutive days. Beginning at the onset of the dark phase, mice were food-deprived for 16–18 hours with water available *ad libitum*. Cumulative food intake was measured for 2 hours following rotational or control stimulation. For temperature monitoring, an infrared camera (FOTRIC 280, Thermal Imaging Technology, China) was mounted above the arena and captured thermal images of the mouse every 30 s. Mice were habituated to the arena for 30 min before stimulation, during which baseline body temperature was recorded. After stimulation, mice were returned to the arena, and body temperature was recorded for 1 hour. Thermal images were analyzed using FOTRIC AnalyzIR software.

### Repeated rotation-induced MS-like behavior paradigm

To assess MS-like responses during repeated stimulation, mice were habituated to a white acrylic circular open-field arena (28 cm in diameter) for 15 min and then restrained in the stationary rotation tube for 3 min. Mice subsequently underwent ventral vertical rotation at 5 g for 40 s once daily for 10 consecutive days. Immediately after each rotation session, mice were returned to the open-field arena, and behavior was recorded using an overhead camera (30 frames/s; Point Grey Research, Canada). Mice underwent daily rotation for 10 consecutive days. Control mice underwent the same procedure without rotation. Behavioral scores were assessed during the first 10 s post-rotation on days 1-5. On days 6-10, cumulative head-orientation angles and average velocity during the first 10 s post-rotation were quantified using VisuTrack XR-VT animal behavior analysis software.

### Measuring anticipatory tone effects on repeated rotation-induced MS-like behavior

Broadband white noise (WN) (5-45 kHz) was generated using custom Python scripts and delivered using BatSound 4.0 software through a free-field speaker positioned adjacent to the rotation apparatus. Sound intensity was set to 60 dB sound pressure level (SPL), measured at the position of the rotary device using a sound level meter (Suwei Technology, China).

Mice were assigned to three groups. In the Rotation+Fixed Tone group, a 3-s white noise cue was presented immediately before rotation onset and during the final 3 s before rotation offset. In the Rotation+Random Tone group, which served as an auditory-exposure control for the Fixed-Tone condition, two 3-s white-noise cues were presented at randomly selected times during each 40-s rotation session, excluding the fixed-cue time windows. The Rotation group underwent rotation without auditory cues. All groups underwent the corresponding protocol once daily for 10 consecutive days. Immediately after each rotation session, behavior was recorded in the open-field arena using an overhead camera (30 frames/s; Point Grey Research, Canada). Behavioral scores were assessed during the first 10 s post-rotation on days 1–5, whereas cumulative head-orientation angles and average velocity during the same interval were quantified on days 6–10 using VisuTrack XR-VT software. Food intake and body temperature were measured on days 1 and 10 as described above.

### AAV vectors

AAV capsids and variants used in this study included AAV2/9, AAV2/2, and AAV2-retro. The AAVs used in this study are listed in Supplementary Table S1. Viral particles were purchased from Shanghai Taitool Bioscience Inc. and Brain VTA Inc. Stock titers ranged from 0.8 to 1.5 × 10^13^ viral particles/ml. The final titer used for AAV injection after dilution with PBS was 2 × 10^12^ or 4.8 × 10^12^ viral particles/ml.

### Stereotaxic viral injection

Mice were anesthetized with tribromoethanol (125–250 mg/kg, intraperitoneal injection) and placed in a stereotaxic frame (RWD Life Science, China). Standard stereotaxic procedures were used to expose the brain surface above the MVN, vlPAG, NTS, PBN, or CeA. Injection coordinates were as follows: MVN, bregma −6.00 mm, lateral ±0.60 mm, ventral −4.30 mm; vlPAG, bregma −4.84 mm, lateral ±0.45 mm, ventral −2.65 mm; NTS, bregma −7.32 mm, lateral ±0.25 mm, ventral −3.15 mm; PBN, bregma −5.20 mm, lateral ±1.40 mm, ventral −2.50 mm; CeA, bregma −1.22 mm, lateral ±2.75 mm, ventral −4.60 mm. AAVs were injected using a glass pipette connected to a Nanoliter Injector 201 (World Precision Instruments) at 0.15 μl/min to minimize tissue damage. The pipette was left in place for at least 20 min after viral injection before withdrawal.

### Optic fiber implantation

Thirty minutes after AAV injection, a ceramic ferrule carrying an optic fiber (optogenetics: 200 μm in diameter, N.A. 0.22; fiber photometry: 230 μm in diameter, N.A. 0.37) was implanted with the fiber tip positioned above the MVN (bregma −6.00 mm, lateral ±0.60 mm, ventral −4.10 mm), vlPAG (bregma -4.84 mm, lateral ±0.45 mm, ventral −2.50 mm), NTS (bregma −7.48 mm, lateral −0.25 mm, ventral −4.30 mm), CeA (bregma −1.22 mm, lateral -1.75 mm, ventral −4.40 mm). The ferrule was secured on the skull with dental cement. After implantation, the skin was sutured, and topical antibiotics were applied to the wound. Experiments were conducted at least 2–3 weeks after fiber implantation. Viral expression and fiber placement were verified histologically after completion of the experiments.

### Slice physiology

Brain slices containing the MVN, vlPAG, NTS, or CeA were prepared from adult mice anesthetized with isoflurane before decapitation. Brains were rapidly removed and placed in ice-cold oxygenated (95% O_2_ and 5% CO_2_) cutting solution (CS: 228 mM sucrose, 11 mM glucose, 26 mM NaHCO_3_, 1 mM NaH_2_PO_4_, 2.5 mM KCl, 7 mM MgSO_4_, and 0.5 mM CaCl_2_). Coronal brain slices (250 μm) were cut using a vibratome (VT 1200S, Leica Microsystems, Wetzlar, Germany). The slices were incubated at 28 °C in oxygenated artificial cerebrorotational fluid (ACSF: 119 mM NaCl, 2.5 mM KCl, 1 mM NaH_2_PO_4_, 1.3 mM MgSO_4_, 26 mM NaHCO_3_, 10 mM glucose, and 2.5 mM CaCl_2_) for 30 min, and were then kept at room temperature under the same conditions for 1 hour before transfer to the recording chamber. The ACSF was perfused at 1 ml/min. The MVN, vlPAG, NTS, or CeA slices were visualized with a ×40 Olympus water immersion lens, differential interference contrast (DIC) optics (Olympus Inc., Japan), and a CCD camera (QImaging Rolera-XR, BC, Canada). Patch pipettes were pulled from borosilicate glass capillary tubes (Cat #64-0793, Warner Instruments, Hamden, CT, USA) using a PC-10 pipette puller (Narishige Inc., Tokyo, Japan). Patch electrodes had resistances of 3.0-3.5 MΩ.

For current-clamp recordings, pipettes were filled with solution (in mM: 135 K-methanesulfonate, 10 HEPES, 1 EGTA, 1 Na-GTP, 4 Mg-ATP, pH 7.4). For voltage-clamp recordings from ChR2-mCherry-negative vlPAG or NTS neurons, pipettes were filled with solution (in mM: 135 CsCl_2_, 10 HEPES, 1 EGTA, 1 Na-GTP, 4 Mg-ATP, pH 7.4). For recording from ChR2-mCherry-negative and EGFP^+^ MVN neurons, the membrane potential was held at −70 and 0 mV, respectively. Pipettes were filled with solution (in mM: 130 CsMeSO3, 10 NaCl, 10 EGTA, 4 MgATP, 0.3 Na3GTP, 10 HEPES, 290 mOsm, adjusted to 7.4 with CsOH). EPSCs and IPSCs were recorded at holding potentials of −70 and 0 mV, respectively. Signals were acquired using a MultiClamp 700B and Clampex 10 data acquisition software (Molecular Devices). After establishing the whole-cell configuration and equilibrating the intracellular pipette solution with the cytoplasm, series resistance was compensated to 10-15 MΩ. Recordings with series resistances > 15 MΩ were rejected. An optical fiber (200 μm in diameter) was positioned above the brain slices, with laser intensity adjusted to 20 mW. Pulse onset, duration, and frequency of light stimulation were controlled by a programmable pulse generator attached to the laser system.

Light-evoked action potentials from ChR2-mCherry^+^ neurons were triggered by a light-pulse train (473 nm, 1 ms, 10 Hz, 20 mW) synchronized with Clampex 10 data acquisition software (Molecular Devices). Light-evoked synaptic currents from ChR2-mCherry-negative vlPAG, NTS, or EGFP^+^ MVN neurons were triggered by single light pulses (1 ms) in the presence of 4-AP (20 μM) and TTX (1 μM). In separate experiments, glutamate receptor antagonists (D-AP5, 50 μM; CNQX, 20 μM) or the GABA receptor antagonists (PTX, 50 μM) were perfused to determine whether the evoked responses were abolished and thereby identify the neurotransmitter phenotype of the recorded neurons.

### Immunohistochemical procedures

The immunohistochemical staining procedure was performed according to a previously published protocol. Mice were anesthetized with tribromoethanol (125–250 mg/kg) and sequentially perfused with saline and PBS containing 4% paraformaldehyde. Brains were removed and incubated in PBS containing 30% sucrose until they sank to the bottom. Cryostat sections (40 μm) containing the MVN, vlPAG, NTS, PBN, or CeA were collected, incubated for 2 h with blocking solution (PBS containing 10% goat serum and 0.7% Triton X-100), and then treated with primary antibodies diluted with blocking solution for overnight at 4 °C. Primary antibodies used for immunohistochemistry are provided in Supplementary Table S1. Primary antibodies were washed three times with washing buffer (PBS containing 0.7% Triton X-100) before incubation with secondary antibodies (tagged with Cy2 or Cy3; dilution 1:500) for 2 h at room temperature. Sections were then washed three times with washing buffer, stained with DAPI, and then washed with PBS. Sections were transferred onto Super Frost slides and mounted under glass coverslips with mounting media. Sections were imaged using a Leica Aperio Versa 8 microscope, Olympus VS 200 (10x objective lens), or a Zeiss 980 laser scanning confocal microscope (60 oil-immersion objective lens). Samples were excited by 488- or 546-nm lasers in sequential acquisition mode to avoid signal leakage. Confocal images were analyzed using ImageJ software.

### RNA in situ hybridization (FISH)

RNA in situ hybridization was performed using the RNAscope Multiplex Fluorescent Reagent Kit v2 (ACD, 323100) according to the manufacturer’s protocol. Mice were perfused with 0.9% Sodium chloride prepared in (Guangzhou Chemical Reagent Factory) RNase-free ddH_2_O, followed by PBS containing 4% PFA (Aladdin). Brains were post-fixed overnight in ddH_2_O-treated PBS-PFA solution and then placed in PBS-treated 30% sucrose solution at 4 °C until they sank to the bottom. Brain sections (12-μm thick) were prepared using a cryostat (Leica, CM3050S) and collected on the glass slides. Fluorescence in situ hybridization (FISH) was performed as described as follows. Briefly, brain sections were rinsed with ddH_2_O-treated PBS for 5 min, and then placed in a dryer at 60 °C for 30 min. Brain sections were then treated with 4% PFA (PBS-PFA) solution for 15 min. The slides were sequentially immersed in 50% ethanol, 70% ethanol, and anhydrous ethanol for tissue dehydration. Add 2-4 drops of RNA Scope hydrogen peroxide to the dry glass slides to cover the entire brain sections, and incubate at room temperature for 10 min. After washing, the slides were slowly placed into the boiling Co-Detection Target Retrieval solution (ACD, 323166) for 5 min and then were washed with PBST. The brain sections were incubated with primary antibodies (tagged with Cy2; dilution 1:600) against EGFP or mCherry, diluted with Co-Detection Antibody Diluent (ACD, 323160) in a black Immunohistochemical Wet Box at 4 °C for 24 hours. After washing with fresh PBST, brain sections were then treated with 4% PFA (PBS-PFA) solution at room temperature for 30 min. The slides were washed again with fresh PBST. The brain sections were covered with protease plus. After 30 minutes of incubation in the Immunohistochemical Wet Box at 40 °C. After washing, the brain sections were hybridized with the RNAscope Probe-Mm-Cbln2 (ACD, 428551) at 40 °C for 2 hours in the Immunohistochemical Wet Box. After washing with 1 × Wash Buffer (ACD, 320058), remove excess liquid, add AMP1 (ACD) to the slide, and place it in the Immunohistochemical Wet Box to incubate in the dryer for 30 min. The above steps were repeated sequentially with the addition of AMP2 and AMP3. After washing the slides and removing excess liquid, the brain sections were covered with HRP-probes for *Cbln2* and then placed in the wet box in the dryer at 40 °C for 15 min. After washing, the brain sections were incubated with the probe’s secondary antibodies diluted with TSA buffer (dilution 1:15) at 40 °C for 30 min. Washing with 1 × Wash Buffer was repeated, and the brain sections were incubated with HRP blocker at 40 °C for 15 min. To visualize EGFP or mCherry signals, the brain sections were incubated with Alexa Fluor® 488 or 555-conjugated goat anti-rabbit secondary antibodies (1:500, A11034, Invitrogen) at room temperature for 30 min. Sections were imaged using an Olympus VS 200 microscope (10x objective lens) or a Zeiss 980 laser scanning confocal microscope (60 oil-immersion objective lens).

### Fiber photometry

AAV-DIO-GCaMP6s or AAV-DIO-EGFP was stereotaxically injected into the MVN of *Cbln2*-IRES-Cre or *Cck*-IRES-Cre mice. AAV-retro-DIO-flp was stereotaxically injected into the vlPAG, and AAV-fDIO-GCaMP6s or AAV-fDIO-EGFP was stereotaxically injected into the MVN of *Cbln2*-IRES-Cre mice. AAV-retro-DIO-flp was stereotaxically injected into the MVN, and AAV-fDIO-GCaMP6s or AAV-fDIO-EGFP was stereotaxically injected into the CeA of *Vgat*-IRES-Cre mice. After the virus injection, an optical fiber (diameter, 230 μm, NA = 0.37; Fiblaser Technology, China) was inserted above the MVN or CeA.

GCaMP signals were recorded at least 2 weeks after surgery using a fiber-photometry system (Thinker Tech Nanjing Biotech, China). Purple LED light (405/10 nm, model 65133, Edmund Optics) and blue LED light (470/10 nm, model 65144, Edmund Optics) were bandpass filtered, reflected by a dichroic mirror (model 67-069,495 nm long pass, Edmund Optics) and a dichroic mirror (model 87-282, multi-band filter, Edmund Optics), and then focused using a 20× objective lens (Olympus). Excitation light at 470 nm was used to record calcium-dependent fluorescence, and the 405-nm channel served as an isosbestic reference for correction of movement-related artefacts. A flashing light-emitting diode triggered by a 1-s square-wave pulse was simultaneously recorded to synchronize the video and GCaMP signals.

During recording, mice were restrained in a 50-mL tube mounted on the rotary device and subjected to rotation at 5 g, 0.5 g, or 0.05 g for 2 or 4 cycles. For anticipatory tones experiments, mice received a 3 s tone before rotation at 0.5 g for 4 circles. GCaMP signals were aligned to rotation or tone onset. For each trial, *F*_*0*_ was defined as the mean fluorescence signal during the baseline period from −2 to 0 s, and Δ*F*/*F*_*0*_ was calculated as (*V*_signal_−*F*_*0*_)/(*F*_*0*_−*V*_offset_), where *V*_offset_ represents the system offset. Trial-level traces were then averaged within each mouse, and the mouse was used as the biological experimental unit. Viral expression and optical-fiber placement were verified histologically after recording.

### Cell-type-specific anterograde tracing

For anterograde labeling of Cbln2^+^ or Cck^+^ MVN neurons, AAV-DIO-H2B-EGFP-Synaptophysin-mRuby or AAV-DIO-hM3D-mCherry was injected into the MVN of the corresponding Cre-driver mice. Three weeks later, mice were perfused with saline followed by 4% PFA in PBS. Brains were post-fixed in 4% PFA for 8 hours and then incubated in PBS containing 30% sucrose until they sank to the bottom. Coronal brain sections at 40 μm thickness were prepared using a cryostat (Leica CM1900). Sections were stained with DAPI and, where applicable, an antibody recognizing mCherry/mRuby. The coronal brain sections were imaged with an Aperio Versa 8 microscope (10x objective lens).

### Cell-type-specific retrograde tracing

To label NTS- and vlPAG-projecting Cbln2^+^ MVN neurons, AAV2/2retro-DIO-H2B-mCherry and AAV2/2Retro-DIO-H2B-EGFP into the vlPAG and NTS, respectively, of *Cbln2*-IRES-Cre mice. Three weeks later, brains were fixed, cryoprotected, and sectioned coronally at 40 μm as described above. MVN coronal sections were counterstained with DAPI and imaged using an Aperio Versa 8 (10x objective lens) microscope and a Zeiss 980 (60 oil-immersion objective lens) microscope.

### Cell-type-specific RV tracing

Monosynaptic inputs to Cbln2^+^ MVN neurons were mapped using an EnvA-pseudotyped, glycoprotein-deleted rabies-virus tracing system. A 1:1 mixture of AAV2/9-EF1a-DIO-mCherry-2A-TVA and AAV2/9-EF1a-DIO-RVG (total volume, 200 nL; 5 × 10^12^ viral particles/ml each) was injected unilaterally into the MVN of *Cbln2*-IRES-Cre mice. Two weeks later, RV-EnvA-ΔG -EGFP (200 nL; 5 × 10^8^ viral particles/ml) was injected into the same site under a biosafety level-2 lab facility. Starter neurons were characterized by the coexpression of mCherry and EGFP within the MVN. One week after RV injection, mice were perfused and brains were processed into 40 μm coronal sections. Sections were stained with DAPI or the indicated antibodies and imaged using an Olympus VS 200 microscope (10x objective lens) and analyzed with ImageJ software. Brain-region boundaries were defined using the Allen Mouse Brain Atlas. RV-labeled input neurons were counted in the predefined brain regions, and the proportion of input cells in each region was calculated relative to the total number of RV-labeled input cells quantified in each mouse.

### Cell-counting strategies

The cell-counting strategies are summarized in Table S3. For MVN analyses, three coronal sections spanning bregma −5.80 to −6.24 mm and spaced 200 μm apart were analysed from each mouse. Sections were 40 μm thick for immunohistochemistry and 12 μm thick for fluorescence in situ hybridization. For whole-brain analyses, one 40 μm coronal section was sampled every 200 μm. Regional axonal-projection fluorescence and RV-EGFP^+^ input cells were quantified using ImageJ. Analysed regions included the CeA (central amygdaloid nucleus), PnO (pontine reticular nucleus), and selected cortical regions, including M1, M2, S1, S2, DI, and GI). Measurements were first averaged or summed within each mouse, and the mouse was treated as the biological experimental unit.

### Preparation of behavioral tests

After viral injection and fiber implantation, mice were individually housed for 3 weeks before behavioral testing. Mice were handled daily by the experimenters for at least 3 days before testing. On the test day, mice were transferred to the behavioral room and habituated to room conditions for 3 hours before the experiments started. The apparatus was cleaned with 20% ethanol to eliminate odor cues. All behavioral tests were performed during the same circadian period (1 PM–7 PM).

### Chemogenetically evoked MS-like behavior

Mice were habituated to a white acrylic circular open-field arena (28 cm in diameter) for 15 minutes. Mice then received an intraperitoneal injection of CNO (1 mg/kg) or saline (as a control) and returned to the arena. Saline and CNO sessions were performed in a counterbalanced order and separated by a washout interval of 3 days. Behavior was recorded with a visible-light (30 frames/s; Point Grey Research, Canada) and an infrared camera. Chemogenetically evoked MS-like behavior was identified by circling behavior and quantified by measuring the number of circling bouts, behavioral score, average velocity, and body temperature during the 30-60 min period after CNO injection. The ability of CNO to evoke action-potential firing in MVN neurons was validated by slice electrophysiology.

### Optogenetically evoked MS-like behavior

AAV-DIO-hChR2-mCherry or control AAV-DIO-mCherry was injected into the MVN of corresponding Cre mouse lines, and an optical fiber was implanted above the MVN, vlPAG, NTS, IPN or CPu. Two or three weeks later, mice were allowed to explore a square open-field arena (20 cm × 20 cm, square open field) for 15 minutes. Light stimulation was applied to activate MVN neurons or Cbln2^+^ MVN neuron axon terminals in the indicated target regions. Behavior was recorded using two orthogonally positioned cameras (30 frames/s; Point Grey Research, Canada). Behavioral scores, cumulative head-orientation angles and average velocity were quantified during the 5 s stimulation period. Laser-power responses were tested at 5, 10, and 20 mW while maintaining stimulation at 20 Hz, whereas frequency responses were tested at 5, 10, and 20 Hz while maintaining the power at 20 mW. Pulse width and stimulation duration were 10 ms and 5 s, respectively.

### Behavioral assessment after neuronal ablation

To ablate Cbln2^+^ MVN neurons, AAV-Flex-taCasp3 or control AAV-DIO-EGFP was bilaterally injected into the MVN of *Cbln2*-IRES-Cre mice. To ablate vlPAG-projecting Cbln2^+^ MVN neurons, AAV-retro-DIO-flp was bilaterally injected into the vlPAG, followed by bilateral injection of AAV-fDIO-taCasp3 or control AAV-fDIO-EGFP into the MVN. Two or three weeks after the final viral injection, mice were habituated to a circular open-field arena (28 cm in diameter) for 15 min and restrained in the stationary rotation tube for 3 min. Mice were then subjected to ventral vertical rotation at 5 g for 40 s once daily for 10 consecutive days and immediately returned to the arena. Behavior was recorded using an overhead camera (30 frames/s; Point Grey Research, Canada). Behavioral scores during the first 10 s post-rotation were assessed on days 1–5. Cumulative head-orientation angles and average velocity during the same interval were quantified on days 6–10. Food intake was measured on days 1 and 10 as described above. Viral expression, injection sites and ablation efficiency were verified histologically after completion of the experiments.

### Conditioned flavor avoidance behavior

For pathway activation experiments, AAV-hEF1a-DIO-hChR2-mCherry or control AAV-hEF1a-DIO-mCherry was injected into the MVN of *Cbln2*-IRES-Cre mice, and an optical fiber was implanted above the NTS or vlPAG. For experiments combining activation of MVN terminals in the NTS with inhibition of excitatory PBN neurons, AAV-hEF1a-DIO-hChR2-EGFP was injected into the MVN and AAV-mCaMKIIa-hM4D-mCherry or control AAV-CaMKIIa-mCherry was injected into the PBN of *Cbln2*-IRES-Cre mice. Two weeks after AAV injection, mice were housed with two drinking bottles containing unflavored water for 3 days. On days 4-6, mice were familiarized with grape- and orange-flavored water, and intake was measured daily. After 12 hours of water deprivation on day 7, mice underwent conditioning on day 8. One bottle contained grape-flavored water and the other orange-flavored water. Licks from the bottle containing grape- or orange-flavored solution triggered optical stimulation through a lick-detection circuit interfaced with a 1401 digitizer, whereas licks from the other bottle were not paired with stimulation. Intake from each bottle was measured over 2 hours. On day 9, both bottles contained unflavored water. On day 10, grape- and orange-flavored water were presented simultaneously for 2 hours, with bottle positions randomized or counterbalanced across mice. Intake from each bottle was used to assess conditioned flavor avoidance. In experiments involving PBN inhibition, CNO (1 mg/kg, intraperitoneally) was administered 30 min before testing.

### Optogenetic manipulation of inhibitory CeA inputs to Cbln2^+^ MVN neurons during repeated rotation

For pathway activation, AAV-mDLX-hChR2-mCherry or AAV-mDLX-mCherry was bilaterally injected into the CeA of WT mice, and an optical fiber was implanted above the MVN to stimulate CeA axon terminals. For pathway inhibition, AAV-mDLX-GtACR1-EGFP or AAV-mDLX-EGFP was bilaterally injected into the CeA, with fibers implanted above the MVN. Three weeks after viral injection, mice were habituated to a circular open-field arena (28 cm in diameter) for 15 min and restrained in the stationary rotation tube for 3 min. Mice than underwent daily rotation for 10 consecutive days. For pathway activation, light stimulation (20 Hz, 10 ms, 10 mW) was delivered immediately before rotation onset and during the final 3 s before rotation offset. For pathway inhibition, light stimulation (1 Hz, 1000 ms, 10 mW) was delivered together with a 3-s tone cue immediately before rotation onset and rotation offset. Behavior was recorded using an overhead camera (30 frames/s; Point Grey Research, Canada). Behavioral scores during the first 10 s post-rotation were assessed on days 1-5, whereas cumulative head-orientation angles and average velocity during the same interval were quantified on days 6–10 using VisuTrack XR-VT software. Viral expression and optical-fiber placement were verified histologically.

### Statistics and reproducibility

No statistical methods were used to predetermine sample sizes, which were selected based on preliminary experiments, expected variability and previous experience. Data collection and analysis were performed blinded to experimental conditions. Statistical analyses were conducted using GraphPad Prism 8. Paired and unpaired two-group comparisons used two-sided Wilcoxon signed-rank and Mann-Whitney *U* tests, respectively. Multiple-group comparisons used one- or two-way ANOVA followed by Tukey’s multiple-comparisons test. Correlations were assessed using two-sided Pearson or Spearman correlation, as appropriate. The mouse was the biological experimental unit, and *n* denotes the number of mice. For electrophysiological analyses, *n* denotes recorded cells, with the number of contributing mice also reported. Further statistical details are provided in the figure legends and Supplementary Table 4. Unless otherwise stated, representative images and traces were reproduced in at least three independent experiments using biologically independent mice, with similar results.

### Reporting summary

Further information on research design is available in the Nature Portfolio Reporting Summary linked to this article.

## Supplementary information

Supplementary Information

Description of Additional Supplementary Files

Supplementary movie 1

Supplementary movie 2

Supplementary movie 3

Supplementary movie 4

Supplementary movie 5

Supplementary movie 6

Reporting Summary

Transparent Peer Review file

## Source data

Source Data

## Data Availability

All data supporting the findings of this study are provided in the paper and its supplementary information. Source data are provided with this paper.

## Code availability

The MATLAB code used for fiber photometry data analysis has been deposited in Zenodo at https://doi.org/10.5281/zenodo.17425988. The custom Python scripts used to generate broadband white noise stimuli have been deposited in Zenodo at https://doi.org/10.5281/zenodo.17426008.
