# Supplementary material for: The Neural Mechanism of Motion Sickness Alleviation by Anticipatory Sound
Source: Nat Commun. 2026 Sep 24;17:11109. doi: 10.1038/s41467-026-77950-x (PMC13696722; doi:10.1038/s41467-026-77950-x)
Supplement: Supplementary file 1 [file 41467_2026_77950_MOESM10_ESM.pdf]

nature portfolio

Peer Review File

The Neural Mechanism of Motion Sickness Alleviation by Anticipatory Sound

Corresponding Author: Dr Congping Shang

This file contains all reviewer reports in order by version, followed by all author rebuttals in order by version.

Version 0:

Reviewer comments:

Reviewer #1

(Remarks to the Author)
Wan et al. present an interesting investigation into the neural mechanisms underlying motion sickness and its mitigation by auditory cues. The authors developed a paradigm using repetitive rotation to induce motion sickness-like (MS-like) behaviors in mice and identified Cbln2+ glutamatergic neurons in the medial vestibular nuclei (MVN) as key players in this process. They describe two distinct output pathways: one to the ventrolateral periaqueductal gray (vlPAG) mediating general MS-like behavior, and another to the nucleus of the solitary tract (NTS) via the parabrachial nucleus (PBN) specifically involved in nausea. Notably, they identify an inhibitory pathway from the central amygdala (CeA) to MVN Cbln2+ neurons that appears to mediate the ameliorative effects of anticipatory sound cues.

General Assessment
This study tackles an important question with a well-controlled experimental design and produces compelling results. However, several conceptual and methodological concerns should be addressed to strengthen the manuscript and clarify the interpretation of the findings.

Major Comments
Conceptualization and Quantification of Motion Sickness:
The current framing and measurements raise some concerns about the validity of the motion sickness model. The primary behavioral readouts—cumulative head orientation angle and average velocity measured over 10 seconds—may not accurately capture motion sickness symptoms. One would expect motion sickness to decrease rather than increase these measures as we move less when motion sick. Furthermore, motion sickness typically manifests as prolonged symptoms, yet the quantification window is limited to 10 seconds following 40 seconds of 5g rotation.
The extreme nature of the stimulus (5g rotation) also warrants discussion. While such intense vestibular stimulation would likely affect any individual regardless of motion sickness susceptibility (analogous to everyone feeling ill during rocket launch), motion sickness is generally understood as a more subtle phenomenon involving sensory mismatch rather than overwhelming vestibular drive (as in why some but not all individuals experience bus-related sickness). Could the authors clarify whether their paradigm specifically models motion sickness or more general vestibular disorientation? Have other motion sickness-related variables been quantified beyond these two measures?
Additionally, the requirement for 5 consecutive days of training before observing effects is puzzling and deserves explanation. What is the rationale for this extended protocol?

Auditory-Vestibular Interaction Mechanism:
The proposed mechanism linking auditory cues to motion sickness alleviation remains unclear. The authors mention anatomical proximity between the inner ear and ear canal, but this does not sufficiently explain the functional connection. Specifically, what is the relationship between the CeA and the auditory system? How do auditory inputs reach and modulate the CeA-MVN pathway? Additional experiments or discussion would strengthen this critical link in the proposed circuit.

Anticipatory Tone Protocol and Rationale:
Several questions arise regarding the tone presentation protocol:
• Does the anticipatory tone affect behavioral variables beyond cumulative head orientation and average velocity?
• If the tone is "anticipatory," why is it also presented at the end of rotation?
• How were the duration and timing of tone presentation relative to rotation determined? Have other laboratories used similar

protocols?
• Have the authors tested variations in tone timing and duration to optimize the effect?

Ablation Efficiency and Behavioral Correlations:
The caspase-mediated ablations appear incomplete (Figures 3t and 5n). It would be valuable to correlate the efficiency of ablation on an animal-by-animal basis with the magnitude of behavioral effects. This analysis would strengthen confidence in the causal relationship between Cbln2+ neuron function and MS-like behavior.

Calcium Imaging Data Presentation and Interpretation:
Figure 4:
• Panel (a-f): Please specify "in vitro electrophysiological" rather than simply "physiological"
• Panel (g): A progressive buildup in calcium signal is apparent across trials. How does this evolve from day 1 to day 10? The goal being to compare it with behavior.
• The panel organization is difficult to follow (2 circles, then 5g 4 circles, then 0.05g, then 0.5g, then behavior). Consider reorganizing to present behavior immediately adjacent to the neural activity data to facilitate correlation
• The rotation patterns used are unclear. Why alternate between one cycle and four cycles?
• The activity pattern suggests encoding of gravity, with maximal response at upside-down position. Do similar oscillations and behavioral effects occur with rotation in other axes? Can the axis of rotation predict behavioral outcomes? This would help determine whether Cbln2+ MVN neurons are specialized for vertical axis processing compared to CCK neurons.

Given that vertical axis (roll) rotation produces larger modulation than horizontal axis (yaw) rotation, could the authors test tilt-axis rotation to determine whether gravity per se is the critical stimulus for activating Cbln2+ neurons, or whether it is simply the specific rotation axis?

Figure 6 (a-c): Why do the protocols differ from those in Figure 4(g-i)? Please comment on the long-lasting effects evident in the 0.5 circles condition, particularly given that the animal should be upside down at this point (If I understood correctly what circle means)
Figure 7:
• Panel (b): Please select one of the two redundant panels; amplitude is easier to interpret
• Panels (c-d): Use consistent color ranges across conditions to facilitate comparison
• Important control: The author should demonstrate that tone does not modulate activity in Cbln2+ neurons in the absence of rotation
• Individual animals likely show variability in both behavioral responses and tone effects. Correlating animal-by-animal GCaMP activity with MS-like behaviors would provide valuable insight into whether Cbln2+ MVN neuron activity directly predicts symptom severity
Figure 8: To establish the mechanistic link between auditory cues and MVN modulation, the authors should perform calcium imaging of CeA neurons projecting to MVN Cbln2+ neurons during both tone presentation and rotation. Specifically:
• Do tone cues activate CeA→MVN projection neurons?
• Why does a 3-second advance presentation provide temporal predictability?
• Are CeA neurons more strongly activated by anticipatory versus concurrent tones?
These experiments are essential for substantiating the proposed CeA-MVN circuit as the mechanism underlying anticipatory tone effects.

Testing Gravity Specificity:
Minor Comments
• Figure 3 and related figures: Please clarify that panels (m-t) in Figure 3 and (i-n) in Figure 5 involve ablation of Cbln2+ neurons rather than chemogenetic manipulation. The current text appears to conflate these approaches.
• For all viral injection experiments, please show the expression distribution along the anterior-posterior axis of the MVN.

Conclusion
This manuscript presents valuable findings regarding the neural circuits underlying vestibular disorientation and its mitigation. Addressing the conceptual concerns about the motion sickness model, completing the mechanistic characterization of the auditory-CeA-MVN pathway, and strengthening the correlative analyses between neural activity and behavior would significantly enhance the impact of this work.

Reviewer #2

(Remarks to the Author)
This manuscript describes how Cbln2+ neurons in the medial vestibular nuclei mediate rotation-induced motion sickness-like behaviors. Anticipatory auditory tones can inhibit motion sickness symptoms, and this is due to central amygdala-to-MVN Cbln2+ inhibitory pathways. The manuscript reports an interesting observation for motion sickness phenotype and charts the underlying circuit, which is highly relevant for understanding motion sickness and nausea. The main conclusions hold and offer novel insight for the field. I have some issues with specific experiments and their related conclusions; hence, these experiments should not be overinterpreted, and the overall conclusions and mechanisms should be adjusted. Main comments:
1. Major mistake in figures and manuscripts for loss-of-function studies. First of all, Fig 3 &5 loss of function is not chemogenetic inactivation, but apoptotic cell ablation. The manuscript keeps saying “inactivation”, demonstrating a lack of

understanding of the cell death involved? Second, the method to validate cell ablation is wrong – you would need to use Cbln2-Cre crossed with a reporter like tdTomato, then inject AAV-DIO-taCasp3-GFP vs AAV-DIO-GFP, and compare the number of tdTomato neurons for validating ablation. Just looking at taCasp3-GFP signals tells you nothing about what's going on with the Cbln2+ cells – you see less green cells, which could be that you injected incorrectly, infected fewer. And also, why are there green cells left anyway? They should be dead? This applies to all loss-of-function studies in Fig3, Fig 5. So I would not overinterpret conclusions drawn from this approach. Unless these experiments are redone with proper controls, whether these neurons are required remains unaddressed.
2. MVN-NTS pathway for nausea. Do you stimulate this pathway optogenetically and look at MS-like rotational behaviors? And do you stimulate MVN-vlPAG and look at CTA behaviors? Terminal stimulation can activate terminals that propagate to cell bodies and activate the whole circuit wherever the cell bodies project to, not restricted to your stimulation site. Unless these site-specific projections are two distinct populations of Cbln2+ neurons, which the authors don't know, I don't think the current methods can suggest that nausea is mediated by MVN-NTS only. In addition, can you do the loss-of-function of Cbln2+ and the CFA test? How important is this pathway to motion nausea vs rotational phenotypes, compared with the MVN CCK+ pathway (which mediates nausea)?
3. The calcium imaging data are also intriguing. In Fig 4 h, why with 5g 4 circles the calcium response is not locked with ventral body back flip, unlike the 0.5g, 4 circles? If these neurons receive body and rotational inputs from vestibular, then the neurons should show body-position specific signals, that is, you see 4 peaks during 5g 4 circles instead of just 1 peak. How do you explain?
4. In Figure 7, what happens if you rotate animals that are trained to have tone responses during rotation, but do not give them the tone? So you can compare the same animal calcium responses with or without tone during rotation. The statistics and effects within animals would be even more powerful.
5. MVN Cbln2+ neurons project to NTS, and rabies tracing shows that they also receive NTS inputs. How to interpret the back-and-forth projections and inputs to and from NTS?
One minor suggestion: Fig 7C and D, you should use the same scale bar for heat map for calcium intensity side by side, so that you can see visually the difference with tone or without.

Reviewer #3

(Remarks to the Author)
In this paper, Wan, Cao, Huo et al. use a custom-made experimental setup to induce Motion Sickness (MS) following whole-body rotation in mice and address the neuronal basis of MS behavior and one prominent symptom, namely MS-induced conditioning taste aversion. They also show that tone cues, which allow animals to anticipate rotations, alleviate MS behaviors. To show this, they first describe the experimental setup and the two protocols to induce 1) classic MS behavior or 2) MS behavior following repetitive exposure to rotation. They next assess the induction of MS behavior using several behavioral readouts (mainly scoring of MS behavior and average velocity and cumulative head orientation angles, measured after MS induction in an open arena) as well as physiological measurements (body temperature) and show that anticipatory tone cues alleviate MS behaviors. They then explore the possible neuronal pathways responsible for the observed behaviors. They use c-fos staining to explore brain areas active during whole-body rotations; focus on the Medial Vestibular Nucleus (MVN); use a plethora of techniques in genetically modified mice (fiber photometry for calcium imaging, optogenetic excitation and inhibition, chemo-genetic activation and inactivation, retro- and antero-grade tracing) to establish Cbln2+ excitatory cells as the main cell-type inducing MS behaviors – particularly circling behaviors. The authors then show that the MVN(Cbln2+) to the ventrolateral periaqueductal (vlPAG) pathway is responsible for the rotation-induced MS behaviors and the MVN- parabrachial nucleus (PBN) -solitary tract (NTS) mediates the MS-induced taste aversion. They finally show that the inhibitory central amygdala (CeA) to MVN(Cbln2+) pathway is involved in the anticipatory tone-induced alleviation of MS behavior. This study is interesting and could be valuable to the wider community, if it wasn't for serious issues – most of it presentational, but not only – which makes its conclusions difficult to evaluate. These issues need to be seriously and thoroughly addressed prior to considering the paper for publication.

Major comments:

The principal issue with the paper is presentational, but not only. Countless grammatical issues render the reading extremely tedious and in cases makes it very difficult to even understand the experimental design. For instance, "Notably, the rotated mice received fixed tone training, but not random tone training, exhibited significantly reduced cumulative head-orientation angles and average velocity on days 6-10 compared with rotated mice exposed only to ambient noise" (lines 122-125). The reader needs to make an effort to understand what is meant by "random tone training", as this is not explained in the main manuscript and, frankly, quite badly in the methods section. Another example: "Besides, according to the MVN single cell expression profiling and the Allen Brain Atlas mRNA in situ hybridization data, Crh+, Chat+ and Cbln2+ could be the proper candidate excitatory neuronal subtypes" (lines 137-139) – regardless of the grammatical mistakes, it is just very difficult to understand what's meant here. Are these the only markers of excitatory cells in the MVN? Did they choose them because they were profiled by the Allen Institute? Why can Cck not be a "proper candidate"? Further down in the Methods section, the authors write: "A mouse was placed in an adequately ventilated 50-mL conical plastic tube coupled to the rotary device, with its head on the axis of rotation (internal radius from mouse head: 5 cm)" (Methods section, line 19). This does not describe correctly the apparatus shown in movie S5 – specifically, the animal's head is not on the axis of rotation, hence an "internal radius from mouse head is 5 cm" (which presumably is from axis of rotation to the head of the animal and, incidentally, does not appear to be 5cm). There are countless examples of these issues. The figures and legends are better, but some aspects absolutely need improvement. A rather worrying issue, for instance, is found in Fig. 5e, f. First, mW is laser power, not intensity. Second, the mCherry gray bar at laser power 5mW (panel e) is the same as the mCherry grey bar at

20Hz (panel f). And the ChR2-mCherry red bar at 20mW (panel e) is the same as the ChR2-mCherry red bar at 20Hz (panel f). It therefore seems that the authors are comparing, for the two right-most bars in panel f, control mCherry 5mW/20Hz versus ChR2-mCherry 20mW/20Hz, which is not correct.

From the experimental design point of view, the biggest caveat of this paper is the unilateral targeting of the MVN (figure 3a-l) and vlPAG projections (figure a-h) for optogenetic/chemo-genetic activation of Cbln2+ neurons. It is impossible to tell if the relevant behavioral readouts, i.e., average velocity and in particular cumulative head-orientation angles and circling behavior, are not due to the unilateral activation of the MVN. This substantial caveat is somehow attenuated by the experiments showing that the bilateral chemo-genetic inactivation of Cbln2+ neurons reduce MS behavior – although the chemo-genetic inactivation of the MVN-vlPAG pathway is also performed unilaterally (figure 5i-n). The authors should provide at least more evidence to claim that MVN Cbln2+ neurons projecting to the vlPAG mediate MS behaviors (Figures 3 and 5). One line of action is to show that the repetitive rotation procedure elicits clockwise and counterclockwise circling behavior and cumulative head-orientation angles in equal proportions. Then, the authors should show that the unilateral activation of MVN Cbln2+ neurons projecting to the vlPAG (figure 3 and 5) results in both CW and CCW circling behaviors (and cumulative head-orientation angles) in equal proportions and independently of the side where the MVN is activated. Ideally, the authors should perform control experiments where MVN Cbln2+ neurons projecting to the vlPAG are bilaterally targeted and show that the MS behavior is similar to experiments where the neurons were unilaterally targeted.

Minor comments:

A few other (less critical) examples of grammatical issues: "Auditory cues generate temporal expectations, predicting when the next step (?) should occur (?)" (line 47), "in addition, MVN neurons coordinate (?) with multisensory integration (?) nuclei such as …" (line 60), "mice in the rotation group exhibited retropulsion or circling symptoms compared to controls after repetitive provocation (?) on day 5" (line 100), "We then performed 10 days of repetitive rotation matched with tone cues in mice to intervene (?) MS-like behavior". "We found that optogenetic activation of the Cbln2+ neurons induced robust MS-like symptoms compared with the other two neurons (?), with a score of 9 and cumulative head-orientation angles of 416 ± 31 during 5 s of stimulation" (line 144). "At the onset of the ventral rotation stimulus, clear transient increases in GCaMP fluorescence were observed in the Cbln2+ MVN-vlPAG pathway, whereas fluorescence in the EGFP-expressing did not alter (?)" (line 272), "This anatomical segregation suggests that these two neuronal populations may subserve disparate (?) behavioral outputs" (line 479), etc., etc., etc.

The authors write: "Therefore, we developed a custom-made rotational paradigm to assess rotation-induced MS-like behavior in mice evoked by 5 g vertical rotation stimulation for 40 s (Fig. 1a and Fig. S1a)" (line 88). Yet the legends of Fig. S1a read: "a Cumulative head orientation angle in a 10-s open field test following 5 g vertical or horizontal rotation for 30 s in mice (Vertical group: n = 4 mice, Horizontal group: n = 5 mice)." So, is it 40 seconds, or 30? If it is 30 seconds (and not a typo), why did the authors change the duration of rotation for the experiments shown in Fig. S1a?

Also, Fig. S1a shows horizontal rotation, but the authors do not explain why they want to test horizontal rotation and nowhere is it mentioned why they decide to keep vertical rotation as their main paradigm. And then, horizontal rotations resurface in Fig S7a-c for no apparent reason. The authors need to explain in the main manuscript why they choose vertical rotation as their protocol to induce MS behavior and once they've made that choice, horizontal rotations should be dropped. If, on the other hand, they absolutely need to show that Cck neuronal activity is also lower compared to Cbln2+ neurons after horizontal rotations, they should justify why. They have to explain that Machuca-Marquez et al. have shown that Cck+ neurons are responsible for MS behaviors, that Machuca-Marquez et al. experimental design is a combination of horizontal and vertical forces, and that, therefore, Cck neuronal responsiveness should be tested for both vertical and horizontal rotations. This needs to be spelt out clearly.

Similarly, the reader suddenly realizes that there are ventral and back-facing rotations when the authors write: "Moreover, quantitative analyses indicated that GCaMP amplitude of the ventral rotation stimulus was higher than the back-facing rotation stimulus, without significant differences in the unit area under the curve (AUC) (Fig. S6b, c)." (line 204). Why have the authors chosen ventral rotations as against back-facing rotations? This should be presented at the beginning of the manuscript, showing that vertical and ventral rotations are the most robust protocol to induce MS behavior, compared to horizontal or back-facing rotations. Once that's established, there is no reason to introduce a new protocol as an afterthought.

The authors can't claim that chemo-genetic inactivation of Cbln2+ neurons "eliminate" MS behavior (Figures 3 and 8 and in several instances in their manuscript) unless they show it – that is, they show that the animals' behavior is statistically not different from controls (animals that were placed in the chamber, but were not rotated).

The activation of Cck+ neurons appear in line 157 of the manuscript without any justification. Previous results (notably Machuca-Marquez et al.) should be discussed in the introduction, so that the readers understand why suddenly the authors are interested in testing the responsiveness of Cck+ neurons. Another possibility would be to add, after the authors have justified their choice of neuronal types using the Allen Institute in situ hybridization data, that another "proper candidate" is Cck+ neurons. This would mean adding this neuronal cell type to the narrative from the beginning (i.e., from figure 3a onwards) and not as an afterthought.

The legends of Figures 1, 2, S3 stipulate that the average velocity is calculated during a 10-min open-field test. Yet, this is not the case in figures 3, 5, 8, S1, S5, S9, S12 and S13 – what time period was used to calculate the average velocity presented in these figures? Regardless of the time-window used to calculate the average velocity, this should be detailed in

the methods section.

The authors claim that “Mice were fed with ad libitum standard chow and water, unless food-deprived overnight for 16-18 h for fasted experiments.” (Methods section, line 10). Yet, in line 24 of Methods, they write: “For the food intake test, animals were individually acclimated to the cage for 3 days prior to stimulation, then fasted with ad libitum access to water for 24 h at the onset of the dark cycle.” So, animals were food deprived for 16-18h or 24h?

All the scoring of MS behaviors needs to be done blind to the procedure imposed on the animals (rotation or control). If the authors have done this blind, they should say so in the methods – otherwise they need to do this analysis blind.

The authors write: “Furthermore, a pronounced reduction in body temperature after rotation provocation compared to controls (rotation versus control, –3.55 versus 0 °C)” (Line 95). What’s the Standard Deviation on these delta temperature averages?

The way how the schematic of the experimental protocol is presented in figure 1g is confusing, it seems as if animals were subjected to rotations only on days 1 to 5, when in fact they were rotated daily from day 1 to 10. Then again, the reader has to dig in the methods section to understand what’s going on: scoring days 1-5, head-oriented angles and velocity analyzed during days 6-10. Which then raises the question, why didn’t the authors do the scoring and analyze head-orientation/velocity for all days?

There is no obvious explanation why the single rotation protocol inducing classic MS behavior results in a decrease in the average velocity of the animals, while the repetitive rotation paradigm induces MS behavior characterized by an increase in the average velocity of the animal. These are diametrically opposite results that should be at least commented upon in the manuscript. Also, it isn’t clear why the authors use average velocity, which is a roundabout way to express distance. Distance is a simpler and more direct measure of locomotion and certainly, when compared to control animals, of “hypolocomotion” (example, line 156).

The authors write: “Here, to identify the brain regions that were activated by rotation stimulus, immunostaining was performed on the expression of the immediate early gene c-Fos. Neuronal activity in the MVN was significantly elevated during the rotation stimulus than during the control stimulus (Fig. 1m)” (line 107-111). Quantification of c-fos expression in the MVN should be in this main figure. Then, the authors write, “The NTS, CeA, PBN, and ventrolateral periaqueductal gray (vlPAG) were also slightly activated (Fig. S2a, b).” “Slightly” is not a quantifiable measure, the authors need to show quantification of c-fos expression in these regions as well (in Fig. S2b).

Spell out SPL (line 120).

Shouldn’t Fig. 2b, f read Fig. 2b-f (line 125)? Shouldn’t Fig. 2c-e include panel f (line 130)?

The schematic in figure 2a does not show 2 seconds of buffer period. Also, the grey zone overlaps with the 3 sec tone at the end, which looks like if rotations last 37 sec instead of 40 sec (this is the same issue with similar schematics in other figures).

In Figure 3 panels b, c and d, the red bars are called differently: Cbln2-Cre (panel a and b) or Cbln2-ChR2 (panel c). Why have the authors decided to call these bars differently? In Fig. 3d, presumably Cbln2-mCherry is the control that does not express ChR2? But then, doesn’t Cbln2-ChR2 express mCherry as well?

In Fig. 3a-d, the authors omitted the average velocity after activation, it should be shown. In Fig. 3j, the authors quantified circling numbers, cumulative head-orientation angles should be shown. Importantly, the authors say that the activation of Cbln2+ neurons “facilitates MS-like behavior”, but the average velocity actually decreases, matching only the single-rotation “classic” MS behavior, not the repetitive rotation MS behavior. Then, the ablation of Cbln2+ neurons also result in a decrease of average velocity, attenuating (not “eliminating”, by the way) repetitive rotation MS behavior. The authors should present and explain these apparent discrepancies clearly and they should be discussed in detail in the Discussion section.

In line 163, the authors write Fig. 3i, it should read Fig. 3l.

The authors write: “Laser-induced cumulative head-orientation angles of MS-like symptoms were correlated with laser intensity and frequency (Fig. 3c, d). A light pulse train (473 nm, 1 ms, 20 mW, 20 Hz) reliably evoked phase-locked spiking activity in Cbln2+ neurons expressing ChR2-mCherry (Fig. 3e).” The authors jump from in vivo behavioral optogenetic experiments to in vitro brain physiology, but do not tell the reader. By the way, the protocol on slices also includes 10Hz, not only 20Hz.

Figure 4d, can the authors clarify what they are measuring? The scale bar reads 25 Hz, which is wrong. What are the black traces, what is the red trace?

The legend of Fig. 4g reads “Ca2+ signals associated with the trial of MS-like behavioral session. Heatmap showing 27 trials of normalized GCaMP fluorescence changes (△F/F) aligned with MS-like behavior induced by ventral rotation (top)”. It is impossible to understand what the authors mean by “aligned with MS-like behavior”. It is, presumably, aligned to the onset of rotation.

In general, the colocalization figures should be explained better (Figures S4, S5, S6, S11). Example, in Fig. S6a, the legend reads “The proportion of co-localized neurons in Cbln2 mRNA and GCamp6s+ neurons (n = 8 fields from 4 mice)”, which is

already confusing, and then the bars are labeled “Co/GCamp6s+” and “Co/Cbln2+”. It isn’t clear enough and the Methods section does not help.

The authors write: “In contrast, we did not observe changes in fluorescent intensity in EGFP-expressing Cbln2+ MVN neurons, indicating that the recorded signals were not due to motion artifacts.” Where is the reader supposed to see this? The reader is supposed to look at figure 4g, but then, what’s the red trace, what’s the grey trace?

The schematic in Figure 4i is totally useless. What needs to be shown, and in fact above all photometry traces, is the rotation angle of the chamber over time – or even better, acceleration over time. Related to this, the authors should explain precisely how they measured acceleration.

All quantifications of peak deltaF/F in function of acceleration should be incorporated in the main figures, not left in supplementary figures.

The authors do not explain what the pharmacological cocktail applied on brain slices show. Also, the authors have not performed in vivo electrophysiology, as claimed in the authors contributions section.

Figure 5f, in the legends, the authors call correctly panel e “laser power”, but wrongly in the figure itself; and then panel f legends refers to “intensity”, when it should read “frequency”.

The authors write: “Interestingly, at the onset of the rotation with a 3-s tone stimulus on day 17, pronounced decreases in GCaMP fluorescence were observed in the Rotation+tone group compared with the Rotation group (Fig. 7c).” Suddenly, the analysis is performed in day 17, no explanation given. Why not in day 10? Is it because rotations start in day 6 (that is, 6 days after virus injection) and, therefore, what they call day 17 is actually day 11 after the beginning of the repetitive rotation paradigm?

Version 1:

Reviewer comments:

Reviewer #1

(Remarks to the Author)
I thank the authors for their careful responses. The revised manuscript satisfactorily addresses the concerns raised in my initial review.

I have two remaining minor suggestions.

First, regarding the statistical analyses: several comparisons rely on a Student's t-test, which assumes normally distributed data. Given the small sample sizes in these experiments, normality cannot be reliably assessed, and I would recommend a non-parametric alternative. For paired comparisons between two conditions, a Wilcoxon signed-rank test would be more appropriate.

Second, regarding readability: several sentences in the manuscript are long and difficult to parse. I would encourage the authors to break these into shorter, more direct statements, which would substantially improve the clarity of the text.

Reviewer #2

(Remarks to the Author)
The authors have addressed my concerns. The revised manuscript is much better and clearer. The authors should also be applauded for the enormous amount of data included in this manuscript.

Reviewer #3

(Remarks to the Author)
The authors have answered effectively to most of the concerns raised in the previous review and the manuscript is significantly improved. The paper is fine now.

Open Access This Peer Review File is licensed under a Creative Commons Attribution 4.0 International License, which permits use, sharing, adaptation, distribution and reproduction in any medium or format, as long as you give appropriate credit to the original author(s) and the source, provide a link to the Creative Commons license, and indicate if changes were made.
In cases where reviewers are anonymous, credit should be given to 'Anonymous Referee' and the source.
The images or other third party material in this Peer Review File are included in the article's Creative Commons license, unless indicated otherwise in a credit line to the material. If material is not included in the article's Creative Commons license and your intended use is not permitted by statutory regulation or exceeds the permitted use, you will need to obtain permission directly from the copyright holder.
To view a copy of this license, visit https://creativecommons.org/licenses/by/4.0/

Response to Reviews

Manuscript ID: NCOMMS-25-83869

Title: The Neural Mechanism of Motion Sickness Alleviation by Anticipatory Sound

We sincerely appreciate the positive and helpful evaluations provided by the editor and Reviewers. Guided by these thoughtful comments, we have completed an extensive set of additional experiments to address their specific concerns. We have also revised the manuscript where appropriate, incorporating content germane to the comments of each Reviewer. A revised version of the manuscript with tracked changes (highlighted in blue) has been uploaded as a separate file. The detailed changes and our point-by-point responses to each of the Editor's or Reviewers' questions and comments are presented below.

Contents:

Response to Reviewer #1: Page 02-24

Response to Reviewer #2: Page 25-38

Response to Reviewer #3: Page 39-63

Reference: Page 64-67

Response to Reviewer' comments:

Reviewer #1 (Remarks to the Author):

Wan et al. present an interesting investigation into the neural mechanisms underlying motion sickness and its mitigation by auditory cues. The authors developed a paradigm using repetitive rotation to induce motion sickness-like (MS-like) behaviors in mice and identified Cbln2$^+$ glutamatergic neurons in the medial vestibular nuclei (MVN) as key players in this process. They describe two distinct output pathways: one to the ventrolateral periaqueductal gray (vlPAG) mediating general MS-like behavior, and another to the nucleus of the solitary tract (NTS) via the parabrachial nucleus (PBN) specifically involved in nausea. Notably, they identify an inhibitory pathway from the central amygdala (CeA) to MVN Cbln2$^+$ neurons that appears to mediate the ameliorative effects of anticipatory sound cues.

General Assessment

This study tackles an important question with a well-controlled experimental design and produces compelling results. However, several conceptual and methodological concerns should be addressed to strengthen the manuscript and clarify the interpretation of the findings.

Major Comments

1. Conceptualization and Quantification of Motion Sickness:

1) The current framing and measurements raise some concerns about the validity of the motion sickness model. The primary behavioral readouts—cumulative head orientation angle and average velocity measured over 10 seconds—may not accurately capture motion sickness symptoms. One would expect motion sickness to decrease rather than increase these measures as we move less when motion sick. Furthermore, motion sickness typically manifests as prolonged symptoms, yet the quantification window is limited to 10 seconds following 40 seconds of 5 g rotation.

RESPONSE: We would like to thank you for your positive comments regarding our

manuscript and for your guidance towards improving our study. We agree that cumulative head orientation angle and average velocity should not be regarded as standalone measures that fully capture the entire MS state, and that the rationale for focusing on the 10 s post-rotation window should be clarified more explicitly. First, we established an acute single-rotation paradigm to validate that our rotation protocol reliably induces classical MS-like symptoms. Following a single 5 *g* rotation, mice exhibited prolonged hypoactivity, behavioral immobility, and significant reductions in both appetite and body temperature. These sustained autonomic and behavioral alterations closely mirror the classic MS symptoms reported in the rodents[1-3], confirming the successful induction of an acute MS-like state. In addition to these prolonged symptoms, mice displayed immediate tumbling behavior after rotation in the acute paradigm, reflecting an acute vestibular disequilibrium response. However, this acute response was not reliably modulated by a single anticipatory auditory cue in our pilot experiments.

To enable more robust behavioral quantification and assess predictive modulation, we therefore developed a repeated rotation paradigm. Across repeated exposures, mice exhibited a progressive change in behavioral phenotype, evolving from tumbling and retropulsive symptoms during days 1-5 to transient directed circling behavior during days 6-10, which was then followed by prolonged hypoactivity. Importantly, this post-rotation circling represents an immediate, short-lived (~10-20 s), directional locomotor response that occurs directly following vestibular stimulation. Therefore, the cumulative head rotation angle and average velocity measured immediately after rotation were used as quantitative indices of the acute vestibular-triggered motor response, rather than as standalone measures of the global MS state. To sensitively detect and compare group differences within this brief but behaviorally critical window, we specifically analyzed the first 10 s post-rotation. Because circling inherently involves active locomotion, average velocity transiently increases during this window. Thus, the increased velocity in this short window reflects a specific acute motor output rather than an attenuation of the overall MS-like state. This differs from the acute

paradigm, which assesses prolonged post-stimulation effects.

To further assess the broader MS-like state, we have included data on autonomic responses (food intake and body temperature) across the 10-day repeated rotation paradigm (detailed in response to the next comment). Our results show that repeatedly rotated mice exhibit significant and sustained reductions in both appetite and body temperature compared to controls, consistent with classical MS symptoms. These findings confirm that although the circling phenotype is captured within the immediate post-rotation window, the broader MS-like state persists throughout the paradigm. Together, these results demonstrate that the repeated rotation model not only induces a quantifiable acute vestibular-driven circling response but also induces sustained autonomic features characteristic of MS. Notably, we further observed that the anticipatory tone cue not only attenuated repeated rotation-induced circling behavior but also improved appetite. We have clarified this distinction in the revised manuscript to ensure readers can properly interpret our findings.

2) The extreme nature of the stimulus (5 g rotation) also warrants discussion. While such intense vestibular stimulation would likely affect any individual regardless of motion sickness susceptibility (analogous to everyone feeling ill during rocket launch), motion sickness is generally understood as a more subtle phenomenon involving sensory mismatch rather than overwhelming vestibular drive (as in why some but not all individuals experience bus-related sickness). Could the authors clarify whether their paradigm specifically models motion sickness or more general vestibular disorientation? Have other motion sickness-related variables been quantified beyond these two measures?

RESPONSE: We sincerely thank you for raising this important point. We agree that 5 *g* rotation constitutes a relatively strong vestibular stimulus and that its physiological effects are not fully equivalent to the more subtle form of MS that arises in everyday settings from mild sensory conflict and marked inter-individual susceptibility. Specifically, MS encompasses a range of conditions that can be triggered by

provocative motion stimuli, including altered gravitoinertial forces, off-vertical axis rotation, centripetal acceleration, and spaceflight. Depending on the exposure context, it is commonly referred to as seasickness, carsickness, airsickness, space MS, or simulator sickness[4-6]. We have therefore further clarified the scope and interpretation of our model in the revised manuscript.

Our primary reason for using this stimulus intensity was to establish a stable, reproducible, and quantifiable vestibular stimulation paradigm in mice. High-*g* rotational paradigms have been widely used in rodent MS research to reliably evoke MS-like responses[7,8]. For example, a recent study investigating vestibular CCK signaling in MS used 4 *g* rotational acceleration to reliably evoke MS-like behaviors in mice[9]. Thus, high-*g* rotational stimulation represents an established experimental strategy for generating consistent vestibular perturbation in rodent models. From a mechanistic perspective, MS results from a mismatch between expected and actual sensory inputs about self-movement[10-12]. A key subsidiary hypothesis is that the severity of MS is thought to scale with the magnitude and persistence of sensory conflict. In our paradigm, 5 *g* vertical rotation generates a robust sensory conflict by maximally engaging the gravitoinertial/otolith-related signals during rotation, thereby eliciting robust MS-like responses. On this basis, we believe that our paradigm is more appropriately described as a model of strong vestibular stimulation-induced MS-like responses, rather than a complete recapitulation of the milder sensory-mismatch conditions that underlie everyday MS.

Importantly, we also quantified additional physiological correlates of MS beyond locomotor-related measures. Vestibular stimulation is known to induce characteristic autonomic responses associated with MS, including appetite suppression, loss of body temperature, and conditioned taste avoidance (CTA)[1-3]. Therefore, we measured food intake and body temperature in mice subjected to the repeated rotation paradigm for 10 consecutive days. Notably, repeatedly rotated mice exhibited a significant reduction in both appetite and body temperature on days 1 and 10, compared to non-rotated controls. These findings further support that repeated rotation paradigm reliably induces MS-like

autonomic responses in addition to the behavioral phenotypes used for acute quantification (Response Document Figure 1, and also see new Figure 1l,m). These clarifications and additional points have been incorporated into the revised manuscript.

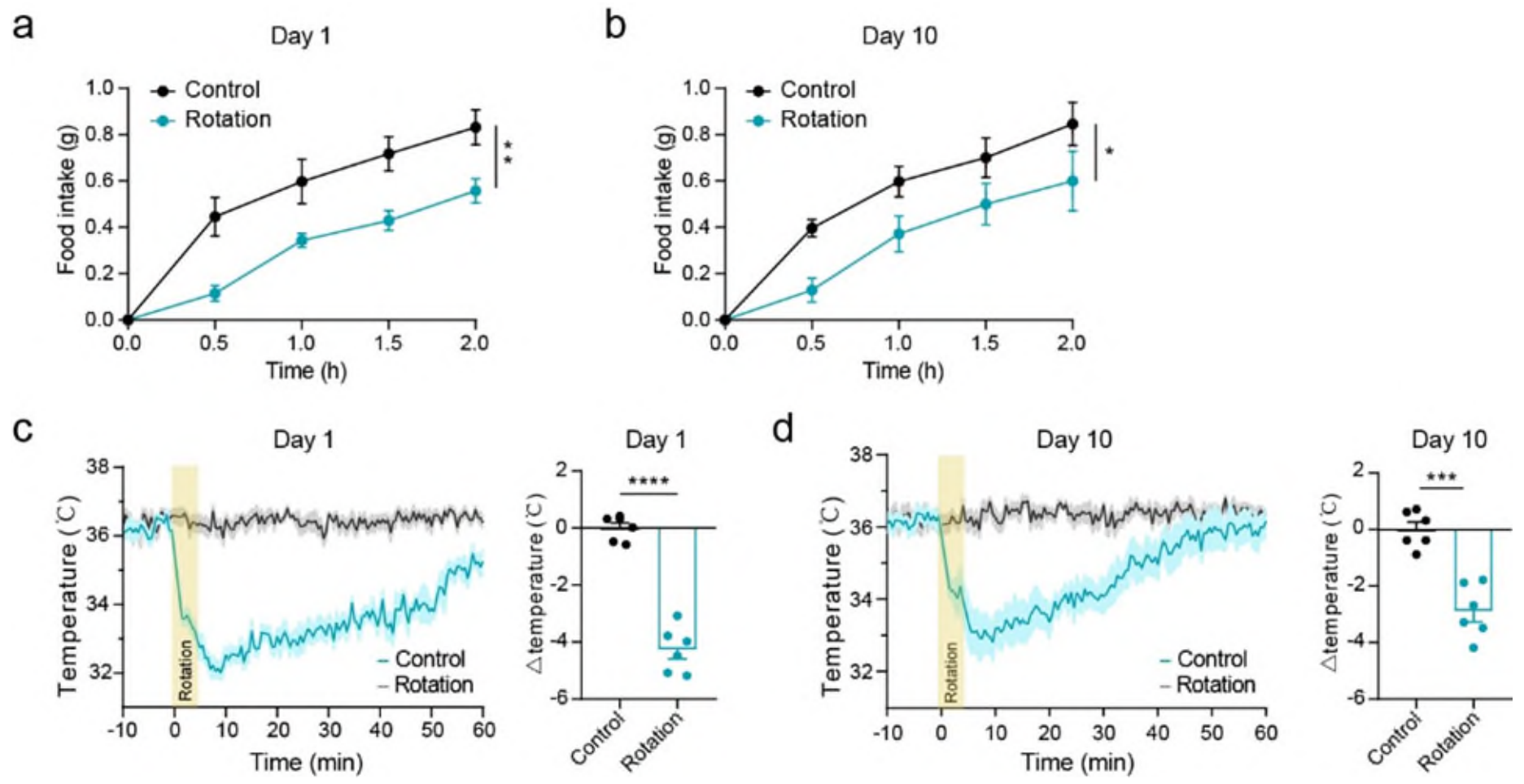

Response Document Figure 1: Effect of repeated rotation-induced MS-like behavior on appetite and body temperature in mice. a,b, Repeated rotation-induced cumulative food intake in hungry mice on days 1 and 10 (*n* = 6 mice; two-sided Student's t-test, *P < 0.05, **P < 0.01). **c,d,** Body temperature changes over 1 h (left) and body temperature differences at 10 min (right) after rotation or control stimulation in mice on days 1 and 10 (*n* = 6 mice; two-sided Student's t-test, ****P < 0.0001).

3) Additionally, the requirement for 5 consecutive days of training before observing effects is puzzling and deserves explanation. What is the rationale for this extended protocol?

RESPONSE: Thank you for your important comment. These 5 consecutive days were not intended simply to prolong stimulus exposure. Rather, the purpose of the training phase was to allow mice to form a stable association between the anticipatory auditory cue and the subsequent rotational stimulus. Adaptation to repeated motion stimuli is a fundamental characteristic of MS across species. Studies have shown that continuous or intermittent rotation leads to progressive attenuation of MS symptoms[13-18], with the time course of adaptation varying considerably across species and stimulation protocols. In our study, we observed that mice exposed to daily 5 *g* vertical rotation underwent a progressive behavioral transition: from initial tumbling symptoms to the emergence of retropulsive movement (days 1-5) and eventually circling behavior (days 6-10). This

temporal evolution reflects the gradual establishment of vestibular habituation.

Importantly, investigating the influence of anticipatory tone cues on MS-like behavior involves an associative learning process that is inherently time-dependent. Cue-conditioned modulation of behavioral responses requires repeated pairings over multiple days to establish stable predictive associations. The 10-day protocol allows sufficient trials for mice to form robust associations between the auditory cue and the impending rotational stimulus, enabling us to assess how learned expectations modulate vestibular responses. We have clarified this rationale in the revised Methods sections.

2. Auditory-Vestibular Interaction Mechanism:

The proposed mechanism linking auditory cues to motion sickness alleviation remains unclear. The authors mention anatomical proximity between the inner ear and ear canal, but this does not sufficiently explain the functional connection. Specifically, what is the relationship between the CeA and the auditory system? How do auditory inputs reach and modulate the CeA-MVN pathway? Additional experiments or discussion would strengthen this critical link in the proposed circuit.

RESPONSE: We sincerely thank you for your incredibly valuable guidance. We agree that the auditory and vestibular systems are anatomically close and both transmit sensory information to the brain via the eighth cranial nerve; these structural features alone are unlikely to account for the anticipatory sound–mediated modulation of MS examined here. Auditory cues can contribute to spatial orientation and balance–a concept often referred to as the "auditory anchor"[19,20]. This audio-vestibular interaction is likely mediated by shared central pathways; both modalities converge in brainstem nuclei (e.g., the MVN) and are integrated for self-motion perception and postural control[21]. Because the anatomical proximity explanation was not essential to the interpretation of our findings and could potentially be misleading, we have removed this description from the revised manuscript and clarified the conceptual framework accordingly.

Accumulating evidence indicates that acoustic experiences, such as sound or noise,

induce structural and functional changes not only in the central auditory system but also in limbic regions, including the amygdala and hippocampus[22-26]. The amygdala is particularly sensitive to sound with valence or meaning and plays a central role in auditory fear conditioning and the regulation of emotional behavioral responses[27-31]. Importantly, the amygdala receives direct neural inputs from both the auditory thalamus (medial geniculate nucleus, MGN) and the auditory cortex[32]. Moreover, the central nucleus of the amygdala (CeA) serves as a key output station that integrates diverse sensory inputs and regulates automatic or autonomic functions[33,34]. We previously identified an inhibitory CeA-MVN pathway that contributes to the anticipatory tone-induced alleviation of repeated rotation-evoked MS-like behavior. To investigate the connection between auditory cues and the CeA-MVN pathway, we performed retrograde tracing experiments. Specifically, we injected Cre-dependent helper AAVs into the CeA of *Vgat*-Cre mice, followed by RV-EGFP into the MVN two weeks later. We observed retrogradely labeled $EGFP^+$ neurons in the CeA, confirming the existence of direct inhibitory projections from CeA to MVN. Moreover, we found that $Vgat^+$ CeA neurons receive inputs from multiple brain regions, including the somatosensory cortex (S1/S2), insular cortex (GI/DI), and notably, the auditory cortex (ACx) (Response Document Figure 2). These findings provide an anatomical basis for auditory information to reach the CeA may through the central auditory cortex and further influence the CeA-MVN pathway.

Based on these anatomical and functional data, we propose the following mechanism: When an anticipatory tone cue is presented, auditory signals are transmitted to central auditory pathways, including the ACx, which in turn activates $Vgat^+$ CeA neurons via direct cortico-amygdalar projections. These CeA neurons then project to the brainstem MVN, where they release GABA. This auditory-driven, feedforward inhibitory signal enhances inhibitory postsynaptic currents (IPSCs) in $Cbln2^+$ MVN neurons, effectively dampening vestibular hyperexcitability during rotation. This expectancy-based mechanism alleviates MS-like behavior. A more detailed discussion of how the CeA-MVN pathway responds to anticipatory tone cues,

including calcium imaging data from this circuit, is provided in our response to the final Major Comment (addressing CeA-MVN pathway calcium imaging). We thank the reviewer for prompting this important mechanistic clarification, which we have incorporated into the revised Discussion section.

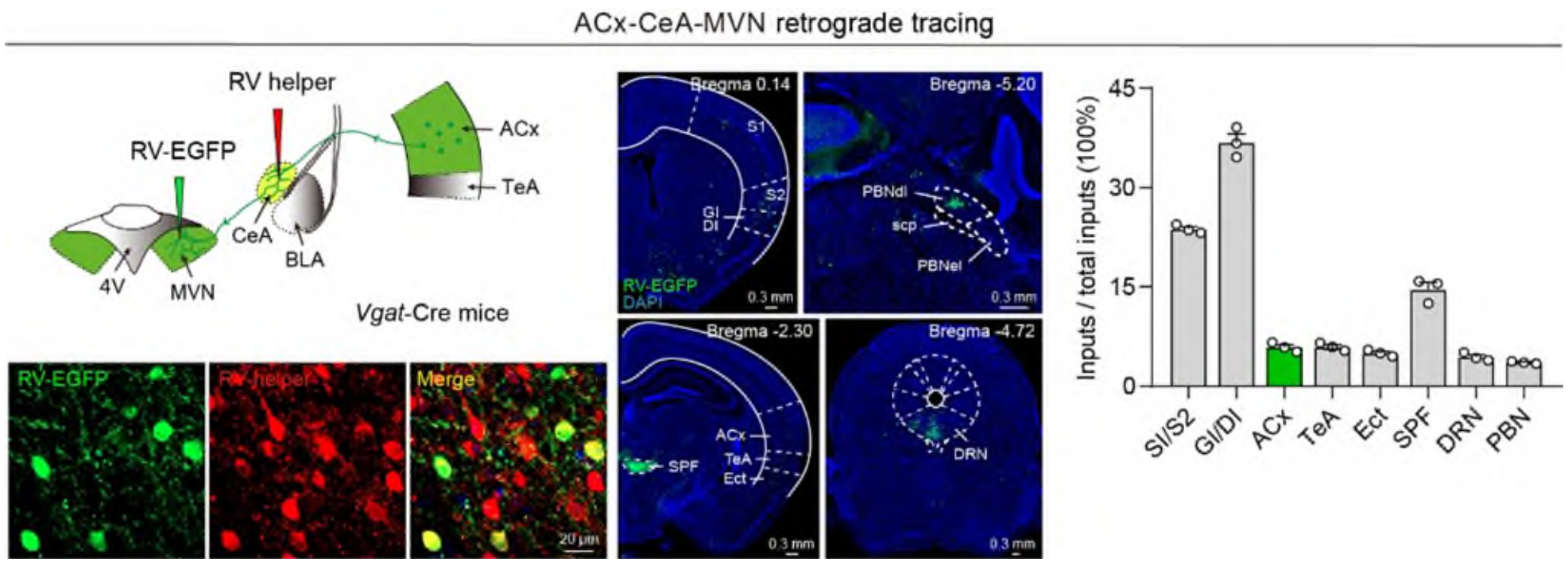

Response Document Figure 2: Retrograde tracing of ACx-CeA-MVN pathway with RV.

3. Anticipatory Tone Protocol and Rationale:

Several questions arise regarding the tone presentation protocol:

• Does the anticipatory tone affect behavioral variables beyond cumulative head orientation and average velocity?

RESPONSE: We thank you for this constructive comment. It is well known that motion-induced MS mediates responses in behavioral and autonomic signs, such as hypolocomotion, appetite suppression, loss of body temperature, or acquisition of a CTA. In response, we have conducted new experiments to investigate the effect of anticipatory tone on appetite and body temperature. We performed repeated rotation matched with tone cues in mice for 10 consecutive days. On day 1, the rotated mice (including fixed or random tone training) showed a marked decrease in both food intake and body temperature compared to non-rotated controls. Notably, on day 10, those that received fixed tone training (but not random tone training) exhibited significantly improved food intake compared to the rotated mice exposed only to ambient noise. However, body temperature did not differ among the three rotated groups (Response Document Figure 3, and also see new Figure 2g,h, and Extended Data Figure 2b,c). These results demonstrated that anticipatory tone does influence other behavioral variables (specifically, appetite) while the effect on body temperature was not

significant in the fixed tone training group. These additional data have been incorporated into the revised manuscript.

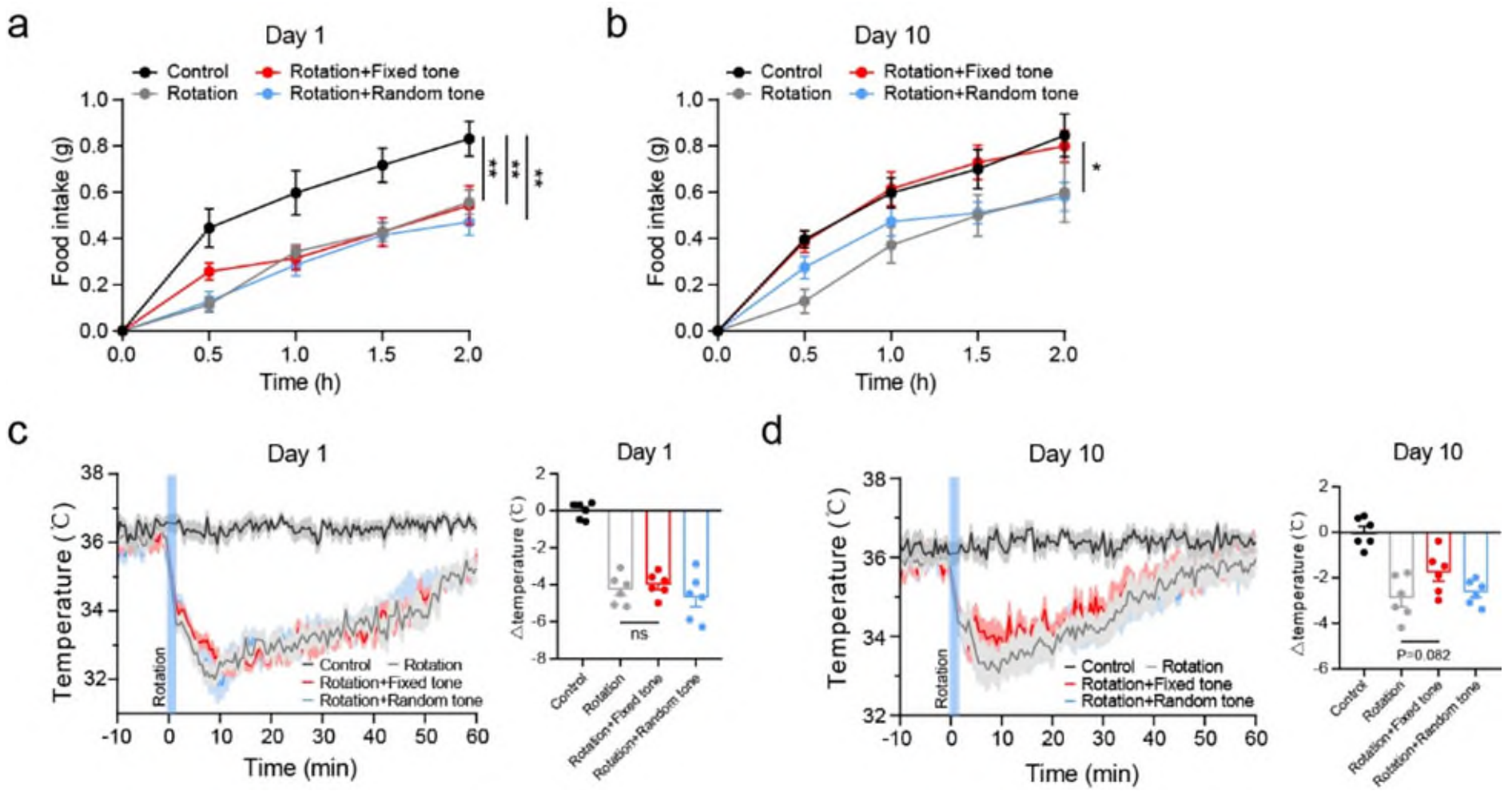

Response Document Figure 3: Effect of anticipatory tone on appetite and body temperature in repeatedly rotated mice. a,b, Repeated rotation paired with tone cues-induced cumulative food intake in hungry mice on days 1 and 10 (*n* = 6 mice; one-way ANOVA, *P < 0.05, **P < 0.01). **c,d,** Body temperature changes over 1 h (left) and body temperature differences at 10 min (right) after rotation paired with tone cues or control stimulation in mice on days 1 and 10.

• If the tone is "anticipatory," why is it also presented at the end of rotation?

• How were the duration and timing of tone presentation relative to rotation determined? Have other laboratories used similar protocols?

• Have the authors tested variations in tone timing and duration to optimize the effect?

RESPONSE: Thank you for your important comment. We presented the 3-s tone both before the onset and before the termination of rotation because both phases involve abrupt changes in motion dynamics. By presenting the auditory cue shortly before each change in motion state, the mice could potentially use the tone as a predictive signal for salient vestibular events during the rotation cycle. This design helps the mice actively adjust their behavioral responses to motion onset and offset and supports the formation of stable cue–motion associations.

The 3-s duration of the auditory tone was chosen based on commonly used parameter ranges in studies of auditory perception, associative learning, and conditioning in rodents. Previous work has shown that auditory cues lasting several seconds are sufficient for reliable detection and for supporting cue–outcome associative

learning in mice and rats[35-37]. Based on this literature, as well as results from our pilot experiments, we selected a 3-s tone that is easily perceived by the animals and can be consistently embedded within the temporal structure of the rotational paradigm.

Given that our 3-s tone already produced significant effects on MS-like behavior, we did not perform a systematic optimization of tone duration or timing. However, we agree that a systematic comparison of different cue durations and lead times would help define the optimal conditions under which predictive auditory cues modulate motion-induced responses. Accordingly, in the revised manuscript, we acknowledge this point as a limitation of the present study and note in the Discussion that future work will be needed to examine a broader range of cue durations and cue-motion intervals to determine the parameter range that maximizes the modulatory effect of predictive auditory signals.

4. Ablation Efficiency and Behavioral Correlations:

The caspase-mediated ablations appear incomplete (Figures 3t and 5n). It would be valuable to correlate the efficiency of ablation on an animal-by-animal basis with the magnitude of behavioral effects. This analysis would strengthen confidence in the causal relationship between Cbln2+ neuron function and MS-like behavior.

RESPONSE: We are grateful for your insightful comments and suggestions. We performed NeuN immunostaining to quantify the density of neurons within the targeted region following viral expression. Several prior studies have used NeuN staining to confirm neuronal loss after caspase-based ablation, demonstrating that Cre-dependent taCasp3 expression leads to a marked reduction of NeuN-positive neurons in the targeted population[38-41]. Additionally, to further improve the efficiency and reliability of ablation, we optimized the viral strategy following previously published protocols[38]. We replaced the virus and used a higher-titer (titer: 4.80×10^{12} genome copies per ml).

Specifically, for ablating the $Cbln2^{+}$ MVN neurons, we bilaterally injected AAV2/9-Flex-taCasp3-TEVp or AAV-DIO-EGFP into the MVN of *Cbln2*-IRES-Cre mice. Two weeks after viral expression, the mice underwent repeated rotation

stimulation for 10 consecutive days. Strikingly, we found that mice with apoptosis of Cbln2$^+$ MVN neurons exhibited significant suppression of MS-like behavior, including decreased behavioral evaluation scores, cumulative head-orientation angles, and average velocity, compared with EGFP (non-apoptosis) mice. Importantly, ablation of Cbln2$^+$ MVN neurons also improved appetite compared to the EGFP group on day 10. Representative movement trajectories on day 10 are shown. We further quantified ablation efficiency by counting the number of surviving neurons, and found that ablation efficiency was linearly correlated with cumulative head-orientation angles on day 10 (Response Document Figure 4, and also see new Figure 3). These findings underscore that Cbln2$^+$ MVN neurons were the primary component required for rotation-induced MS-like behavior.

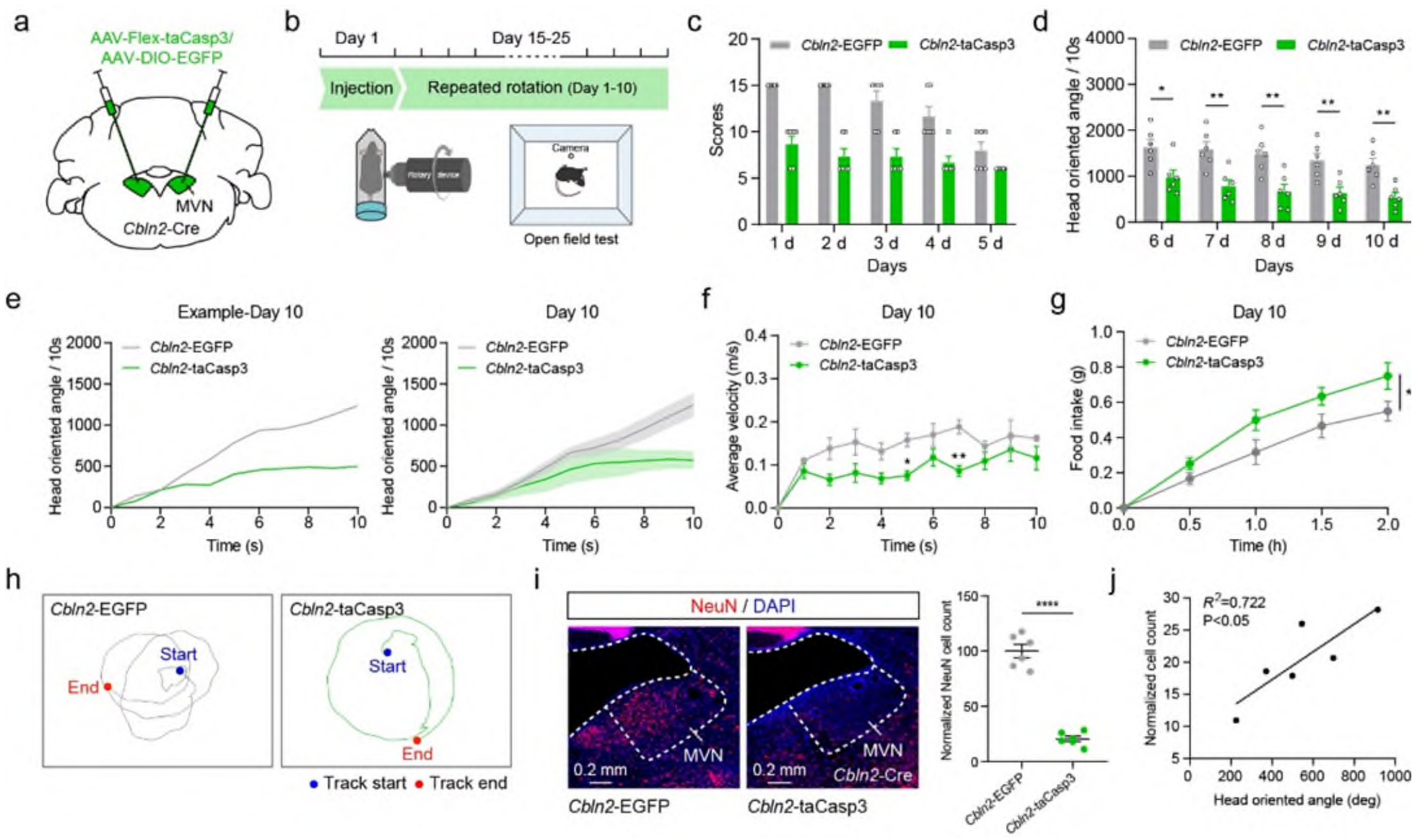

Response Document Figure 4: Cbln2$^+$ MVN neurons are essential for rotation-induced MS-like behavior. a, Viral strategy for targeted ablation of Cbln2$^+$ MVN neurons. **b,** Schematic of the repeated rotation paradigm applied to mice with or without Cbln2$^+$ neuron ablation. **c,d,** Time course of behavioral evaluation scores (c) and cumulative head orientation angles (d) during the first 10 s post-rotation across 10 days (Cbln2-EGFP: n = 6, Cbln2-taCasp3: n = 6). **e-f,** Representative curves of cumulative head orientation angles (e) and average velocity (f) during the first 10 s open-field test following rotation with or without ablation of Cbln2$^+$ neurons in mice on day 10. **g,** Cumulative food intake in hungry mice on day 10 following rotation with or without ablation of Cbln2$^+$ neurons in mice. **h,** Representative curves of movement trajectories during the first 10 s post-rotation on day 10. **i,** Example coronal brain section and summarized for the number of neurons in Cbln2$^+$ MVN neurons in *Cbln2*-IRES-Cre mice. **j,** Scatter plot showing the efficiency of Cbln2$^+$ neurons ablation linearly correlated with cumulative head-orientation angles on day 10. Statistical analyses in (d) and (f) were performed using two-way ANOVA (*P < 0.05, **P < 0.01, ****P < 0.0001). Statistical

analyses in (g) and (i) were performed using a two-sided Student's t-test (****P < 0.0001).

For ablating the Cbln2+ MVN-vlPAG pathway, we bilaterally injected AAV2-retro-DIO-flp into the vlPAG and AAV-fDIO-taCasp3-TEVp or AAV-fDIO-EGFP into the MVN of *Cbln2*-IRES-Cre mice. Three weeks after viral expression, the mice underwent repeated rotation stimulation for 10 consecutive days. Similarly, we found that ablation of the Cbln2+ MVN-vlPAG pathway largely reduced behavioral evaluation scores, cumulative head-orientation angles, and average velocity relative to non-ablated controls. However, ablation of this circuit had no significant effect on appetite compared to the EGFP group on day 10. Representative movement trajectories of both groups following rotation on day 10 are shown. Also, the efficiency of this pathway ablation was quantified by summarizing the number of surviving neurons, and found that ablation efficiency was linearly correlation with cumulative head-orientation angles on day 10 (Response Document Figure 5, and also see new Figure 5). These results imply that the Cbln2+ MVN-vlPAG pathway is sufficient and required for rotation-induced MS-like behavior.

We have replaced the original results with these new data in the revised manuscript, including the relationship between ablation efficiency and behavioral effects.

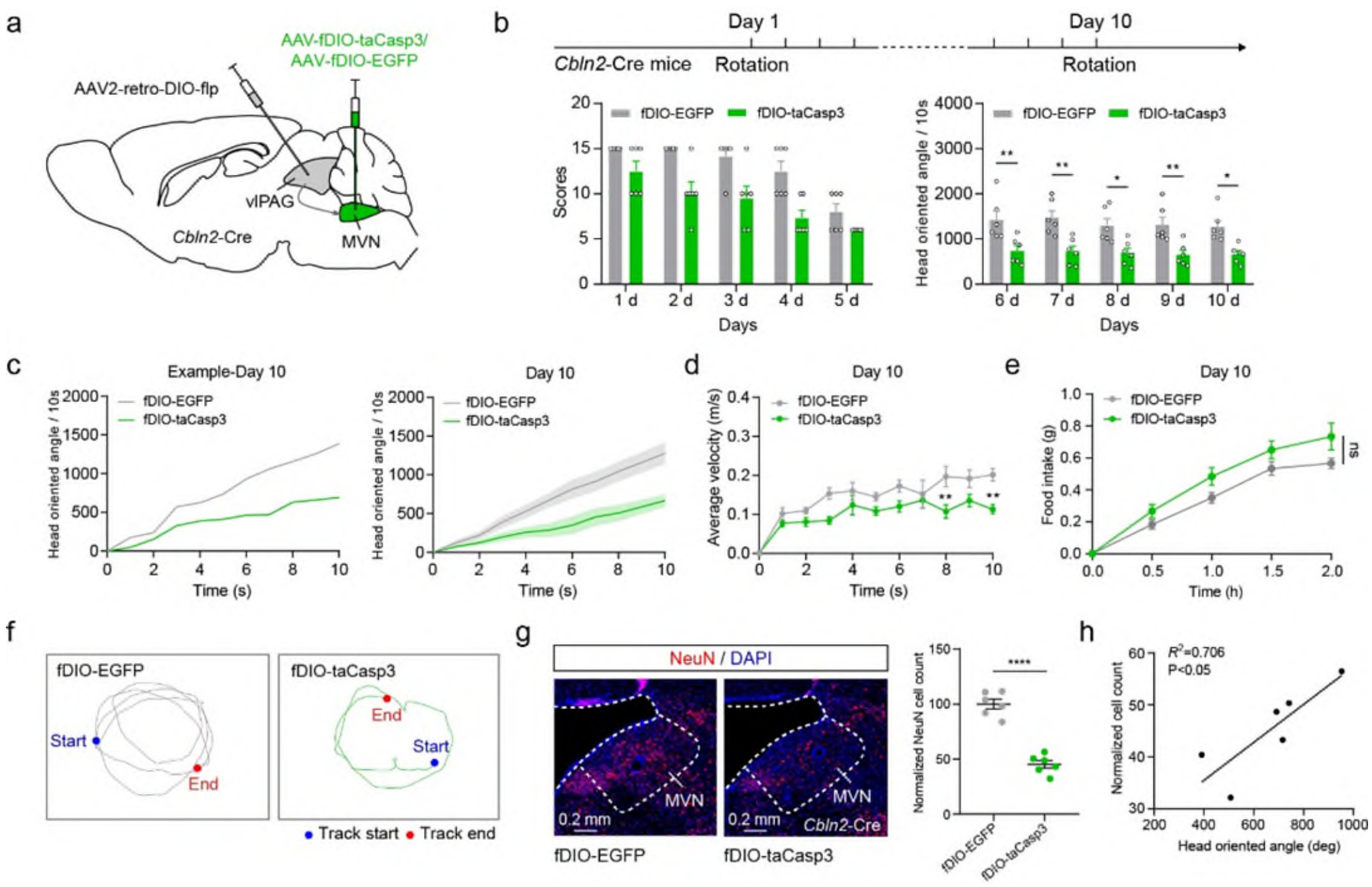

Response Document Figure 5: Cbln2+ MVN-vlPAG pathway is critical for MS-like behavior.

a, Viral injection strategy to express taCasp3 or EGFP in vlPAG-projecting Cbln2$^+$ MVN neurons in *Cbln2*-IRES-Cre mice. **b,** Time course of behavioral evaluation scores (left) and cumulative head orientation angles (right) in mice during the first 10 s post-rotation across 10 days in control (EGFP) and ablated (taCasp3) mice (n = 6 per group). **c-d,** Representative curves of cumulative head orientation angles (c) and average velocity (d) during the first 10 s open-field test following rotation with or without ablation of Cbln2$^+$ MVN-vlPAG neurons in mice on day 10. **e,** Cumulative food intake in hungry mice on day 10 following rotation with or without ablation of Cbln2$^+$ MVN-vlPAG neurons in mice. **f,** Representative curves of movement trajectories during the first 10 s post-rotation on day 10 in control (EGFP) and ablated (taCasp3) mice. **g,** Example coronal brain section and summarized for the number of neurons in vlPAG-projecting Cbln2$^+$ MVN neurons in *Cbln2*-IRES-Cre mice. **h,** Scatter plot showing the efficiency of Cbln2$^+$ MVN-vlPAG pathway ablation linearly correlated with cumulative head-orientation angles on day 10. Statistical analyses in (b) and (d) were performed using two-way ANOVA (*P < 0.05, **P < 0.01). Statistical analyses in (e) and (g) were performed using a two-sided Student's t-test (****P < 0.0001).

5. Calcium Imaging Data Presentation and Interpretation:
Figure 4:
• Panel (a-f): Please specify "in vitro electrophysiological" rather than simply "physiological".

RESPONSE: As suggested, the title of Panel (a–f) has been revised to "In vitro electrophysiological characteristics of Cbln2$^+$ neurons."

• Panel (g): A progressive buildup in calcium signal is apparent across trials. How does this evolve from day 1 to day 10? The goal being to compare it with behavior.

RESPONSE: We apologize for the confusion caused by our imprecise description in the legend of Figure 4g. The data presented in Figure 4g represent pooled trials from 4 mice, with each mouse contributing multiple trials. To generate this panel, we sorted all trials from lowest to highest based on peak fluorescence intensity. This sorting approach reveals the full dynamic range of calcium responses across the population but does not reflect a temporal progression within individual animals. The observed variability in calcium signal amplitude across trials can be attributed to several factors: First, mouse-to-mouse variability in baseline neuronal activity and sensitivity to rotational stimulation. Second, differences in viral expression efficiency and fiber placement relative to GCaMP-expressing neurons affect the absolute fluorescence signal detected.

Additionally, we have previously examined whether calcium signal intensity changes systematically from day 1 to day 10. Our experiments showed that while there was a

slight, non-significant trend toward increased fluorescence intensity over the 10-day period due to continued viral expression, this effect was minimal in the group-to-group comparison. We have updated the figure legend to clarify that the trial sorting in Figure 4g is for visualization of the dynamic range.

• The panel organization is difficult to follow (2 circles, then 5g 4 circles, then 0.05g, then 0.5g, then behavior). Consider reorganizing to present behavior immediately adjacent to the neural activity data to facilitate correlation.

•The rotation patterns used are unclear. Why alternate between one cycle and four cycles?

RESPONSE: Thank you for your rigorous review and constructive suggestion. In response, we have reorganized Figure 4 panels g–i to place the behavioral data immediately adjacent to the corresponding neural activity data. Additionally, a single cycle of 0.05 *g* rotation was initially employed to observe changes in the body position of mice under sufficiently slow rotation. However, to maintain consistency with the four-cycle rotation pattern used for the 0.5 *g* and 5.0 *g* conditions, we have updated data for the 0.05 *g* rotation administered over 4 circles in the revised manuscript (Response Document Figure 6, and also see new Figure 4).

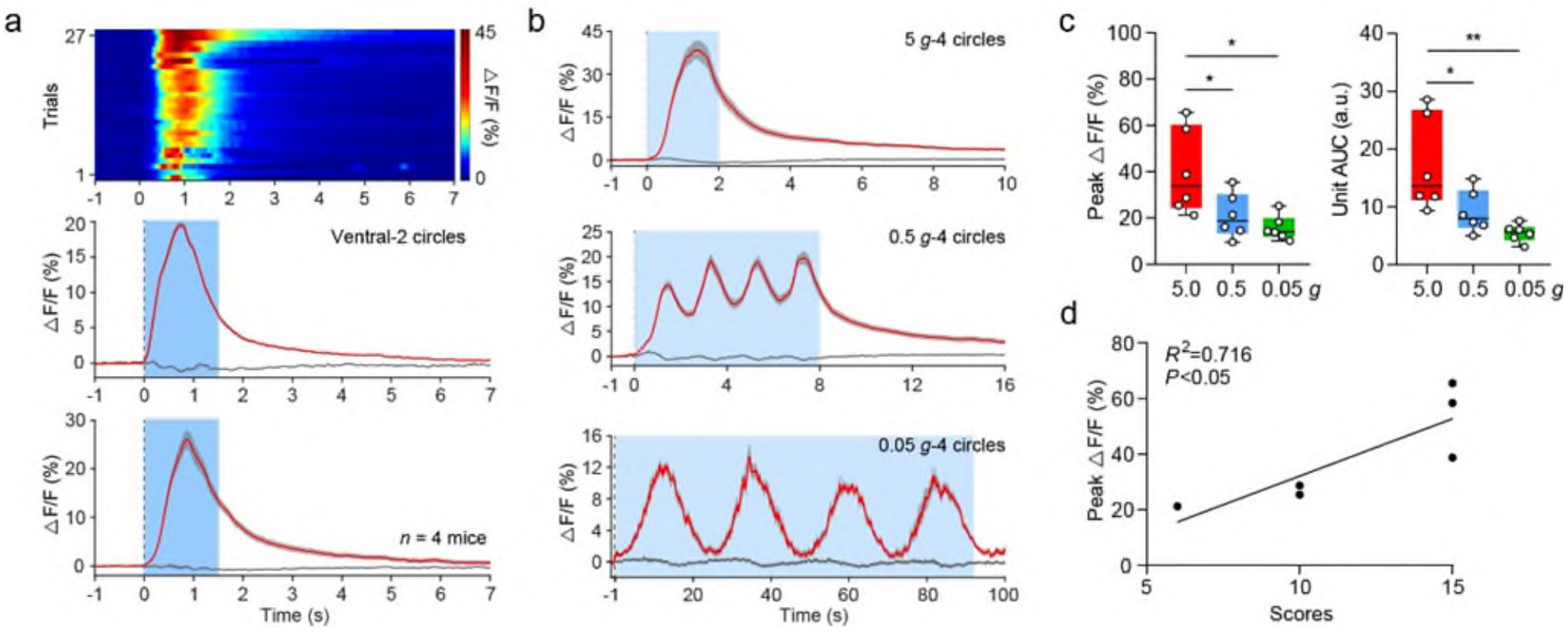

Response Document Figure 6: a, $Ca2^+$ signals associated with the trial of MS-like behavioral session. Heatmap of normalized GCaMP fluorescence changes (ΔF/F) across 27 trials from 4 mice, aligned to ventral rotation onset (5 *g*, 2 circles) (top); normalized GCaMP signal changes in $Cbln2^+$ MVN neurons during MS-like behavior induced by ventral rotation in individual mice (middle); mean $Ca2^+$ transients for the entire test group (*n* = 4 mice) (bottom). **b,** GCaMP signal responses to ventral rotations of different intensities (5 *g*, 0.5 *g*, and 0.05 *g*, 4 circles) (*n* = 6 mice). Red lines indicate recordings from $GCaMP6s^+$ cells, gray lines indicate recordings from reference $EGFP^+$

cells. Thick lines represent the mean, and shaded areas indicate s.e.m. Gray dashed line: onset of ventral rotation; Blue shaded areas: duration of ventral rotation. **c,** Quantitation of peak amplitude (left) and unit AUC (right) of GCaMP responses during ventral rotations of different intensities (*n* = 6 mice; one-way ANOVA, *P < 0.05, **P < 0.01). **d,** GCaMP responses of the vertical rotation (5.0 *g*, 4 circles) linearly correlated with the severity of MS-like behavioral symptoms on a per-animal basis.

• The activity pattern suggests encoding of gravity, with maximal response at upside-down position. Do similar oscillations and behavioral effects occur with rotation in other axes? Can the axis of rotation predict behavioral outcomes? This would help determine whether Cbln2+ MVN neurons are specialized for vertical axis processing compared to CCK neurons. Given that vertical axis (roll) rotation produces larger modulation than horizontal axis (yaw) rotation, could the authors test tilt-axis rotation to determine whether gravity per se is the critical stimulus for activating Cbln2+ neurons, or whether it is simply the specific rotation axis?

RESPONSE: We appreciate your meticulous review and for providing us with such an important hint. As suggested, we performed additional calcium imaging experiments using horizontal and tilt axis (45°) rotation paradigms (0.5 *g*, 4 circles). We observed that both horizontal and tilt rotations also evoked significant GCaMP signals in Cbln2$^+$ MVN neurons, indicating that these neurons are not selectively activated by a specific rotation axis. Notably, tilt-axis rotation also elicited oscillatory calcium dynamics similar to those evoked by vertical rotation, albeit with lower amplitude (Response Document Figure 7a).

However, the calcium signals evoked by horizontal rotation were significantly lower than those evoked by vertical rotation. To further test whether increased rotational load alone could account for this difference, we progressively increased the horizontal rotational acceleration up to 9.0 *g* (4 circles). Even under this higher acceleration, the evoked calcium signal still did not reach the magnitude observed during 5.0 *g* vertical rotation (Response Document Figure 7b). We speculate that the activity of Cbln2$^+$ MVN neurons can be explained by two major components. One component is a rotation-related inertial component, F(n), which reflects the acceleration/load associated with

rotation velocity n. The other is a gravity-related component, G(θ), which depends on the angle θ between the mouse head orientation and the horizontal plane. In this framework, F(n) primarily determines the baseline level of Cbln2$^+$ neuronal activity, whereas G(θ) determines the peak-to-trough oscillatory modulation of the response. Importantly, this component-based analysis could roughly predict the calcium signals observed during tilt-axis and vertical rotation from the calcium dynamics measured during horizontal rotation, yielding consistently high correlation coefficients between the fitted and measured signals especially for the first rotation cycle, thereby supporting the validity of this interpretation (Response Document Figure 7c).

These results indicate that, as you speculated, the response of Cbln2$^+$ neurons likely integrate rotational acceleration with gravity-related information about body orientation, rather than simply encoding one fixed rotation axis. This is consistent with the established role of otolith-canal convergence in vestibular nuclei neurons[42,43], in which vertical rotations produce robust activation by engagement with changes in gravitational orientation.

Additionally, we have included animal-by-animal correlation analysis for the 5.0 *g* (4 circles) vertical rotation (Response Document Figure 7d). We found a linear relationship between GCaMP fluorescence changes and behavioral symptoms, demonstrating that the activity level of Cbln2$^+$ MVN neurons during vertical rotation directly predicts the severity of MS-like behavioral symptoms.

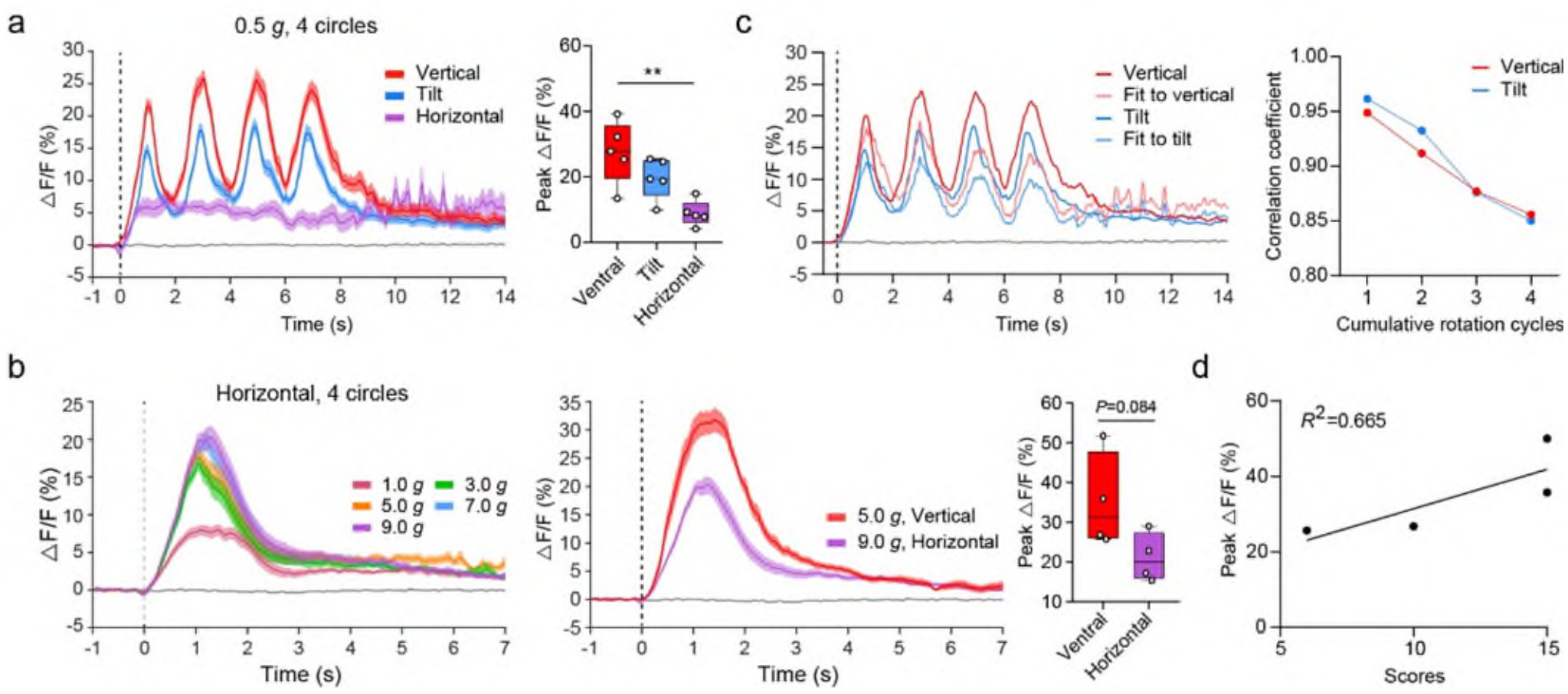

Response Document Figure 7: GCaMP responses of Cbln2$^+$ MVN neurons to different axial rotations. a, Mean GCaMP fluorescence (left) and quantification of peak amplitude (right) in response to different axial rotations (0.5 *g*, 4 circles). **b,** Mean GCaMP fluorescence (left) in

response to horizontal rotation of different intensities. Mean GCaMP fluorescence (right) in response to vertical (5.0 *g*) and horizontal (9.0 *g*) rotations. **c,** Left: Mean calcium responses during tilt and vertical rotation predicted from horizontal-rotation calcium signals. The red trace indicates the mean measured calcium signal during vertical rotation, and the light red trace indicates the corresponding fitted signal. The blue trace indicates the mean measured calcium signal during tilt-axis rotation, and the light blue trace indicates the corresponding fitted signal. Right: Line plots of the correlation coefficients between measured and fitted calcium signals across cumulative rotation cycles. The blue line represents the correlation coefficients between the measured and fitted tilt-axis rotation calcium signals, whereas the red line represents the correlation coefficients between the measured and fitted vertical-rotation calcium signals. The x-axis indicates the cumulative number of rotation cycles included in the analysis. **d,** GCaMP responses of the vertical rotation (5.0 *g*, 4 circles) linearly correlated with the severity of MS-like behavioral symptoms.

Figure 6 (a-c): Why do the protocols differ from those in Figure 4(g-i)? Please comment on the long-lasting effects evident in the 0.5 circles condition, particularly given that the animal should be upside down at this point (If I understood correctly what circle means).

RESPONSE: Thanks for your careful review. Figure 4 (g-i) used a multi-cycle rotation paradigm to examine the relationship between neural activity and the behavioral output. In contrast, Figure 6 (a-c) was designed to dissect how the Cbln2^{+} MVN-vlPAG pathway responds to the transient dynamics produced by motion stimulus and the change in body orientation relative to gravity during rotation. Under the 0.5 circles condition, the mouse is indeed in an upside-down (supine) position, and this abnormal orientation relative to gravity is maintained. Therefore, although the transient rotational component has ended, the gravity-related postural mismatch persists, which likely accounts for the sustained calcium signal observed under this condition. To address this concern and to align the experimental design with Figure 4 (g-i), we have updated the data obtained using the 4 circles paradigm under the 5 *g*, 0.5 *g*, and 0.05 *g* conditions. These updated results show that the activity of this pathway increases progressively with rotational intensity. These revised data have been incorporated into the revised manuscript (Response Document Figure 8c,d, and also see new Figure 6c,d).

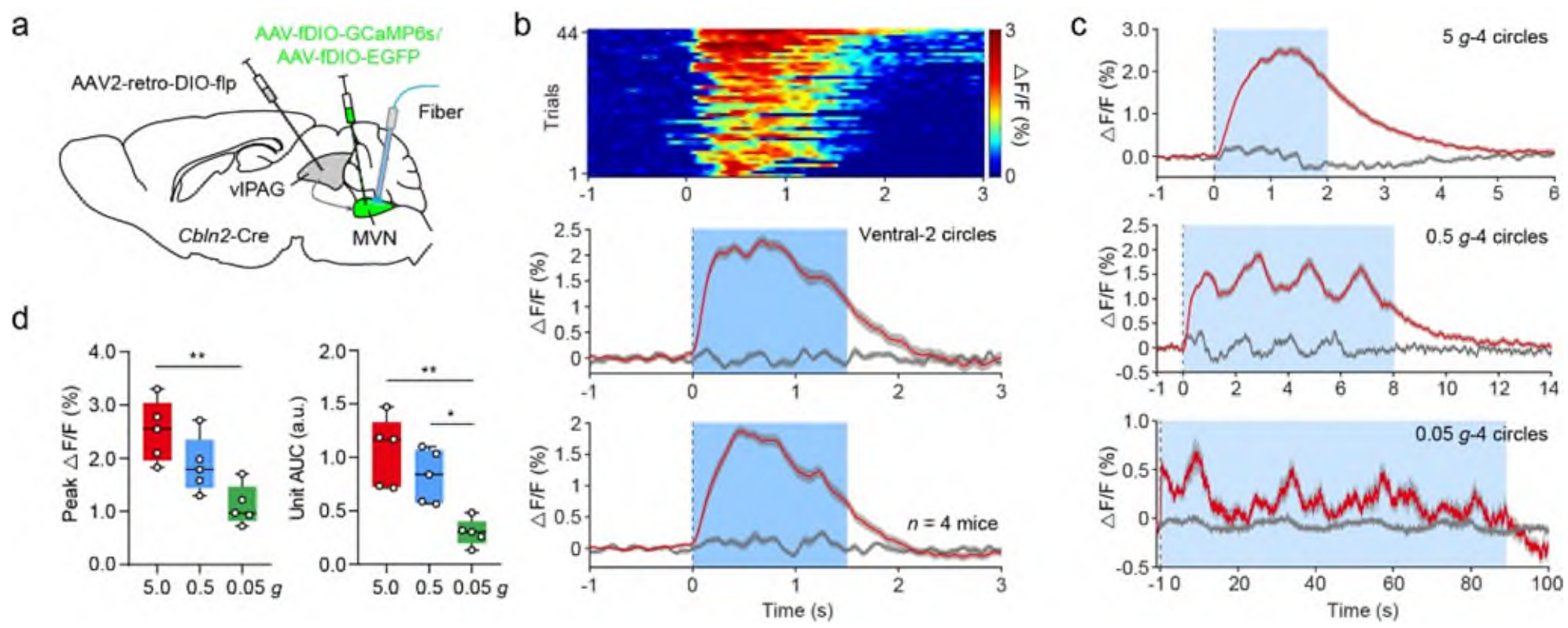

Response Document Figure 8: Calcium imaging of vlPAG-projecting Cbln2⁺ MVN neurons during ventral rotation. a, Viral injection strategy to express GCaMP6s in vlPAG-projecting Cbln2⁺ MVN neurons in *Cbln2*-IRES-Cre mice, followed by optical fiber implantation above the MVN. **b,** Ca2⁺ signals associated with the trial of MVN-vlPAG pathway-mediated MS session. Heatmap of normalized GCaMP fluorescence changes (ΔF/F) across 44 trials from 4 mice, aligned to ventral rotation onset (5 *g*, 2 circles) (top); normalized GCaMP signal changes in vlPAG-projecting Cbln2⁺ MVN neurons during MS-like behavior induced by ventral rotation in individual mice (middle); mean Ca2⁺ transients for the entire test group (*n* = 4 mice) (bottom). **c,** GCaMP signal responses of the Cbln2⁺ MVN-vlPAG pathway to ventral rotations of different intensities (5 *g*, 0.5 *g*, and 0.05 *g*, 4 circles) (*n* = 4 mice). Red lines indicate recordings from GCaMP6s⁺ cells, gray lines indicate recordings from reference EGFP⁺ cells. Thick lines represent the mean, and shaded areas indicate s.e.m. Gray dashed line: onset of ventral rotation; Blue shaded areas: duration of ventral rotation. **d,** Quantitation of peak amplitude (left) and unit AUC (right) of GCaMP responses during ventral rotations of different intensities (*n* = 4 mice; one-way ANOVA, *P < 0.05, **P < 0.01).

Figure 7:

• Panel (b): Please select one of the two redundant panels; amplitude is easier to interpret.

• Panels (c-d): Use consistent color ranges across conditions to facilitate comparison.

RESPONSE: Thank you for your helpful suggestions regarding figure presentation. For panel (b), we have removed the peak-to-valley amplitude difference graph and added a new graph showing the relationship between GCaMP responses and behavioral symptoms. For panels (c-d), we have used consistent color ranges across all conditions in the calcium signal heatmaps to facilitate direct comparison.

• Important control: The author should demonstrate that tone does not modulate activity in Cbln2+ neurons in the absence of rotation.

RESPONSE: Thank you for your important comment. We have supplemented new data showing that tone exposure does not modulate the activity of Cbln2^{+} neurons in the absence of rotation. These additional results have been incorporated into the revised manuscript (Response Document Figure 9, and also see new Extended Data Figure 10a).

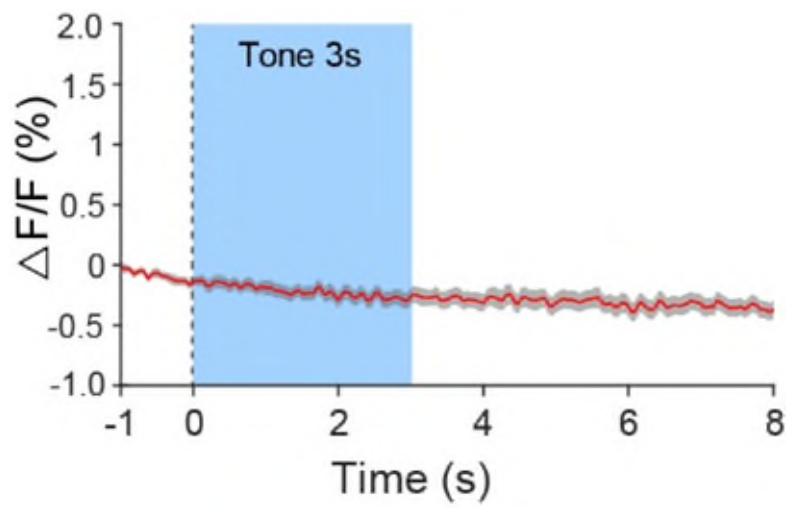

Response Document Figure 9: GCaMP signal responses to tone exposure in the absence of rotation.

• Individual animals likely show variability in both behavioral responses and tone effects. Correlating animal-by-animal GCaMP activity with MS-like behaviors would provide valuable insight into whether Cbln2+ MVN neuron activity directly predicts symptom severity.

RESPONSE: Thank you for your valuable and important guidance. We quantified the relationship between GCaMP fluorescence changes and cumulative head-orientation angles on an animal-by-animal basis in the Rotation+tone group on day 10. Our analysis revealed a linear correlation between these two measures. This finding provides direct evidence that the activity of Cbln2^{+} MVN neurons is closely linked to the MS-like behavior. These additional results have been incorporated into the revised manuscript (Response Document Figure 10, and also see new Figure 7c).

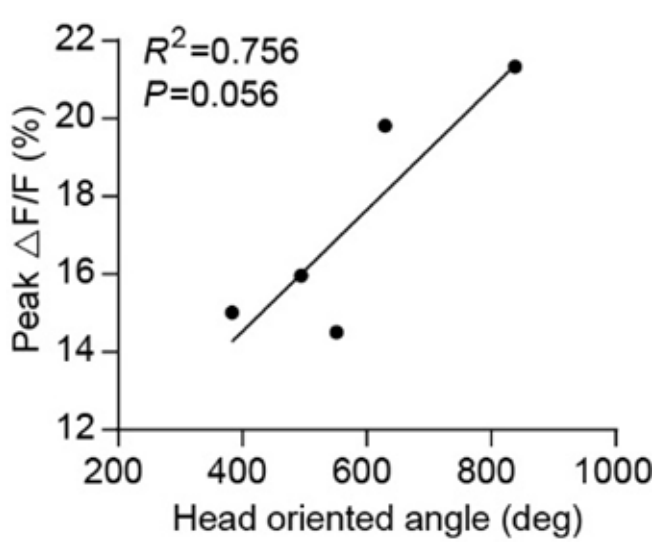

Response Document Figure 10: GCaMP fluorescence changes of the Rotation+tone group linearly correlated with cumulative head-orientation angles on day 10.

Figure 8: To establish the mechanistic link between auditory cues and MVN modulation, the authors should perform calcium imaging of CeA neurons projecting to MVN Cbln2+ neurons during both tone presentation and rotation. Specifically:

• Do tone cues activate CeA→MVN projection neurons?

• Why does a 3-second advance presentation provide temporal predictability?

• Are CeA neurons more strongly activated by anticipatory versus concurrent tones?

These experiments are essential for substantiating the proposed CeA-MVN circuit as the mechanism underlying anticipatory tone effects.

RESPONSE: Thank you for your helpful and thought-provoking guidance. As suggested, we performed calcium imaging to monitor the activity of the CeA-MVN pathway in response to anticipatory tones. Given our previous finding that the inhibitory CeA-MVN pathway contributes to tone-induced alleviation of repeated rotation-evoked MS-like behavior, we injected AAV2-retro-DIO-flp into the MVN and AAV-fDIO-GCaMP6s into the CeA of *Vg*at-IRES-Cre mice, followed by implantation of an optical fiber above the axon terminals in the CeA (Response Document Figure 11a, and also see new Figure 8f). Three weeks after viral expression, mice underwent 10 consecutive days of repeated rotation, either with or without anticipatory tone pairing. On day 10, both groups (Rotation alone and Rotation+Tone) received tone presentation before rotation onset. Strikingly, the Rotation+Tone group exhibited higher GCaMP fluorescence in $Vgat^{+}$ CeA-MVN projection neurons compared to the Rotation group. On day 11, when both groups underwent rotation without tone presentation, there was no significant difference in GCaMP signals between the two groups. However, within the Rotation+Tone group, GCaMP signals were lower on day 11 (Non-tone) than on day 10 (tone present) (Response Document Figure 11b-e, and also see new Figure 8g-i). The activity of these neurons was negatively correlated with the severity of MS-like behavior in the Rotation+Tone group on day 10 (Response Document Figure 11c, and also see new Figure 8j). Importantly, on day 10, we compared GCaMP signals within the Rotation+Tone group under anticipatory tone versus concurrent tone conditions. While concurrent tone stimulation also elevated GCaMP fluorescence, the increase was

not significant when compared to either the anticipatory tone condition or the no-tone control group (Response Document Figure 11f). These results indicate that anticipatory tone reliably enhances the activation of the $Vgat^+$ CeA-MVN pathway during rotation, thereby contributing to the alleviation of MS-like behavior.

We also tested whether tone presentation alone (without rotation) could activate CeA-MVN projection neurons. We found that a 3 s tone cue delivered in the absence of rotation increased GCaMP fluorescence in $Vgat^+$ CeA-MVN neurons compared to baseline, confirming that auditory cues alone can engage this pathway and establishing a direct functional link between auditory input and MVN modulation via the CeA (Response Document Figure 11g, and also see new Extended Data Figure 11h).

The rationale for how the 3-s advance presentation provides temporal predictability has been addressed in our response to Major Comment #3. In brief, this 3-s interval constitutes a brief but functionally effective anticipatory window, allowing the auditory cue to engage the CeA-MVN pathway prior to the onset of the vestibular stimulus. While we acknowledge that other temporal parameters might also be effective, our findings indicate that this 3-s window is functionally sufficient to support predictive learning under our experimental conditions. This interpretation is corroborated by our control experiments using randomly presented tones, in which the behavioral modulation was mitigated when the fixed temporal relationship between the tone and rotation was disrupted. We have incorporated these new data into the revised manuscript to further support the mechanism by which the predictive tone exerts its effects via the CeA-MVN circuit.

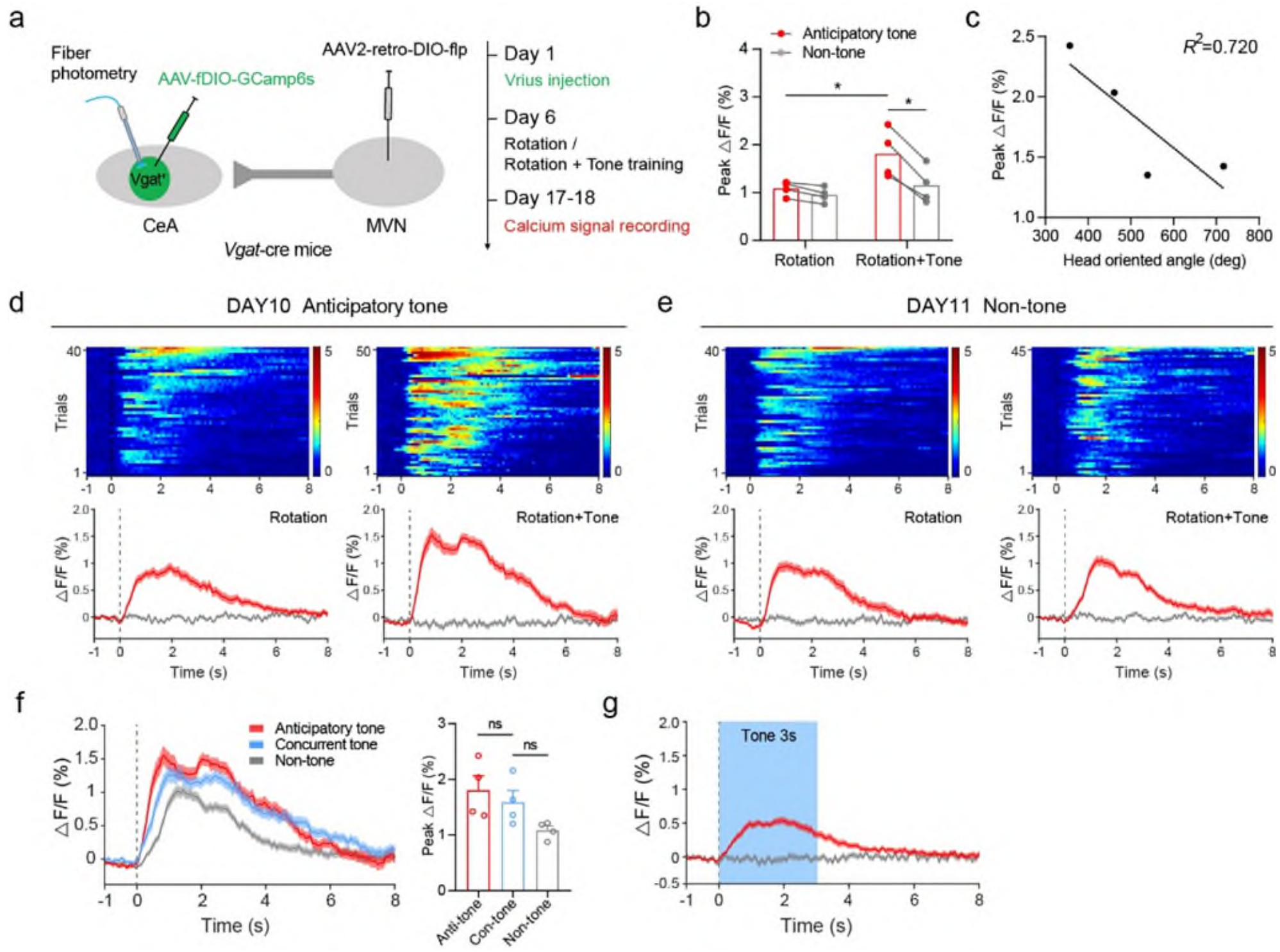

Response Document Figure 11: Anticipatory tone presentation modulates rotation-induced MS-like behavior via the Vgat+ CeA-MVN pathway. a, Schematic diagram of viral injection and optical fiber implantation for projection-specific calcium imaging in the CeA-MVN inhibitory pathway in *Vgat*-IRES-Cre mice. **b,** Quantitation of peak GCaMP responses in Vgat+ CeA-MVN projection neurons during rotation with or without anticipatory tone in test (Rotation+Tone) and control (Rotation) mice (n = 4 mice per group; two-way ANOVA, $*P < 0.05$). **c,** Linear negative correlation between peak GCaMP responses and cumulative head-orientation angles in the Rotation+Tone group on day 10. **d,** Heatmaps of normalized GCaMP fluorescence changes ($\Delta F/F$) and average GCaMP response curve in Vgat+ CeA-MVN projection neurons aligned to the onset of rotation with anticipatory tone in test and control mice on day 10. Red lines indicate recordings from GCaMP6s+ cells, gray lines indicate recordings from reference 405 nm. Thick lines represent the mean, and shaded areas indicate s.e.m. Gray dashed line: onset of ventral rotation; Blue shaded areas: duration of ventral rotation. **e,** Same as (d), but aligned to rotation onset on day 11 (no tone presentation). **f,** Comparison of GCaMP responses in Vgat+ CeA-MVN projection neurons during rotation and rotation with tone in Rotation+Tone mice on day 10. **g,** GCaMP signal responses to tone exposure in the absence of rotation.

6. Testing Gravity Specificity:

Minor Comments

• Figure 3 and related figures: Please clarify that panels (m-t) in Figure 3 and (i-n) in Figure 5 involve ablation of Cbln2+ neurons rather than chemogenetic manipulation. The current text appears to conflate these approaches.

RESPONSE: Thank you for pointing out the critical errors in our work. We apologize

for our oversight in the inaccurate terminology used in our original manuscript. We have carefully revised the entire manuscript accordingly, replacing all instances of "inactivation" with "ablation".

• For all viral injection experiments, please show the expression distribution along the anterior-posterior axis of the MVN.

RESPONSE: As suggested, we injected AAV-DIO-hM3D-mCherry, AAV-DIO-chR2-mCherry, or AAV-DIO-GCamp6s into the MVN of *Cbln2*-IRES-Cre mice and have presented the expression distribution of Cbln2^{+} neurons along the anterior-posterior axis of the MVN (Response Document Figure 12).

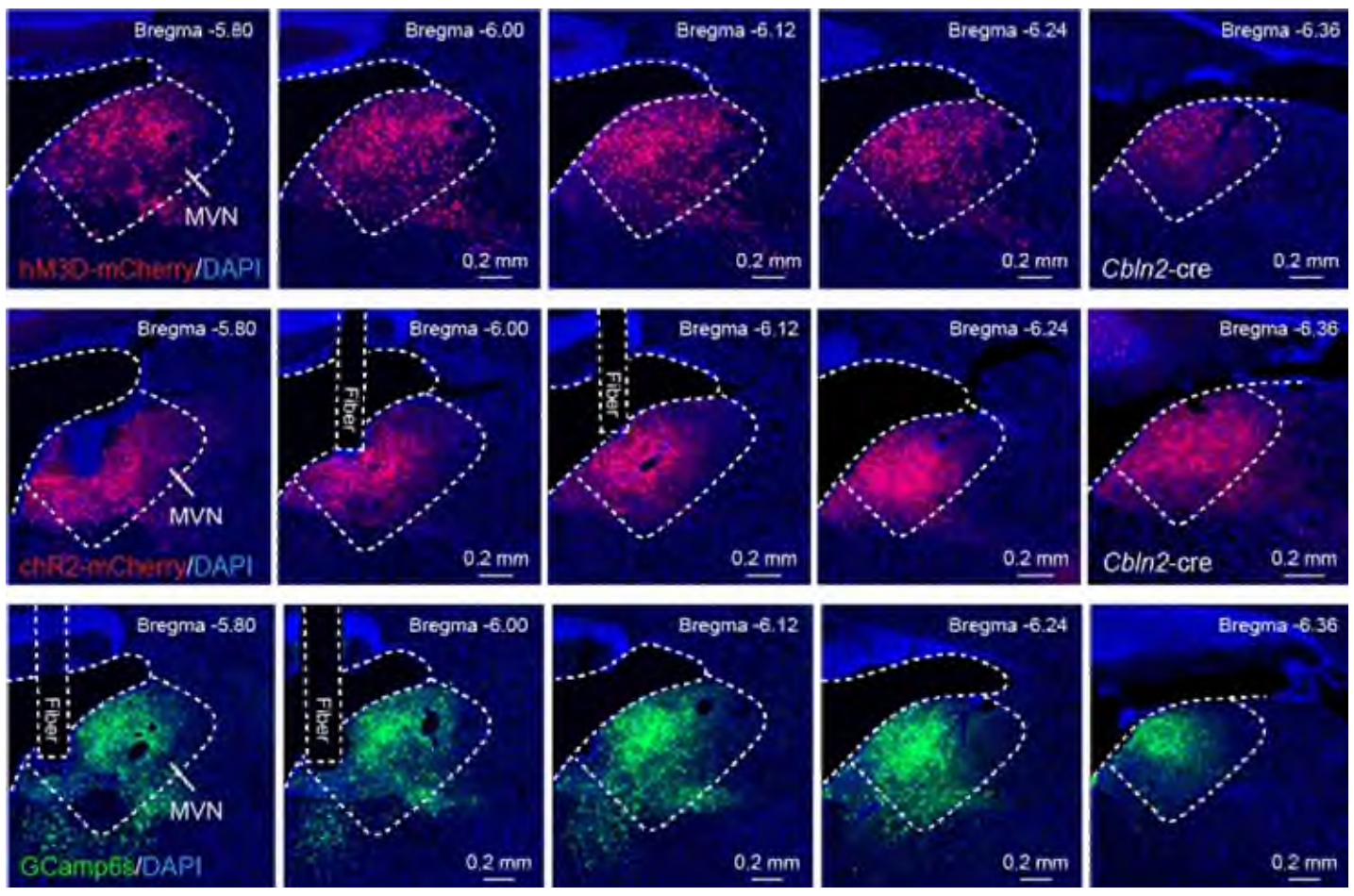

Response Document Figure 12: The expression distribution of Cbln2^{+} neurons along the anterior-posterior axis of the MVN.

Conclusion

This manuscript presents valuable findings regarding the neural circuits underlying vestibular disorientation and its mitigation. Addressing the conceptual concerns about the motion sickness model, completing the mechanistic characterization of the auditory-CeA-MVN pathway, and strengthening the correlative analyses between neural activity and behavior would significantly enhance the impact of this work.

We again thank you for your time, careful review, and expert guidance, which has helped to us to improve the rigor of our work and reinforce the conclusions of our study.

Reviewer #2 (Remarks to the Author):

This manuscript describes how Cbln2+ neurons in the medial vestibular nuclei mediate rotation-induced motion sickness-like behaviors. Anticipatory auditory tones can inhibit motion sickness symptoms, and this is due to central amygdala-to-MVN Cbln2+ inhibitory pathways. The manuscript reports an interesting observation for motion sickness phenotype and charts the underlying circuit, which is highly relevant for understanding motion sickness and nausea. The main conclusions hold and offer novel insight for the field. I have some issues with specific experiments and their related conclusions; hence, these experiments should not be overinterpreted, and the overall conclusions and mechanisms should be adjusted.

Main comments:

1. Major mistake in figures and manuscripts for loss-of-function studies. First of all, Fig 3 &5 loss of function is not chemogenetic inactivation, but apoptotic cell ablation. The manuscript keeps saying "inactivation", demonstrating a lack of understanding of the cell death involved? Second, the method to validate cell ablation is wrong – you would need to use Cbln2-Cre crossed with a reporter like tdTomato, then inject AAV-DIO-taCasp3-GFP vs AAV-DIO-GFP, and compare the number of tdTomato neurons for validating ablation. Just looking at taCasp3-GFP signals tells you nothing about what's going on with the Cbln2+ cells – you see less green cells, which could be that you injected incorrectly, infected fewer. And also, why are there green cells left anyway? They should be dead? This applies to all loss-of-function studies in Fig3, Fig 5. So I would not overinterpret conclusions drawn from this approach. Unless these experiments are redone with proper controls, whether these neurons are required remains unaddressed.

RESPONSE: We would like to thank you for your professional insights and for pointing out critical errors in our work, which have guided several improvements to our study. First, we apologize for our oversight in the inaccurate terminology used in our original manuscript. We have carefully revised the entire manuscript accordingly,

replacing all instances of "inactivation" with "ablation".

As suggested, we completely agree that crossing *Cbln2*-IRES-Cre mice with a reporter like tdTomato would be an ideal approach to validate ablation efficiency. Due to the substantial time required for mouse breeding, we were unfortunately unable to generate these reporter mice within the limited timeframe, which prevented us from conducting the subsequent validation experiments. To address this concern as rigorously as possible under these constraints, we performed NeuN immunostaining to quantify the density of neurons within the targeted region following viral expression. Several prior studies have used NeuN staining to confirm neuronal loss after caspase-based ablation, demonstrating that Cre-dependent taCasp3 expression leads to a marked reduction of NeuN-positive neurons in the targeted population[38-41]. Additionally, to further improve the efficiency and reliability of ablation, we optimized the viral strategy following previously published protocols[38]. We replaced the virus and used a higher-titer (titer: 4.80×10^{12} genome copies per ml).

Specifically, for ablating the $Cbln2^+$ MVN neurons, we bilaterally injected AAV2/9-Flex-taCasp3-TEVp or AAV-DIO-EGFP into the MVN of *Cbln2*-IRES-Cre mice. Two weeks after viral expression, the mice underwent repeated rotation stimulation for 10 consecutive days. Strikingly, we found that mice with apoptosis of $Cbln2^+$ MVN neurons exhibited significant suppression of MS-like behavior, including decreased behavioral evaluation scores, cumulative head-orientation angles, and average velocity, compared with EGFP (non-apoptosis) mice. Importantly, ablation of $Cbln2^+$ MVN neurons also improved appetite compared to the EGFP group on day 10. Representative movement trajectories on day 10 are shown. We further quantified ablation efficiency by counting the number of surviving neurons, and found that ablation efficiency was linearly correlated with cumulative head-orientation angles on day 10 (Response Document Figure 13, and also see new Figure 3). These findings underscore that $Cbln2^+$ MVN neurons were the primary component required for rotation-induced MS-like behavior.

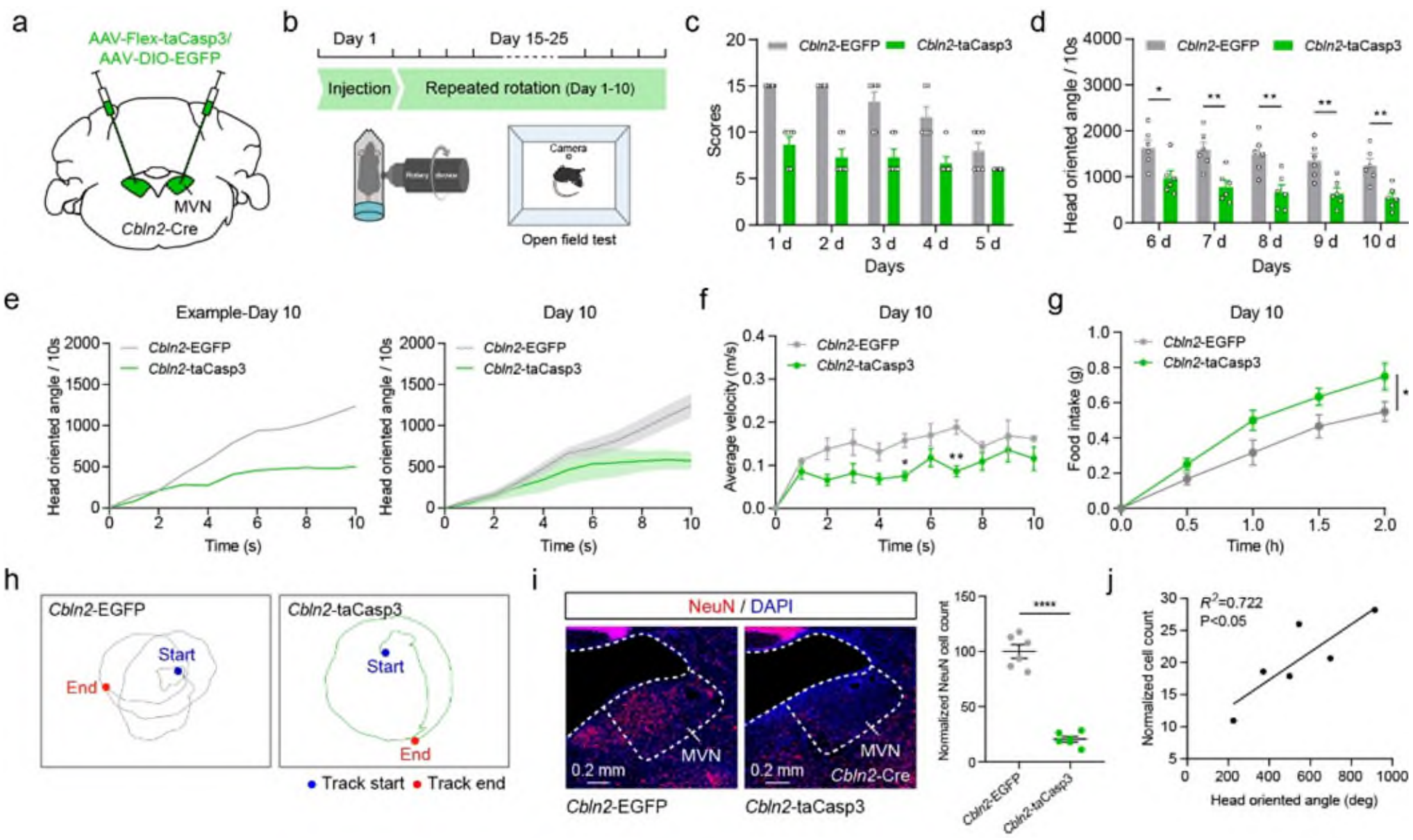

Response Document Figure 13: Cbln2$^+$ MVN neurons are essential for rotation-induced MS-like behavior. a, Viral strategy for targeted ablation of Cbln2$^+$ MVN neurons. **b,** Schematic of the repeated rotation paradigm applied to mice with or without Cbln2$^+$ neuron ablation. **c,d,** Time course of behavioral evaluation scores (c) and cumulative head orientation angles (d) during the first 10 s post-rotation across 10 days (Cbln2-EGFP: n = 6, Cbln2-taCasp3: n = 6). **e-f,** Representative curves of cumulative head orientation angles (e) and average velocity (f) during the first 10 s open-field test following rotation with or without ablation of Cbln2$^+$ neurons in mice on day 10. **g,** Cumulative food intake in hungry mice on day 10 following rotation with or without ablation of Cbln2$^+$ neurons in mice. **h,** Representative curves of movement trajectories during the first 10 s post-rotation on day 10. **i,** Example coronal brain section and summarized for the number of neurons in Cbln2$^+$ MVN neurons in *Cbln2*-IRES-Cre mice. **j,** Scatter plot showing the efficiency of Cbln2$^+$ neurons ablation linearly correlated with cumulative head-orientation angles on day 10. Statistical analyses in (d) and (f) were performed using two-way ANOVA (*P < 0.05, **P < 0.01, ****P < 0.0001). Statistical analyses in (g) and (i) were performed using a two-sided Student's t-test (****P < 0.0001).

For ablating the Cbln2$^+$ MVN-vlPAG pathway, we bilaterally injected AAV2-retro-DIO-flp into the vlPAG and AAV-fDIO-taCasp3-TEVp or AAV-fDIO-EGFP into the MVN of *Cbln2*-IRES-Cre mice. Three weeks after viral expression, the mice underwent repeated rotation stimulation for 10 consecutive days. Similarly, we found that ablation of the Cbln2$^+$ MVN-vlPAG pathway largely reduced behavioral evaluation scores, cumulative head-orientation angles, and average velocity relative to non-ablated controls. However, ablation of this circuit had no significant effect on appetite compared to the EGFP group on day 10. Representative movement trajectories of both groups following rotation on day 10 are shown. Also, the efficiency of this pathway

ablation was quantified by summarizing the number of surviving neurons, and found that ablation efficiency was linearly correlation with cumulative head-orientation angles on day 10 (Response Document Figure 14, and also see new Figure 5). These results imply that the Cbln2^{+} MVN-vlPAG pathway is sufficient and required for rotation-induced MS-like behavior.

We have replaced the original results with these new data in the revised manuscript, including the relationship between ablation efficiency and behavioral effects. We agree that direct quantification of Cbln2^{+} neuron ablation would provide stronger evidence, and we have acknowledged this limitation in the revised manuscript. We are currently pursuing breeding of the reporter line for future studies to rigorously validate ablation efficiency.

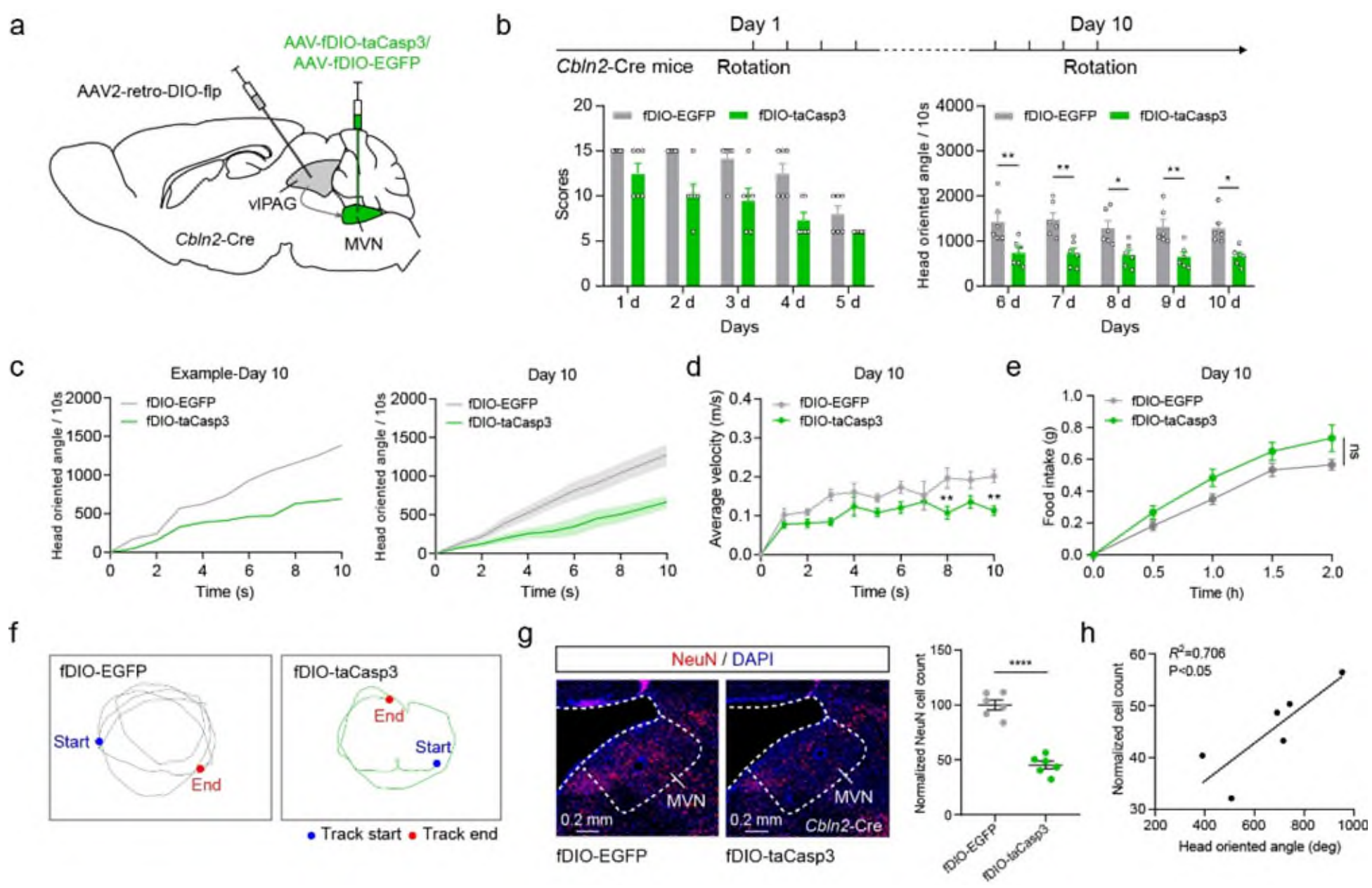

Response Document Figure 14: Cbln2^{+} MVN-vlPAG pathway is critical for MS-like behavior. **a,** Viral injection strategy to express taCasp3 or EGFP in vlPAG-projecting Cbln2^{+} MVN neurons in *Cbln2*-IRES-Cre mice. **b,** Time course of behavioral evaluation scores (left) and cumulative head orientation angles (right) in mice during the first 10 s post-rotation across 10 days in control (EGFP) and ablated (taCasp3) mice (n = 6 per group). **c-d,** Representative curves of cumulative head orientation angles (c) and average velocity (d) during the first 10 s open-field test following rotation with or without ablation of Cbln2^{+} MVN-vlPAG neurons in mice on day 10. **e,** Cumulative food intake in hungry mice on day 10 following rotation with or without ablation of Cbln2^{+} MVN-vlPAG neurons in mice. **f,** Representative curves of movement trajectories during the first 10 s post-rotation on day 10 in control (EGFP) and ablated (taCasp3) mice. **g,** Example coronal brain section and summarized for the number of neurons in vlPAG-projecting Cbln2^{+} MVN neurons in *Cbln2*-IRES-Cre mice. **h,** Scatter plot showing the efficiency of Cbln2^{+} MVN-vlPAG pathway ablation linearly

correlated with cumulative head-orientation angles on day 10. Statistical analyses in (b) and (d) were performed using two-way ANOVA (*$P < 0.05$, **$P < 0.01$). Statistical analyses in (e) and (g) were performed using a two-sided Student's t-test (****$P < 0.0001$).

2. MVN-NTS pathway for nausea. Do you stimulate this pathway optogenetically and look at MS-like rotational behaviors? And do you stimulate MVN-vlPAG and look at CTA behaviors? Terminal stimulation can activate terminals that propagate to cell bodies and activate the whole circuit wherever the cell bodies project to, not restricted to your stimulation site. Unless these site-specific projections are two distinct populations of Cbln2+ neurons, which the authors don't know, I don't think the current methods can suggest that nausea is mediated by MVN-NTS only. In addition, can you do the loss-of-function of Cbln2+ and the CFA test? How important is this pathway to motion nausea vs rotational phenotypes, compared with the MVN CCK+ pathway (which mediates nausea)?

RESPONSE: We appreciate your meticulous review and for providing us with such an important hint. Specific questions from this comment are addressed individually below:

Q1: Do you stimulate this pathway optogenetically and look at MS-like rotational behaviors?

RESPONSE: Thank you for your important comment. As suggested, we have supplemented new data in which we activate the $Cbln2^{+}$ MVN-NTS pathway. Our results showed that optogenetic activation of this pathway did not induce any overt MS-like rotational behaviors, as assessed by behavioral evaluation scores and cumulative head-orientation angles, compared to the $Cbln2^{+}$ MVN-vlPAG pathway. We have updated these results in the revised manuscript (Response Document Figure 15, and also see new Extended Data Figure 8c).

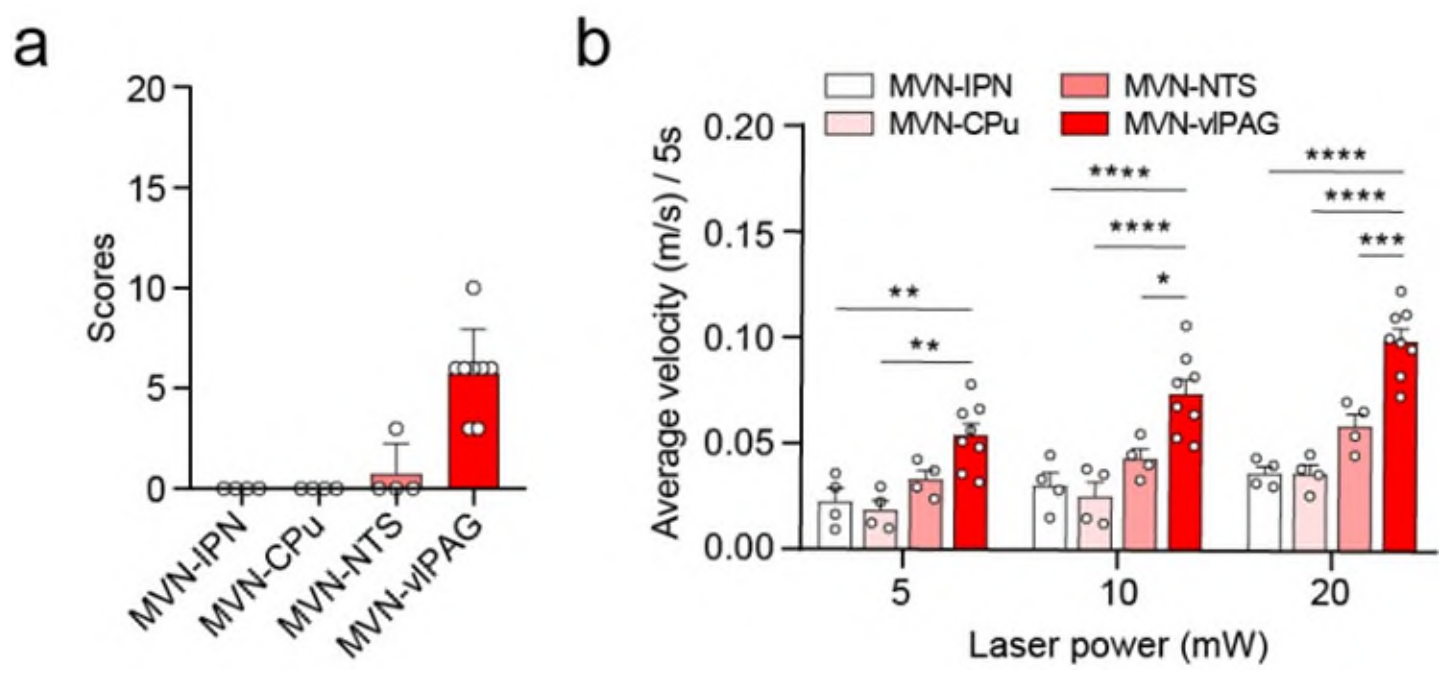

Response Document Figure 15: Comparison of MS-like behaviors evoked by optogenetic activation of distinct Cbln2$^+$ MVN downstream pathways. a,b, Behavioral evaluation scores (a) and cumulative head orientation angles (b) during 5 s optogenetic activation of Cbln2$^+$ MVN projections to IPN, CPu, NTS, and vlPAG (MVN-IPN, MVN-CPu, and MVN-NTS: *n* = 4 mice, MVN-vlPAG: *n* = 8 mice; two-way ANOVA, *P < 0.05, ***P < 0.001, ****P < 0.0001).

Q2: And do you stimulate MVN-vlPAG and look at CTA behaviors?

RESPONSE: As suggested, we have conducted new experiments in which we activate the Cbln2$^+$ MVN-vlPAG pathway in combination with conditioned flavor aversion (CFA) to determine whether this pathway promotes CFA. Specifically, we injected AAV-DIO-ChR2-mCherry into the MVN and implanted optical fibers in the vlPAG of *Cbln2*-IRES-Cre mice. In the memory test phase, mice expressing ChR2-mCherry in the Cbln2$^+$ MVN-vlPAG pathway exhibited conditioned aversion compared to mice expressing mCherry. One possible explanation for this result is that activation of this pathway-induced rotational movement may disturb drinking behavior, potentially confounding the assessment of conditioned aversion (Response Document Figure 16, and also see new Figure 6i). We have incorporated these results into the revised manuscript.

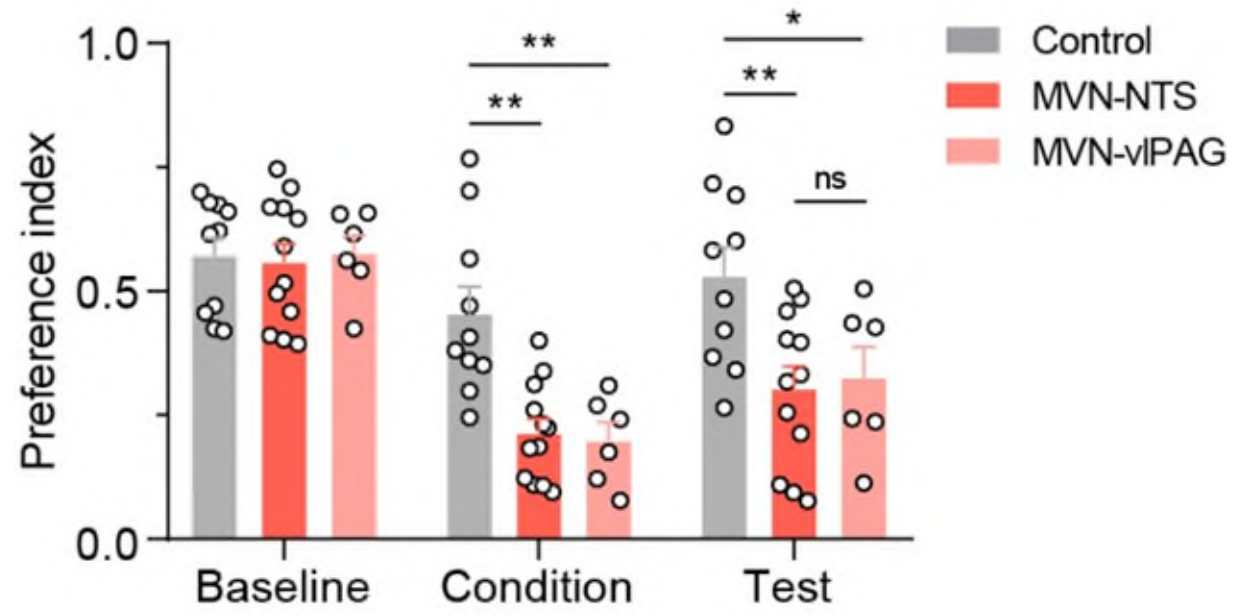

Response Document Figure 16: Quantitative of CFA preference after activation of MVN-NTS versus MVN-vlPAG pathways (Control: *n* = 10 mice, MVN-NTS: *n* = 12 mice, MVN-vlPAG: *n* = 6 mice).

Q3: Terminal stimulation can activate terminals that propagate to cell bodies and activate the whole circuit wherever the cell bodies project to, not restricted to your stimulation site. Unless these site-specific projections are two distinct populations of Cbln2+ neurons, which the authors don't know, I don't think the current methods can suggest that nausea is mediated by MVN-NTS only.

RESPONSE: Thank you for your important and thoughtful comment. We fully agree that, in the absence of projection-specific genetic tools, terminal stimulation cannot definitively rule out co-activation of collateral branches projecting to other brain regions. To address this concern, we applied retrograde tracing by injecting AAV2/2Retro-DIO-H2B-mCherry and AAV2/2Retro-DIO-H2B-EGFP into the vlPAG and NTS of *Cbln2*-IRES-Cre mice, respectively. Our results showed that retro-labeled Cbln2^{+} neurons were primarily distributed in the ipsilateral MVN, and only 3.2% of these neurons were double-labeled, indicating that the two pathways originate from largely distinct Cbln2^{+} neuronal populations and are unlikely to be co-activated. In addition, loss-of-function data (see response to Q4 and Q5) revealed that ablation of the Cbln2^{+} MVN-NTS pathway did not affect rotation-induced circling behavior, whereas ablation of the Cbln2^{+} MVN-vlPAG pathway did not alter rotation-induced suppression of food intake (nausea-related). These anatomical and functional results provide evidence that the observed effects on nausea-related behaviors are mediated predominantly by the Cbln2^{+} MVN-NTS pathway (Response Document Figure 17, and also see new Figure 6j). We have incorporated these results into the revised manuscript.

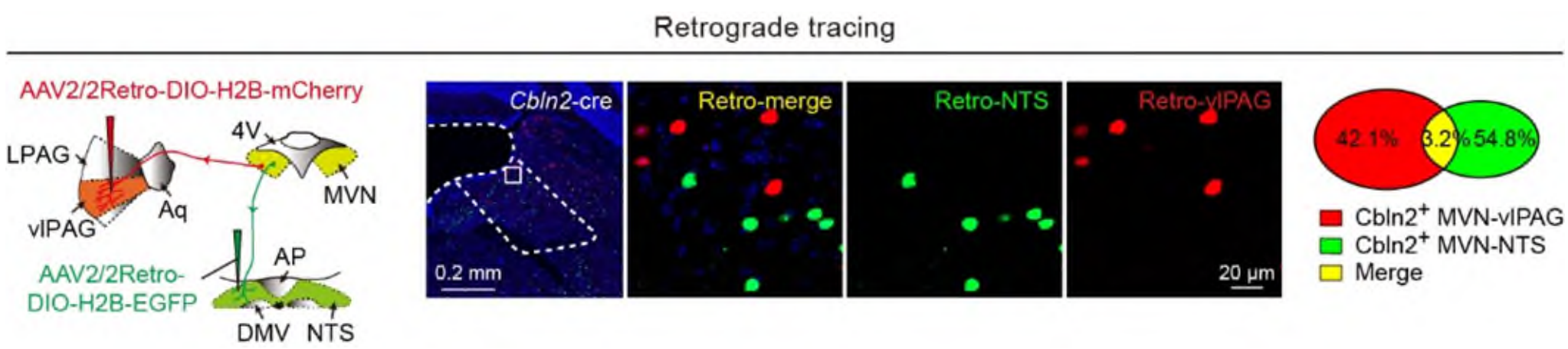

Response Document Figure 17: Dual retrograde tracing of Cbln2^{+} MVN neurons projecting to the NTS and vlPAG.

Q4: In addition, can you do the loss-of-function of Cbln2+ and the CFA test?

RESPONSE: As suggested, we have conducted new experiments to ablate Cbln2^{+}

MVN neurons or the MVN-vlPAG pathway. We agree that CFA is a useful proxy in rodent nausea research. However, because CFA is an associative learning-based paradigm and its outcome can be influenced by processes beyond nausea per se, its implementation and interpretation are less straightforward in our chronic ablation design combined with repeated rotational stimulation. Therefore, we used food intake as a complementary nausea-/malaise-related behavioral readout in this set of experiments. Motion-induced appetite suppression has been used as one behavioral readout of MS-like malaise/nausea-related in rodents[44,45]. Specifically, the mice underwent repeated rotation stimulation for 10 consecutive days following ablation of Cbln2$^+$ MVN neurons or the MVN-vlPAG pathway. On day 1, mice with ablation of Cbln2$^+$ MVN neurons or the MVN-vlPAG pathway showed appetite comparable to that of EGFP (non-ablation) group. Notably, by day 10, mice with ablation of Cbln2$^+$ MVN neurons exhibited improved appetite relative to the EGFP group. However, mice with ablation of the MVN-vlPAG pathway did not differ from the non-ablation group in appetite. Together, these results suggest that the Cbln2$^+$ MVN neurons contribute to both nausea-related and rotational behaviors, whereas the Cbln2$^+$ MVN-vlPAG pathway is specifically involved in rotational behaviors, with no detectable effect on appetite (Response Document Figure 18, and also see new Figure 3s and Figure 5m). We have incorporated these results into the revised manuscript.

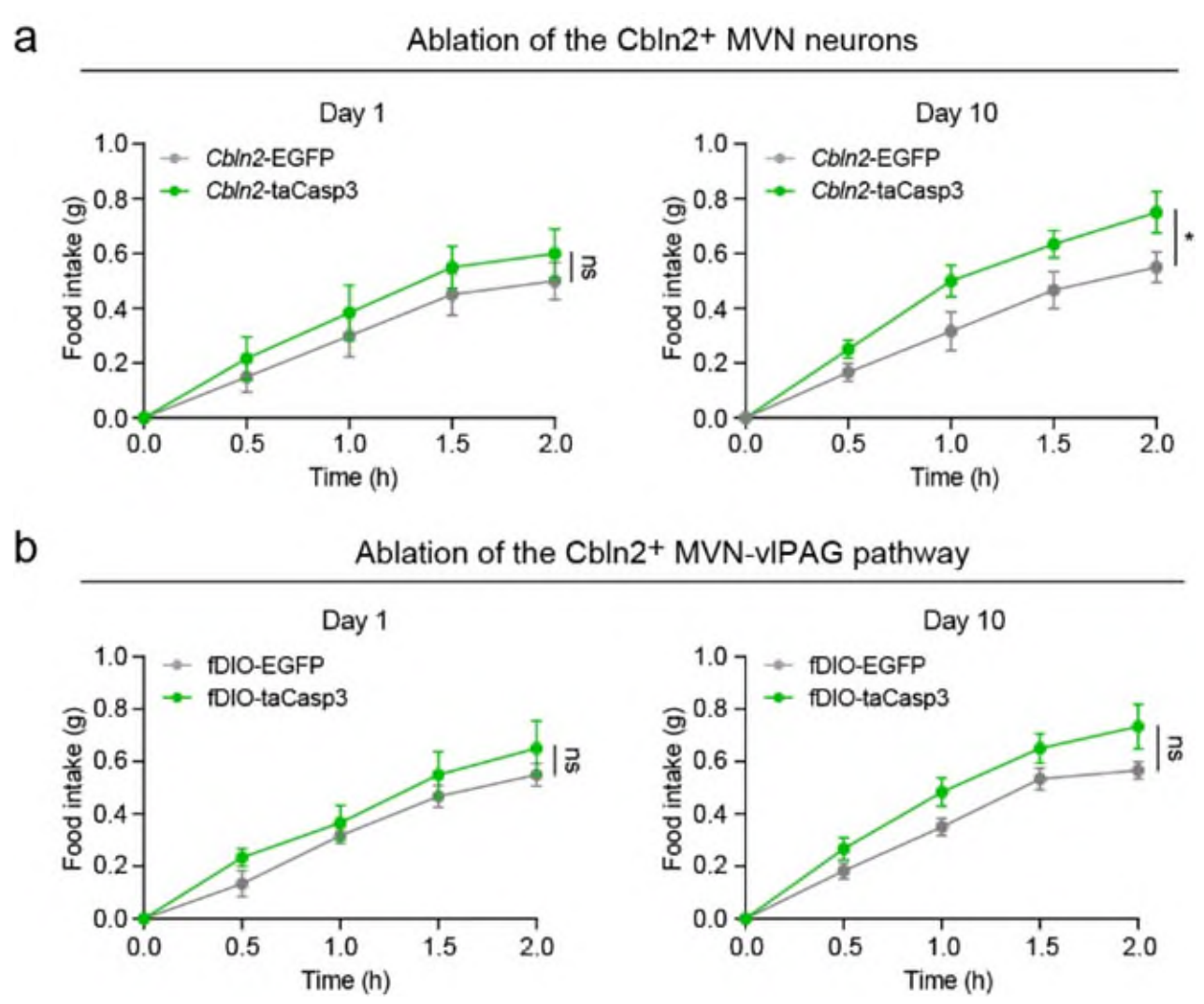

Response Document Figure 18: Effect of ablating Cbln2$^+$ MVN neurons or the MVN-vlPAG pathway on appetite in rotated mice. a,b, Repeated rotation-induced cumulative food intake after Cbln2$^+$ MVN neuron ablation or Cbln2$^+$ MVN-vlPAG pathway ablation on days 1 and 10 (n = 6

mice; two-sided Student's t-test, *P < 0.05).

Q5: How important is this pathway to motion nausea vs rotational phenotypes, compared with the MVN CCK+ pathway (which mediates nausea)?

RESPONSE: Thank you for your constructive comment. To assess the relative importance of the $Cbln2^+$ MVN-NTS pathway in motion nausea-like versus rotational phenotypes, we performed ablation of this pathway. Specifically, we injected AAV2-retro-DIO-flp into the NTS and AAV-fDIO-taCasp3-TEVp into the MVN of *Cbln2*-IRES-Cre mice. Three weeks after viral expression, the mice underwent repeated rotation stimulation for 10 consecutive days. Our results showed that mice with apoptosis of the $Cbln2^+$ MVN-NTS pathway did not differ from the non-ablation group in rotational phenotypes on most days, except for a transient difference on day 7, suggesting that this pathway is not required for motion-induced rotational behaviors. In contrast, on day 10, mice with ablation of this pathway exhibited significantly improved appetite compared to the non-ablation group, indicating a reduction in motion nausea-like responses. Moreover, the extent of $Cbln2^+$ MVN-NTS ablation was linearly correlated with cumulative head-orientation angles on day 10 (Response Document Figure 19). These results imply that the $Cbln2^+$ MVN-NTS pathway contributes preferentially to rotation-induced nausea-like behavior but does not play a primary role in generating rotational phenotypes.

In addition, our previous results suggest that $Cbln2^+$ and CCK^+ neurons likely represent distinct functional subpopulations within the vestibular/MVN circuitry. Their downstream targets also differ. The published CCK^+ VN-PBN branch appears to be important for the aversive/CTA dimension of MS-like behavior. By contrast, our data identify a $Cbln2^+$ MVN-NTS branch that preferentially regulates rotation-induced hypophagia, a nausea-related behavioral readout, while largely sparing rotational behavior. Thus, we suggest that both pathways contribute to MS-related nausea, but through different cell populations and downstream circuits. We have revised the manuscript to clarify that the $Cbln2^+$ MVN-NTS pathway is an important nausea-related branch of the vestibular circuit, but not necessarily the sole pathway mediating

MS-like nausea-related responses.

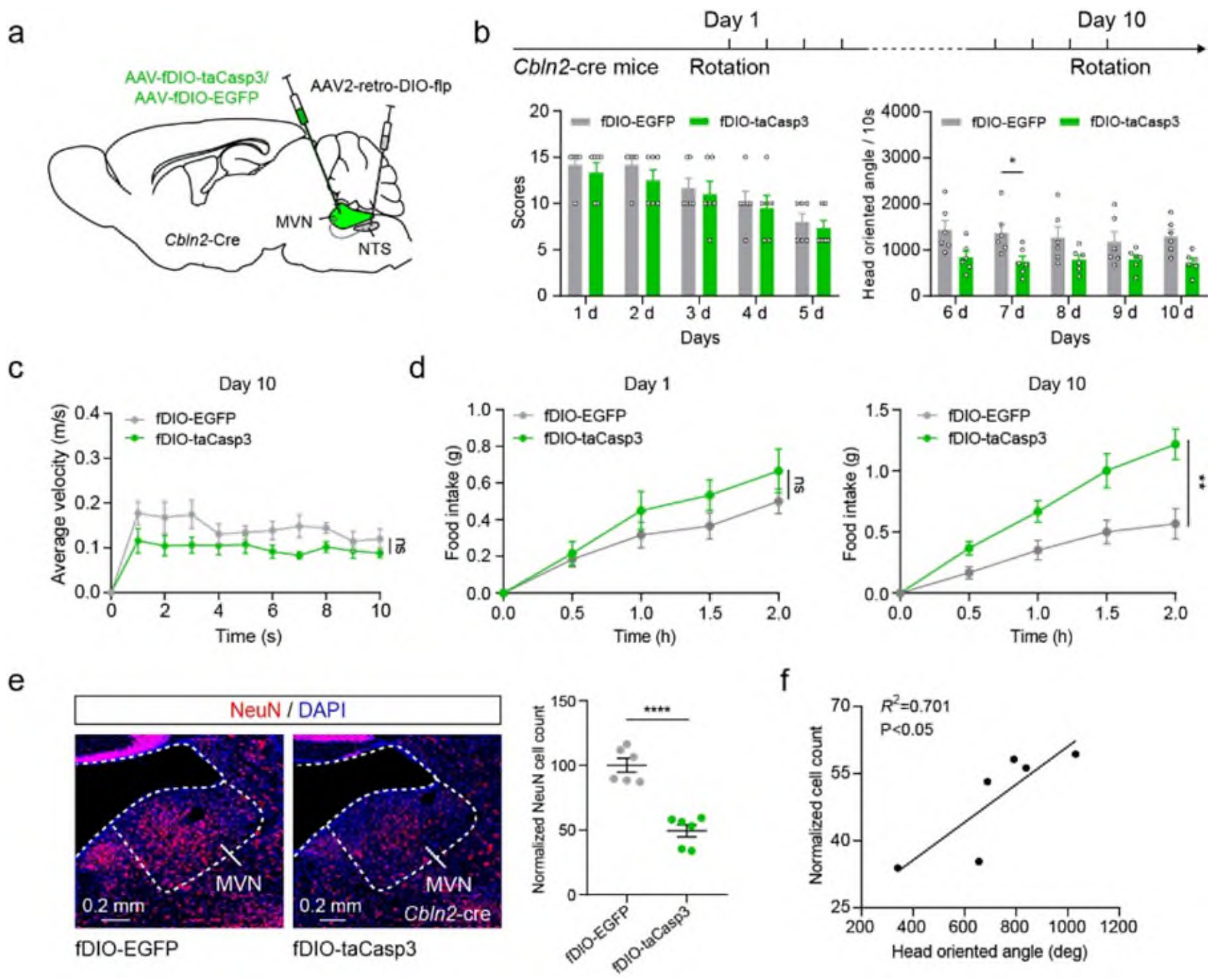

Response Document Figure 19: Cbln2⁺ MVN-NTS pathway is critical for rotation-induced nausea behavior. a, Viral injection strategy to express taCasp3 or EGFP in NTS-projecting Cbln2⁺ MVN neurons in *Cbln2*-IRES-Cre mice. **b,** Time course of behavioral evaluation scores (left) and cumulative head orientation angles (right) in mice during the first 10 s post-rotation across 10 days in control (EGFP) and ablated (taCasp3) mice (n = 6 per group). **c,** Average velocity during the first 10 s open-field test following rotation with or without ablation of Cbln2⁺ MVN-NTS neurons in mice on day 10. **d,** Cumulative food intake in hungry mice following rotation with or without ablation of Cbln2⁺ MVN-NTS neurons in mice on days 1 and 10. **e,** Example coronal brain section and summarized for the number of neurons in NTS-projecting Cbln2⁺ MVN neurons in *Cbln2*-IRES-Cre mice. **f,** Scatter plot showing the efficiency of Cbln2⁺ MVN-NTS pathway ablation linearly correlated with cumulative head-orientation angles on day 10. Statistical analysis in (b) was performed using two-way ANOVA (*P < 0.05). Statistical analyses in (d) and (e) were performed using a two-sided Student's t-test (**P < 0.01, ****P < 0.0001).

3. The calcium imaging data are also intriguing. In Fig 4 h, why with 5g 4 circles the calcium response is not locked with ventral body back flip, unlike the 0.5g, 4 circles? If these neurons receive body and rotational inputs from vestibular, then the neurons should show body-position specific signals, that is, you see 4 peaks during 5g 4 circles instead of just 1 peak. How do you explain?

RESPONSE: Thank you for your rigorous review and insightful comment. We propose the following explanations based on vestibular neurophysiology and the properties of calcium indicators: First, vestibular hair cells and central vestibular neurons have a

limited dynamic range. Under gravity and high-intensity stimulation (e.g., 5 *g* rotation), sensory inputs may approach or reach saturation, reducing the neuron's ability to encode successive body position changes. In this state, neurons may primarily respond to the strong transient onset of motion, generating a single large calcium transient, while subsequent periodic body position signals are compressed or masked. This would result in a single prominent peak rather than four discrete peaks in the calcium trace. Second, under 5 *g* stimulation, neurons may fire at extremely high frequencies or enter a tonic firing mode. Calcium indicators, even fast variants like GCaMP, have limited temporal resolution and integrate calcium influx over time. When spikes occur in rapid succession, the resulting calcium transients can merge into a broad, smoothed peak, obscuring the individual peaks that would correspond to each rotation cycle.

To further investigate this, we have recorded calcium signals under the same 5 *g*, 4-circle condition using GCaMP7f, a variant with faster kinetics and higher sensitivity. In these recordings, we observed four discernible but attenuated peaks corresponding to each rotation cycle (Response Document Figure 20). This suggests that the $Cbln2^{+}$ MVN neurons do receive and encode body-position-specific vestibular inputs even under high-intensity stimulation, but the signals are partially compressed due to the factors described above, and their detection is influenced by the kinetic properties of the calcium indicator.

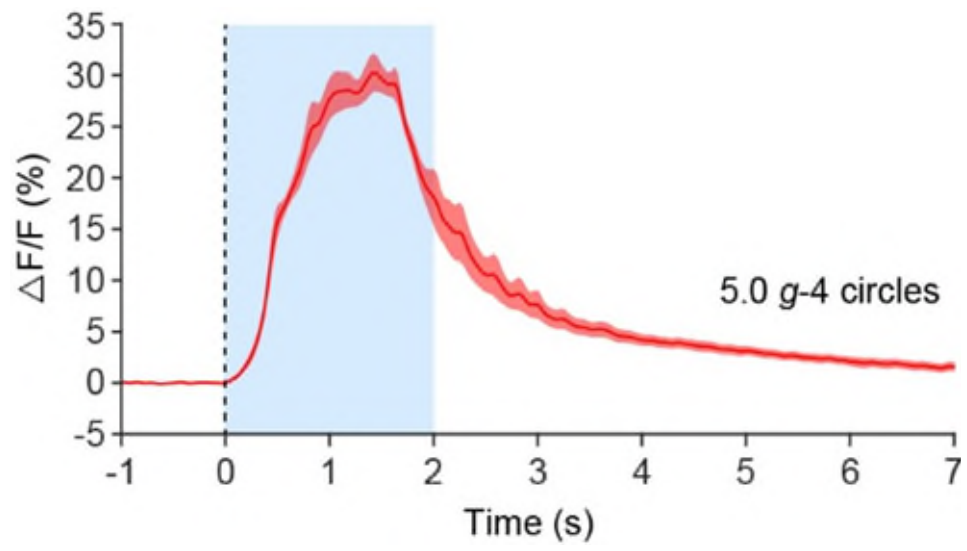

Response Document Figure 20: GCaMP signals recorded from $Cbln2^{+}$ MVN neurons during 5 *g* rotation over 4 circles.

4. In Figure 7, what happens if you rotate animals that are trained to have tone responses during rotation, but do not give them the tone? So you can compare the same animal calcium responses with or without tone during rotation. The statistics and effects within

animals would be even more powerful.

RESPONSE: Thank you for your insightful comment and for the opportunity to clarify our experimental design. Rotation began on day 6 (6 days after viral injection). What were originally labeled as days 17 and 18 in the manuscript actually correspond to days 10 and 11 of repeated rotation, respectively. We have corrected this labeling in the revised manuscript to avoid any misunderstanding. Regarding your specific question about comparing calcium responses with and without tone in the same animals, we actually performed this experiment, and the data were already included in our original results, although the time point labeling may have obscured this point. Here is what we found:

On day 10, both groups of mice (Rotation alone and Rotation+Tone) received tone presentation before rotation onset. We observed that the Rotation+Tone group exhibited significantly lower GCaMP fluorescence intensity in Cbln2$^+$ MVN neurons compared to the Rotation group. In addition, the activity of Cbln2$^+$ neurons was correlated with the severity of MS-like behavior in the Rotation+Tone group. On day 11, when both groups underwent rotation without tone presentation, there was no significant difference in GCaMP fluorescence between the two groups. However, within the Rotation+Tone group, GCaMP signals were significantly higher on day 11 (Non-tone) than day 10 (tone present). These results provide evidence that the anticipatory tone attenuates rotation-induced activation of Cbln2$^+$ MVN neurons, thereby alleviating MS-like behaviors. We have clarified this point in the revised manuscript and updated the figure labels (Response Document Figure 21, also see new Figure 7a-e).

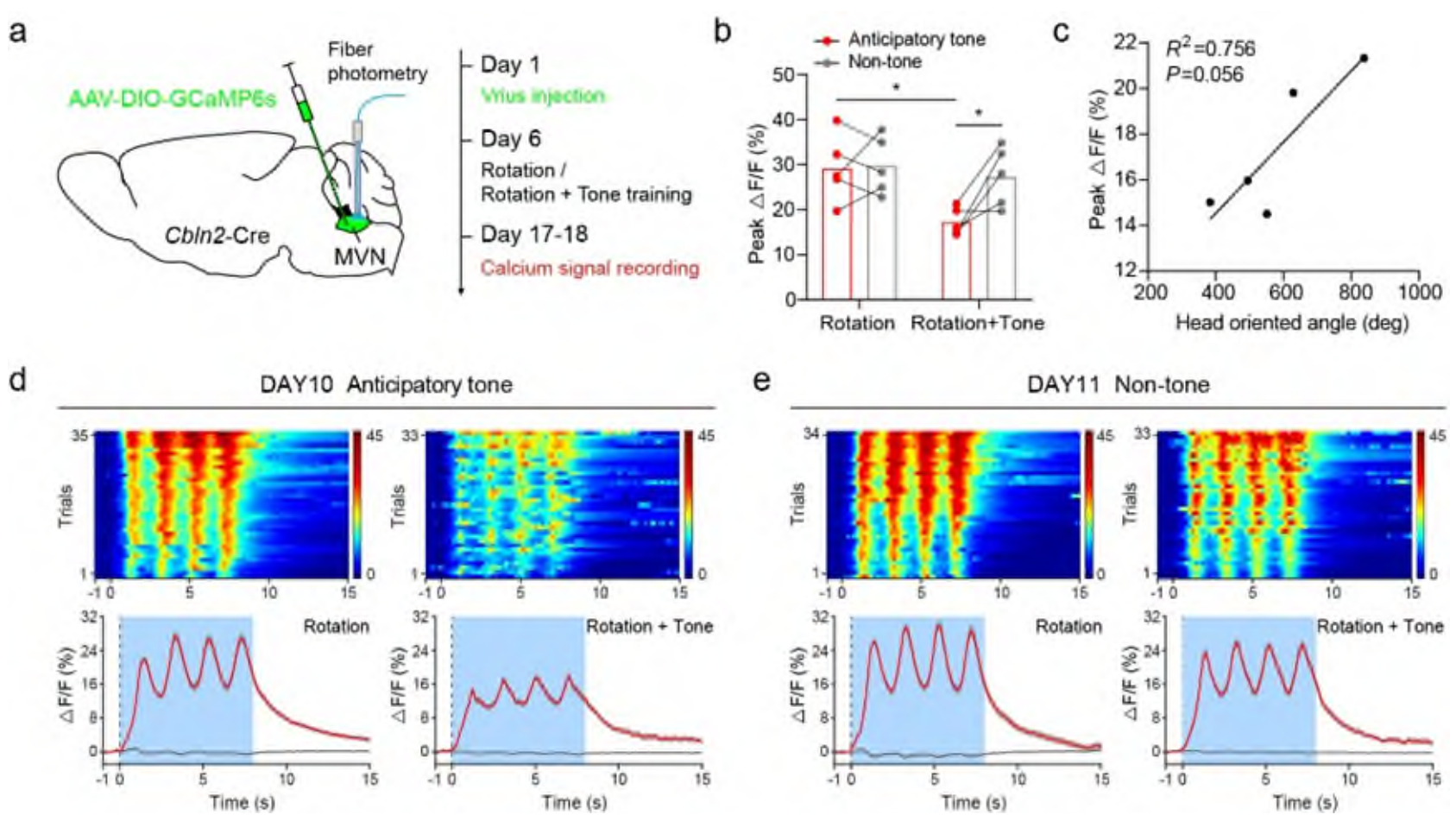

Response Document Figure 21: Anticipatory tone modulates rotation-induced MS-like behavior via MVN Cbln2$^+$ neurons. a, Schematic diagram showing the position of virus injection and optical fiber implantation in *Cbln2*-IRES-Cre mice. **b,** Quantitation of peak GCaMP responses in Cbln2$^+$ MVN neurons during the second rotation cycle with or without anticipatory tone in test (Rotation+Tone) and control (Rotation) mice (n = 5 mice per group; two-way ANOVA, *P < 0.05). **c,** Linear correlation between peak GCaMP responses and cumulative head-orientation angles in the Rotation+Tone group on day 10. **d,** Heatmaps of normalized GCaMP fluorescence changes (ΔF/F) and average GCaMP response curve in Cbln2$^+$ MVN neurons aligned to the onset of rotation with anticipatory tone in test and control mice on day 10. Red lines indicate recordings from GCaMP6s$^+$ cells, gray lines indicate recordings from reference 405 nm. Thick lines represent the mean, and shaded areas indicate s.e.m. Gray dashed line: onset of ventral rotation; Blue shaded areas: duration of ventral rotation. **e,** Same as (d), but aligned to rotation onset on day 11 (no tone presentation).

5. MVN Cbln2+ neurons project to NTS, and rabies tracing shows that they also receive NTS inputs. How to interpret the back-and-forth projections and inputs to and from NTS?

RESPONSE: Thank you for raising this fascinating point. You correctly note that Cbln2$^+$ MVN neurons project to the NTS, while rabies tracing reveals that they also receive inputs from the NTS. This reciprocal organization raises important questions about the functional architecture of this circuit. Physiological studies have shown that NTS neurons receiving gastrointestinal, pulmonary, or baroreceptor inputs are modulated by vertical vestibular stimulation, and that vestibular and visceral information converge in the NTS at the level of single neurons[46-48]. Consistent with this framework, our anatomical and functional results show that Cbln2$^+$ MVN neurons project to the NTS and contribute to rotation-induced nausea-related behaviors. In addition, stimulation of identified MVN neurons projecting to the "gastric" region of the NTS decreases gastric tone[49,50], further supporting the idea that vestibular outputs can directly engage visceral effectors relevant to MS-related malaise. Together, these findings support the interpretation that the Cbln2$^+$ MVN-NTS projection serves as a feedforward vestibulo-visceral pathway through which vestibular signals can influence autonomic and nausea-related functions.

Importantly, our rabies-tracing data also indicate that Cbln2$^+$ MVN neurons receive direct inputs from the NTS. Prior physiological work has shown that emetic or

gastrointestinal stimulation can alter the spontaneous firing and vestibular response gain of neurons in the caudal vestibular nuclei, including the caudal MVN, consistent with a visceral feedback influence on vestibular processing[48,51]. Thus, a plausible interpretation is that the NTS-MVN pathway conveys information about the current internal visceral state, such as nausea, gastrointestinal discomfort, cardiorespiratory status, or autonomic imbalance, back to vestibular circuits, thereby modulating how ongoing motion-related signals are processed. Taken together, the Cbln2^{+} MVN-NTS pathway likely conveys vestibular information to brainstem autonomic/visceral centers, whereas the NTS-MVN pathway may provide visceral-state feedback that shapes vestibular processing during MS. This reciprocal architecture may help coordinate the sensory, autonomic, and behavioral components of rotation-induced malaise. At the same time, we agree that the limits of this conclusion should be defined cautiously. Our current results support reciprocal connectivity between the MVN and NTS, but do not demonstrate that these inputs and outputs necessarily form a cell-by-cell closed loop.

One minor suggestion: Fig 7C and D, you should use the same scale bar for heat map for calcium intensity side by side, so that you can see visually the difference with tone or without.

RESPONSE: As suggested, we have used the same scale bar for the heat map for calcium intensity side by side to facilitate direct comparison (see new Figure 7d,e).

We would like to again thank you for your extremely helpful guidance and supportive comments that have helped to substantially improve our study.

Reviewer #3 (Remarks to the Author):

In this paper, Wan, Cao, Huo et al. use a custom-made experimental setup to induce Motion Sickness (MS) following whole-body rotation in mice and address the neuronal basis of MS behavior and one prominent symptom, namely MS-induced conditioning taste aversion. They also show that tone cues, which allow animals to anticipate rotations, alleviate MS behaviors. To show this, they first describe the experimental setup and the two protocols to induce 1) classic MS behavior or 2) MS behavior following repetitive exposure to rotation. They next assess the induction of MS behavior using several behavioral readouts (mainly scoring of MS behavior and average velocity and cumulative head orientation angles, measured after MS induction in an open arena) as well as physiological measurements (body temperature) and show that anticipatory tone cues alleviate MS behaviors. They then explore the possible neuronal pathways responsible for the observed behaviors. They use c-fos staining to explore brain areas active during whole-body rotations; focus on the Medial Vestibular Nucleus (MVN); use a plethora of techniques in genetically modified mice (fiber photometry for calcium imaging, optogenetic excitation and inhibition, chemo-genetic activation and inactivation, retro- and antero-grade tracing) to establish Cbln2+ excitatory cells as the main cell-type inducing MS behaviors – particularly circling behaviors. The authors then show that the MVN(Cbln2+) to the ventrolateral periaqueductal (vlPAG) pathway is responsible for the rotation-induced MS behaviors and the MVN- parabrachial nucleus (PBN) -solitary tract (NTS) mediates the MS-induced taste aversion. They finally show that the inhibitory central amygdala (CeA) to MVN(Cbln2+) pathway is involved in the anticipatory tone-induced alleviation of MS behavior. This study is interesting and could be valuable to the wider community, if it wasn't for serious issues – most of it presentational, but not only – which makes its conclusions difficult to evaluate. These issues need to be seriously and thoroughly addressed prior to considering the paper for publication.

Major comments:

1. The principal issue with the paper is presentational, but not only. Countless grammatical issues render the reading extremely tedious and in cases makes it very difficult to even understand the experimental design. For instance, “Notably, the rotated mice received fixed tone training, but not random tone training, exhibited significantly reduced cumulative head-orientation angles and average velocity on days 6-10 compared with rotated mice exposed only to ambient noise” (lines 122-125). The reader needs to make an effort to understand what is meant by “random tone training”, as this is not explained in the main manuscript and, frankly, quite badly in the methods section.

RESPONSE: We would like to thank you for your positive review and expert insights, which have guided several improvements to our study. First, thank you for your careful reading and constructive criticism. We sincerely apologize for the frustration caused by the numerous grammatical issues and unclear descriptions in our original manuscript, which made the experimental design difficult to follow. In response to your comments, we have thoroughly revised the entire manuscript to improve its readability and clarity. Regarding the specific example you pointed out (lines 122-125), we have clarified the meaning of "random tone training" both in the Methods section and the corresponding figure legends. Briefly, "random tone training", which served as a control condition for the fixed tone training, refers to a paradigm in which mice were presented with two 3-s tone cues at random, unpredictable intervals during the 40-s rotation period. These tone cues were temporally uncoupled from the rotation onset or offset. This control condition accounts for non-specific auditory exposure, where the tone is present but provides no temporal prediction about rotation.

2. Another example: “Besides, according to the MVN single cell expression profiling and the Allen Brain Atlas mRNA in situ hybridization data, Crh+, Chat+ and Cbln2+ could be the proper candidate excitatory neuronal subtypes” (lines 137-139) – regardless of the grammatical mistakes, it is just very difficult to understand what’s meant here. Are these the only markers of excitatory cells in the MVN? Did they choose

them because they were profiled by the Allen Institute? Why can Cck not be a "proper candidate"?

RESPONSE: We appreciate your meticulous review and for providing us with such an important comment. We identified several candidate marker-defined neuronal populations in the MVN, including Crh^+, $Chat^+$, and $Cbln2^+$ cells. These were not intended to represent the only excitatory markers in the MVN; rather, they were selected as candidate populations for functional screening because they showed detectable/enriched expression in the MVN and were considered potentially relevant to autonomic responses[52-54]. Importantly, we fully agree that Cck^+ neurons should be included as a candidate population, consistent with recent evidence showing that CCK-expressing vestibular nucleus neurons play a key role in motion-induced malaise[9]. We have revised related content in the manuscript to clearly state, as follows:

> *"Guided by previous single-cell expression profiling studies of the MVN[52] and mRNA in situ hybridization data from the Allen Brain Atlas, we identified several marker-defined neuronal populations in the MVN, including Crh^+, $Chat^+$, Cck^+, and $Cbln2^+$ neurons. These populations showed detectable or enriched expression in the MVN and were selected as candidate populations potentially relevant to autonomic responses[53,54]. Cck^+ neurons were also included because recent work evidence that CCK-expressing vestibular neurons are involved in motion-induced malaise[9]. To identify MVN key excitatory neuronal subtypes that mediate rotation-induced MS-like behavior, we optogenetically activated these marker-defined populations in the corresponding Cre mouse lines, using Vglut2-IRES-Cre mice as a positive control for excitatory vestibular neurons. Specifically, we injected AAV-DIO-ChR2-mCherry into the MVN of these five Cre mouse lines and implanted optical fibers above the MVN (Fig. 3a). Allen Brain Atlas in situ hybridization data supported the expression of these marker genes in the MVN (Fig. S4a), and immunostaining confirmed accurate viral targeting within the MVN (Fig. S4b)."*

3. Further down in the Methods section, the authors write: "A mouse was placed in an adequately ventilated 50-mL conical plastic tube coupled to the rotary device, with its head on the axis of rotation (internal radius from mouse head: 5 cm)" (Methods section, line 19). This does not describe correctly the apparatus shown in movie S5 – specifically, the animal's head is not on the axis of rotation, hence an "internal radius from mouse head is 5 cm" (which presumably is from axis of rotation to the head of the animal and, incidentally, does not appear to be 5cm). There are countless examples of these issues.

RESPONSE: As suggested, we have thoroughly revised the entire Methods section to improve its readability and clarity. Regarding the specific example you pointed out (Methods section, line 19), we have revised the Methods section to clarify that the mouse's head was not on the axis of rotation. Instead, the restraint tube was mounted such that the head was positioned at an estimated radial distance of approximately 5 cm from the rotation center. The revised text reads as follows:

"To establish a mouse model of MS-like behavior, we used a custom-made rotational stimulation device. The apparatus consisted of a speed-adjustable rotator and a modified restraint system made from a well-ventilated, visually shielded 50-mL conical plastic tube. Mice were habituated to the restraint tube for 3 consecutive days (3 min per day) before testing to minimize stress-related confounds. On the test day, each mouse was placed into the tube, which was mounted horizontally onto the rotator arm, such that the estimated position of the animal's head was approximately 5.0 ± 0.1 cm from the center of rotation. Mice were allowed to rest in the stationary tube for 3 min before stimulation to adapt to the restraint. Rotation was then initiated at a constant speed of 300 ± 5 rpm, generating a centripetal acceleration of 5.0 ± 0.2 g as calculated by the formula $a = (2\pi n/60)^2 \times r$ *(where n is the rotational speed in rpm and r is the radius in meters). Each rotation stimulus lasted 40 s. In addition, depending on the experimental paradigm, mice were subjected to ventral vertical, dorsal vertical, horizontal rotation. In some experiments, ventral vertical rotation was delivered at different*

intensities (5 g, 2.5 g, or 1.25 g; 5 g, 0.5 g, or 0.05 g). Control mice underwent the same handling and restraint procedures without rotation."

4. The figures and legends are better, but some aspects absolutely need improvement. A rather worrying issue, for instance, is found in Fig. 5e, f. First, mW is laser power, not intensity. Second, the mCherry gray bar at laser power 5mW (panel e) is the same as the mCherry grey bar at 20Hz (panel f). And the ChR2-mCherry red bar at 20mW (panel e) is the same as the ChR2-mCherry red bar at 20Hz (panel f). It therefore seems that the authors are comparing, for the two right-most bars in panel f, control mCherry 5mW/20Hz versus ChR2-mCherry 20mW/20Hz, which is not correct.

RESPONSE: Thanks for your careful review. First, we apologize for our oversight in the inaccurate terminology used in our original manuscript. We have carefully revised the Figure accordingly, replacing "laser intensity" with "laser power". Second, thank you for pointing out this important inconsistency. We apologize for our oversight in the presentation of Figure 5. The data in Figures 5e and 5f were collected from the same batch of mice. Specifically, Figure 5e shows recordings obtained under graded laser power (5-20 mW) while keeping the laser frequency constant at 20 Hz. Figure 5f shows recordings obtained under graded laser frequency (5-20 Hz) while keeping the laser power constant at 20 mW. Therefore, the data point corresponding to 20 Hz in Figure 5f is indeed the same as the data point corresponding to 20 mW in Figure 5e (Response Document Figure 22, and also see new Figure 5e,f). Unfortunately, due to our oversight, the 20 Hz mCherry control data shown in Figure 5f inadvertently used the same 5 mW mCherry control data from Figure 5e, rather than the correct control data obtained under the same conditions.

We have updated Figure 5f with the correct mCherry control data (20 mW, 20 Hz) and revised the figure legend to explicitly state the stimulation parameters for each panel.

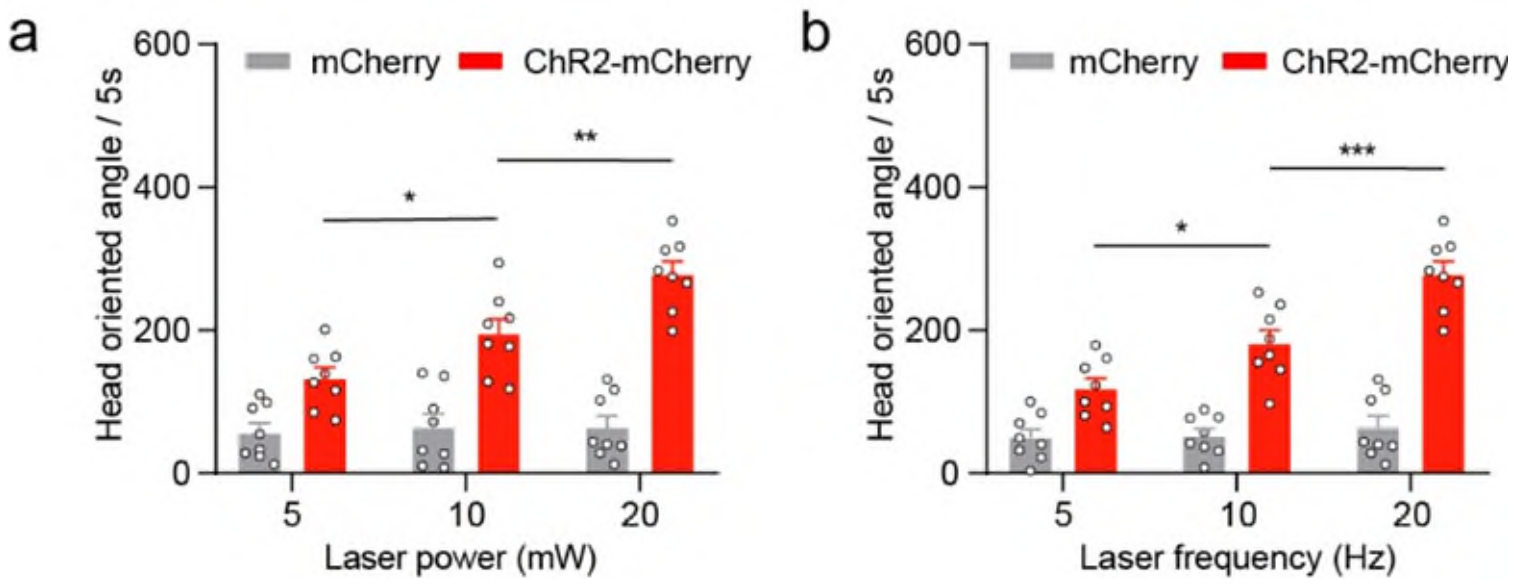

Response Document Figure 22: Quantitative analysis of the cumulative head orientation angles of mice following optogenetic activation of the MVN-vlPAG pathway. a, Cumulative head orientation angles as a function of graded laser power (5-20 mW) at 20 Hz. **b,** Cumulative head orientation angles as a function of graded laser frequency (5-20 Hz) at 20 mW.

5. From the experimental design point of view, the biggest caveat of this paper is the unilateral targeting of the MVN (figure 3a-l) and vlPAG projections (figure a-h) for optogenetic/chemo-genetic activation of Cbln2+ neurons. It is impossible to tell if the relevant behavioral readouts, i.e., average velocity and in particular cumulative head-orientation angles and circling behavior, are not due to the unilateral activation of the MVN. This substantial caveat is somehow attenuated by the experiments showing that the bilateral chemo-genetic inactivation of Cbln2+ neurons reduce MS behavior – although the chemo-genetic inactivation of the MVN-vlPAG pathway is also performed unilaterally (figure 5i-n). The authors should provide at least more evidence to claim that MVN Cbln2+ neurons projecting to the vlPAG mediate MS behaviors (Figures 3 and 5). One line of action is to show that the repetitive rotation procedure elicits clockwise and counterclockwise circling behavior and cumulative head-orientation angles in equal proportions. Then, the authors should show that the unilateral activation of MVN Cbln2+ neurons projecting to the vlPAG (figure 3 and 5) results in both CW and CCW circling behaviors (and cumulative head-orientation angles) in equal proportions and independently of the side where the MVN is activated. Ideally, the authors should perform control experiments where MVN Cbln2+ neurons projecting to the vlPAG are bilaterally targeted and show that the MS behavior is similar to experiments where the neurons were unilaterally targeted.

RESPONSE: We are grateful for your excellent comment and for bringing it to our attention. First, we would like to clarify that both the $Cbln2^{+}$ MVN neuron ablation and

the MVN-vlPAG pathway ablation experiments were performed bilaterally, not unilaterally. Your concern mainly applies to our unilateral activation experiments. In our hands, unilateral optogenetic activation of Cbln2^{+} neurons or the MVN-vlPAG pathway reliably evoked clockwise circling behavior, not counterclockwise. We agree that this raises the important question of whether the observed behavior reflects a direct motor command or, instead, a motor manifestation of vestibular imbalance associated with MS-like states. Prior studies have shown that unilateral activation of vestibular glutamatergic neurons is sufficient to induce immediate postural imbalance, directional turning/spinning, and impaired motor coordination[55,56]. In parallel, activation of vestibular CCK^{+} neurons can induce several MS-like responses[9], indicating that vestibular neuronal activation can simultaneously engage malaise-related and motor/postural outputs. We therefore favor a cautious interpretation: unilateral activation of Cbln2^{+} MVN neurons or the MVN-vlPAG pathway likely produces a vestibular-imbalance-associated MS-related behavior, in which circling is generated, at least in part, as a reflexive or compensatory response to dizziness, imbalance, or a disturbed estimate of body orientation[57], rather than simply reflecting control of one isolated motor action[58,59]. At the same time, a more direct contribution to motor output remains possible, and our current data do not distinguish between these possibilities.

To further examine the directional sensitivity of Cbln2^{+} neurons, we conducted an additional rotation experiment in *Cbln2*-Cre mice. We unilaterally injected Cre-dependent GCaMP6s virus into the MVN and implanted an optical fiber above the site. Mice were placed in a restraint tube in the normal prone position and then subjected to 0.5 *g*, 90° rotation toward either the ipsilateral or contralateral side relative to the optical fiber site. We observed that ipsilateral rotation evoked significantly stronger GCaMP signals compared to contralateral rotation (Response Document Figure 23). This result suggests that unilateral activation of MVN neurons may preferentially enhance vestibular output on the stimulated side and, via the predominantly inhibitory commissural interactions between the bilateral vestibular nuclei, reduce activity on the contralateral side. The resulting vestibular imbalance could in turn bias body orientation

and locomotor output toward one direction, leading to directional circling. However, this interpretation remains tentative, as our current data do not directly measure contralateral suppression.

Importantly, when we performed bilateral activation of Cbln2$^+$ neurons or the MVN-vlPAG pathway, mice exhibited pronounced retropulsion/backward movement rather than clockwise circling. This phenotype closely resembles the severe retropulsion observed during the early phase of repeated rotation stimulation. We therefore speculate that bilateral activation of this pathway leads to a more severe manifestation of MS-like imbalance, overwhelming the system's ability to generate coordinated directional turning and instead resulting in retropulsion. We have incorporated the related content into the revised discussion section.

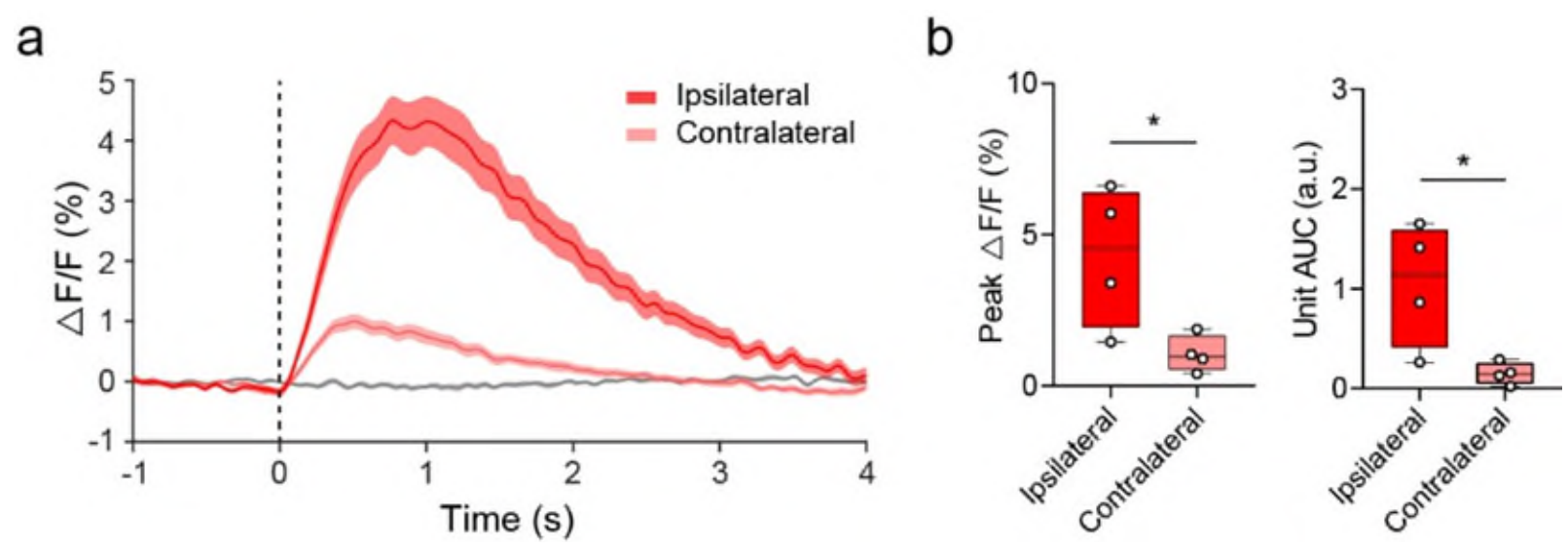

Response Document Figure 23: GCaMP responses of ipsilateral or contralateral rotation-evoked MS-like behavior in Cbln2$^+$ MVN neurons. a, Mean GCaMP signal after ipsilateral or contralateral rotation in Cbln2$^+$ MVN neurons. **b,** Quantitation of peak amplitude (left) and unit AUC of GCaMP responses (right) in Cbln2$^+$ MVN neurons after ipsilateral or contralateral rotation (n = 4 mice; two-sided Student's t-test, *$P < 0.05$).

Minor comments:

A few other (less critical) examples of grammatical issues: "Auditory cues generate temporal expectations, predicting when the next step (?) should occur (?)" (line 47), "in addition, MVN neurons coordinate (?) with multisensory integration (?) nuclei such as …" (line 60), "mice in the rotation group exhibited retropulsion or circling symptoms compared to controls after repetitive provocation (?) on day 5" (line 100), "We then performed 10 days of repetitive rotation matched with tone cues in mice to intervene (?) MS-like behavior". "We found that optogenetic activation of the Cbln2+ neurons induced robust MS-like symptoms compared with the other two neurons (?), with a

score of 9 and cumulative head-orientation angles of 416 ± 31 during 5 s of stimulation" (line 144). "At the onset of the ventral rotation stimulus, clear transient increases in GCaMP fluorescence were observed in the Cbln2+ MVN-vlPAG pathway, whereas fluorescence in the EGFP-expressing did not alter (?)" (line 272), "This anatomical segregation suggests that these two neuronal populations may subserve disparate (?) behavioral outputs" (line 479), etc., etc., etc.

RESPONSE: Thanks for your careful review. We have corrected grammatical errors and awkward phrasing throughout the manuscript to improve its readability and clarity.

The authors write: "Therefore, we developed a custom-made rotational paradigm to assess rotation-induced MS-like behavior in mice evoked by 5 g vertical rotation stimulation for 40 s (Fig. 1a and Fig. S1a)" (line 88). Yet the legends of Fig. S1a read: "a Cumulative head orientation angle in a 10-s open field test following 5 g vertical or horizontal rotation for 30 s in mice (Vertical group: n = 4 mice, Horizontal group: n = 5 mice)." So, is it 40 seconds, or 30? If it is 30 seconds (and not a typo), why did the authors change the duration of rotation for the experiments shown in Fig. S1a?

RESPONSE: We apologize for the confusion caused by our ambiguous description. In Fig. S1a, the rotation duration was 30 s, not 40 s. This is not a typo, but part of our preliminary experiments. In these pilot experiments, we tested both 5 *g* ventral vertical rotation and 5 *g* horizontal rotation for 30 s and 40 s. Under the 40 s condition, mice subjected to ventral vertical rotation consistently showed immediate tumbling and marked disorientation after stimulation, whereas only a subset of mice subjected to horizontal rotation exhibited tumbling and disorientation. By contrast, under the 30 s condition, mice reliably exhibited rotation behavior, which could be quantified by the cumulative head-orientation angle. We observed that ventral vertical rotation for 30 s produced a significantly greater cumulative head-orientation angle than horizontal rotation for 30 s, thereby more clearly demonstrating the specific effect of ventral vertical rotation in inducing MS-like behavior. However, to maintain consistency with the main experimental paradigm used throughout the study, we removed the 30 s data

from Fig. S1a and instead present the corresponding 40 s data in Fig. 1c for both ventral vertical and horizontal rotation. We have also added the 40 s dorsal vertical rotation data (see response to the comment after next) (Response Document Figure 24, and also see new Figure 1c).

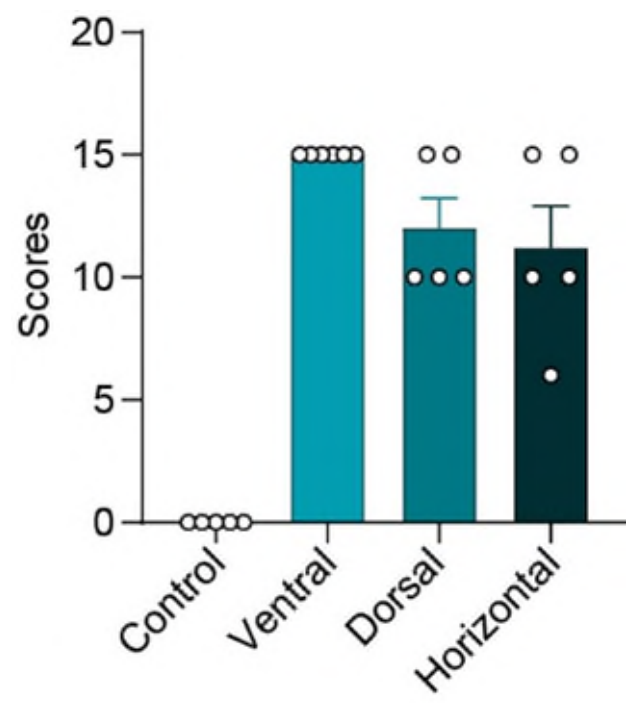

Response Document Figure 24: Behavioral evaluation scores during the first 10 s following 5 *g* ventral/vertical, dorsal/vertical, and horizontal rotation for 40 s in mice.

Also, Fig. S1a shows horizontal rotation, but the authors do not explain why they want to test horizontal rotation and nowhere is it mentioned why they decide to keep vertical rotation as their main paradigm. And then, horizontal rotations resurface in Fig S7a-c for no apparent reason. The authors need to explain in the main manuscript why they choose vertical rotation as their protocol to induce MS behavior and once they've made that choice, horizontal rotations should be dropped. If, on the other hand, they absolutely need to show that Cck neuronal activity is also lower compared to Cbln2+ neurons after horizontal rotations, they should justify why. They have to explain that Machuca-Marquez et al. have shown that Cck+ neurons are responsible for MS behaviors, that Machuca-Marquez et al. experimental design is a combination of horizontal and vertical forces, and that, therefore, Cck neuronal responsiveness should be tested for both vertical and horizontal rotations. This needs to be spelt out clearly.

RESPONSE: Thank you for your valuable comment. We have revised the manuscript to add these points as follows: As reported by Machuca-Marquez et al., Cck^+ neurons are involved in motion-induced malaise. Importantly, their experimental design included both horizontal and vertical force components during rotation. To build upon this work, we separately tested vertical and horizontal rotation paradigms. The vertical

rotation paradigm was further subdivided into ventral vertical and dorsal vertical rotation, and we examined the responsiveness of both Cbln2^{+} and Cck^{+} neurons under each condition. We have updated the manuscript to clearly explain these points.

Similarly, the reader suddenly realizes that there are ventral and back-facing rotations when the authors write: "Moreover, quantitative analyses indicated that GCaMP amplitude of the ventral rotation stimulus was higher than the back-facing rotation stimulus, without significant differences in the unit area under the curve (AUC) (Fig. S6b, c)." (line 204). Why have the authors chosen ventral rotations as against back-facing rotations? This should be presented at the beginning of the manuscript, showing that vertical and ventral rotations are the most robust protocol to induce MS behavior, compared to horizontal or back-facing rotations. Once that's established, there is no reason to introduce a new protocol as an afterthought.

RESPONSE: As suggested, we have supplemented new data using a 5 *g* dorsal rotation for 40 s. Specifically, mice were subjected to ventral vertical, dorsal vertical, or horizontal rotation at 5 g for 40 s, whereas control mice underwent the same handling procedure without rotation. MS-like symptoms were then assessed using a behavioral evaluation score during the subsequent open-field test. Mice exhibited transient MS-like symptoms to varying degrees immediately after rotation, with ventral vertical rotation inducing the most overt tumbling symptoms, confirming that ventral vertical rotation is the most effective protocol for inducing robust MS-like behavior (Response Document Figure 25, and also see new Figure 1c). This added data has been incorporated into the revised manuscript.

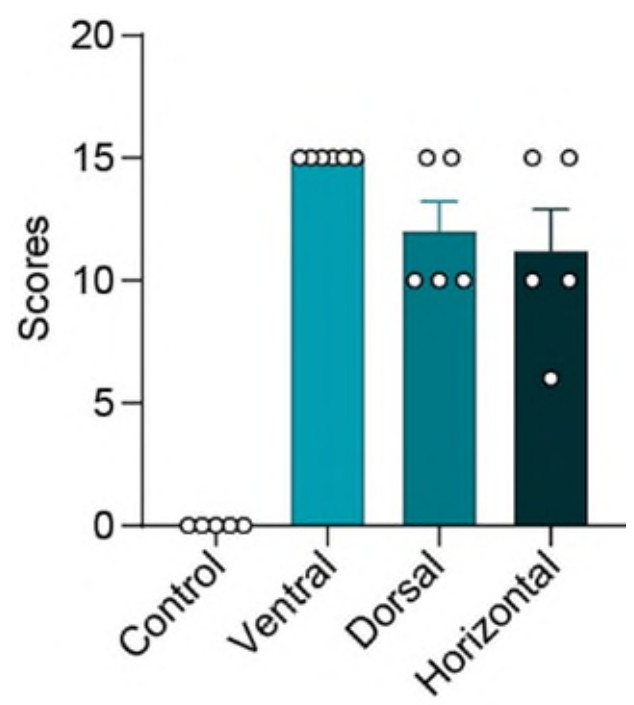

Response Document Figure 25: Behavioral evaluation scores during the first 10 s following 5

***g* ventral/vertical, dorsal/vertical, and horizontal rotation for 40 s in mice**.

The authors can't claim that chemo-genetic inactivation of Cbln2+ neurons "eliminate" MS behavior (Figures 3 and 8 and in several instances in their manuscript) unless they show it – that is, they show that the animals' behavior is statistically not different from controls (animals that were placed in the chamber, but were not rotated).

RESPONSE: As suggested, we have carefully revised the manuscript and figures accordingly, replacing all instances of "eliminate" with more accurate terms such as "attenuate," "alleviate," or "suppress".

The activation of Cck+ neurons appear in line 157 of the manuscript without any justification. Previous results (notably Machuca-Marquez et al.) should be discussed in the introduction, so that the readers understand why suddenly the authors are interested in testing the responsiveness of Cck+ neurons. Another possibility would be to add, after the authors have justified their choice of neuronal types using the Allen Institute in situ hybridization data, that another "proper candidate" is Cck+ neurons. This would mean adding this neuronal cell type to the narrative from the beginning (i.e., from figure 3a onwards) and not as an afterthought.

RESPONSE: We are grateful for your insightful feedback. We have revised the Introduction to include a discussion of previous findings, particularly the work by Machuca-Marquez et al. Additionally, in the Results section, we have explicitly introduced Cck^{+} neurons as another appropriate candidate for investigation.

The legends of Figures 1, 2, S3 stipulate that the average velocity is calculated during a 10-min open-field test. Yet, this is not the case in figures 3, 5, 8, S1, S5, S9, S12 and S13 – what time period was used to calculate the average velocity presented in these figures? Regardless of the time-window used to calculate the average velocity, this should be detailed in the methods section.

RESPONSE: Thank you for your careful observation. For Figures 3, 5, 8, S1, S5, S9, S12, and S13, the average velocity was calculated during a 10 s open-field test

following rotation stimulation. We have updated the corresponding figure legends in the revised manuscript.

The authors claim that "Mice were fed with ad libitum standard chow and water, unless food-deprived overnight for 16-18 h for fasted experiments." (Methods section, line 10). Yet, in line 24 of Methods, they write: "For the food intake test, animals were individually acclimated to the cage for 3 days prior to stimulation, then fasted with ad libitum access to water for 24 h at the onset of the dark cycle." So, animals were food deprived for 16-18h or 24h?

RESPONSE: Thank you for identifying this inconsistency in the Methods section. In all fasting experiments reported in this study, mice were food-deprived for 16-18 h before behavioral testing. We have corrected this in the revised Methods section.

All the scoring of MS behaviors needs to be done blind to the procedure imposed on the animals (rotation or control). If the authors have done this blind, they should say so in the methods – otherwise they need to do this analysis blind.

RESPONSE: Thank you for raising this important point regarding experimental rigor. We confirm that all scoring of MS-like behaviors in this study was performed blind to experimental conditions. We have explicitly stated this in the revised Methods section.

The authors write: "Furthermore, a pronounced reduction in body temperature after rotation provocation compared to controls (rotation versus control, −3.55 versus 0 °C)" (Line 95). What's the Standard Deviation on these delta temperature averages?

RESPONSE: As suggested, we have included the Standard Deviation for the temperature change data. The revised sentence reads as follows: "Furthermore, mice subjected to ventral vertical rotation exhibited significant reductions in body temperature (rotation versus control, −3.55 ± 0.42 °C versus 0 ± 0.20 °C) compared to controls (Fig. 1d-f), consistent with previously described MS responses in rodents." We have updated this accordingly in the revised manuscript.

The way how the schematic of the experimental protocol is presented in figure 1g is confusing, it seems as if animals were subjected to rotations only on days 1 to 5, when in fact they were rotated daily from day 1 to 10. Then again, the reader has to dig in the methods section to understand what's going on: scoring days 1-5, head-oriented angles and velocity analyzed during days 6-10. Which then raises the question, why didn't the authors do the scoring and analyze head-orientation/velocity for all days?

RESPONSE: We apologize for the confusion caused by the schematic in Figure 1g. We have revised Figure 1g to clearly indicate that mice were subjected to daily rotation from day 1 to day 10. Specifically, during the first 5 days of rotation, mice exhibited transient tumbling and retropulsion immediately after the stimulus, which gradually transitioned to prolonged hypoactivity. Because tumbling involves erratic, non-stereotyped movements, it cannot be reliably quantified using cumulative head-orientation angles or average velocity. Therefore, we used the behavioral scoring system (based on predefined criteria) to assess the severity of MS-like symptoms during this early phase. From day 6 onward, mice consistently displayed transient circling behavior without tumbling, followed by prolonged hypoactivity. Since behavioral scores showed no further variation across days, we switched to quantitative measures (cumulative head-orientation angles and average velocity), which provide more objective and sensitive readouts for circling behavior. This two-stage analysis approach allowed us to capture both the early dynamic changes (using scoring) and the later stable phenotype (using precise quantification). We have clarified this rationale in the revised Methods section.

There is no obvious explanation why the single rotation protocol inducing classic MS behavior results in a decrease in the average velocity of the animals, while the repetitive rotation paradigm induces MS behavior characterized by an increase in the average velocity of the animal. These are diametrically opposite results that should be at least commented upon in the manuscript. Also, it isn't clear why the authors use average

velocity, which is a roundabout way to express distance. Distance is a simpler and more direct measure of locomotion and certainly, when compared to control animals, of "hypolocomotion" (example, line 156).

RESPONSE: We are grateful for your incisive and helpful comments. To validate our single rotation-induced acute MS-like behavioral paradigm, we selected a consecutive 10 min observation window after rotation. During this period, mice immediately exhibited tumbling symptoms followed by marked hypoactivity and behavioral immobility, reflecting a sustained decrease in overall locomotor activity. This prolonged depressive effect on locomotion is a hallmark of acute MS-like responses and confirms the successful establishment of our acute MS paradigm. Thus, the observed decrease in average velocity reflects this generalized suppression of movement.

In contrast, the repeated rotation paradigm was designed to capture the signature circling phenotype of MS-like behavior. This circling behavior occurs immediately after rotation onset, is transient (lasting approximately 10-20 s), and represents an active, directional motor response. To sensitively detect and compare group differences within this brief but behaviorally critical window, we specifically analyzed the first 10 s post-rotation. Because circling inherently involves active locomotion, average velocity transiently increases during this window. Thus, the increased velocity in this short window reflects a specific acute motor output rather than an attenuation of the overall MS-like state. Therefore, the opposite changes in velocity are not contradictory but rather reflect the different behavioral states and measurement windows. Additionally, we have included data on autonomic responses (appetite and body temperature) across the 10-day repeated rotation paradigm. Our results show that repeatedly rotated mice exhibit significant and sustained reductions in both appetite and body temperature compared to controls, consistent with classical MS symptoms (Response Document Figure 26, and also see new Extended Data Figure 1l,m). These results demonstrate that the repeated rotation model not only induces a quantifiable acute vestibular-driven circling response but also induces sustained autonomic features characteristic of MS.

We agree with you that distance is a simple and direct measure of locomotion. The average velocity and distance are equivalent within a fixed time window in our analysis. We chose to report average velocity because it more directly reflects the intensity of continuous, directional movement (such as circling). We have clarified these points in the revised Discussion sections.

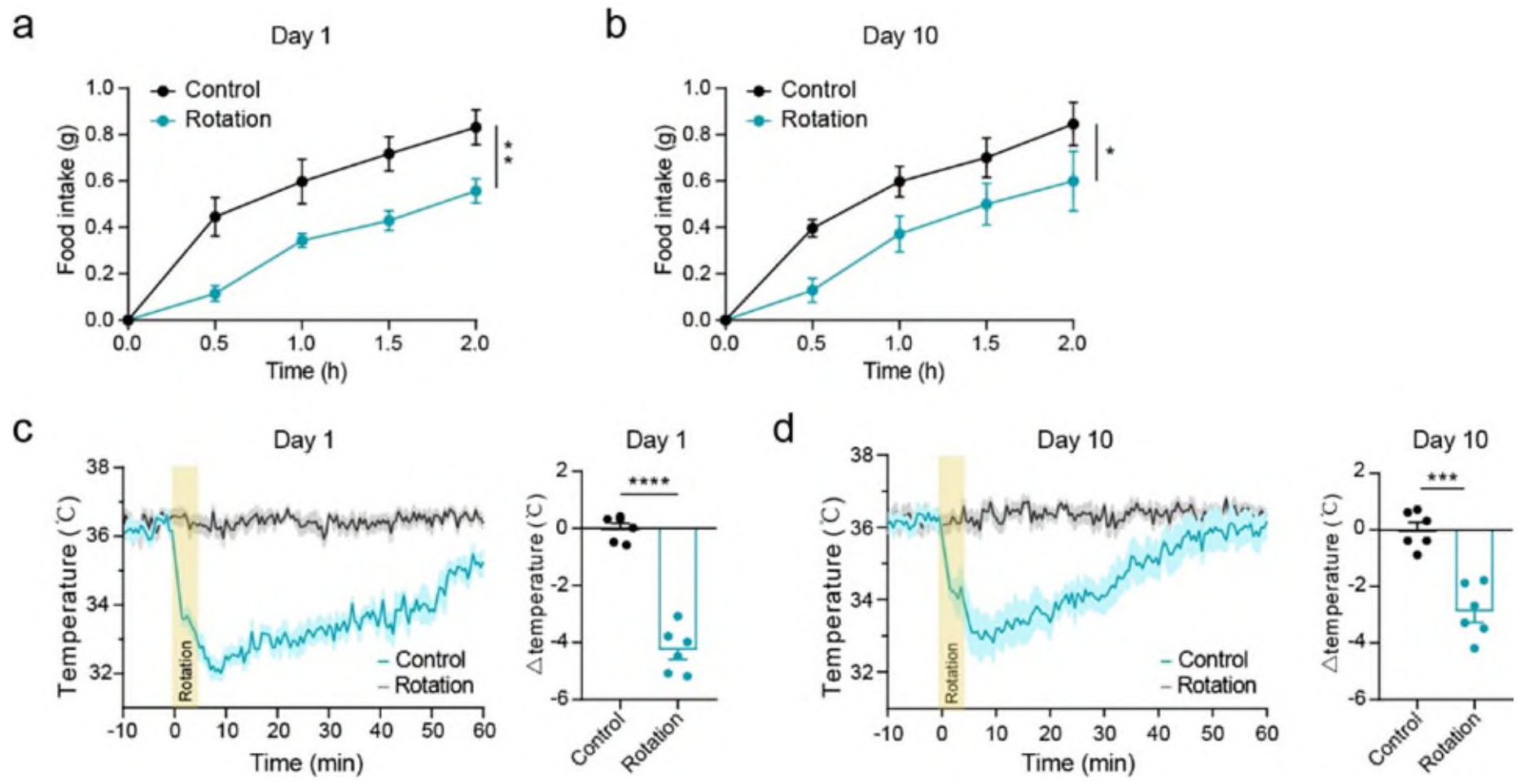

Response Document Figure 26: Effect of repeated rotation-induced MS-like behavior on appetite and body temperature in mice. a,b, Repeated rotation-induced cumulative food intake in hungry mice on days 1 and 10 (n = 6 mice; two-sided Student's t-test, *P < 0.05, **P < 0.01). **c,d,** Body temperature changes over 1 h (left) and body temperature differences at 10 min (right) after rotation or control stimulation in mice on days 1 and 10 (n = 6 mice; two-sided Student's t-test, ****P < 0.0001).

The authors write: "Here, to identify the brain regions that were activated by rotation stimulus, immunostaining was performed on the expression of the immediate early gene c-Fos. Neuronal activity in the MVN was significantly elevated during the rotation stimulus than during the control stimulus (Fig. 1m)" (line 107-111). Quantification of c-fos expression in the MVN should be in this main figure. Then, the authors write, "The NTS, CeA, PBN, and ventrolateral periaqueductal gray (vlPAG) were also slightly activated (Fig. S2a, b)." "Slightly" is not a quantifiable measure, the authors need to show quantification of c-fos expression in these regions as well (in Fig. S2b).

RESPONSE: Thank you for your detailed and thoughtful suggestions. As suggested, we have included the quantification of *c-Fos* expression in the MVN directly in Figure 1n of the main text. Furthermore, we have added quantification of *c-Fos* expression in

the NTS, CeA, PBN, and vlPAG in Figure S2b (Response Document Figure 27, and also see new Extended Data Figure 1c).

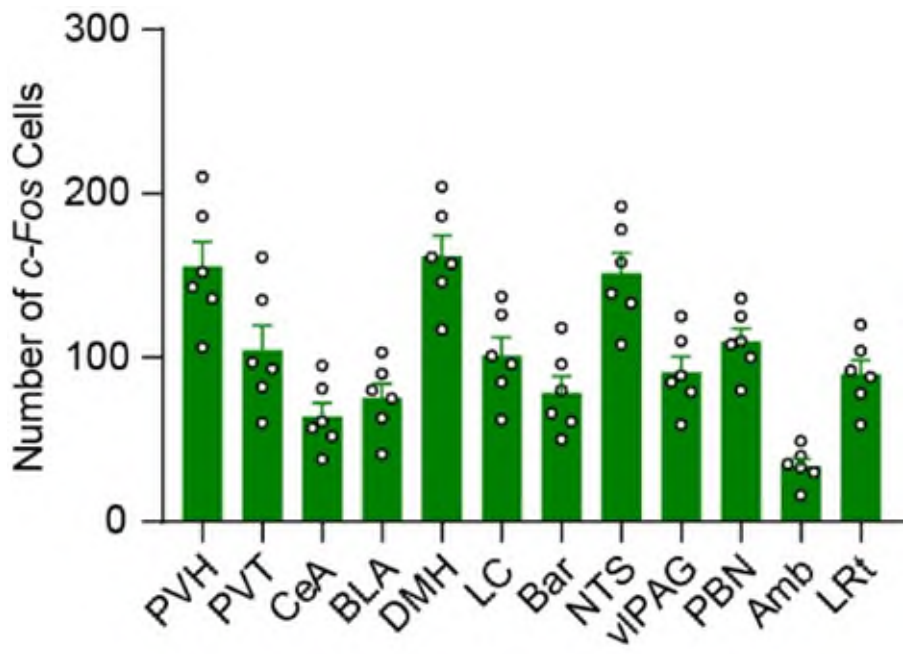

Response Document Figure 27: Number of c-Fos$^+$ cells induced by rotation stimulation in whole-brain regions.

Spell out SPL (line 120).

RESPONSE: As suggested, we have revised the manuscript accordingly: the abbreviation SPL is spelled out as sound pressure levels in the revised manuscript.

Shouldn't Fig. 2b, f read Fig. 2b-f (line 125)? Shouldn't Fig. 2c-e include panel f (line 130)?

RESPONSE: As suggested, we have corrected the in-text citations as follows: "Fig. 2b, f" has been corrected to "Fig. 2b". Also, the citation correctly reads "Fig. 2c-e", and panel f is cited separately.

The schematic in figure 2a does not show 2 seconds of buffer period. Also, the grey zone overlaps with the 3 sec tone at the end, which looks like if rotations last 37 sec instead of 40 sec (this is the same issue with similar schematics in other figures).

RESPONSE: Thank you for pointing out these inaccuracies in our schematic. The 2 s buffer period has been removed from the legend of Figure 2a, as it was not part of the actual experimental protocol. The gray zone represents the 40 s rotation period, which includes the 3 s tone at the end. We have corrected Figure 2a and similar schematics in other figures to ensure consistency throughout the manuscript.

In Figure 3 panels b, c and d, the red bars are called differently: Cbln2-Cre (panel a and

b) or Cbln2-ChR2 (panel c). Why have the authors decided to call these bars differently? In Fig. 3d, presumably Cbln2-mCherry is the control that does not express ChR2? But then, doesn't Cbln2-ChR2 express mCherry as well?

RESPONSE: As suggested, we have unified the nomenclature throughout Figure 3. "*Cbln2*-ChR2" has been corrected to "*Cbln2*-Cre". In Figure 3d, the "*Cbln2*-mCherry" control group (*Cbln2*-cre mice injected with AAV-DIO-mCherry, which does not express ChR2) has been removed to avoid potential confusion. Since panel d aims to compare within the ChR2-expressing group across different laser frequencies, the inclusion of a separate mCherry control was unnecessary. We present only the *Cbln2*-Cre (ChR2) data across frequencies in panel d (Response Document Figure 28, and also see new Figure 3d).

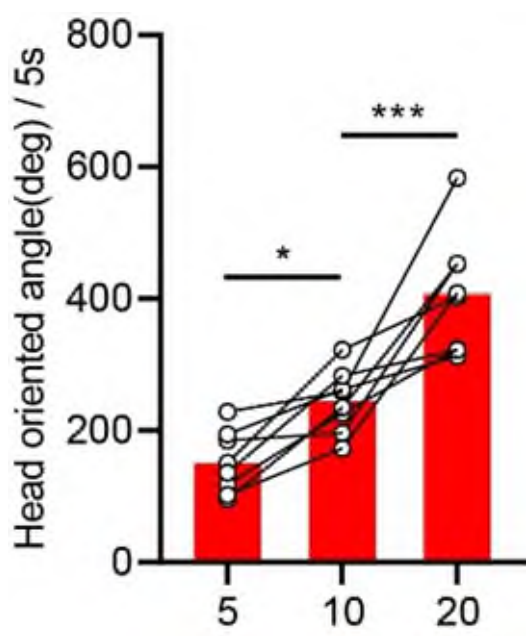

Response Document Figure 28: Quantitative analyses of cumulative head orientation angles of mice after optogenetic activation of Cbln2$^+$ MVN neurons in *Cbln2*-IRES-Cre mice. The head orientation angles of MS-like behavior during light evocation are a function of laser frequency.

In Fig. 3a-d, the authors omitted the average velocity after activation, it should be shown. In Fig. 3j, the authors quantified circling numbers, cumulative head-orientation angles should be shown. Importantly, the authors say that the activation of Cbln2+ neurons "facilitates MS-like behavior", but the average velocity actually decreases, matching only the single-rotation "classic" MS behavior, not the repetitive rotation MS behavior. Then, the ablation of Cbln2+ neurons also result in a decrease of average velocity, attenuating (not "eliminating", by the way) repetitive rotation MS behavior. The authors should present and explain these apparent discrepancies clearly and they should be discussed in detail in the Discussion section.

RESPONSE: We sincerely appreciate your insightful comments. As suggested, we have supplemented the revised manuscript and the corresponding supplementary figures with the following additional data: For Figure 3a-d, we have included the average velocity data following optogenetic activation of the Cbln2$^+$ MVN neurons (Response Document Figure 29, and also see new Extended Data Figure 3c,d). For the activation of Cbln2$^+$ neurons, we appreciate the opportunity to clarify these points: Unlike optogenetic activation, which produces precisely timed neuronal firing, chemogenetic activation via hM3Dq triggers sustained neuronal activity following CNO administration[60]. This effect persists for hours after CNO administration, resulting in continuous[61,62] tonic elevation of neuronal activity rather than phasic, time-locked firing. Upon chemogenetic activation of Cbln2$^+$ neurons, mice exhibited intermittent, stereotypic MS-like circling behavior during prolonged activation. However, because the circling occurred discontinuously and was often interrupted by periods of inactivity or other behaviors, cumulative head-orientation angle quantification became unreliable. Therefore, we quantified circling numbers instead of cumulative angles (Fig. 3j).

Importantly, the prolonged chemogenetic activation of Cbln2$^+$ neurons also induced a significant reduction in overall locomotor activity and body temperature. We interpret this pattern as reflecting the coexistence of a transient rotation-related motor output and a more sustained MS-like depressive state, resembling the hypolocomotion and hypothermia observed after acute rotation. In this context, the transient increase in movement associated with circling is outweighed by the broader suppression of spontaneous locomotion when average velocity is measured across the longer recording window.

As previously described, the repeated rotation paradigm was designed to capture the immediate, transient circling phenotype in a short 10-s post-rotation window. During this brief period, active circling behavior results in a transient increase in average velocity compared to resting or freely exploring states. Conversely, ablation of Cbln2$^+$ neurons attenuate this short-window circling behavior, leading to lower average velocity in the ablation group compared to non-ablated controls during the same 10 s

window. Therefore, the opposite directional changes in average velocity are not contradictory but rather reflect the different behavioral states and time windows being measured. We have added a detailed discussion of these points in the revised Discussion section.

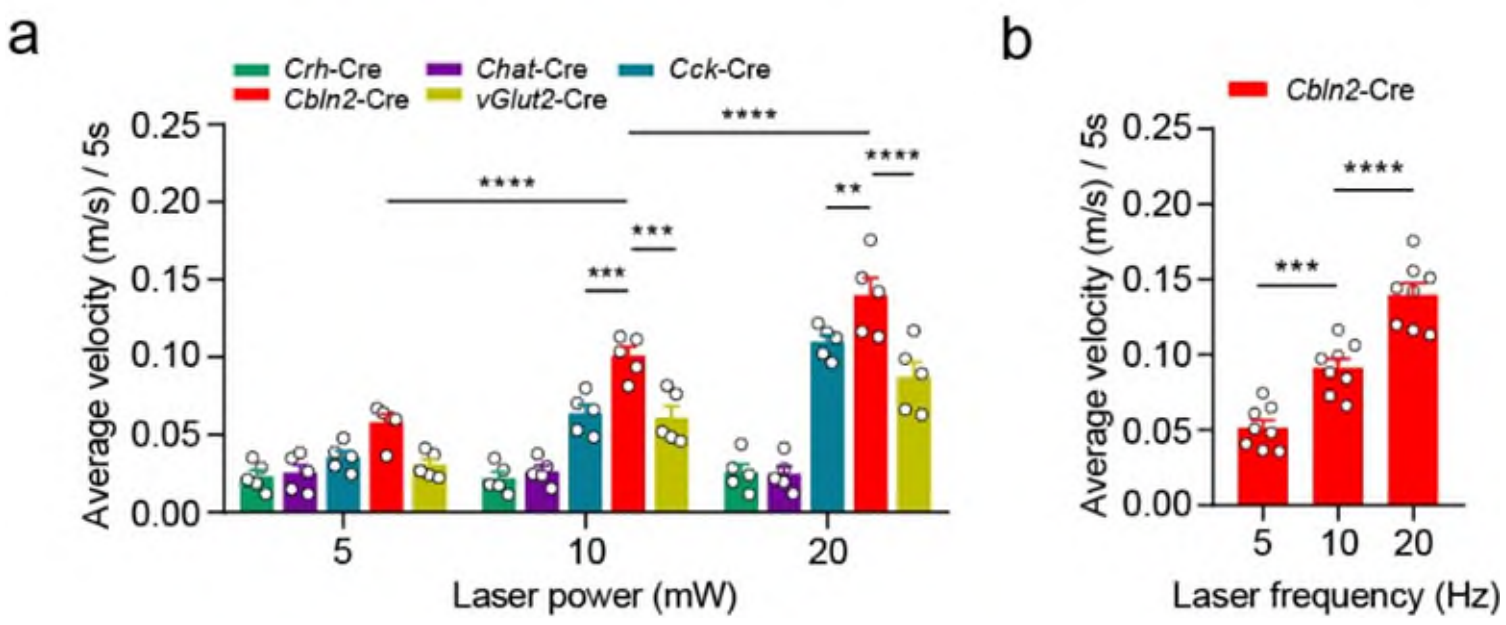

Response Document Figure 29: Average velocity of different Cre mouse lines during 5 s optogenetic activation. a, Average velocity of different Cre mouse lines during 5 s optogenetic activation under graded laser power. For *Cbln2*-IRES-Cre mice, the average velocity during light evocation is a function of laser power. **b,** For *Cbln2*-IRES-Cre mice, the average velocity during light evocation is a function of laser frequency. Statistical analysis in (a) was performed using two-way ANOVA (**P < 0.01, ***P < 0.001, ****P < 0.0001). Statistical analysis in (b) was performed using one-way ANOVA (***P < 0.001, ****P < 0.0001).

In line 163, the authors write Fig. 3i, it should read Fig. 3l.

RESPONSE: As suggested, we have corrected "Fig. 3i" to "Fig. 3l".

The authors write: "Laser-induced cumulative head-orientation angles of MS-like symptoms were correlated with laser intensity and frequency (Fig. 3c, d). A light pulse train (473 nm, 1 ms, 20 mW, 20 Hz) reliably evoked phase-locked spiking activity in Cbln2+ neurons expressing ChR2-mCherry (Fig. 3e)." The authors jump from in vivo behavioral optogenetic experiments to in vitro brain physiology, but do not tell the reader. By the way, the protocol on slices also includes 10Hz, not only 20Hz.

RESPONSE: As suggested, we have revised the sentence and accurately reflect the stimulation parameters used, as follows: In acute MVN slices, a light pulse train (473 nm, 1 ms, 20 mW, 10 or 20 Hz) reliably evoked phase-locked spiking activity in $Cbln2^{+}$ neurons expressing ChR2-mCherry (Fig. 3e). We have updated this accordingly in the revised manuscript.

Figure 4d, can the authors clarify what they are measuring? The scale bar reads 25 Hz, which is wrong. What are the black traces, what is the red trace?

RESPONSE: For Figure 4d, we characterized the in vitro electrophysiological properties of Cbln2$^+$ MVN neurons to examine their firing responses to depolarizing current injection. We agree that the original scale bar label was incorrect; "25 Hz" has been corrected to "25 mV". In the left panel, the red traces show representative membrane potential responses of an EGFP-labeled Cbln2$^+$ MVN neuron to depolarizing current steps of 0.1, 0.2, and 0.3 nA. The black traces below indicate the corresponding injected current steps. These recordings show that Cbln2$^+$ MVN neurons generate action potentials with a gradually adapting firing pattern in response to depolarizing current injection. In the right panel, the black lines represent the spike numbers recorded from individual cells at each injected current intensity, whereas the red line indicate the mean ± s.e.m. across all recorded cells. Thus, this panel quantifies the input-output relationship between injected current and evoked spike number in Cbln2$^+$ MVN neurons. We have updated the figure legend accordingly to clearly describe the experimental method.

The legend of Fig. 4g reads “Ca2+ signals associated with the trial of MS-like behavioral session. Heatmap showing 27 trials of normalized GCaMP fluorescence changes (△F/F) aligned with MS-like behavior induced by ventral rotation (top)”. It is impossible to understand what the authors mean by “aligned with MS-like behavior”. It is, presumably, aligned to the onset of rotation.

RESPONSE: As suggested, we have revised the sentence, as follows: Heatmap of normalized GCaMP fluorescence changes (ΔF/F) across 27 trials from 4 mice, aligned to ventral rotation onset (5 g, 2 circles) (top). We have updated this accordingly in the revised figure legend.

In general, the colocalization figures should be explained better (Figures S4, S5, S6,

S11). Example, in Fig. S6a, the legend reads "The proportion of co-localized neurons in Cbln2 mRNA and GCamp6s+ neurons (n = 8 fields from 4 mice)", which is already confusing, and then the bars are labeled "Co/GCamp6s+" and "Co/Cbln2+". It isn't clear enough and the Methods section does not help.

RESPONSE: We sincerely appreciate your detailed and thoughtful suggestions. We have revised the corresponding supplementary figure legends. For example, in Figure S6a, the legend reads: Quantitative analyses of the specificity and efficiency of GCaMP6s expression for labeling *Cbln2* mRNA^{+} MVN neurons in *Cbln2*-IRES-Cre mice. We have also clarified the meaning of the labels in the figure. "Co/GCaMP6s^{+}" indicates the percentage of GCaMP6s^{+} neurons that were also positive for *Cbln2* mRNA (i.e., the specificity of GCaMP6s expression for *Cbln2* mRNA^{+} neurons). "Co/Cbln2^{+}" indicates the percentage of *Cbln2* mRNA^{+} neurons that were also GCaMP6s^{+} (i.e., the labeling efficiency of GCaMP6s for Cbln2^{+} neurons). These clarifications have been incorporated into the revised figure legends for Figures S4, S5, S6, and S11.

The authors write: "In contrast, we did not observe changes in fluorescent intensity in EGFP-expressing Cbln2+ MVN neurons, indicating that the recorded signals were not due to motion artifacts." Where is the reader supposed to see this? The reader is supposed to look at figure 4g, but then, what's the red trace, what's the grey trace?

RESPONSE: As suggested, we have revised the manuscript and figure legends. First, we have added a citation to Figure 4g after this sentence in the Results section. Second, we have updated the legend for Figure 4g: "Red lines indicate recordings from GCaMP6s^{+} cells; gray lines indicate recordings from reference EGFP^{+} cells. Thick lines represent the mean, and shaded areas indicate s.e.m. Gray dashed line: onset of ventral rotation; blue shaded area: duration of ventral rotation". We have applied similar clarifications to the legends of all other calcium imaging figures.

The schematic in Figure 4i is totally useless. What needs to be shown, and in fact above all photometry traces, is the rotation angle of the chamber over time – or even better,

acceleration over time. Related to this, the authors should explain precisely how they measured acceleration.

RESPONSE: We agree that the previous schematic was insufficient. In fact, our results show that Cbln2⁺ MVN neurons encode both the rotational acceleration and changes in tail/body position. Specifically, we observed that calcium signals increased when mice were in the ventral-up (supine) position and decreased when mice returned to the normal prone position, while rotational speed remained constant during the stimulation period. To better illustrate Figure 4 results, we have replaced Figure 4i with a new panel showing the correlation between GCaMP activity and behavior, which more directly demonstrates the functional relevance of Cbln2⁺ neuron activity to MS-like behavior (Response Document Figure 30d, and also see new Figure 4i). Additionally, we have added a detailed description of how acceleration was calculated in the revised Methods section: "Rotational speed was set at 300 ± 5 rpm to generate 5.0 ± 0.2 *g* centripetal acceleration, calculated using the formula $a = (2\pi n/60)^2 \times r$ (where n represents rotational speed in rpm, and r indicates rotation radius in meters)".

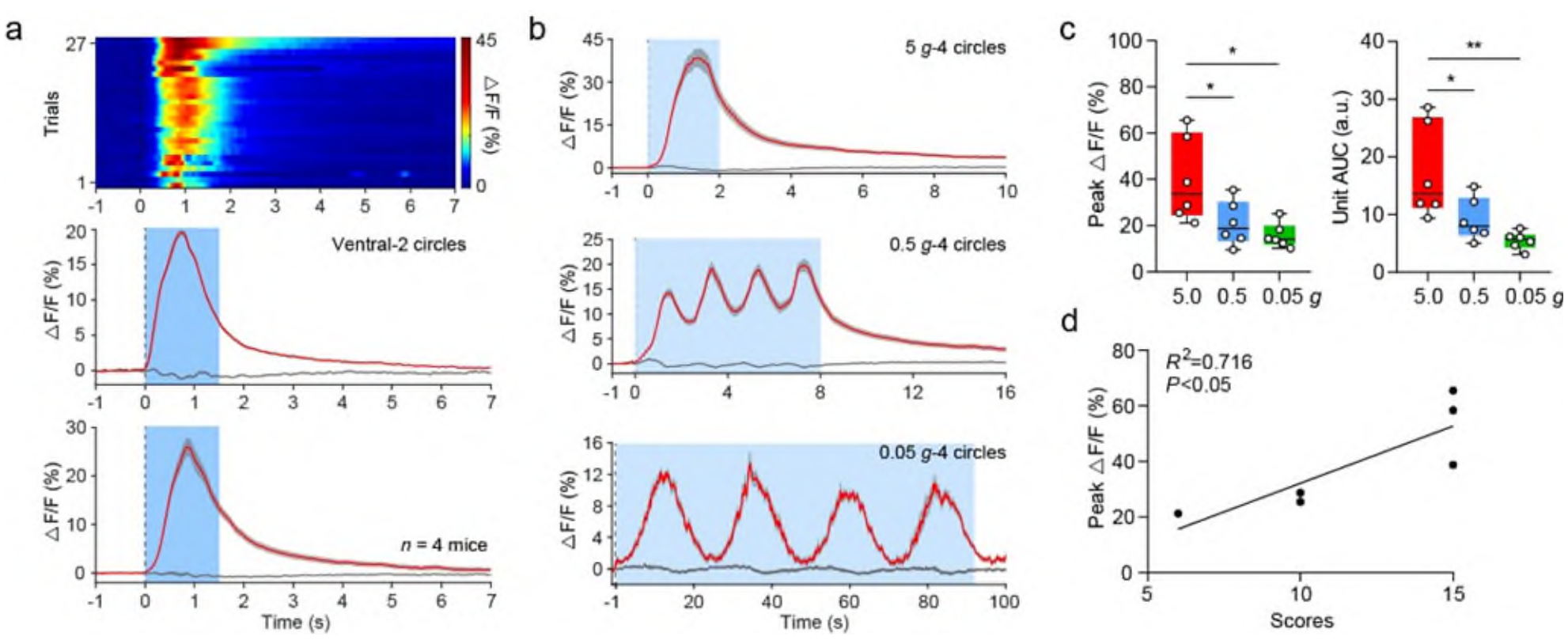

Response Document Figure 30: a, Ca2⁺ signals associated with the trial of MS-like behavioral session. Heatmap of normalized GCaMP fluorescence changes (ΔF/F) across 27 trials from 4 mice, aligned to ventral rotation onset (5 *g*, 2 circles) (top); normalized GCaMP signal changes in Cbln2⁺ MVN neurons during MS-like behavior induced by ventral rotation in individual mice (middle); mean Ca2⁺ transients for the entire test group (*n* = 4 mice) (bottom). **b,** GCaMP signal responses to ventral rotations of different intensities (5 *g*, 0.5 *g*, and 0.05 *g*, 4 circles) (*n* = 6 mice). Red lines indicate recordings from GCaMP6s⁺ cells, gray lines indicate recordings from reference EGFP⁺ cells. Thick lines represent the mean, and shaded areas indicate s.e.m. Gray dashed line: onset of ventral rotation; Blue shaded areas: duration of ventral rotation. **c,** Quantitation of peak amplitude (left) and unit AUC (right) of GCaMP responses during ventral rotations of different intensities (*n* = 6 mice; one-way ANOVA, *P < 0.05, **P < 0.01). **d,** GCaMP responses of the vertical rotation (5.0 *g*, 4 circles) linearly correlated with the severity of MS-like behavioral symptoms on a per-

animal basis.

All quantifications of peak deltaF/F in function of acceleration should be incorporated in the main figures, not left in supplementary figures.

RESPONSE: As recommended, we have revised the figures accordingly. All quantifications of peak ΔF/F as a function of acceleration are incorporated into the main figures.

The authors do not explain what the pharmacological cocktail applied on brain slices show. Also, the authors have not performed in vivo electrophysiology, as claimed in the authors contributions section.

RESPONSE: We thank you for pointing this out, we have added an explanation in the revised manuscript. Specifically, we perfused either glutamate receptor antagonists (AP5, 50 μM; CNQX, 20 μM) or GABA receptor antagonists (PTX, 50 μM) to determine whether each set of antagonists completely abolished the evoked responses, thereby identifying the neurotransmitter phenotype of these neurons. For Figure 5g and Figure 6h, explanation is as follows: "Light pulses (473 nm, 2 ms, 20 mW) reliably evoked postsynaptic currents (PSCs) in vlPAG or NTS neurons, and these responses were abolished by CNQX and AP5, indicating glutamatergic transmission". Additionally, we apologize for the inaccuracy in the author contributions section. we have corrected "in vivo electrophysiology" to "in vivo fiber photometry".

Figure 5f, in the legends, the authors call correctly panel e “laser power”, but wrongly in the figure itself; and then panel f legends refers to “intensity”, when it should read “frequency”.

RESPONSE: As suggested, we have revised Figure 5f and its corresponding legend accordingly. Specifically, for Figure 5f, "laser intensity" has been corrected to "laser power". For panel f legends, "laser intensity" has been corrected to "laser frequency" to describe the stimulation parameter accurately.

The authors write: “Interestingly, at the onset of the rotation with a 3-s tone stimulus on day 17, pronounced decreases in GCaMP fluorescence were observed in the Rotation+tone group compared with the Rotation group (Fig. 7c).” Suddenly, the analysis is performed in day 17, no explanation given. Why not in day 10? Is it because rotations start in day 6 (that is, 6 days after virus injection) and, therefore, what they call day 17 is actually day 11 after the beginning of the repetitive rotation paradigm?

RESPONSE: We apologize for the confusion caused by our imprecise description of the time points in the original manuscript. Specifically, rotation began on day 6 (6 days after viral injection). What were originally labeled as days 17 and 18 in the manuscript actually correspond to days 10 and 11 of repeated rotation, respectively. We have corrected this labeling in the revised manuscript to avoid any misunderstanding.

We again thank you for your time, meticulous review, and helpful suggestions, which have greatly contributed to improving the rigor and clarity of our work.

References:

[1] Murakami, D. M., Erkman, L., Hermanson, O., Rosenfeld, M. G. & Fuller, C. A., Evidence for vestibular regulation of autonomic functions in a mouse genetic model. *Proc Natl Acad Sci U S A* **99** 17078 (2002).

[2] Nalivaiko, E., Thermoregulation and nausea. *Handb Clin Neurol* **156** 445 (2018).

[3] Nalivaiko, E., Rudd, J. A. & So, R. H., Motion sickness, nausea and thermoregulation: The "toxic" hypothesis. *Temperature (Austin)* **1** 164 (2014).

[4] Golding, J. F., Motion sickness susceptibility. *Autonomic neuroscience: basic & clinical* **129** 67 (2006).

[5] Muth, E. R., Motion and space sickness: intestinal and autonomic correlates. *Autonomic neuroscience: basic & clinical* **129** 58 (2006).

[6] Golding, J. F. & Gresty, M. A., Motion sickness. *CURR OPIN NEUROL* **18** 29 (2005).

[7] Choi, J. S., Hong, J. E., Jung, Y. K., Kim, K. & Kim, H. J., Modulation of vestibular function and receptor expression by experimental hypergravity in a rat model. *SCI REP-UK* **15** 23377 (2025).

[8] Fang, S. *et al.*, Effects of vestibular habituation training on synaptic plasticity in the medial prefrontal cortex of mice with motion sickness. *NEUROSCI LETT* **875** 138524 (2026).

[9] Machuca-Marquez, P. *et al.*, Vestibular CCK signaling drives motion sickness-like behavior in mice. *Proc Natl Acad Sci U S A* **120** e1990034176 (2023).

[10] Zhang, L. L. *et al.*, Motion Sickness: Current Knowledge and Recent Advance. *CNS NEUROSCI THER* **22** 15 (2016).

[11] Reason, J. T., Motion sickness adaptation: a neural mismatch model. *J R Soc Med* **71** 819 (1978).

[12] Bles, W., Bos, J. E., de Graaf, B., Groen, E. & Wertheim, A. H., Motion sickness: only one provocative conflict? *BRAIN RES BULL* **47** 481 (1998).

[13] Cohen, H., Cohen, B., Raphan, T. & Waespe, W., Habituation and adaptation of the vestibuloocular reflex: a model of differential control by the vestibulocerebellum. *EXP BRAIN RES* **90** 526 (1992).

[14] Yen, P. S. F., Billar, J., Gresty, M. A. & Golding, J. F., Effect of a novel motion desensitization training regime and controlled breathing on habituation to motion sickness. *Percept Mot Skills* **101** 244 (2005).

[15] Golding, J. F., Motion sickness susceptibility. *Auton Neurosci* **129** 67 (2006).

[16] Dumontheil, I., Panagiotaki, P. & Berthoz, A., Dual adaptation to sensory conflicts during whole-body rotations. *BRAIN RES* **1072** 119 (2006).

[17] Ossenkopp, K. P., Rabi, Y. J., Eckel, L. A. & Hargreaves, E. L., Reductions in body temperature and spontaneous activity in rats exposed to horizontal rotation: abolition following chemical labyrinthectomy. *PHYSIOL BEHAV* **56** 319 (1994).

[18] Wilpizeski, C. R., Lowry, L. D., Miller, R. A., Smith, B. J. & Goldman, W., Adaptation and habituation of motion-induced vomiting in squirrel monkeys. *Aviat Space Environ Med* **58** A22 (1987).

[19] Angelaki, D. E. & Cullen, K. E., Vestibular system: the many facets of a multimodal sense. *ANNU REV NEUROSCI* **31** 125 (2008).
[20] Cullen, K. E., The vestibular system: multimodal integration and encoding of self-motion for motor control. *TRENDS NEUROSCI* **35** 185 (2012).
[21] Shayman, C. S. *et al.*, Frequency-dependent integration of auditory and vestibular cues for self-motion perception. *J NEUROPHYSIOL* **123** 936 (2020).
[22] Illing, R., Michler, S. A., Kraus, K. S. & Laszig, R., Transcription factor modulation and expression in the rat auditory brainstem following electrical intracochlear stimulation. *EXP NEUROL* **175** 226 (2002).
[23] Wang, H., Brozoski, T. J. & Caspary, D. M., Inhibitory neurotransmission in animal models of tinnitus: maladaptive plasticity. *HEARING RES* **279** 111 (2011).
[24] Sah, P., Faber, E. S. L., Lopez De Armentia, M. & Power, J., The amygdaloid complex: anatomy and physiology. *PHYSIOL REV* **83** 803 (2003).
[25] Winer, J. A. & Lee, C. C., The distributed auditory cortex. *HEARING RES* **229** 3 (2007).
[26] Munoz-Lopez, M. M., Mohedano-Moriano, A. & Insausti, R., Anatomical pathways for auditory memory in primates. *FRONT NEUROANAT* **4** 129 (2010).
[27] Du, Y., Huang, Q., Wu, X., Galbraith, G. C. & Li, L., Binaural unmasking of frequency-following responses in rat amygdala. *J NEUROPHYSIOL* **101** 1647 (2009).
[28] Savage, L. M. & Ramos, R. L., Reward expectation alters learning and memory: the impact of the amygdala on appetitive-driven behaviors. *BEHAV BRAIN RES* **198** 1 (2009).
[29] Boso, M., Politi, P., Barale, F. & Enzo, E., Neurophysiology and neurobiology of the musical experience. *Functional neurology* **21** 187 (2006).
[30] Limb, C. J., Structural and functional neural correlates of music perception. *The anatomical record. Part A, Discoveries in molecular, cellular, and evolutionary biology* **288** 435 (2006).
[31] Omar, R. *et al.*, The structural neuroanatomy of music emotion recognition: evidence from frontotemporal lobar degeneration. *NEUROIMAGE* **56** 1814 (2011).
[32] LeDoux, J., The amygdala. *Current biology: CB* **17** R868 (2007).
[33] Tahera, Y., Meltser, I., Johansson, P. & Canlon, B., Restraint stress modulates glucocorticoid receptors and nuclear factor kappa B in the cochlea. *NEUROREPORT* **17** 879 (2006).
[34] Tahera, Y., Meltser, I., Johansson, P., Salman, H. & Canlon, B., Sound conditioning protects hearing by activating the hypothalamic-pituitary-adrenal axis. *NEUROBIOL DIS* **25** 189 (2007).
[35] Galaj, E. *et al.*, Muscarinic and NMDA Receptors in the Substantia Nigra Play a Role in Reward-Related Learning. *The international journal of neuropsychopharmacology* **26** 80 (2023).
[36] Grau-Perales, A. B., Gámiz, F. & Gallo, M., Effect of hippocampal 6-OHDA lesions on the contextual modulation of taste recognition memory. *BEHAV BRAIN RES* **409** 113320 (2021).
[37] Sederholm, F. & Swedberg, M. D. B., Establishment of auditory discrimination and detection of tinnitus induced by salicylic acid and intense tone exposure in the rat.

BRAIN RES **1510** 48 (2013).
38 Wang, H. *et al.*, A molecularly defined amygdala-independent tetra-synaptic forebrain-to-hindbrain pathway for odor-driven innate fear and anxiety. *NAT NEUROSCI* **27** 514 (2024).
39 Yang, C. F. *et al.*, Sexually dimorphic neurons in the ventromedial hypothalamus govern mating in both sexes and aggression in males. *CELL* **153** 896 (2013).
40 Gao, T. *et al.*, G-Protein-Coupled Estrogen Receptor (GPER) in the Rostral Ventromedial Medulla Is Essential for Mobilizing Descending Inhibition of Itch. *The Journal of neuroscience: the official journal of the Society for Neuroscience* **41** 7727 (2021).
41 Yeh, C. *et al.*, Mossy Cells Control Adult Neural Stem Cell Quiescence and Maintenance through a Dynamic Balance between Direct and Indirect Pathways. *NEURON* **99** 493 (2018).
42 Angelaki, D. E. & Yakusheva, T. A., How vestibular neurons solve the tilt/translation ambiguity. Comparison of brainstem, cerebellum, and thalamus. *ANN NY ACAD SCI* **1164** 19 (2009).
43 Angelaki, D. E., Bush, G. A. & Perachio, A. A., Two-dimensional spatiotemporal coding of linear acceleration in vestibular nuclei neurons. *The Journal of neuroscience: the official journal of the Society for Neuroscience* **13** 1403 (1993).
44 Carter, M. E., Soden, M. E., Zweifel, L. S. & Palmiter, R. D., Genetic identification of a neural circuit that suppresses appetite. *NATURE* **503** 111 (2013).
45 Wilpizeski, C. R. & Lowry, L. D., A two-factor model of rotation-induced motion sickness syndrome in squirrel monkeys. *AM J OTOLARYNG* **8** 7 (1987).
46 Sugiyama, Y., Suzuki, T., DeStefino, V. J. & Yates, B. J., Integrative responses of neurons in nucleus tractus solitarius to visceral afferent stimulation and vestibular stimulation in vertical planes. *American journal of physiology. Regulatory, integrative and comparative physiology* **301** R1380 (2011).
47 Porter, J. D. & Balaban, C. D., Connections between the vestibular nuclei and brain stem regions that mediate autonomic function in the rat. *Journal of vestibular research: equilibrium & orientation* **7** 63 (1997).
48 Balaban, C. D. & Beryozkin, G., Vestibular nucleus projections to nucleus tractus solitarius and the dorsal motor nucleus of the vagus nerve: potential substrates for vestibulo-autonomic interactions. *EXP BRAIN RES* **98** 200 (1994).
49 Aleksandrov, V. G., Bagaev, V. A. & Nozdrachev, A. D., Gastric related neurons in the rat medial vestibular nucleus. *NEUROSCI LETT* **250** 66 (1998).
50 Gagliuso, A. H., Chapman, E. K., Martinelli, G. P. & Holstein, G. R., Vestibular neurons with direct projections to the solitary nucleus in the rat. *J NEUROPHYSIOL* (2019).
51 Arshian, M. S. *et al.*, Effects of visceral inputs on the processing of labyrinthine signals by the inferior and caudal medial vestibular nuclei: ramifications for the production of motion sickness. *EXP BRAIN RES* **228** 353 (2013).
52 Kodama, T. *et al.*, Neuronal classification and marker gene identification via single-cell expression profiling of brainstem vestibular neurons subserving cerebellar learning. *J NEUROSCI* **32** 7819 (2012).

[53] Ohno, K., Takeda, N., Yamano, M., Matsunaga, T. & Tohyama, M., Coexistence of acetylcholine and calcitonin gene-related peptide in the vestibular efferent neurons in the rat. *BRAIN RES* **566** 103 (1991).
[54] Nijsen, M. J. *et al.*, The role of the CRH type 1 receptor in autonomic responses to corticotropin-releasing hormone in the rat. *NEUROPSYCHOPHARMACOL* **22** 388 (2000).
[55] Abe, C. *et al.*, VGLUT2-expressing neurons in the vestibular nuclear complex mediate gravitational stress-induced hypothermia in mice. *Commun Biol* **3** 227 (2020).
[56] Q. Montardy, M. W. X. L., Selective optogenetic stimulation of glutamatergic, but not GABAergic, vestibular nuclei neurons induces immediate and reversible postural imbalance in mice. *PROG NEUROBIOL* **204** 102085 (2021).
[57] Zachou, A. & Bronstein, A. M., Vestibulo-spatial navigation: pathways and sense of direction. *J NEUROPHYSIOL* **129** 672 (2023).
[58] Solié, C. *et al.*, Superior Colliculus to VTA pathway controls orienting response and influences social interaction in mice. *NAT COMMUN* **13** 817 (2022).
[59] Villalobos, C. A. & Basso, M. A., Optogenetic activation of the inhibitory nigro-collicular circuit evokes contralateral orienting movements in mice. *CELL REP* **39** 110699 (2022).
[60] K Hler, I. *et al.*, Chemogenetic activation of Gq signaling modulates dendritic development of cortical neurons in a time- and layer-specific manner. *FRONT CELL NEUROSCI* **19** 1524470 (2025).
[61] Ne Oldová, M. & Stuchlík, A., Chemogenetic Tools and their Use in Studies of Neuropsychiatric Disorders. *PHYSIOL RES* **73** S449 (2024).
[62] Campbell, E. J. & Marchant, N. J., The use of chemogenetics in behavioural neuroscience: receptor variants, targeting approaches and caveats. *BRIT J PHARMACOL* **175** 994 (2018).

Response to Reviews

Manuscript ID: NCOMMS-25-83869

Title: The Neural Mechanism of Motion Sickness Alleviation by Anticipatory Sound

We sincerely appreciate the positive and constructive feedback provided by the editor and the reviewers. Guided by these thoughtful comments, we have thoroughly revised the manuscript to address all remaining concerns, including the statistical and readability issues raised by Reviewer #1. A revised version of the manuscript with tracked changes (highlighted in blue) has been uploaded as a separate file. Detailed point-by-point responses to each of the reviewers' comments are presented below.

Contents:

Response to Reviewer #1: Page 02-03

Response to Reviewer #2: Page 04

Response to Reviewer #3: Page 05

Response to Reviewer' comments:

Reviewer #1 (Remarks to the Author):

I thank the authors for their careful responses. The revised manuscript satisfactorily addresses the concerns raised in my initial review.

RESPONSE: We sincerely thank you for your thoughtful and constructive review. We are very glad that our revisions have satisfactorily addressed your concerns. Your input has been invaluable in strengthening this manuscript. Thank you again for your time and expertise.

I have two remaining minor suggestions.

1. Regarding the statistical analyses: several comparisons rely on a Student's t-test, which assumes normally distributed data. Given the small sample sizes in these experiments, normality cannot be reliably assessed, and I would recommend a non-parametric alternative. For paired comparisons between two conditions, a Wilcoxon signed-rank test would be more appropriate.

RESPONSE: Thank you for your insightful statistical comment. We agree that with small sample sizes, normality cannot be reliably assumed. Accordingly, we have re-analyzed all paired comparisons using the Wilcoxon signed-rank test and all unpaired comparisons using the Mann-Whitney U test. For two paired comparisons where $n = 4$ (Fig. S5c and Fig. S9c), the Wilcoxon test yielded a two-sided P value of 0.125, which is the smallest possible P value for this sample size. We therefore describe these comparisons as showing a trend, while noting that they did not reach statistical significance. All other conclusions remain unchanged. The manuscript has been updated accordingly in the Methods, Results, and figure legends. Thank you again for improving the statistical rigor of our study.

2. Regarding readability: several sentences in the manuscript are long and difficult to parse. I would encourage the authors to break these into shorter, more direct statements,

which would substantially improve the clarity of the text.

RESPONSE: Thank you very much for your constructive suggestion regarding manuscript readability. We agree that several long sentences hindered clarity. Following your advice, we have systematically restructured lengthy sentences throughout the manuscript into shorter, more direct statements. These changes are highlighted in blue in the revised manuscript. For example, on page 3, lines 53-57, the original long sentence has been broken into three shorter sentences. We believe these changes have substantially improved the clarity of the manuscript. We thank you again for your careful reading and valuable advice.

Reviewer #2 (Remarks to the Author):

The authors have addressed my concerns. The revised manuscript is much better and clearer. The authors should also be applauded for the enormous amount of data included in this manuscript.

RESPONSE: We sincerely thank you for your positive comments and for recognizing the effort in this work. We are pleased that our revisions have addressed your concerns and that the manuscript is now clearer. Your insightful comments and constructive suggestions throughout the review process have been extremely helpful in strengthening our study. We thank you again for your time and expertise in reviewing our paper.

Reviewer #3 (Remarks to the Author):

The authors have answered effectively to most of the concerns raised in the previous review and the manuscript is significantly improved. The paper is fine now.

RESPONSE: Thank you very much for your positive and encouraging feedback. We are delighted to hear that our revisions have effectively addressed your concerns and that the manuscript has significantly improved. Your insightful comments and constructive suggestions throughout the review process have been invaluable in strengthening our work. We greatly appreciate your time and expertise in reviewing our paper.
