# Supplementary material for: The Neural Mechanism of Motion Sickness Alleviation by Anticipatory Sound
Source: Nat Commun. 2026 Sep 24;17:11109. doi: 10.1038/s41467-026-77950-x (PMC13696722; doi:10.1038/s41467-026-77950-x)
Supplement: Supplementary file 2 [file 41467_2026_77950_MOESM1_ESM.pdf]

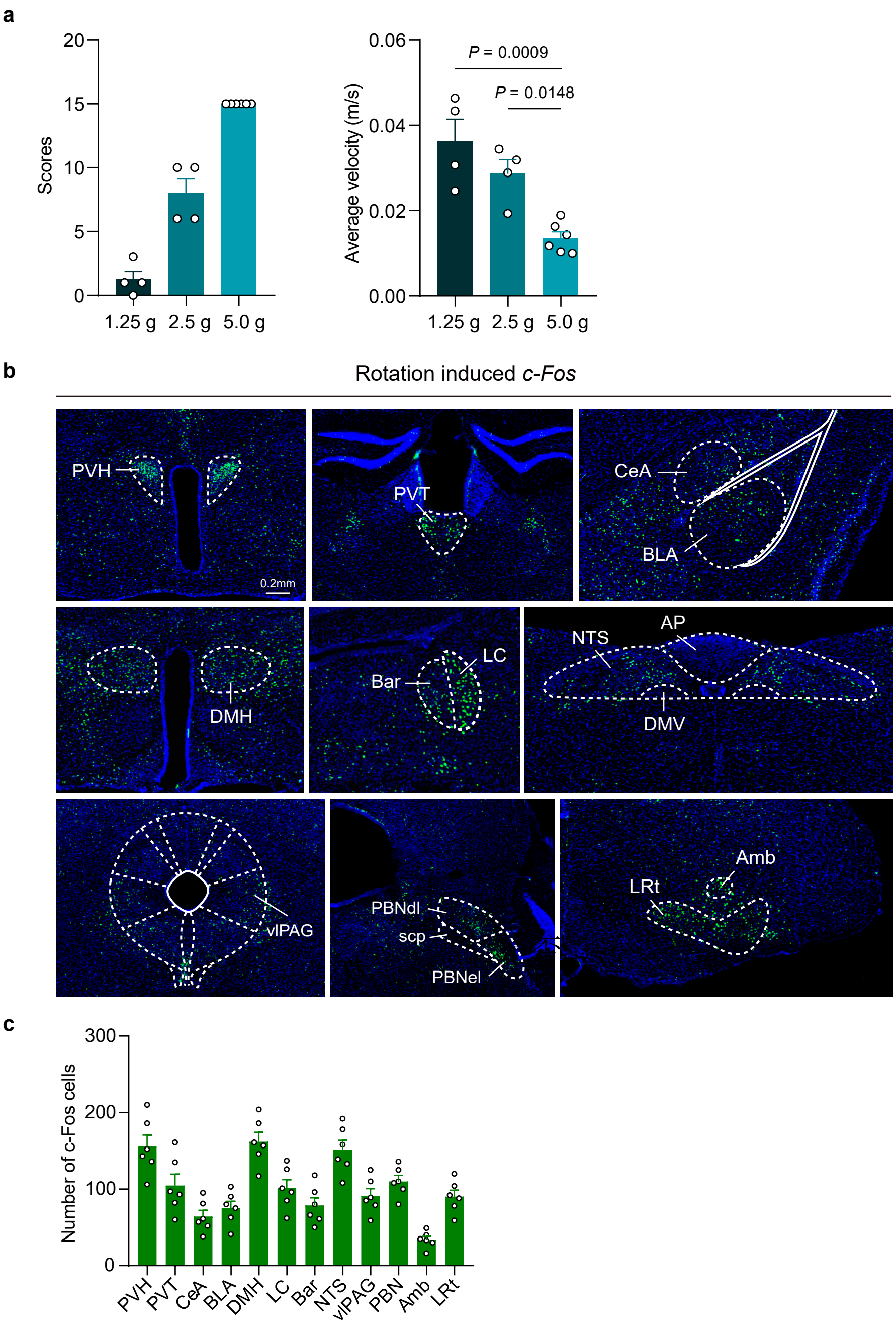

Supplementary Fig. 1 Establishment of a rotation-induced MS-like model in mice.

(a) Behavioral scores (left) and average velocity during a 10-min open-field test (right) after 40 s ventral vertical rotation at 1.25, 2.5 or 5.0 g (1.25 g and 2.5 g: n = 4 mice per group; 5.0 g: n = 6 mice). **(b)** Representative whole-brain coronal section showing c-Fos immunostaining after ventral rotation. **(c)** Quantification of c-Fos+ cells across brain regions identified from serial whole-brain sections after ventral rotation (n = 6 mice). Data are mean ± s.e.m.; error bars indicate s.e.m. Data in **(a)** were analyzed using one-way ANOVA followed by two-sided Tukey's multiple-comparisons test. ANOVA statistics are provided in Supplementary Table 4, and adjusted P values are shown in the figure. Scale bars are indicated in the images. Source data are provided as a Source Data file.

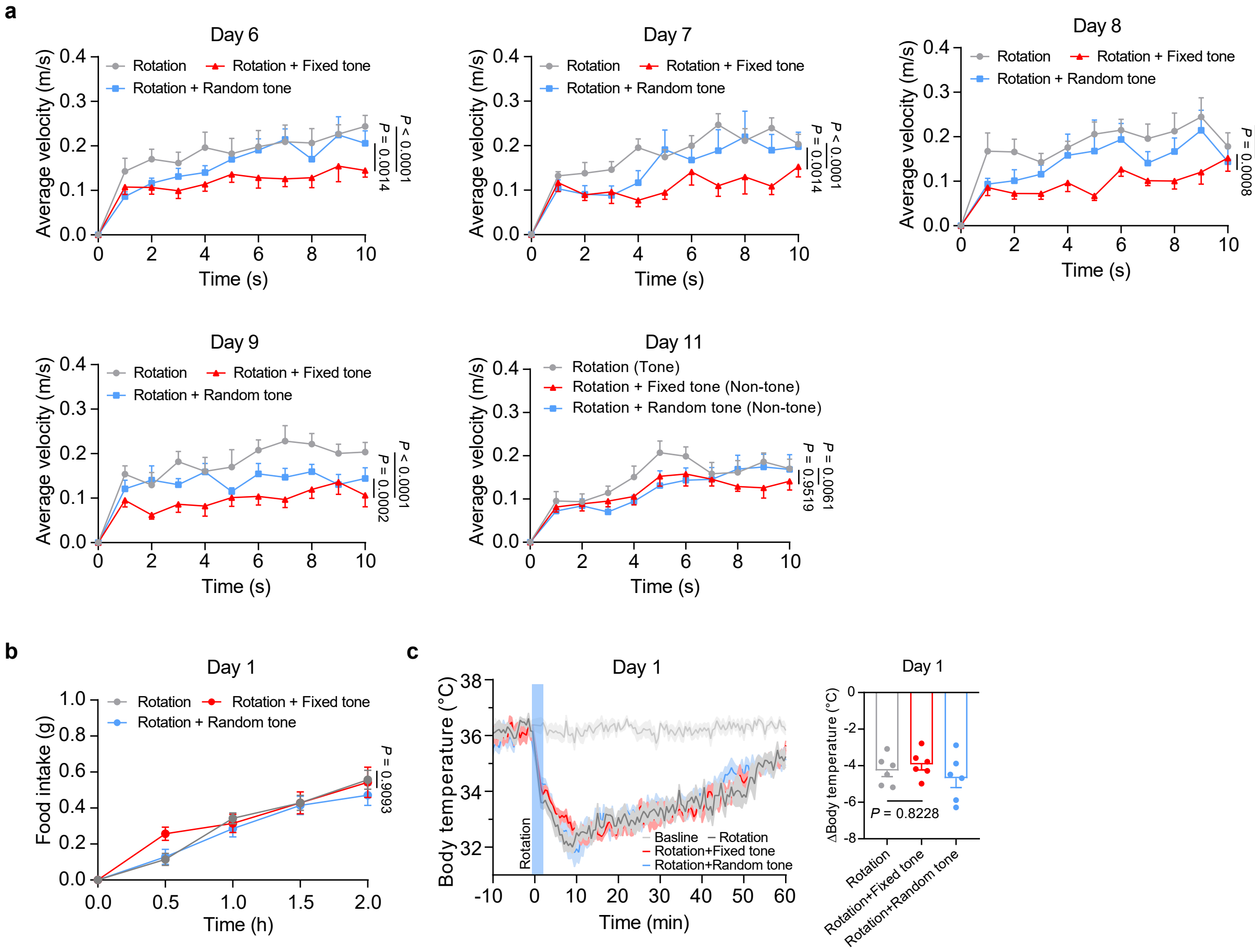

Supplementary Fig. 2 Effect of anticipatory tone on repeated rotation-induced MS-like behavior.
(a) Average velocity during the first 10 s of the post-rotation open-field test on days 6-9 and day 11 in Rotation, Rotation+Fixed tone and Rotation+Random tone mice. No tone was presented on day 11 (n = 8 mice per group). **(b)** Cumulative food intake over 2 h in hungry mice on day 1 following rotation with or without tone pairing (n = 7 mice per group). **(c)** Body temperature changes over 1 h after rotation (left) and changes from baseline at 10 min (right) on day 1 in the three groups (n = 6 mice per group). The pale-gray trace and shading indicate the baseline recording before rotation; dark-gray, red and blue traces and shading indicate the post-rotation responses in the Rotation, Rotation+Fixed tone and Rotation+Random tone groups, respectively. Data are mean ± s.e.m.; error bars and shaded areas indicate s.e.m. Data in **(a)** were analyzed using two-way ANOVA followed by two-sided Tukey's multiple-comparisons test. Data in **(b)** and **(c)** were analyzed using one-way ANOVA followed by two-sided Tukey's multiple-comparisons test. ANOVA statistics are provided in Supplementary Table 4, and adjusted P values are shown in the figure. Source data are provided as a Source Data file.

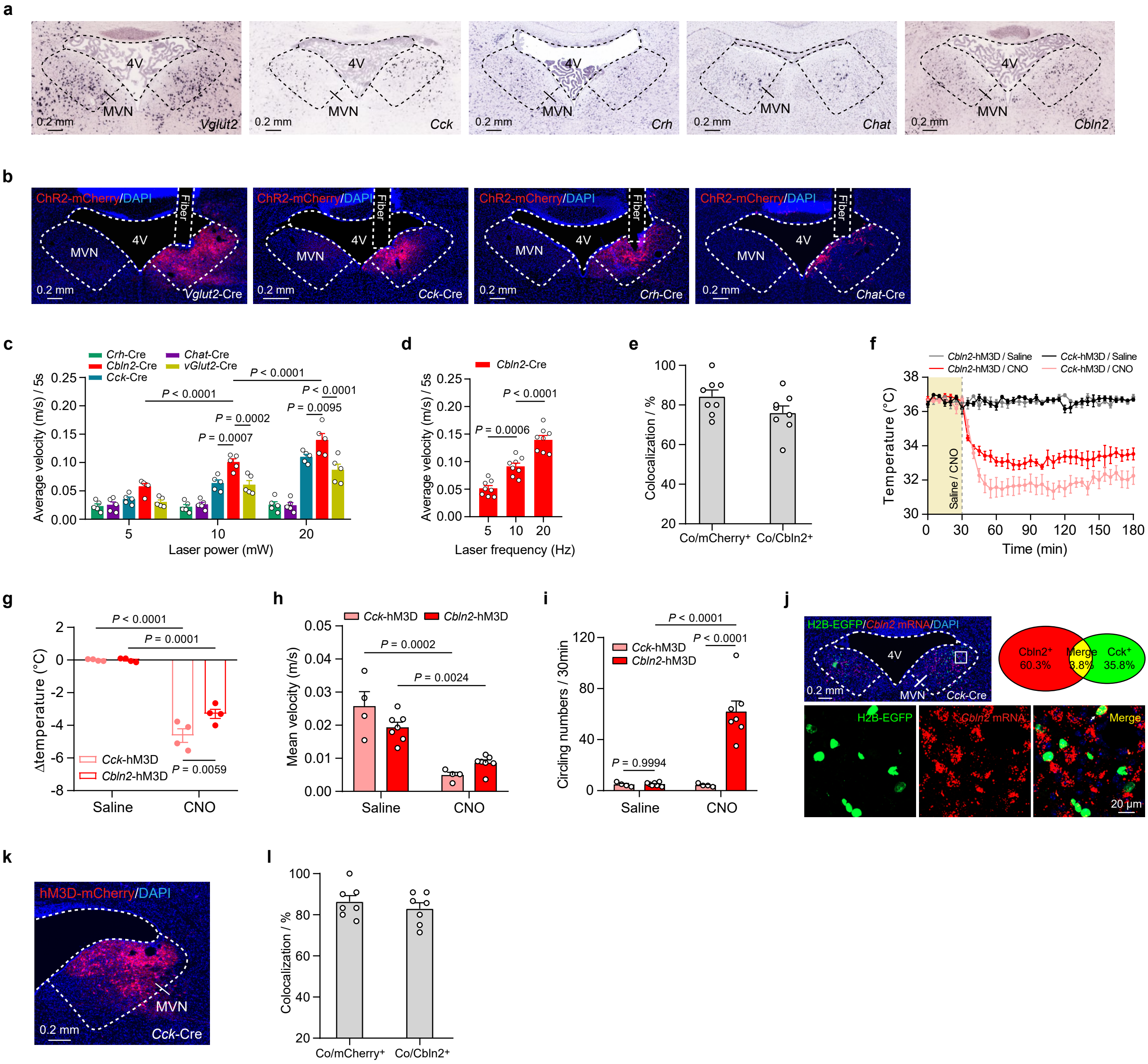

Supplementary Fig. 3 Comparison of MS-like behavior evoked by activation of Vglut2^{+}, Cck^{+}, Crh^{+}, Chat^{+}, and Cbln2^{+} MVN neurons. **(a)** In situ hybridization images from the Allen Brain Atlas showing MVN expression of marker genes. **(b)** ChR2-mCherry expression and fiber track in the MVN of *Vglut2*-IRES-Cre, *Crh*-IRES-Cre, *Cck*-IRES-Cre, and *Chat*-IRES-Cre mice. **(c)** Average velocity during optogenetic activation across five Cre lines; power responses in *Cbln2*-IRES-Cre mice were assessed at 5-20 mW (20 Hz; n = 5 per group). **(d)** Average velocity of *Cbln2*-IRES-Cre mice as a function of laser frequency (5-20 Hz at 20 mW; n = 8 mice). **(e)** Specificity and efficiency of ChR2-mCherry labeling of *Cbln2* mRNA^{+} MVN neurons (n = 8 fields from 4 mice). Co/mCherry^{+} and Co/Cbln2^{+} denote percentages of ChR2-mCherry^{+} neurons positive for *Cbln2* mRNA and *Cbln2* mRNA+ neurons positive for ChR2-mCherry, respectively. **(f, g)** Body temperature changes over 1 h **(f)** and change from baseline at 10 min **(g)** after saline or CNO activation of Cck^{+} or Cbln2^{+} MVN neurons (n = 4 mice per group). **(h, i)** Average velocity **(h)** and total circling numbers **(i)** within 30 min of saline or CNO administration (Cck-hM3D: n = 4 mice, Cbln2-hM3D: n = 7 mice). **(j)** Representative section and magnified field showing double-label in situ hybridization assay for *Cbln2* mRNA and H2B-EGFP^{+} MVN neurons of *Cck*-IRES-Cre mice; the fraction of double-label was shown on the right (n = 6 fields from 3 mice). **(k)** Representative hM3D-mCherry expression in the MVN of *Cck*-IRES-Cre mice. **(l)** Specificity and efficiency of hM3D-mCherry labeling of *Cbln2* mRNA^{+} MVN neurons, defined as in **(e)** (n = 7 fields from 4 mice). Data are mean ± s.e.m.; error bars indicate s.e.m. Data in **(c)** and **(d)** were analyzed using two-way ANOVA and one-way ANOVA, respectively, followed by two-sided Tukey's multiple-comparisons test. Data in **(g-i)** were analyzed using two-way repeated-measures ANOVA followed by two-sided Sidak's multiple-comparisons test. ANOVA statistics are provided in Supplementary Table 4, and adjusted P values are shown in the figure. Scale bars are indicated in the images. Source data are provided as a Source Data file.

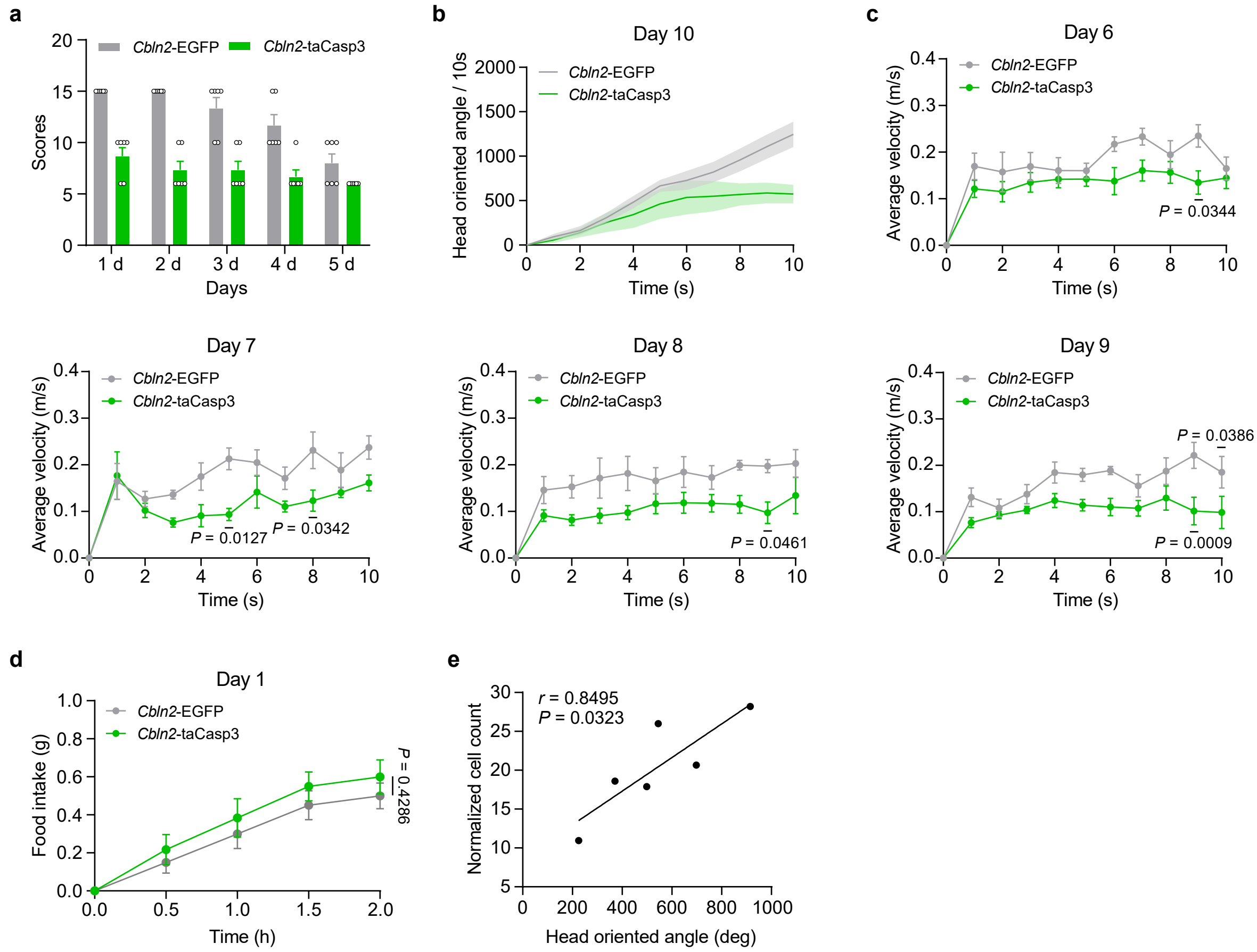

Supplementary Fig. 4 Effects of Cbln2⁺ MVN neuron ablation on rotation-induced MS-like behavior.
(a) Behavioral scores during the first 10 s post-rotation on days 1-5 in mice with or without Cbln2⁺ MVN neuron ablation (*n* = 6 mice per group). **(b)** Cumulative head-orientation angles during the first 10 s of the post-rotation open-field test on day 10 (*n* = 6 mice per group). **(c)** Average velocity during the first 10 s of the post-rotation open-field test on days 6-9 in mice with or without Cbln2⁺ MVN neuron ablation (*n* = 6 mice per group). **(d)** Cumulative food intake in food-deprived mice on day 1 after rotation with or without Cbln2⁺ MVN neuron ablation (*n* = 6 mice per group). **(e)** Association between the number of surviving MVN neurons and cumulative head-orientation angles on day 10 (*n* = 6 mice; two-sided Pearson correlation, *r* = 0.8495, 95% CI 0.1222-0.9832, *P* = 0.0323). Data are mean ± s.e.m.; error bars and shaded areas indicate s.e.m. Data in **(c)** were analyzed using two-way repeated-measures ANOVA followed by two-sided Sidak's multiple-comparisons test. Data in **(d)** were analyzed using two-sided Mann-Whitney *U* tests. ANOVA statistics are provided in Supplementary Table 4, and adjusted and exact *P* values are shown in the figure. Source data are provided as a Source Data file.

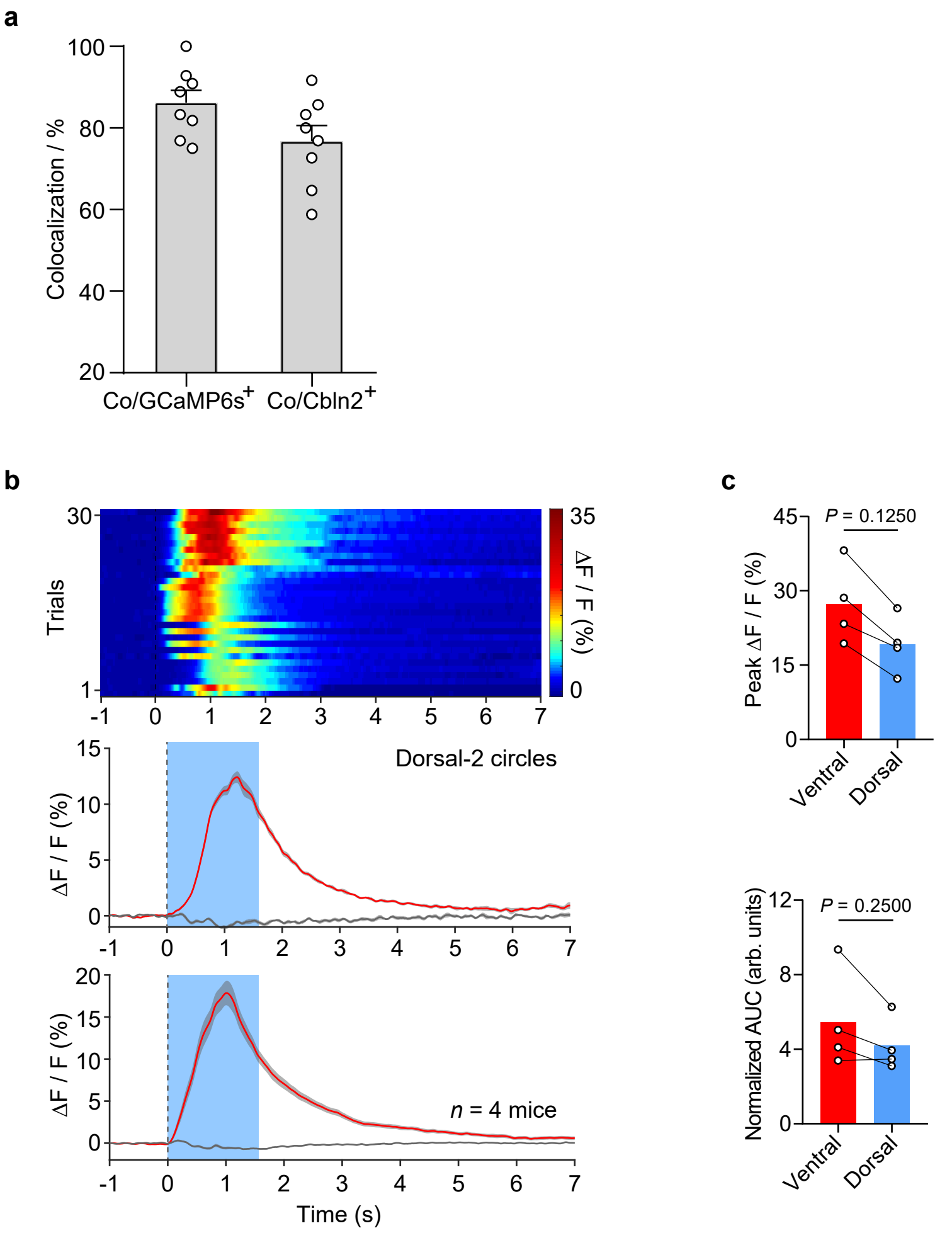

Supplementary Fig. 5 GCaMP6s responses of Cbln2⁺ MVN neurons to ventral and dorsal rotation. **(a)** Quantification of the specificity and efficiency of GCaMP6s labelling of *Cbln2* mRNA⁺ MVN neurons in *Cbln2*-IRES-Cre mice (*n* = 8 fields from 4 mice). Co/GCaMP6s⁺ denotes the percentage of GCaMP6s⁺ neurons positive for *Cbln2* mRNA, whereas Co/Cbln2⁺ denotes the percentage of *Cbln2* mRNA⁺ neurons expressing GCaMP6s. **(b)** Heatmap of normalized GCaMP fluorescence changes (ΔF/F) across 30 trials from 4 mice, aligned to dorsal rotation onset (5 g, 2 cycles) (top); normalized GCaMP signal changes in Cbln2⁺ MVN neurons during MS-like behavior induced by dorsal rotation in individual mice (middle); mean Ca2⁺ transients for the entire test group (*n* = 4 mice) (bottom). Red and gray traces indicate GCaMP6s and EGFP control signals, respectively; thick traces and shaded regions denote mean ± s.e.m. Dashed lines mark rotation onset, and blue shading indicates the rotation period. **(c)** Peak amplitude and time-normalized AUC of GCaMP6s responses in Cbln2⁺ MVN neurons following ventral or dorsal rotation (*n* = 4 mice; two-sided Wilcoxon signed-rank tests). Data are mean ± s.e.m.; error bars and shaded areas indicate s.e.m. Exact *P* values are shown in the graphs. Source data are provided as a Source Data file.

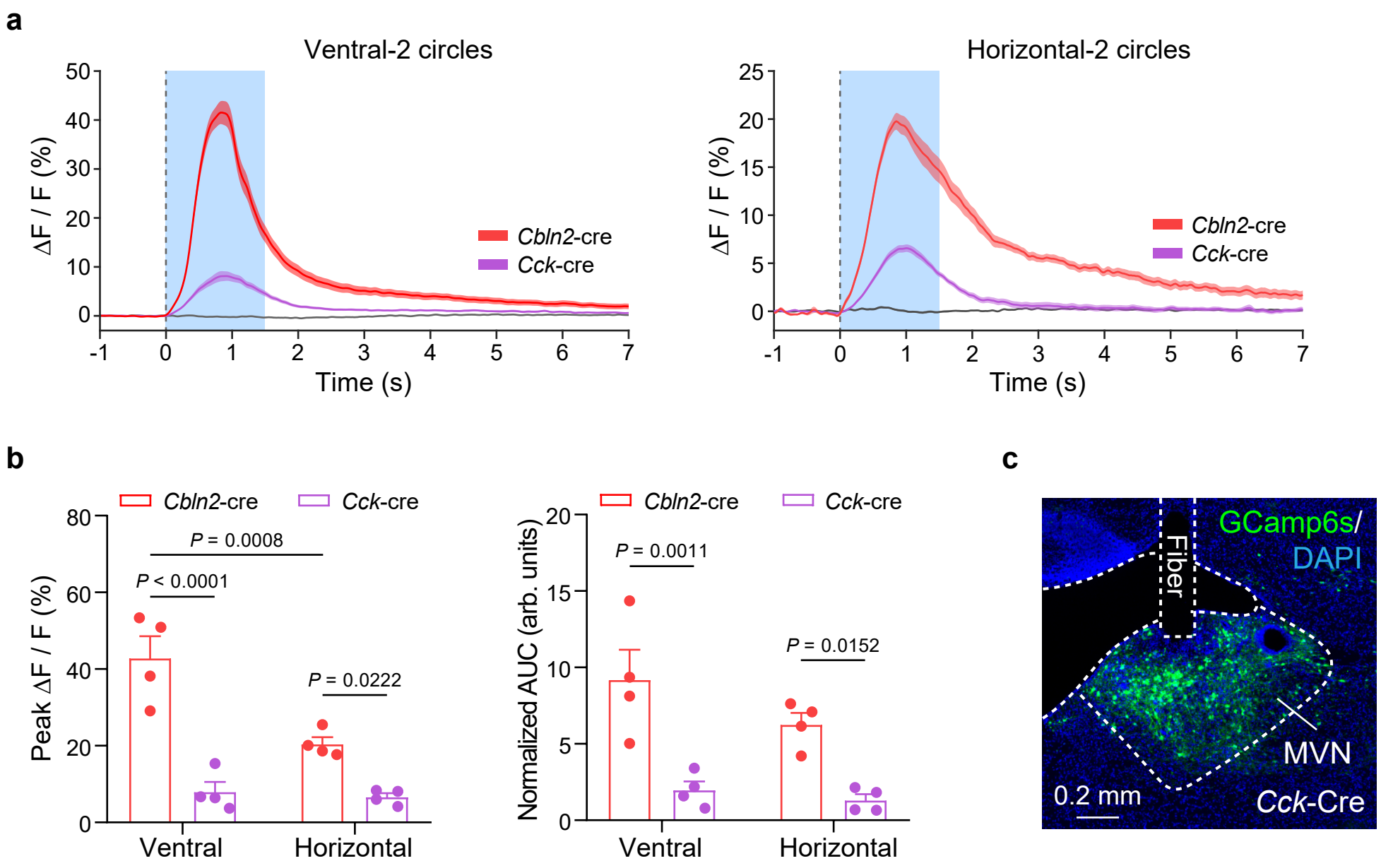

Supplementary Fig. 6 GCaMP6s responses of Cbln2⁺ and Cck⁺ MVN neurons to ventral and horizontal rotation.

(a) Rotation-aligned GCaMP6s activity in Cbln2⁺ and Cck⁺ MVN neurons during ventral (left) and horizontal (right) rotation (*n* = 4 mice per neuronal population). Red and purple traces indicate GCaMP6s responses in Cbln2⁺ and Cck⁺ MVN neurons, respectively. Gray traces indicate EGFP control signals. Thick traces and shaded regions denote mean ± s.e.m. Dashed lines mark rotation onset, and blue shading indicates the rotation period. **(b)** Quantitation of peak amplitude (left) and time-normalized AUC (right) of GCaMP6s responses to ventral or horizontal rotation in Cbln2⁺ and Cck⁺ MVN neurons (*n* = 4 mice per neuronal population). **(c)** Schematic of the injection of AAV-DIO-GCaMP6s into the MVN of *Cck*-IRES-Cre mice, followed by optical fiber implantation above the MVN. Data are mean ± s.e.m.; error bars and shaded areas indicate s.e.m. Data in **(b)** were analyzed using two-way ANOVA followed by two-sided Sidak's multiple-comparisons test. ANOVA statistics are provided in Supplementary Table 4, and adjusted *P* values are shown in the figure. Scale bars are indicated in the images. Source data are provided as a Source Data file.

a

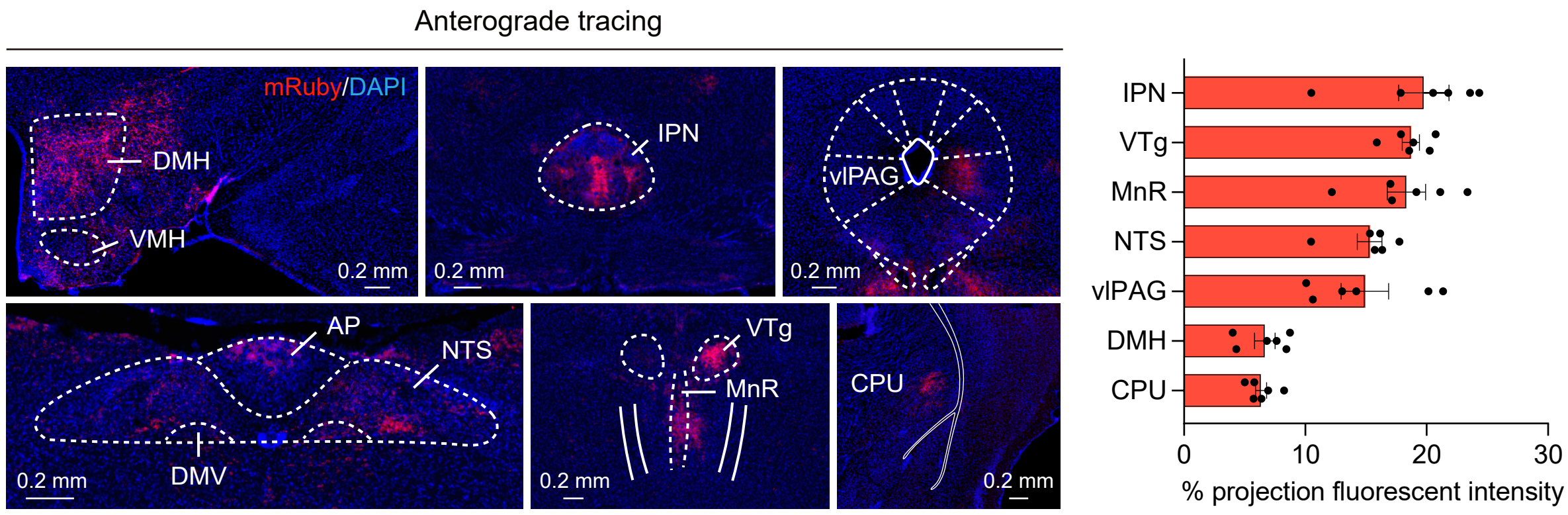

b

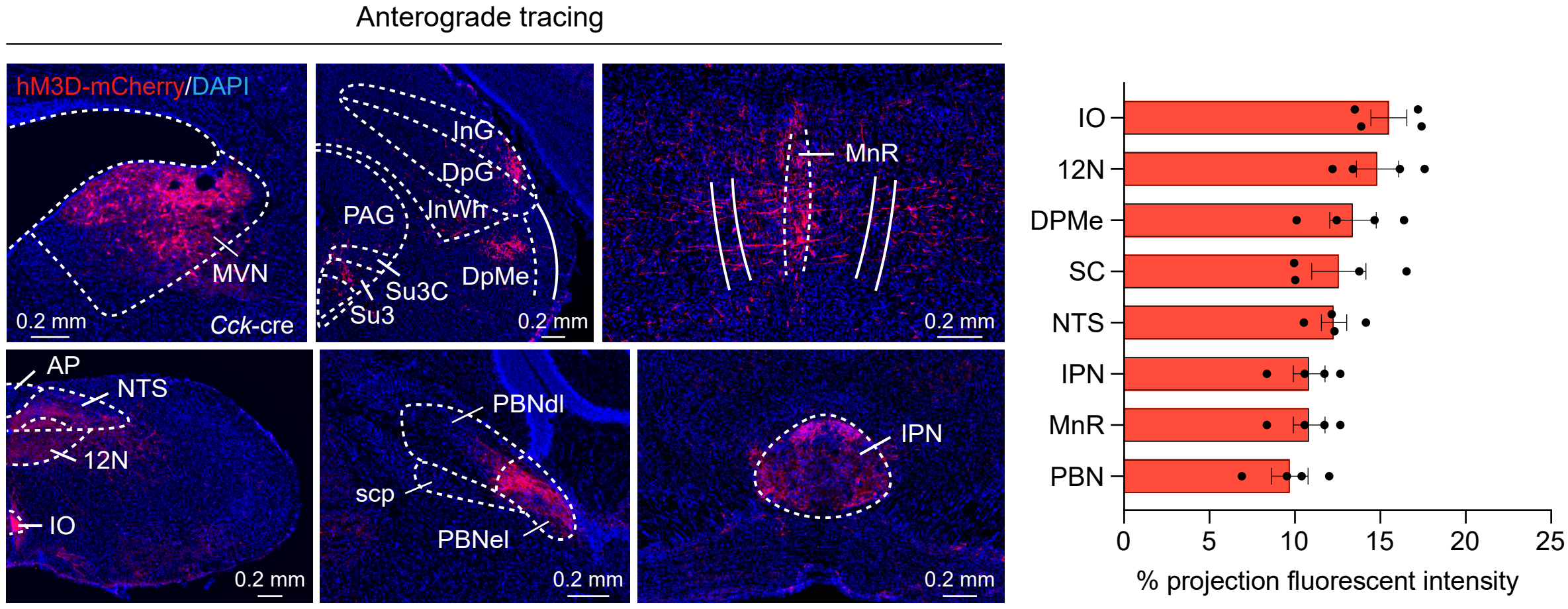

Supplementary Fig. 7 Anterograde projections of Cbln2⁺ and Cck⁺ MVN neurons.

(a) Representative coronal sections showing mRuby-labelled axonal projections of Cbln2⁺ MVN neurons in the indicated brain regions (left), and regional fractions of total projection fluorescence (right; *n* = 6 mice). Fractions were calculated as the fluorescence intensity in each region divided by the summed intensity across all quantified regions. **(b)** Representative coronal sections showing mCherry expression in the MVN and mCherry-labelled axonal projections of Cck⁺ MVN neurons in the indicated brain regions (left), and regional fractions of total projection fluorescence (right; *n* = 4 mice), calculated as in **(a)**. Data are mean ± s.e.m.; error bars indicate s.e.m. Scale bars are indicated in the images. Source data are provided as a Source Data file.

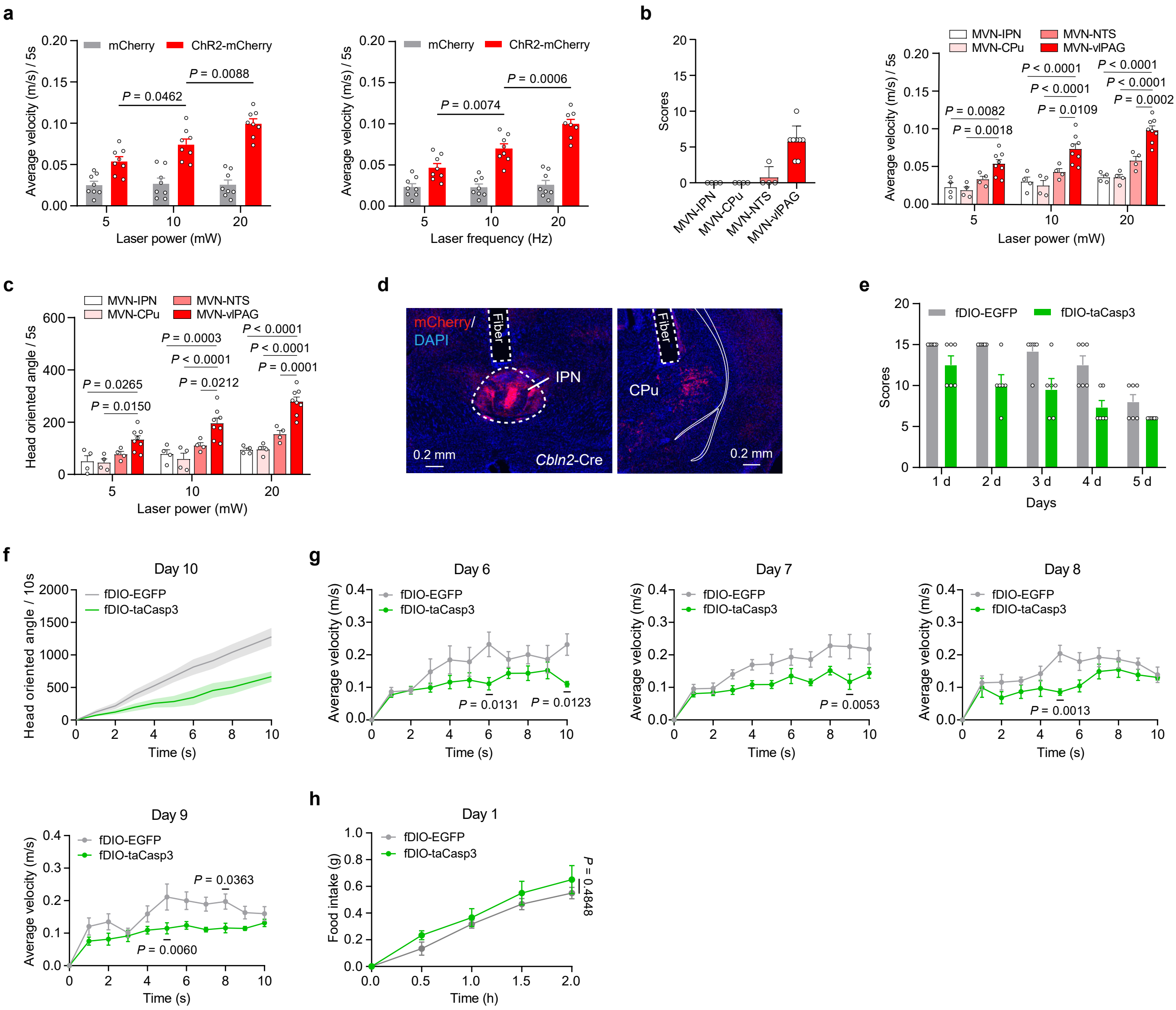

Supplementary Fig. 8 Behavioral effects of activating distinct Cbln2⁺ MVN projections and ablating the MVN–vlPAG pathway. **(a)** Average velocity during optogenetic activation of the Cbln2⁺ MVN-vlPAG pathway at different laser powers (5-20 mW; 20 Hz) and laser frequency (5-20 Hz; 20 mW) (*n* = 8 mice per group). **(b-c)** Behavioral evaluation scores, average velocity **(b)**, and cumulative head orientation angles **(c)** during 5 s optogenetic activation of Cbln2⁺ MVN projections to IPN, CPu, NTS or vlPAG (MVN-IPN, MVN-CPu, and MVN-NTS: *n* = 4 mice per group, MVN-vlPAG: *n* = 8 mice). **(d)** Example coronal brain section showing the optical fiber track above ChR2-mCherry⁺ axonal projections of Cbln2⁺ MVN neurons in the IPN and CPu. **(e)** Behavioral scores during the first 10 s post-rotation on days 1-5 in mice with or without ablation of vlPAG-projecting Cbln2⁺ MVN neurons (*n* = 6 mice per group). **(f)** Cumulative head-orientation angles during the first 10 s of the post-rotation open-field test on day 10 (*n* = 6 mice per group). **(g)** Average velocity during the first 10 s of the post-rotation open-field test on days 6-9 in mice with or without ablation of vlPAG-projecting Cbln2⁺ MVN neurons (*n* = 6 mice per group). **(h)** Cumulative food intake in hungry mice on day 1 following rotation with or without ablation of vlPAG-projecting Cbln2⁺ MVN neurons (*n* = 6 mice per group). Data are mean ± s.e.m.; error bars and shading indicate s.e.m. Data in **(a)** and **(b)** were analyzed using two-way ANOVA followed by two-sided Tukey's and Sidak's multiple-comparisons test, respectively. Data in **(g)** were analyzed using two-way repeated-measures ANOVA followed by two-sided Sidak's multiple-comparisons test. Data in **(h)** were analyzed using two-sided Mann-Whitney *U* test. ANOVA statistics are provided in Supplementary Table 4, and adjusted and exact *P* values are shown in the figure. Scale bars are indicated in the images. Source data are provided as a Source Data file.

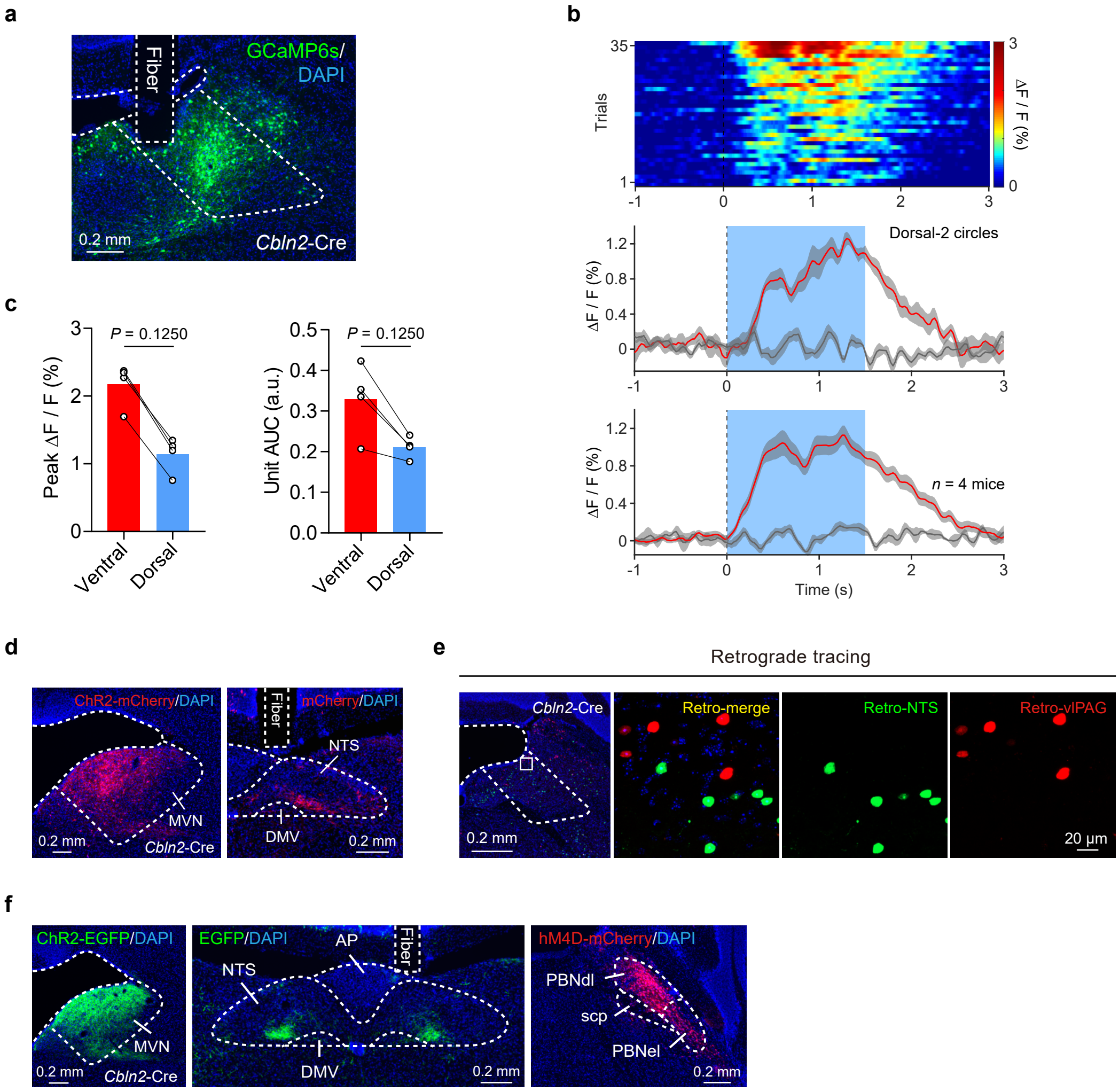

Supplementary Fig. 9 Rotation-evoked activity of vlPAG-projecting Cbln2⁺ MVN neurons and targeting of the MVN-NTS-PBN circuit.

(a) Representative coronal section showing GCaMP6s expression in vlPAG-projecting Cbln2⁺ MVN neurons and the optical-fiber track above the MVN. **(b)** Heatmap of normalized GCaMP fluorescence changes (ΔF/F) across 35 trials from 4 mice, aligned to dorsal rotation onset (5 g, 2 circles) (top); normalized GCaMP signal changes in vlPAG-projecting Cbln2⁺ MVN neurons during dorsal rotation in individual mice (middle); mean $Ca2^+$ transients for the entire test group (*n* = 4 mice) (bottom). Red and gray traces indicate GCaMP6s and EGFP control signals, respectively. Thick traces and shaded regions denote mean ± s.e.m. Dashed lines mark rotation onset, and blue shading indicates the rotation period. **(c)** Quantitation of peak amplitude (left) and unit AUC (right) of GCaMP responses in vlPAG-projecting Cbln2⁺ MVN neurons following ventral or dorsal rotation (*n* = 4 mice; two-side Wilcoxon signed-rank test). **(d)** Representative coronal sections showing ChR2-mCherry expression in Cbln2⁺ MVN neurons and the optical-fiber track above ChR2-mCherry-labelled axonal projections in the NTS. **(e)** Example coronal brain section and magnified fields showing the distribution of NTS-projecting (EGFP⁺) and vlPAG-projecting (mCherry⁺) Cbln2⁺ neurons in the MVN. **(f)** Example coronal brain section showing ChR2-EGFP expression in Cbln2⁺ MVN neurons (left), the optical fiber track above ChR2-EGFP-labelled axonal projections in the NTS (middle), and hM4D-mCherry expression in the PBN (right). Data are mean ± s.e.m.; error bars and shading indicate s.e.m. Exact *P* values are shown in the graphs. Scale bars are indicated in the images. Source data are provided as a Source Data file.

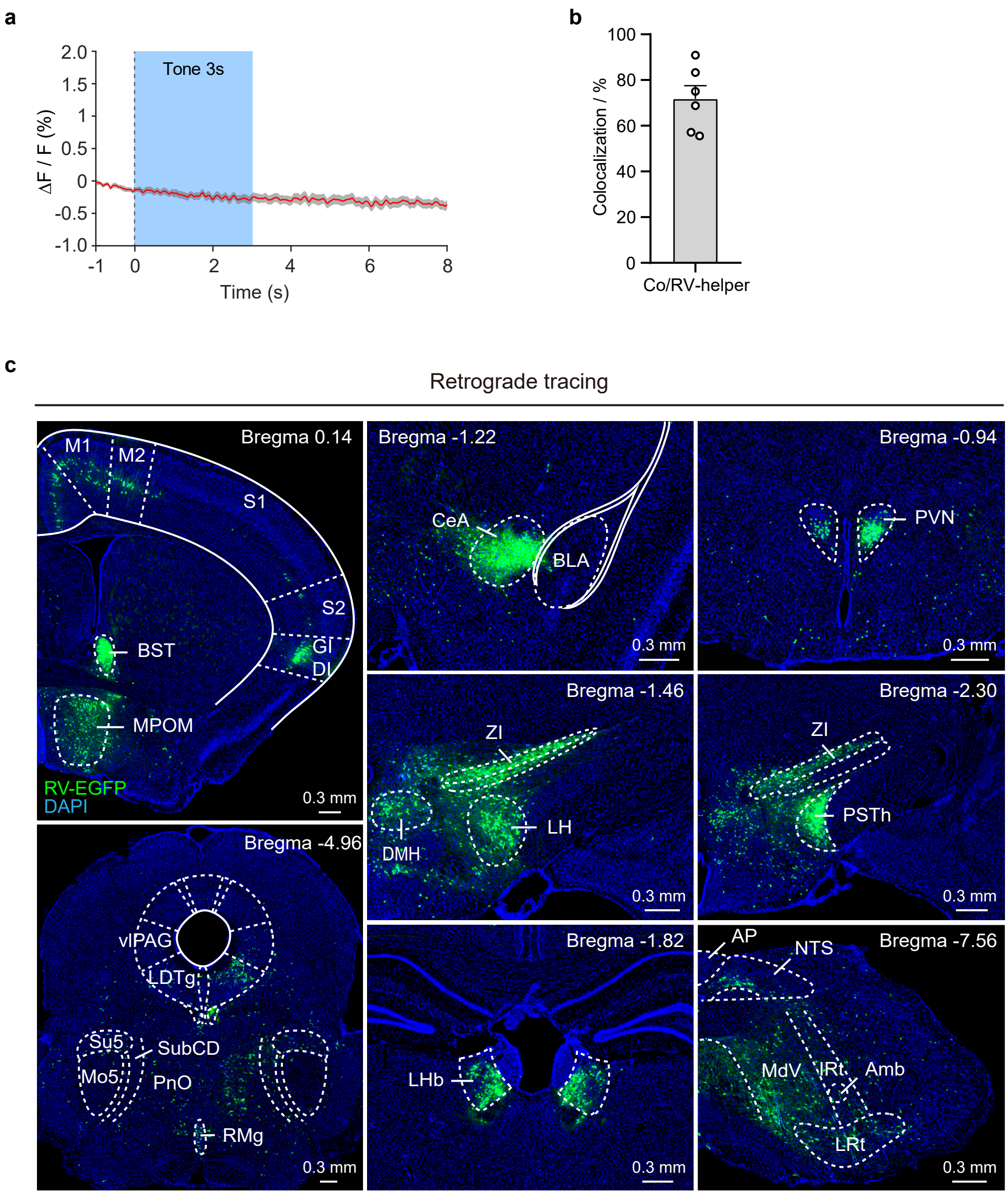

Supplementary Fig. 10 Tone-evoked activity and monosynaptic inputs to Cbln2⁺ MVN neurons.

(a) Tone-aligned GCaMP6s activity in Cbln2⁺ MVN neurons in the absence of rotation (*n* = 5 mice). Thick traces and shaded regions denote mean ± s.e.m. Dashed lines mark tone onset, and blue shading indicates the tone period. **(b)** Proportion of helper-virus-labelled MVN neurons that were also RV-labelled, representing starter cells (*n* = 6 mice). **(c)** Representative micrographs showing monosynaptically RV-labelled input neurons in eight indicated brain regions; similar labelling patterns were observed in 6 biologically independent mice. M1: primary motor cortex; M2: secondary motor cortex; S1: primary somatosensory cortex; S2: secondary somatosensory cortex; GI: granular insular cortex; DI: dysgranular insular cortex; BST: Bed nuclei of the stria terminalis; MPOM: medial preoptic nucleus, medial part; CeA: Central amygdalar nucleus; BLA: Basolateral amygdalar nucleus; PVN: Paraventricular hypothalamic nucleus; ZI: zona incerta; DMH: dorsomedial hypothalamic nucleus; LH: lateral hypothalamic area; PSTh: parasubthalamic nucleus; LDTg: laterodorsal tegmental nucleus; PnO: pontine reticular nucleus, oral part; RMg: raphe magnus nucleus; LHb: lateral habenular nucleus; NTS: Nucleus of the solitary tract; IRt: intermediate reticular nucleus; MdV: medullary reticular nucleus, ventral part; LRt: lateral reticular nucleus. Data are mean ± s.e.m.; error bars and shaded areas indicate s.e.m. Scale bars are indicated in the images. Source data are provided as a Source Data file.

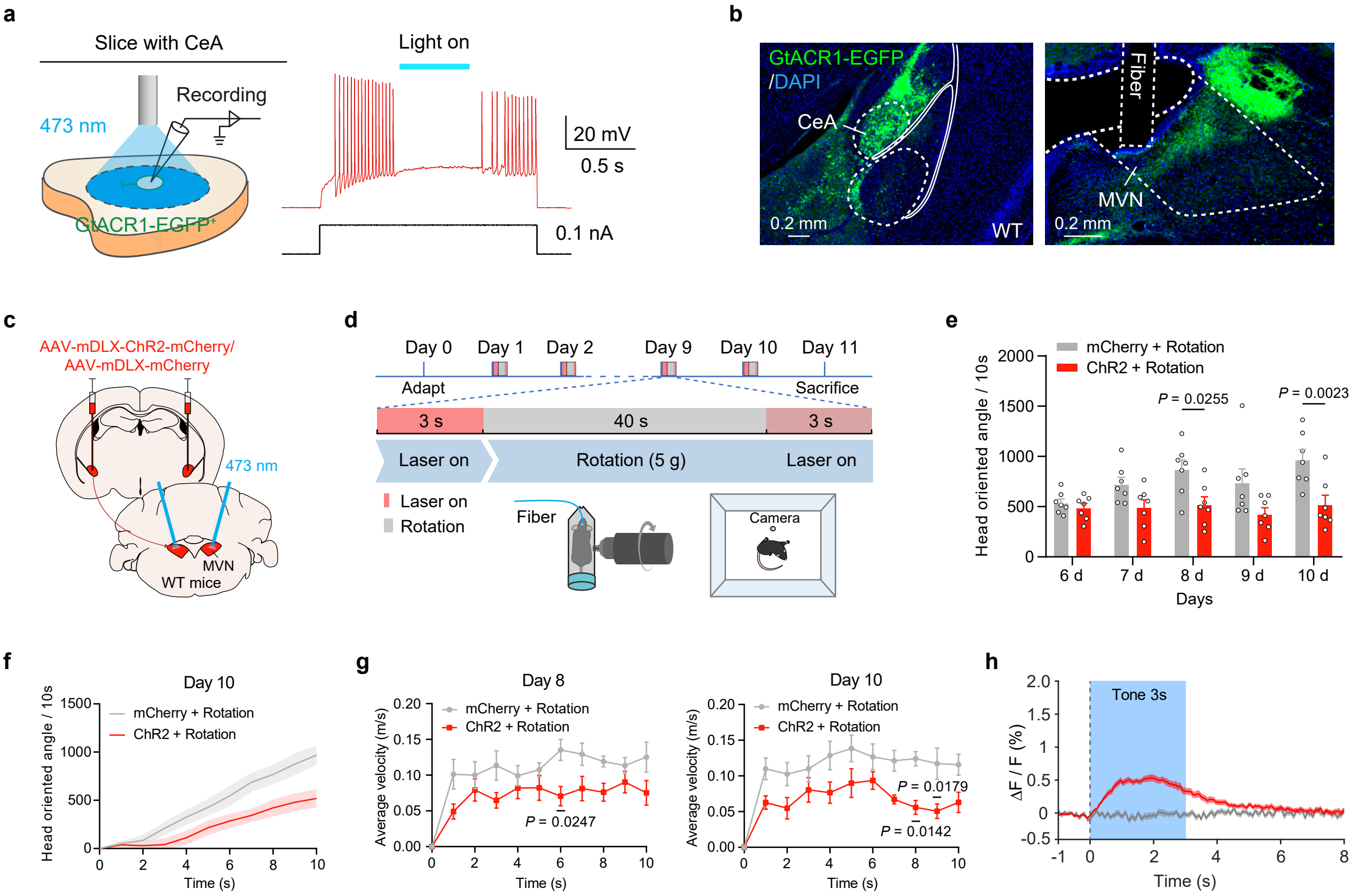

Supplementary Fig. 11 Optogenetic inhibition of the CeA-MVN inhibitory pathway during tone-paired repeated rotation.
(a) Whole-cell current-clamp recording from GtACR1-EGFP⁺ neurons in acute slice with CeA (left); an example trace showing suppression of action-potential firing by light stimulation of GtACR1-expressing CeA neurons (right). **(b)** Example coronal brain section showing the expression of GtACR1-EGFP in the CeA (left) and the optical fiber track above GtACR1-EGFP⁺ axonal projections of CeA neurons in the MVN (right). **(c)** Virus injection and optical fiber implantation strategy for optogenetic inhibition of CeA-MVN projections in WT mice. **(d)** Experimental timeline. Mice underwent 10 days of repeated rotation with anticipatory tone pairing and concurrent optogenetic inhibition (10 ms, 10 mW) of CeA-MVN projections during each rotation session. **(e, f)** Cumulative head-orientation angles during the first 10 s of the post-rotation open-field test on days 6-10 (n = 7 mice per group). **(g)** Average velocity during the first 10 s of the post-rotation open-field test on days 8 and 10 (n = 7 mice per group). **(h)** Tone-aligned activity of Vgat⁺ CeA-MVN projection neurons in the absence of rotation (n = 4 mice). Thick traces and shaded regions denote mean ± s.e.m.; dashed lines mark tone onset. Blue shading indicates the tone period. Data are mean ± s.e.m.; error bars and shaded areas indicate s.e.m. Data in **(e)** and **(g)** were analyzed using two-way ANOVA followed by two-sided Sidak's multiple-comparisons test. ANOVA statistics are provided in Supplementary Table 4, and adjusted P values are shown in the figure. Scale bars are indicated in the images. Source data are provided as a Source Data file.

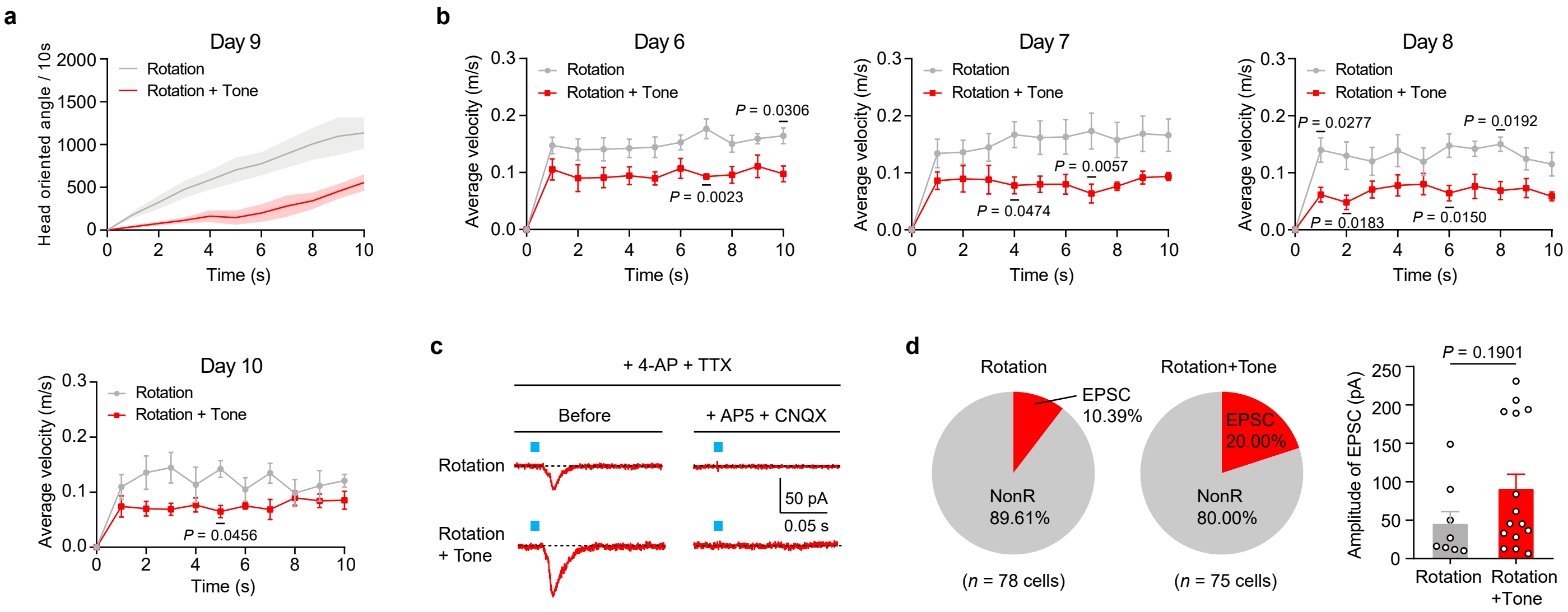

Supplementary Fig. 12 Activation of the CeA-MVN^{Cbln2} inhibitory pathway attenuates rotation-induced MS-like behavior. **(a)** Cumulative head-orientation angles during the first 10 s of the post-rotation open-field test on day 9 (*n* = 6 mice per group). **(b)** Average velocity during the first 10 s of the post-rotation open-field test on days 6-8 and day 10 following repeated rotation with or without tone pairing (*n* = 6 mice per group). **(c)** Schematic diagram showing recording of light-evoked EPSCs from ChR2-mCherry$^-$ and EGFP$^+$ MVN neurons during photostimulation of CeA terminals (left). Responses abolished by bath application of AP5 and CNQX (right). **(d)** Quantitative analyses showing fractions and amplitude of light-evoked EPSCs recorded from ChR2-mCherry$^-$ and EGFP$^+$ MVN neurons in the Rotation group (*n* = 78 cells from 5 mice) and Rotation+Tone group (*n* = 75 cells from 5 mice). Data are mean ± s.e.m.; error bars and shading indicate s.e.m. Data in **(b)** were analyzed using two-way ANOVA followed by two-sided Sidak's multiple-comparisons test. Data in **(d)** were analyzed using two-sided Mann-Whitney *U* test. ANOVA statistics are provided in Supplementary Table 4, and adjusted and exact *P* values are shown in the figure. Source data are provided as a Source Data file.

Supplementary Table 1 Information on mice and reagents

**Mouse Lines**		
Vglut2-IRES-Cre	JAX Mice	Stock No: 016963
Vgat-IRES-Cre	JAX Mice	Stock No: 028862
Cbln2-IRES-Cre	JAX Mice	Stock No: 032949
Crh-IRES-Cre	JAX Mice	Stock No: 012704
Chat-IRES-Cre	JAX Mice	Stock No: 006410
Cck-IRES-Cre	JAX Mice	Stock No: 012706
**Antibodies**		
Rabbit polyclonal anti-EGFP	Abcam	ab290
Chicken polyclonal anti-EGFP	Abcam	ab13970
Chicken polyclonal anti-mCherry	Abcam	ab205402
Rabbit monoclonal Anti-Fos	Cell Signaling Technology	2250
Rabbit monoclonal Anti-NeuN	Abcam	AB177487
Goat anti-Rabbit IgG (H+L) Alexa 488	Thermo Fisher Scientific	A11034
Goat anti-Chicken IgG (H+L) Alexa 488	Thermo Fisher Scientific	A11001
Goat anti-Rabbit IgG (H+L) Alexa 546	Thermo Fisher Scientific	A11010
Goat anti-Chicken IgY (H+L) Alexa 546	Thermo Fisher Scientific	A11040
Goat anti-Mouse IgG (H+L) Alexa 546	Thermo Fisher Scientific	A11030
**Chemicals, peptides, and recombinant proteins**		
D-AP5	Tocris	0106
CNQX	Tocris	0190
Picrotoxin	Tocris	1128
TTX	Tocris	1078

4-AP	Tocris	0940
DAPI	Solarbio	C0065
Clozapine N-oxide	ENZO	BML-NS105
Co-Detection Antibody Diluent	ACD	323160
Co-Detection Target Retrieval solution	ACD	323166
RNAscope Probe-Mm-Cbln2	ACD	428551
**Bacterial and viral strains**		
AAV2/9-hSyn-DIO-hM3Dq-mCherry	TaiToll	N/A
AAV2/9-hEF1a-DIO-hChR2-mCherry	TaiToll	N/A
AAV2/9-EF1a-DIO-hChR2-EGFP	BrainVTA	N/A
AAV2/9-hSyn-DIO-mCherry	TaiToll	N/A
AAV2/9-EF1a-DIO-EGFP	BrainVTA	N/A
AAV2/9-EF1a-Flex-taCasp3	BrainVTA	N/A
AAV2/9-EF1a-DIO-mCherry	BrainVTA	N/A
AAV2/9-EF1a-DIO-RV-G	BrainVTA	N/A
RV-EnvA-DG-EGFP	BrainVTA	N/A
rAAV-EF1α-DIO-H2B-EGFP	BrainVTA	N/A
AAV-hSyn-DIO-H2B-EGFP-Synaptophysin-mRuby	BrainVTA	N/A
AAV2/9-CAG-DIO-GCaMp6s	BrainVTA	N/A
AAV2/retro-EF1a-DIO-flp	BrainVTA	N/A
AAV2/retro-EF1a-DIO-H2B-EGFP	BrainVTA	N/A
AAV2/retro-EF1a-DIO-H2B-mCherry	BrainVTA	N/A
AAV2/2-EF1a-fDIO-taCasp3	BrainVTA	N/A
AAV2/2-EF1a-fDIO-EGFP	BrainVTA	N/A

AAV2/2-EF1a-fDIO-GCaMp6s	BrainVTA	N/A
AAV2/9-mCaMKIIa-hM4Di-mCherry	TaiToll	N/A
AAV2/9-mCaMKIIa-mCherry	TaiToll	N/A
AAV2/9-mDlx-hChR2-mCherry	TaiToll	N/A
AAV2/9-mDlx-mCherry	TaiToll	N/A
AAV2/9-mDlx-GtACR1-EGFP	TaiToll	N/A
AAV2/9-mDlx-EGFP	TaiToll	N/A
AAV2/9-hSyn-hChR2-mCherry	TaiToll	N/A

Supplementary Table 2 Summary of all experimental designs

Figures	Aims	Mouse lines	AAV injection & optical fiber implantation	Type of data
Fig.1a-1m Fig.S1a	Quantitative analysis of motion sickness induced by rotation	*WT*	No AAV injection. No optical fiber implantation	Behavior
Fig.1n Fig.S1b-2c	Identify nuclei associated with MS-like behavior	*WT*	No AAV injection. No optical fiber implantation	Behavior Histology
Fig.2a-2h Fig.S2a-3c	Quantitative analysis of anticipatory tone interventions for MS-like behavior	*WT*	No AAV injection. No optical fiber implantation	Behavior
Fig. 3a-3l Fig.S3a-3l	Activation of MVN neuron subtypes	*Cbln2*-IRES-Cre *Crh*-IRES-Cre *Chat*-IRES-Cre *Cbln2*-IRES-Cre *Vglut2*-IRES-Cre *Cck*-IRES-Cre	AAV-DIO-hM3D(Gq)-mCherry or AAV-DIO-mCherry injected into the MVN; AAV2/9-DIO-hChR2-mCherry, or AAV-DIO-mCherry injected into the MVN, optical fiber implanted above the MVN	Behavior Histology Slice physiology
Fig.3m-3s Fig.S4a-4e	Ablation of Cbln2 neurons of MVN	*Cbln2*-IRES-Cre	AAV2/9-EF1a-Flex-taCasp3 or AAV2/9-EF1a-DIO-EGFP injected into the MVN	Behavior Histology
Fig.4a-4c	Colocalization of Vglut2⁺ or Vgat⁺ neurons and *Cbln2* mRNA;	*Vglut2*-IRES-Cre *Vgat*-IRES-Cre	AAV-DIO-H2B-EGFP injected into the MVN	Histology
Fig.4d	Cbln2⁺ MVN neurons' electrophysiological properties	*Cbln2*-IRES-Cre	AAV-Ef1α-DIO-EGFP injected into the MVN	Slice physiology
Fig.4e-4j Fig.S5 Fig.S6	Recording Cbln2⁺ or Cck⁺ neuronal activity in rotation-induced MS-like behavior	*Cbln2*-IRES-Cre *Cck*-IRES-Cre	AAV2/9-CAG-DIO-GCaMp6s or AAV2/9-EF1a-fDIO-EGFP injected into the MVN and optical fiber implanted above the MVN	Behavior Fiber photometry
Fig.5a-5b Fig.S7	Anterograde tracing downstream pathway of Cbln2⁺ MVN neurons. Anterograde tracing downstream pathways of Cck⁺ MVN neurons.	*Cbln2*-IRES-Cre *Cck*-IRES-Cre	AAV-hSyn-DIO-H2B-EGFP-Synaptophysin-mRuby injected into the MVN in *Cbln2*-IRES-Cre; AAV-hSyn-DIO-hM3D-mCherry injected into the MVN in *Cck*-IRES-Cre;	Histology

Fig.5c-h Fig.S8a-8d	Activation of MVN-vlPAG, -IPN, -NTS, and -CPU pathways	*Cbln2*-IRES-Cre	AAV2/9-hEF1a-DIO-hChR2-mCherry or AV2/9-hEF1a-DIO-mCherry injected into the MVN, optical fiber implanted above the vlPAG, IPN, NTS, or CPU	Histology Behavior Slice physiology
Fig.5i-5o Fig. S8e-8h	Ablation of the MVN-vlPAG pathway	*Cbln2*-IRES-Cre	AAV2/Retro-DIO-flp injected into the vlPAG, AAV2/9-EF1a-fDIO-taCasp3 or AAV2/9-EF1a-fDIO-EGFP injected into the MVN	Behavior Histology
Fig.6a-6d Fig.S9a-9c	Recording Cbln2⁺ neuronal activity in the MVN-vlPAG pathway in rotation-induced MS-like behavior	*Cbln2*-IRES-Cre	AAV2/Retro-DIO-flp injected into the vlPAG, AAV2/9-EF1a-fDIO-GCaMp6s or AAV2/9-EF1a-fDIO-EGFP injected into the MVN and optical fiber implanted above the MVN	Behavior Slice physiology
Fig.6e-6l Fig.S9d-9f	Activation of the MVN-NTS pathway and inhibition of PBN excitatory neurons. Activation of the MVN-vlPAG pathway	*Cbln2*-IRES-Cre	AAV2/9-hEF1a-DIO-hChR2-mCherry or AV2/9-hEF1a-DIO-mCherry injected into the MVN, AAV2/9-mCaMKIIa-hM4Di-mCherry or AAV2/9-mCaMKIIa-mCherry injected into the PBN, optical fiber implanted above the NTS	Behavior Histology
Fig.7a-7e Fig.S10a	Recording Cbln2⁺ neuronal activity in tone interventions for MS-like behavior	*Cbln2*-IRES-Cre	AAV2/9-CAG-DIO-GCaMp6s injected into the MVN and optical fiber implanted above the MVN	Behavior Fiber photometry
Fig.7f-7h Fig.S10b-11c	RV tracing of projecting Cbln2⁺ MVN neurons	*Cbln2*-IRES-Cre	AAV-DIO-mCherry-T2A-TVA and AAV-DIO-RVG in MVN, RV-EvnA-G-EGFP in MVN (Injection strategy see Fig. 7e)	Histology
Fig.8a-o Fig.S11 Fig.S12	Activation and calcium imaging of the CeA-MVN pathway in rotation-induced MS-like behavior	*WT* *Cbln2*-IRES-Cre *Vgat*-IRES-Cre	AAV2/9-mDlx-GtACR1-EGFP or AAV2/9-mDlx-EGFP in CeA, and optical fiber implanted above the MVN; Retro-DIO-flp in MVN, AV2/9-fDIO-GCamP6s in CeA, and optical fiber implanted above the CeA; AAV2/9-hSyn-hChR2-mCherry in CeA and AAV2/9-EF1a-DIO-EGFP in MVN	Behavior Slice physiology Histology

Supplementary Table 3 Summary of Cell Counting Strategy

Brain region	MVN	CeA	The whole brain
Section type	Coronal section (12 or 40 μm)	Coronal section (40 μm)	Coronal section (40 μm)
Section Range	Bregma (-5.88 to -6.24)	Bregma (-1.22 to -1.70)	The whole brain
Total collection	Approximately 30 or 9 sections	Approximately 12 sections	Approximately 300 sections
Sampling	To sample 1 section every 5 sections to get 10 or 6 sections evenly spaced by 60 or 200 μm	To sample 1 section every 5 sections to get 8 sections evenly spaced by 200 μm	To sample 1 section every 5 sections to get 50 sections evenly spaced by the whole brain

Supplementary Table 4. Summary of statistical analyses

Figure	Sample size（*n*）	Statistical test	*P* values
1d	Control: *n* = 6 mice<br>Rotation: *n* = 6 mice	Mann-Whitney *U* test	Rotation vs. Control: *P* = 0.0022 **
1e	Control: *n* = 6 mice<br>Rotation: *n* = 6 mice	Mann-Whitney *U* test	Rotation vs. Control: *P* = 0.0022 **
1f	Control: *n* = 6 mice<br>Rotation: *n* = 6 mice	Mann-Whitney *U* test	Rotation vs. Control: *P* = 0.0022 **
1h	Control: *n* = 5 mice<br>Rotation: *n* = 6 mice	Mann-Whitney *U* test	6 d vs. Control: *P* = 0.0022 **
1j	Control: *n* = 6 mice<br>Rotation: *n* = 6 mice	Mann-Whitney *U* test	Rotation vs. Control: *P* = 0.0022 **
1l	Control: *n* = 6 mice<br>Rotation: *n* = 6 mice	Mann-Whitney *U* test	Rotation vs. Control: *P* = 0.0152 *
1m	Control: *n* = 6 mice<br>Rotation: *n* = 6 mice	Mann-Whitney *U* test	Rotation vs. Control: *P* = 0.0022 **
1n	Control: *n* = 4 mice<br>Rotation: *n* = 6 mice	Mann-Whitney *U* test	Rotation vs. Control: *P* = 0.0095 **
2c	Rotation: *n* = 8 mice<br>Rotation + Fixed tone: *n* = 8 mice<br>Rotation + Random tone: *n* = 8 mice	Two-way ANOVA	Head orientation angle / 10 s:<br>7 d: Rotation vs. Rotation + Fixed tone: *P* = 0.0092 **<br>8 d: Rotation vs. Rotation + Fixed tone: *P* = 0.0175 *<br>Rotation vs. Rotation + Random tone: *P* = 0.0079 **<br>9 d: Rotation vs. Rotation + Fixed tone: *P* = 0.0116 *<br>Rotation vs. Rotation + Random tone: *P* = 0.0355 *<br>10 d: Rotation vs. Rotation + Fixed tone: *P* = 0.0050 **<br>11 d: Rotation vs. Rotation + Fixed tone: *P* = 0.0952 n.s<br>Day effect: *F* (3.689, 77.47) = 5.766, *P* = 0.0006; Group effect: *F* (2, 21) = 18.12, *P* < 0.0001; Interaction: *F* (10, 105) = 0.4626, *P* = 0.9105
2e	Rotation: *n* = 8 mice<br>Rotation + Fixed tone: *n* = 8 mice<br>Rotation + Random tone: *n* = 8 mice	Two-way ANOVA	Average velocity / 10 s:<br>Rotation vs. Rotation + Fixed tone: *P* < 0.0001 ****<br>Rotation + Fixed tone vs. Rotation + Random tone: *P* = 0.0069 **<br>Day effect: *F* (4.821, 101.2) = 12.66, *P* < 0.0001; Group effect: *F* (2, 21) = 5.628, *P* = 0.0110; Interaction: *F* (20, 210) = 0.7093, *P* = 0.8146
2g	Rotation: *n* = 6 mice<br>Rotation + Fixed tone: *n* = 6 mice<br>Rotation + Random tone: *n* = 6 mice	One-way ANOVA	Food intake / 2 h:<br>Rotation vs. Rotation + Fixed tone: *P* = 0.0496 *<br>*F* (2, 18) = 3.516, *P* = 0.0514

2h	Rotation: n = 6 mice<br>Rotation + Fixed tone: n = 6 mice<br>Rotation + Random tone: n = 6 mice	One-way ANOVA	Body temperature / 1 h:<br>Rotation vs. Rotation + Fixed tone: P = 0.0819 n.s<br>F (2, 15) = 2.994, P = 0.0805
3c	*Crh*-IRES-Cre: n = 5 mice<br>*Chat*-IRES-Cre: n = 5 mice<br>*Cck*-IRES-Cre: n = 5 mice<br>*Cbln2*-IRES-Cre: n = 5 mice<br>*Vglut2*-IRES-Cre: n = 5 mice	Two-way ANOVA	Laser power of head orientation angle / 5 s:<br>10 mW: *Cbln2*-IRES-Cre vs. *Vglut2*-IRES-Cre: P = 0.0020 **<br>*Cbln2*-IRES-Cre vs. *Cck*-IRES-Cre: P = 0.0114 *<br>20 mW: *Cbln2*-IRES-Cre vs. *Vglut2*-IRES-Cre: P < 0.0001 ****<br>*Cbln2*-IRES-Cre vs. *Cck*-IRES-Cre: P = 0.0082 **<br>*Cbln2*-IRES-Cre: 10 mW vs. 5 mW: P = 0.0047 **<br>*Cbln2*-IRES-Cre: 20 mW vs. 10 mW: P < 0.0001 ****<br>Power effect: F (2, 60) = 41.08, P < 0.0001; Group effect: F (4, 60) = 69.55, P < 0.0001; Interaction: F (8, 60) = 7.746, P < 0.0001
3d	*Cbln2*-IRES-Cre: n = 8 mice	One-way ANOVA	Laser frequency of head orientation angle / 5 s:<br>10 Hz vs. 5 Hz: P = 0.0061 **; 20 Hz vs.10 Hz: P = 0.0092 **<br>Frequency effect: F (7, 14) = 1.509, P = 0.2424
3h	Saline: Ctrl n = 7 mice, hM3D n = 7 mice;<br>CNO: Ctrl n = 7 mice, hM3D n = 7 mice	Two-way ANOVA	Average velocity / 30 min:<br>Saline: Ctrl vs. hM3D: P = 0.8915 n.s<br>CNO: Ctrl vs. hM3D: P = 0.0041 **<br>Treatment effect: F (1, 12) = 27.41, P = 0.0002; Group effect: F (1, 12) = 6.802, P = 0.0229; Interaction: F (1, 12) = 5.132, P = 0.0428
3j	Saline: Ctrl n=7 mice, hM3D n = 7 mice;<br>CNO: Ctrl n = 7 mice, hM3D n = 7 mice	Two-way ANOVA	Rotation numbers / 30 min:<br>Saline: Ctrl vs. hM3D: P = 0.9943 n.s<br>CNO: Ctrl vs. hM3D: P < 0.0001 ****<br>Treatment effect: F (1, 12) = 45.99, P < 0.0001; Group effect: F (1, 12) = 46.74, P < 0.0001; Interaction: F (1, 12) = 48.85, P < 0.0001
3o	Cbln2-EGFP: n = 6 mice<br>Cbln2-taCasp3: n = 6 mice	Two-way ANOVA	Head orientation angle / 10 s:<br>6 d: Cbln2-taCasp3 vs. Cbln2-EGFP: P = 0.0801 n.s<br>7 d: Cbln2-taCasp3 vs. Cbln2-EGFP: P = 0.0173 *<br>8 d: Cbln2-taCasp3 vs. Cbln2-EGFP: P = 0.0213 *<br>9 d: Cbln2-taCasp3 vs. Cbln2-EGFP: P = 0.0264 *<br>10 d: Cbln2-taCasp3 vs. Cbln2-EGFP: P = 0.0158 *<br>Day effect: F (3.029, 30.29) = 2.874, P = 0.0520; Group effect: F (1, 10) = 38.57, P = 0.0001; Interaction: F (4, 40) = 0.1331, P = 0.9693
3p	Cbln2-EGFP: n = 6 mice<br>Cbln2-taCasp3: n = 6 mice	Two-way ANOVA	Average velocity / 10 s:<br>2 s: Cbln2-taCasp3 vs. Cbln2-EGFP: P = 0.0196 *

			5 s: Cbln2-taCasp3 vs. Cbln2-EGFP: *P* = 0.0277 *<br>7 s: Cbln2-taCasp3 vs. Cbln2-EGFP: *P* = 0.0011 **<br>Time effect: *F* (3.049, 30.49) = 11.63, *P* < 0.0001; Group effect: *F* (1, 10) = 16.57, P = 0.0022; Interaction: *F* (10, 100) = 1.596, *P* = 0.1185
3q	Cbln2-EGFP: *n* = 6 mice<br>Cbln2-taCasp3: *n* = 6 mice	Mann-Whitney *U* test	Food intake / 2 h:<br>Cbln2-taCasp3 vs. Cbln2-EGFP: *P* = 0.0455 *, *U* = 5.500
3s	Cbln2-EGFP: *n* = 6 mice<br>Cbln2-taCasp3: *n* = 6 mice	Mann-Whitney *U* test	NeuN cell count:<br>Cbln2-taCasp3 vs. Cbln2-EGFP: *P* = 0.0022 **, *U* = 0
4i	0.05 g: *n* = 6 mice<br>0.5 g: *n* = 6 mice<br>5.0 g: *n* = 6 mice	One-way ANOVA	Peak ∆F/F (%): 5.0 g vs. 0.5 g: *P* = 0.0113 *<br>5.0 g vs. 0.05 g: *P* = 0.0475 *<br>Unit AUC (a.u.): 5.0 g vs. 0.5 g: *P* = 0.0445 *<br>5.0 g vs. 0.05 g: *P* = 0.0040 **<br>Peak ∆F/F effect: *F* (2, 15) = 6.234, *P* = 0.0107<br>Unit AUC (a.u.) effect: *F* (2, 15) = 7.842, *P* = 0.0047
5e	mCherry: *n* = 8 mice<br>ChR2-mCherry: *n* = 8 mice	Two-way ANOVA	Laser power of head orientation angle / 5 s:<br>10 mW vs. 5 mW: *P* = 0.0397 *<br>20 mW vs. 10 mW: *P* = 0.0039 **<br>Power effect: *F* (2, 42) = 10.08, *P* = 0.0003; Group effect: *F* (1, 42) = 102.0, *P* < 0.0001; Interaction: *F* (2, 42) = 8.308, *P* = 0.0009
5f	mCherry: *n* = 8 mice<br>ChR2-mCherry: *n* = 8 mice	Two-way ANOVA	Laser frequency of head orientation angle / 5 s:<br>10 mW vs. 5 mW: *P* = 0.0138 *<br>20 mW vs. 10 mW: *P* = 0.0001 ***<br>Frequency effect: *F* (2, 42) = 17.36, *P* < 0.0001; Group effect: *F* (1, 42) = 127.1, *P* < 0.0001; Interaction: *F* (2, 42) = 12.10, *P* < 0.0001
5g right	*n* = 10 cells	One-way ANOVA	Amplitude of PSCs:<br>Before vs. PTX: *P* = 0.1108 n.s<br>Before vs. APV+CNQX: *P* = 0.0022 **<br>Amplitude of PSCs effect: *F (*2, 27) = 8.040, *P* = 0.0018
5j	fDIO-EGFP: *n* = 6 mice<br>fDIO-taCasp3: *n* = 6 mice	Two-way ANOVA	Head orientation angle / 10 s:<br>7 d: fDIO-taCasp3 vs. fDIO-EGFP: *P* = 0.0192 *<br>9 d: fDIO-taCasp3 vs. fDIO-EGFP: *P* = 0.0422 *<br>10 d: fDIO-taCasp3 vs. fDIO-EGFP: *P* = 0.0238 *<br>Day effect: *F* (3.142, 31.42) = 0.3859, *P* = 0.7727; Group effect: *F* (1, 10) = 57.58, *P* < 0.0001; Interaction: *F* (4, 40) = 0.09371, *P* = 0.9839
5k	fDIO-EGFP: *n* = 6 mice<br>fDIO-taCasp3: *n* = 6 mice	Two-way ANOVA	Average velocity / 10 s:<br>8 s: fDIO-taCasp3 vs. fDIO-EGFP: *P* = 0.0041 **<br>10 s: fDIO-taCasp3 vs. fDIO-EGFP: *P* = 0.0045 **<br>Time effect: *F* (10, 100) = 20.06, *P* < 0.0001; Group effect: *F* (1, 10) = 10.53, *P* = 0.0088; Interaction: *F* (10, 100) = 1.914, *P* = 0.0518

5l	fDIO-EGFP: *n* = 6 mice<br>fDIO-taCasp3: *n* = 6 mice	Mann-Whitney<br>*U* test	Food intake / 2 h:<br>fDIO-taCasp3 vs. fDIO-EGFP: *P* = 0.0584 n.s, *U* = 6
5n	fDIO-EGFP: *n* = 6 mice<br>fDIO-taCasp3: *n* = 6 mice	Mann-Whitney<br>*U* test	Normalized NeuN cell counts in MVN-vlPAG:<br>fDIO-taCasp3 vs. fDIO-EGFP: *P* = 0.0022 **, *U* = 0
6d	0.05 g: *n* = 6 mice<br>0.5 g: *n* = 6 mice<br>5.0 g: *n* = 6 mice	One-way<br>ANOVA	Peak ∆F/F (%): 5.0 g vs. 0.05 g: *P* = 0.0023 **<br>Unit AUC (a.u.): 5.0 g vs. 0.05 g: *P* = 0.0011 **<br>0.5 g vs. 0.05 g: *P* = 0.0144 *<br>Peak ∆F/F (%) effect: *F* (2, 12) = 9.679, *P* = 0.0031<br>Unit AUC (a.u.) effect: *F* (2, 12) = 12.30, *P* = 0.0012
6g	mCherry: *n* = 10 mice<br>ChR2-mCherry: *n* = 12 mice	Two-way<br>ANOVA	Preference index:<br>Baseline: ChR2-mCherry vs. mCherry: *P* = 0.9952 n.s<br>Condition: ChR2-mCherry vs. mCherry: *P* = 0.0007 ***<br>Test: ChR2-mCherry vs. mCherry: *P* = 0.0015 **<br>Treatment effect: *F* (2, 60) = 14.40, *P* < 0.0001; Group effect: *F* (1, 60) = 20.23, *P* < 0.0001; Interaction: *F* (2, 60) = 4.273, *P* = 0.0184
6h	*n* = 8 cells	One-way<br>ANOVA	Amplitude of PSCs:<br>Before vs. PTX: *P* = 0.6727 n.s<br>Before vs. APV+CNQX: *P* = 0.0004 ****<br>Amplitude of PSCs effect: *F* (2, 21) = 11.71, *P* = 0.0004
6i	Control: *n* = 10 mice<br>MVN-NTS: *n* = 12 mice<br>MVN-vlPAG: *n* = 6 mice	Two-way<br>ANOVA	Preference index:<br>Condition: MVN-NTS vs. Control: *P* = 0.0011 **<br>MVN-vlPAG vs. Control: *P* = 0.0055 **<br>Test: MVN-NTS vs. Control: *P* = 0.0024 **<br>MVN-vlPAG vs. Control: *P* = 0.0489 *<br>MVN-NTS vs. MVN-vlPAG: *P* > 0.9999<br>Treatment effect: F (2, 75) = 27.03, P < 0.0001; Group effect: *F* (2, 75) = 12.47, *P* < 0.0001; Interaction: *F* (4, 75) = 2.903, *P* = 0.0273
6l	mCherry: *n* = 7 mice<br>hM4D-mCherry: *n* = 8 mice	Two-way<br>ANOVA	Preference index:<br>Baseline: hM4D-mCherry vs. mCherry: *P* = 0.9394 n.s<br>Condition: hM4D-mCherry vs. mCherry: *P* = 0.9458 n.s<br>Test: hM4D-mCherry vs. mCherry: *P* = 0.0001 ***<br>mCherry: Baseline vs. Condition: *P* = 0.0002 ***<br>hM4D-mCherry: Baseline vs. Condition: *P* < 0.0001 ****<br>hM4D-mCherry: Test vs. Condition: *P* = 0.0002 ***<br>Treatment effect: *F* (2, 39) = 22.84, *P* < 0.0001; Group effect: *F* (1, 39) = 4.241, *P* = 0.0462; Interaction: *F* (2, 39) = 8.637, *P* = 0.0008
7b	Rotation: *n* = 5 mice<br>Rotation + Tone: *n* = 5 mice	Two-way<br>ANOVA	Peak ∆F/F (%):<br>Anticipatory Tone: Rotation + Tone vs. Rotation: *P* = 0.0146 *<br>Non-tone: Rotation + Tone vs. Rotation: *P* = 0.7852 n.s<br>Rotation: Tone vs. Non-tone: *P* = 0.9878 n.s

			Rotation + Tone: Tone vs. Non-tone: *P* = 0.0400 *<br>Treatment effect: *F* (1, 16) = 6.850, *P* = 0.0187; Group effect: *F* (1, 16) = 3.699, *P* = 0.0724; Interaction: F (1, 16) = 2.972, *P* = 0.1040
8c	EGFP + Rotation + tone: *n* = 7 mice<br>GtACR1 + Rotation + tone: *n* = 7 mice	Two-way ANOVA	Head orientation angle / 10 s:<br>10 d: GtACR1 + Rotation + tone vs. EGFP + Rotation + tone: *P* = 0.0182 *<br>Day effect: *F* (4, 60) = 3.343, *P* = 0.0155; Group effect: *F* (1, 60) = 21.33, *P* < 0.0001; Interaction: *F* (4, 60) = 0.4259, *P* = 0.7894
8e	EGFP + Rotation + tone: *n* = 7 mice<br>GtACR1 + Rotation + tone: *n* = 7 mice	Two-way ANOVA	Average velocity / 10 s:<br>3 s: GtACR1 + Rotation + tone vs. EGFP + Rotation + tone: *P* = 0.0149 *<br>4 s: GtACR1 + Rotation + tone vs. EGFP + Rotation + tone: *P* = 0.0409 *<br>5 s: GtACR1 + Rotation + tone vs. EGFP + Rotation + tone: *P* = 0.0438 *<br>Time effect: *F* (10, 132) = 7.616, *P* < 0.0001; Group effect: *F* (1, 132) = 50.18, *P* < 0.0001; Interaction: *F* (10, 132) = 0.9689, *P* = 0.4736
8l	Rotation: *n* = 4 mice<br>Rotation + Tone: *n* = 4 mice	Two-way ANOVA	Head orientation angle / 10 s:<br>7 d: Rotation vs. Rotation + tone: *P* = 0.0435 *<br>8 d: Rotation vs. Rotation + tone: *P* = 0.0101 *<br>8 d: Rotation vs. Rotation + tone: *P* = 0.0484 *<br>Day effect: *F* (4, 50) = 0.9205, *P* = 0.4595; Group effect: *F* (1, 50) = 36.69, *P* < 0.0001; Interaction: *F* (4, 50) = 0.1135, *P* = 0.9772
8h	Rotation: *n* = 4 mice<br>Rotation + Tone: *n* = 4 mice	Two-way ANOVA	Peak ΔF/F (%):<br>Anticipatory tone: Rotation + Tone vs. Rotation: *P* = 0.0213 *<br>Rotation + Tone: Anticipatory tone vs. Non-tone: *P* = 0.0343 *<br>Treatment effect: *F* (1, 12) = 7.407, *P* = 0.0186; Group effect: *F* (1, 12) = 5.553, *P* = 0.0363; Interaction: *F* (1, 12) = 2.388, *P* = 0.1482
8o right	Rotation: *n* = 77 Cells<br>Rotation + Tone: *n* = 74 Cells	Mann-Whitney *U* test	Amplitude of IPSCs:<br>Rotation + Tone vs. Rotation: *P* = 0.0019 **, *U* = 13
S1a	1.25 g: *n* = 4 mice<br>2.5 g: *n* = 4 mice<br>5.0 g: *n* = 6 mice	One-way ANOVA	Average velocity / 10 min:<br>2.5 g vs.1.25 g: *P* =0.2936 n.s<br>2.5 g vs.1.25 g: *P* = 0.0148 *<br>5.0 g vs.1.25 g: *P* = 0.0009 ***<br>Average velocity effect: *F* (2, 11) = 14.31, *P* = 0.0009
S2a	Rotation: *n* = 8 mice	Two-way	Average velocity / 10 s:

	Rotation + Fixed tone: *n* = 8 mice<br>Rotation + Random tone: *n* = 8 mice	ANOVA	Day6: Rotation vs. Rotation + Fixed tone: *P* < 0.0001 ****<br>Rotation + Fixed tone vs. Rotation + Random tone: *P* = 0.0014 **<br>Day7: Rotation vs. Rotation + Fixed tone: *P* < 0.0001 ****<br>Rotation + Fixed tone vs. Rotation + Random tone: *P* = 0.0014 **<br>Day8: Rotation vs. Rotation + Fixed tone: *P* < 0.0001 ****<br>Rotation + Fixed tone vs. Rotation + Random tone: *P* = 0.0008 ***<br>Day9: Rotation vs. Rotation + Fixed tone: *P* < 0.0001 ****<br>Rotation + Fixed tone vs. Rotation + Random tone: *P* = 0.0002 ***<br>Day11: Rotation (Tone) vs. Rotation + Fixed tone (Non-tone): *P* = 0.0061 **<br>Rotation + Fixed tone (Non-tone) vs. Rotation + Random tone (Non-tone): *P* = 0.9519 n.s<br>Time effect: *F* (10, 231) = 13.32, *P* < 0.0001; Group effect: *F* (2, 231) = 35.84, *P* < 0.0001; Interaction: *F* (20, 231) = 1.007, *P* = 0.4546
S2b	Rotation: *n* = 8 mice<br>Rotation + Fixed tone: *n* = 8 mice<br>Rotation + Random tone: *n* = 8 mice	One-way ANOVA	Food intake / 2 h:<br>Rotation vs. Rotation + Fixed tone: *P* = 0.9093<br>Food intake effect: *F* (2, 18) = 0.5165, *P* = 0.6052
S2c	Rotation: *n* = 8 mice<br>Rotation + Fixed tone: *n* = 8 mice<br>Rotation + Random tone: *n* = 8 mice	One-way ANOVA	Body temperature / 1 h:<br>Rotation vs. Rotation + Fixed tone: *P* = 0.8228<br>Body temperature effect: *F* (2, 15) = 0.9114, *P* = 0.4231
S3c	*Crh*-IRES-Cre: *n* = 5 mice<br>*Chat*-IRES-Cre: *n* = 5 mice<br>*Cck*-IRES-Cre: *n* = 5 mice<br>*Cbln2*-IRES-Cre: *n* = 5 mice<br>*Vglut2*-IRES-Cre: *n* = 5 mice	Two-way ANOVA	Laser power of head orientation angle / 5 s:<br>10 mW: *Cbln2*-IRES-Cre vs. *Vglut2*-IRES-Cre: *P* = 0.0002 ***<br>*Cbln2*-IRES-Cre vs. *Cck*-IRES-Cre: *P* = 0.0007 ***<br>20 mW: *Cbln2*-IRES-Cre vs. *Vglut2*-IRES-Cre: *P* < 0.0001 ****<br>*Cbln2*-IRES-Cre vs. *Cck*-IRES-Cre: *P* = 0.0095 **<br>*Cbln2*-IRES-Cre: 10 mW vs. 5 mW: *P* < 0.0001 ****<br>*Cbln2*-IRES-Cre: 20 mW vs. 10 mW: *P* < 0.0001 ****<br>Power effect: *F* (2, 60) = 60.84, *P* < 0.0001; Group effect: *F* (4, 60) = 80.53, *P* < 0.0001; Interaction: *F* (8, 60) = 10.49, *P* < 0.0001
S3d	*Cbln2*-IRES-Cre: *n* = 8 mice	One-way ANOVA	Laser frequency of head orientation angle / 5 s:<br>10 Hz vs. 5 Hz: *P* = 0.0006 ***<br>20 Hz vs.10 Hz: *P* < 0.0001 ****

			Frequency effect: F (2, 21) = 48.73, P < 0.0001
S3g	Cck-hM3D: n = 4 mice<br>Cbln2-hM3D: n = 4 mice	Two-way ANOVA	Temperature / 150 min:<br>Saline: Cbln2-hM3D vs. Cck-hM3D: P > 0.9999 n.s<br>CNO: Cbln2-hM3D vs. Cck-hM3D: P = 0.0059 **<br>Cbln2-hM3D: CNO vs. Saline: P = 0.0001 ****<br>Cck-hM3D: CNO vs. Saline: P < 0.0001 ****<br>Treatment effect: F (1, 6) = 295.1, P < 0.0001; Group effect: F (1, 6) = 5.882, P = 0.0515; Interaction: F (1, 6) = 8.304, P = 0.0280
S3h	Cck-hM3D: n = 4 mice<br>Cbln2-hM3D: n = 7 mice	Two-way ANOVA	Average velocity / 30 min:<br>Saline: Cbln2-hM3D vs. Cck-hM3D: P = 0.0769 n.s<br>CNO: Cbln2-hM3D vs. Cck-hM3D: P = 0.3816 n.s<br>Cbln2-hM3D: CNO vs. Saline: P = 0.0024 **<br>Cck-hM3D: CNO vs. Saline: P = 0.0002 ****<br>Treatment effect: F (1, 9) = 68.05, P < 0.0001; Group effect: F (1, 9) = 0.3896, P = 0.5480; Interaction: F (1, 9) = 7.012, P = 0.0266
S3i	Cck-hM3D: n = 4 mice<br>Cbln2-hM3D: n = 7 mice	Two-way ANOVA	Rotation numbers / 30 min:<br>Saline: Cbln2-hM3D vs. Cck-hM3D: P = 0.9994 n.s<br>CNO: Cbln2-hM3D vs. Cck-hM3D: P < 0.0001 ****<br>Cbln2-hM3D: CNO vs. Saline: P < 0.0001 ****<br>Treatment effect: F (1, 9) = 25.59, P = 0.0007; Group effect: F (1, 9) = 24.70, P = 0.0008; Interaction: F (1, 9) = 26.50, P = 0.0006
S4c	Cbln2-EGFP: n = 6 mice<br>Cbln2-taCasp3: n = 6 mice	Two-way ANOVA	Average velocity / 10 s:<br>Day6: 9 s: Cbln2-taCasp3 vs. Cbln2-EGFP: P = 0.0344 *<br>Day7: 5 s: Cbln2-taCasp3 vs. Cbln2-EGFP: P = 0.0127 *<br>8 s: Cbln2-taCasp3 vs. Cbln2-EGFP: P = 0.0342 *<br>Day8: 9 s: Cbln2-taCasp3 vs. Cbln2-EGFP: P = 0.0461 *<br>Day9: 9 s: Cbln2-taCasp3 vs. Cbln2-EGFP: P = 0.0009 ***<br>10 s: Cbln2-taCasp3 vs. Cbln2-EGFP: P = 0.0386 *<br>Day effect: F (3.865, 38.65) = 12.64, P < 0.0001; Group effect: F (1, 10) = 13.58, P = 0.0042; Interaction: F (10, 100) = 1.616, P = 0.1127
S4d	Cbln2-EGFP: n = 6 mice<br>Cbln2-taCasp3: n = 6 mice	Mann-Whitney U test	Food intake / 2 h:<br>Cbln2-taCasp3 vs. Cbln2-EGFP: P = 0.4286 n.s, U = 12.50
S5c	Ventral: n = 4 mice<br>Dorasl: n = 4 mice	Wilcoxon signed-rank test	Peak ΔF/F (%): Ventral vs. Doras: P = 0.1250 n.s<br>Unit AUC (a.u.): Ventral vs. Doras: P = 0.2500 n.s
S6b	Cck-IRES-Cre: n = 4 mice<br>Cbln2-IRES-Cre: n = 4 mice	Two-way ANOVA	Peak ΔF/F (%):<br>Vertical: *Cbln2*-IRES-Cre vs. *Cck*-IRES-Cre: P < 0.0001 ****<br>Horizontal: *Cbln2*-IRES-Cre vs. *Cck*-IRES-Cre: P = 0.0222 *

			Cbln2-IRES-Cre: Vertical vs. Horizontal: *P* = 0.0008 ***<br>Unit AUC (a.u.):<br>Vertical: *Cbln2*-IRES-Cre vs. *Cck*-IRES-Cre: *P* = 0.0011 ***<br>Horizontal: *Cbln2*-IRES-Cre vs. *Cck*-IRES-Cre: *P* = 0.0152 *<br>Peak ∆F/F effect: *F* (1, 12) = 13.26, *P* = 0.0034; Group effect: *F* (1, 12) = 55.61, *P* < 0.0001; Interaction: *F* (1, 12) = 10.37, *P* = 0.0074<br>Unit AUC (a.u.) effect: *F* (1, 12) = 2.725, *P* = 0.1247; Group effect: *F* (1, 12) = 30.99, *P* = 0.0001; Interaction: *F* (1, 12) = 1.087, *P* = 0.3178
S8a	mCherry: *n* = 8 mice<br>ChR2-mCherry: *n* = 8 mice	Two-way ANOVA	Laser power and frequency of head orientation angle / 5 s:<br>Laser power: 10 mW vs. 5 mW: *P* = 0.0462 *<br>20 mW vs. 10 mW: *P* = 0.0088 **<br>Laser frequency: 10 mW vs. 5 mW: *P* = 0.0074 **<br>20 mW vs. 10 mW: *P* = 0.0006 ***<br>Power effect: *F* (2, 42) = 7.993, *P* = 0.0011; Group effect: *F* (1, 42) = 112.3, *P* < 0.0001; Interaction: *F* (2, 42) = 7.741, *P* = 0.0014<br>Frequency effect: *F* (2, 42) = 14.73, *P* < 0.0001; Group effect: *F* (1, 42) = 129.2, *P* < 0.0001; Interaction: *F* (2, 42) = 11.99, *P* < 0.0001
S8c	MVN-IPN: *n* = 4 mice<br>MVN-CPU: *n* = 4 mice<br>MVN-VLPAG: *n* = 8 mice	Two-way ANOVA	Laser power of head orientation angle / 5 s:<br>5 mW: MVN-VLPAG vs. MVN-IPN: *P* = 0.0256 *<br>MVN-VLPAG vs. MVN-Cpu: *P* = 0.0150 **<br>10 mW: MVN-VLPAG vs. MVN-IPN: *P* = 0.0003 ****<br>MVN-VLPAG vs. MVN-Cpu: *P* < 0.0001 ****<br>MVN-VLPAG vs. MVN-NTS: *P* = 0.0211 *<br>20 mW: MVN-VLPAG vs. MVN-IPN: *P* < 0.0001 ****<br>MVN-VLPAG vs. MVN-Cpu: *P* < 0.0001 ****<br>MVN-VLPAG vs. MVN-NTS: *P* = 0.0001 ***<br>Power effect: *F* (2, 48) = 16.63, *P* < 0.0001; Group effect: *F* (3, 48) = 49.39, *P* < 0.0001; Interaction: *F* (6, 48) = 1.624, *P* = 0.1611
S8g	fDIO-EGFP: *n* = 6 mice<br>fDIO-taCasp3: *n* = 6 mice	Two-way ANOVA	Average velocity / 10 s:<br>Day6: 6 s: taCasp3-EGFP vs. EGFP: *P* = 0.0113 *<br>10 s: taCasp3-EGFP vs. EGFP: *P* = 0.0123 *<br>Day7: 9 s: taCasp3-EGFP vs. EGFP: *P* = 0.0053 **<br>Day8: 5 s: taCasp3-EGFP vs. EGFP: *P* = 0.0013 **<br>Day9: 5 s: taCasp3-EGFP vs. EGFP: *P* = 0.0060 **<br>8 s: taCasp3-EGFP vs. EGFP: *P* = 0.0363 *<br>Day effect: *F* (2, 48) = 16.63, *P* < 0.0001; Group effect: *F* (3, 48) = 49.39, *P* < 0.0001; Interaction: *F* (6, 48) = 1.624, *P* = 0.1611

S9c	Ventral: *n* = 4 mice<br>Dorsal: *n* = 4 mice	Wilcoxon signed-rank test	Peak ΔF/F (%): Ventral vs. Back-facing: *P* = 0.1250 n.s<br>Unit AUC (a.u.): Ventral vs. Back-facing: *P* = 0.1250 n.s
S11e	mCherry + Rotation: *n* = 7 mice<br>ChR2 + Rotation: *n* = 7 mice	Two-way ANOVA	Head orientation angle / 10 s:<br>8 d: ChR2 + Rotation vs. mCherry + Rotation: *P* = 0.0255 *<br>10 d: ChR2 + Rotation vs. mCherry + Rotation: *P* = 0.0023 **<br>Day effect: *F* (4, 60) = 2.317, *P* = 0.0674; Group effect: *F* (1, 60) = 26.43, *P* < 0.0001; Interaction: *F* (4, 60) = 1.555, *P* = 0.1979
S11g	mCherry + Rotation: *n* = 7 mice<br>ChR2 + Rotation: *n* = 7 mice	Two-way ANOVA	Average velocity / 10 s:<br>Day8: 6 s: ChR2 + Rotation vs. mCherry + Rotation: *P* = 0.0247 *<br>Day10: 8 s: ChR2 + Rotation vs. mCherry + Rotation: *P* = 0.0142 *<br>9 s: ChR2 + Rotation vs. mCherry + Rotation: *P* = 0.0179 *<br>Day effect: *F* (10, 132) = 8.635, *P* < 0.0001; Group effect: *F* (1, 132) = 52.87, *P* < 0.0001; Interaction: *F* (10, 132) = 0.8449, *P* = 0.5865
S12b	Rotation: *n* = 6 mice<br>Rotation + Tone: *n* = 6 mice	Two-way ANOVA	Average velocity / 10 s:<br>Day6: 7 s: Rotation vs. Rotation + Tone: *P* = 0.0023 **<br>10 s: Rotation vs. Rotation + Tone: *P* = 0.0306 *<br>Day7: 4 s: Rotation vs. Rotation + Tone: *P* = 0.0474 *<br>7 s: Rotation vs. Rotation + Tone: *P* = 0.0057 **<br>Day8: 2 s: Rotation vs. Rotation + Tone: *P* = 0.0183 *<br>6 s: Rotation vs. Rotation + Tone: *P* = 0.0150 *<br>8 s: Rotation vs. Rotation + Tone: *P* = 0.0192 *<br>Day10: 5 s: Rotation vs. Rotation + Tone: *P* = 0.0456 *<br>Day effect: *F* (10, 110) = 5.261, *P* < 0.0001; Group effect: *F* (1, 110) = 27.35, *P* < 0.0001; Interaction: *F* (10, 110) = 0.9815, *P* = 0.4638
S12d	Rotation: *n* = 78 Cells<br>Rotation + Tone: *n* = 75 Cells	Mann-Whitney *U* test	Amplitude of EPSCs:<br>Rotation + Tone vs. Rotation: *P* = 0.1901 n.s, *U* = 39
