# Supplementary material for: The Neural Mechanism of Motion Sickness Alleviation by Anticipatory Sound
Source: Nat Commun. 2026 Sep 24;17:11109. doi: 10.1038/s41467-026-77950-x (PMC13696722; doi:10.1038/s41467-026-77950-x)
Supplement: Supplementary file 3 [file 41467_2026_77950_MOESM2_ESM.pdf]

Description of Additional Supplementary Files

Supplementary Movie 1. Repeated rotation-induced MS-like behavior.
Repeated rotation-induced MS-like behavior in a representative WT mouse on day 6, shown together with the open-field recording and movement trajectory.

Supplementary Movie 2. Optogenetic activation of Cbln2$^+$ MVN neurons.
Optogenetic activation of Cbln2$^+$ MVN neurons using 20 Hz light stimulation at 10 mW evoked MS-like circling behavior in a representative mouse expressing AAV-DIO-ChR2-mCherry in the MVN, with an optical fiber implanted above the MVN.

Supplementary Movie 3. Chemogenetic activation of Cbln2$^+$ MVN neurons. Chemogenetic activation of Cbln2$^+$ MVN neurons evoked pronounced MS-like circling behavior in a representative mouse expressing AAV-DIO-hM3Dq-mCherry in the MVN.

Supplementary Movie 4. Chemogenetic activation of Cck$^+$ MVN neurons. Chemogenetic activation of Cck$^+$ MVN neurons evoked MS-like body tottering in a representative mouse expressing AAV-DIO-hM3Dq-mCherry in the MVN.

Supplementary Movie 5. Calcium activity of Cbln2$^+$ MVN neurons during rotation-induced MS-like behavior.
Fiber photometry recording of GCaMP signals from Cbln2$^+$ MVN neurons during ventral rotation-induced MS-like behavior in a representative mouse. The calcium signal increased in temporal association with the rotation-induced behavioral response.

Supplementary Movie 6. Optogenetic activation of the Cbln2$^+$ MVN-vlPAG pathway. Optogenetic activation of the Cbln2$^+$ MVN-vlPAG pathway using 20 Hz light stimulation at 10 mW evoked MS-like circling behavior in a representative mouse.
