# Supplementary material for: The Neural Mechanism of Motion Sickness Alleviation by Anticipatory Sound
Source: Nat Commun. 2026 Sep 24;17:11109. doi: 10.1038/s41467-026-77950-x (PMC13696722; doi:10.1038/s41467-026-77950-x)
Supplement: Supplementary file 4 [file 41467_2026_77950_MOESM9_ESM.pdf]

nature portfolio

Corresponding author(s): Congping Shang

Last updated by author(s): May 02, 2026

Reporting Summary

Nature Portfolio wishes to improve the reproducibility of the work that we publish. This form provides structure for consistency and transparency in reporting. For further information on Nature Portfolio policies, see our Editorial Policies and the Editorial Policy Checklist.

Statistics

For all statistical analyses, confirm that the following items are present in the figure legend, table legend, main text, or Methods section.

n/a	Confirmed	
☐	☒	The exact sample size (*n*) for each experimental group/condition, given as a discrete number and unit of measurement
☐	☒	A statement on whether measurements were taken from distinct samples or whether the same sample was measured repeatedly
☐	☒	The statistical test(s) used AND whether they are one- or two-sided<br>*Only common tests should be described solely by name; describe more complex techniques in the Methods section.*
☒	☐	A description of all covariates tested
☐	☒	A description of any assumptions or corrections, such as tests of normality and adjustment for multiple comparisons
☐	☒	A full description of the statistical parameters including central tendency (e.g. means) or other basic estimates (e.g. regression coefficient) AND variation (e.g. standard deviation) or associated estimates of uncertainty (e.g. confidence intervals)
☐	☒	For null hypothesis testing, the test statistic (e.g. *F*, *t*, *r*) with confidence intervals, effect sizes, degrees of freedom and *P* value noted<br>*Give P values as exact values whenever suitable.*
☒	☐	For Bayesian analysis, information on the choice of priors and Markov chain Monte Carlo settings
☒	☐	For hierarchical and complex designs, identification of the appropriate level for tests and full reporting of outcomes
☒	☐	Estimates of effect sizes (e.g. Cohen's *d*, Pearson's *r*), indicating how they were calculated

Our web collection on statistics for biologists contains articles on many of the points above.

Software and code

Policy information about availability of computer code

Data collection	Behavioral videos were recorded using commercial camera (Wexinshijie tech, #WX200). To deliver tone stimuli, broadband white noise was generated with Python 3.11 custom scripts, BatSound 4.0 software, and a free-field speaker. Custom scripts is presented in Zenodo at https://doi.org/10.5281/zenodo.17426008.<br>For slice physiological recording, the current and voltage signals were recorded with MultiClamp 700B and Clampex 10 data acquisition software (Molecular Devices, USA).<br>For fiber photometry, fluorescence signals were acquired with a multi-channel fiber photometry system (Thinker Tech, China).<br>Images were captured on a Zeiss LSM980 confocal microscope or Olympus VS200 Virtual Microscopy Slide Scanning System.
Data analysis	For behavior experiments, mice behavioral parameters and body temperature were analyzed using VisuTrack-AI and FOTRIC AnalyzIR software, respectively.<br>For slice physiological recording, the data was analyzed using Clampfit 10.4.<br>For fiber photometry data analysis, raw data was analyzed using Spike2 Version 7.03 and MATLAB R2017b. MATLAB code is presented in Zenodo at https://doi.org/10.5281/zenodo.17425988.<br>Microscope imaging data were analyzed using OlyVIA V4.1 (Olynpus, Japan), ZEN2012 black/blue edition, or ImageJ (FIJI) (version 2.0.0).<br>All data was replotted and statistical analyzed using GraphPad Prism 8.

For manuscripts utilizing custom algorithms or software that are central to the research but not yet described in published literature, software must be made available to editors and reviewers. We strongly encourage code deposition in a community repository (e.g. GitHub). See the Nature Portfolio guidelines for submitting code & software for further information.

Data

Policy information about availability of data

All manuscripts must include a data availability statement. This statement should provide the following information, where applicable:

- Accession codes, unique identifiers, or web links for publicly available datasets
- A description of any restrictions on data availability
- For clinical datasets or third party data, please ensure that the statement adheres to our policy

All data supporting the findings of this study are provided in the paper and its Supplementary Information. Source data are provided with this paper.

Research involving human participants, their data, or biological material

Policy information about studies with human participants or human data. See also policy information about sex, gender (identity/presentation), and sexual orientation and race, ethnicity and racism.

Reporting on sex and gender	NA
Reporting on race, ethnicity, or other socially relevant groupings	NA
Population characteristics	NA
Recruitment	NA
Ethics oversight	NA

Note that full information on the approval of the study protocol must also be provided in the manuscript.

Field-specific reporting

Please select the one below that is the best fit for your research. If you are not sure, read the appropriate sections before making your selection.

- [x] Life sciences
- [ ] Behavioural & social sciences
- [ ] Ecological, evolutionary & environmental sciences

For a reference copy of the document with all sections, see nature.com/documents/nr-reporting-summary-flat.pdf

Life sciences study design

All studies must disclose on these points even when the disclosure is negative.

Sample size	No formal statistical method was used to predetermine sample sizes. Sample sizes were chosen based on preliminary experiments, our experience with the expected variability of the measurements, and sample sizes commonly used in previous studies employing similar experimental paradigms. These sample sizes were considered sufficient to obtain reproducible results across biologically independent animals while minimizing animal use.
Data exclusions	For behavioral analyses, the mice with missed viral injections/optical fiber and unhealthy conditions were excluded for experimental analyses. For whole-cell recordings, the neurons with Ra larger than 15 MΩ were excluded from further analyses, because Ra larger than 15 MΩ indicates poor patch clamp.
Replication	Behavioral experiments are replicated multiples times with independent mice, and two people independently analyzed the behavioral data. All the experimental recordings were reliably reproduced. Numbers of replicates (n) are indicated in the figure legends.
Randomization	All mice were randomly assigned to different groups.
Blinding	All investigators were blinded to group allocation during data collection and analysis.

Reporting for specific materials, systems and methods

We require information from authors about some types of materials, experimental systems and methods used in many studies. Here, indicate whether each material, system or method listed is relevant to your study. If you are not sure if a list item applies to your research, read the appropriate section before selecting a response.

Materials & experimental systems

n/a	Involved in the study
[ ]	[x] Antibodies
[x]	[ ] Eukaryotic cell lines
[x]	[ ] Palaeontology and archaeology
[ ]	[x] Animals and other organisms
[x]	[ ] Clinical data
[x]	[ ] Dual use research of concern
[x]	[ ] Plants

Methods

n/a	Involved in the study
[x]	[ ] ChIP-seq
[x]	[ ] Flow cytometry
[x]	[ ] MRI-based neuroimaging

Antibodies

Antibodies used	Primary antibodies:<br>rabbit anti-c-Fos (CST, clone 9F6, #2250S, dilution 1:500); chicken anti-mCherry (Abcam, #ab205402, dilution 1:2000); rabbit anti-EGFP (Abcam, #ab290, dilution 1:2000); rabbit anti-GABA (Sigma-Aldrich, #A2052, dilution 1:500), rabbit anti-glutamate (Sigma-Aldrich, #G6642, dilution 1:500).<br>Secondary antibodies:<br>goat anti-rabbit, Alexa Fluor 488 (Invitrogen, Cat# A-11034, dilution 1:500); goat anti-chicken, Alexa Fluor Plus 555 (Thermo Fisher cientific, Cat# A-32932, dilution 1:500).
Validation	All antibodies are commonly used in the field and have been validated by the manufacturer. All antibodies listed below were validated for immunohistochemical use in rodent in previously published work: PMID: 38288368.

Animals and other research organisms

Policy information about studies involving animals; ARRIVE guidelines recommended for reporting animal research, and Sex and Gender in Research

Laboratory animals	All animals used in this study were mice (Mus musculus), as described in the Methods (Mice) section. C57BL/6J mice (#000013) were obtained from the Guangdong GemPharmatech Co., Ltd (Foshan, China). Chat-IRES-Cre (#006410), Crh-IRES-Cre (#012704), Cck-IRES-Cre (#012706), Vglut2-IRES-Cre (#028863), and Vgat-IRES-Cre (#028862) mouse lines were obtained from the Jackson Laboratory (JAX Mice and Services). The Cbln2-IRES-Cre mouse line was obtained from the Professor Peng Cao of NIBS. All mouse lines were maintained on a C57BL/6J genetic background. Mice were housed at room temperature (23 ± 1°C) with a stable humidity (50 ± 5%) on a 12-h light/12-h dark cycle. Mice were fed with ad libitum standard chow and water, unless food-deprived overnight for 16–18h for fasted experiments. Mice used in individual experiments were 8–12 weeks old and were at least 8 weeks old at the time of viral injection. After virus injection, mice were individually housed for 2-3 weeks before subsequent experiments.
Wild animals	No wild animals were used in this study.
Reporting on sex	Male mice were used in all experiments to minimize sex-related variability, given sex-dependent differences in motion-sickness susceptibility in humans and rodent models.
Field-collected samples	No field-collected samples were used in this study.
Ethics oversight	All experimental procedures were carried out in strict compliance with protocols approved by the Administrative Panel on Laboratory Animal Care at the Guangzhou laboratory (Guangzhou, China; approval No. GZLAB-AUCP-2024-04-A10).

Note that full information on the approval of the study protocol must also be provided in the manuscript.

Plants

Seed stocks	*Report on the source of all seed stocks or other plant material used. If applicable, state the seed stock centre and catalogue number. If plant specimens were collected from the field, describe the collection location, date and sampling procedures.*
Novel plant genotypes	*Describe the methods by which all novel plant genotypes were produced. This includes those generated by transgenic approaches, gene editing, chemical/radiation-based mutagenesis and hybridization. For transgenic lines, describe the transformation method, the number of independent lines analyzed and the generation upon which experiments were performed. For gene-edited lines, describe the editor used, the endogenous sequence targeted for editing, the targeting guide RNA sequence (if applicable) and how the editor was applied.*
Authentication	*Describe any authentication procedures for each seed stock used or novel genotype generated. Describe any experiments used to assess the effect of a mutation and, where applicable, how potential secondary effects (e.g. second site T-DNA insertions, mosiacism, off-target gene editing) were examined.*
